# A Silyl Sulfinylamine Reagent Enables the Modular Synthesis of Sulfonimidamides via Primary Sulfinamides

**DOI:** 10.1021/acs.orglett.2c00347

**Published:** 2022-02-21

**Authors:** Mingyan Ding, Ze-Xin Zhang, Thomas Q. Davies, Michael C. Willis

**Affiliations:** Department of Chemistry, University of Oxford, Chemistry Research Laboratory, Mansfield Road, Oxford, OX1 3TA, U.K.

## Abstract

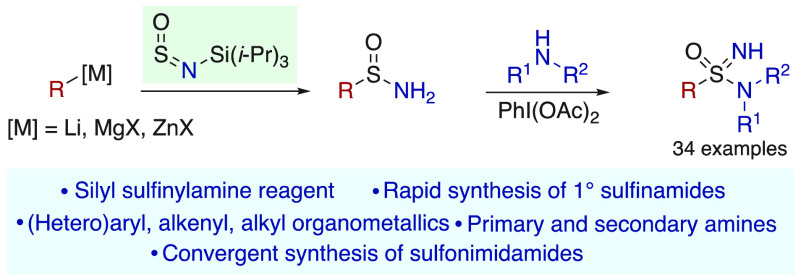

A new *N*-silyl sulfinylamine reagent allows the rapid preparation of a broad range of (hetero)aryl, alkenyl, and alkyl primary sulfinamides, using Grignard, organolithium, or organozinc reagents to introduce the carbon fragment. Treatment of these primary sulfinamides with an amine in the presence of a hypervalent iodine reagent leads directly to NH-sulfonimidamides. This two-step sequence is straightforward to perform and provides a modular approach to sulfonimidamides, allowing ready variation of both reaction components, including primary and secondary amines.

Sulfonimidamides^1^ are becoming established as valuable motifs in medicinal chemistry^2^ and feature in molecules used in an increasing range of therapeutic areas.^3^ The growth in use of sulfonimidamides has been mirrored by recent innovations^4^ in their synthesis.^5^ Approaches that employ sulfonimidoyl halides,^6^ or sulfonimidates,^7^ have been used extensively; however, access to these substrates can be challenging. The imination, or imination/oxidation, of lower oxidation-state precursors have emerged as useful methods to access sulfonimidamides. In this context, Bull has shown that an iodosobenzene/ammonium carbamate combination can be used to convert tertiary sulfenamides directly to sulfonimidamides,^8^ and Stockman has employed related reagents with tertiary sulfinamide substrates (Scheme 1a,b).^9^ Both of these methods are efficient, and both show commendable scope. However, both approaches are essentially linear; the last step in each is installation of an imidic NH group to a functionalized precursor, where the key S–N bond, linking the S-fragment and the N-fragment, has been established earlier in the reaction sequence. To provide more convergency, and to enable analogue synthesis, we conceived of an approach to NH-sulfonimidamides in which primary sulfinamides are combined with diversely substituted amines, using a hypervalent iodine reagent, as the final step of the synthesis (Scheme 1d). Our confidence in the success of this final step was due to in part to the chemistry from Bull, and Stockman, but also to the pioneering work from Malacria and Fensterbank, who converted primary sulfinamides into sulfonimidates using iodosobenzene with alcoholic solvents (Scheme 1c).^10^

**Scheme 1:**
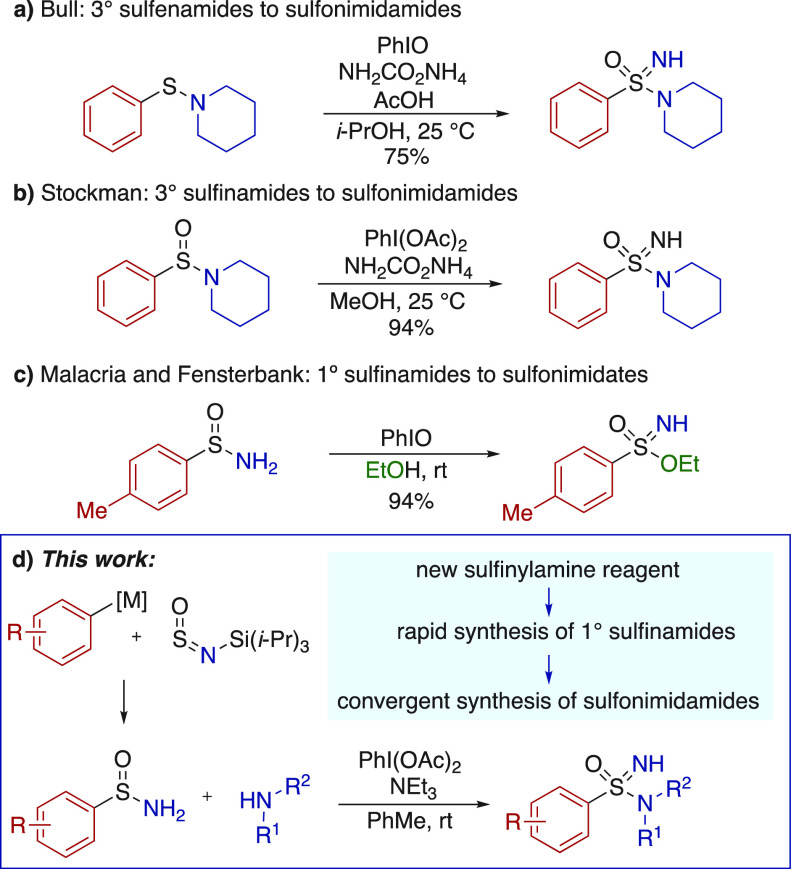
(a–c) Hypervalent-Iodine Mediated Synthesis of Sulfonimidamides and Sulfonimidates; (d) *This Work:* A Modular Route to Sulfonimidamides

To deliver a flexible, fully modular sulfonimidamide synthesis, our approach would also require a straightforward method to access primary sulfinamides. Although a number of primary sulfinamide syntheses are known, most require several steps,^11^ or the need to use thiol substrates;^12^ we wished to avoid both of these constraints. To address this, we proposed the combination of a suitable sulfinylamine reagent with an organometallic nucleophile, which should directly provide the required primary sulfinamides (Scheme 1d). Herein, we report the successful realization of this plan.

Although sulfinylamines (R-NSO) have been known for over 140 years,^13^ there has only recently been a flurry of activity using these reagents.^14,5d,6a,7a,15^ For our proposed synthesis we required a sulfinylamine reagent with an N-substituent that would be easily removed using mild conditions, ideally avoiding strongly acidic or basic media. To meet these requirements, we settled on a *N*-silyl substituted reagent, and initially considered the *N*-triphenylsilyl derivative, originally prepared by Ismail and co-workers.^16^ However, it was soon apparent that the *N*-triphenylsilyl sulfinylamine was prone to hydrolysis, and its use after storage was challenging. To achieve the desired balance between stability and reactivity, we turned to the *N*-triisopropylsilyl derivative **1**. This novel sulfinylamine could be prepared on a multigram scale, in high yield, in two steps starting from triisopropylsilyl chloride and ammonia (Scheme 2). Reagent **1** is a light-yellow colored liquid that is stable to refrigerated storage for at least one month.^17^

**Scheme 2:**
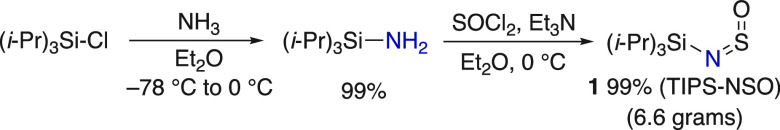
Preparation of Triisopropylsilyl Sulfinylamine **1**

Sulfinylamine **1** (TIPS-NSO) showed good reactivity with a broad range of Grignard, organolithium, and organozinc reagents (Scheme 3). Although the intermediate *N*-silyl sulfinamides could be isolated, it was more convenient to treat the reaction mixtures directly with TBAF to form the desired primary sulfinamides. In this way, aryl organometallics of varied steric (**2a**–**2c**) and electronic character (**2d**–**2f**) could be smoothly converted to the corresponding primary sulfinamides in excellent yields. A gram-scale reaction provided *p*-fluoro-derivative **2d** in 89% yield. Heteroaryl organometallics derived from pyridine, thiophene, and benzofuran could be employed (**2g**–**2j**). Alkyl sulfinamides could also be prepared, with representative primary, benzylic, secondary, and tertiary Grignard reagents being used (**2k**–**2n**). An alkenyl-Grignard reagent was also successful (**2o**). Finally, the aryl core of the COX-2 inhibitor Celecoxib was incorporated (**2p**).

**Scheme 3:**
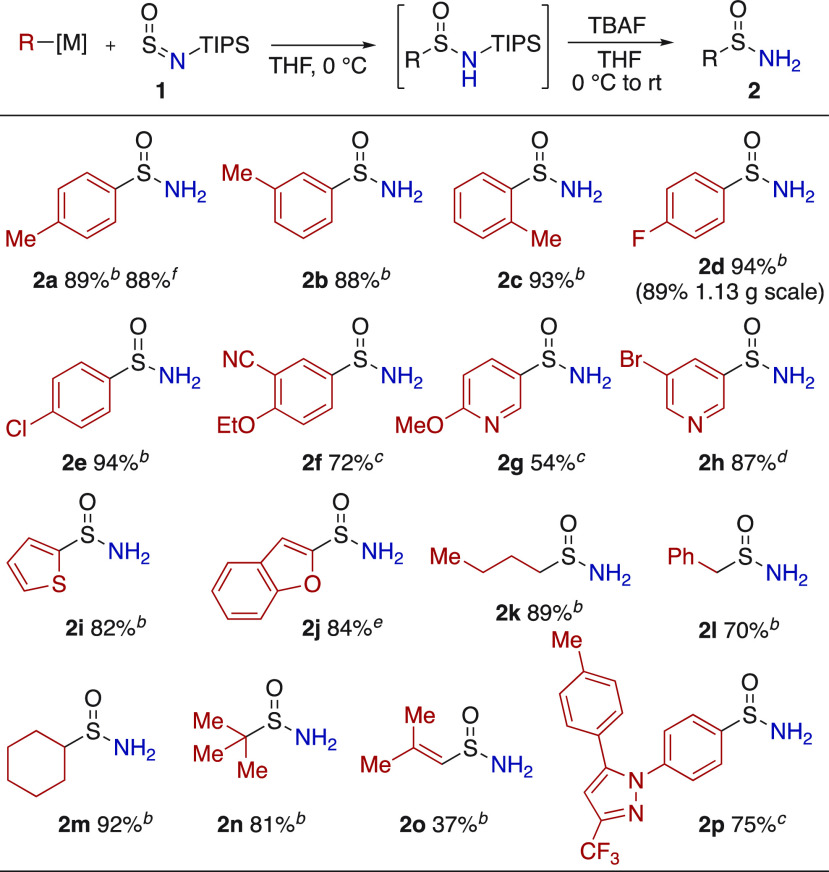
Synthesis of Primary Sulfinamides **2** Reaction conditions: TIPS-NSO (1.00 equiv), R-[M] (1.20 equiv), THF (0.1 M), 0 °C, 5 min; *then* TBAF (2.00 equiv), 0 °C to rt, 10 min. Isolated yields. Commercial Grignard reagent. Organolithium reagent, generated using lithium-halogen exchange (*n*-BuLi). Grignard reagent, generated from aryl bromide using *i*-Pr-MgCl.LiCl. Organolithium reagent, generated by deprotonation, using *n*-BuLi. Using Ar-ZnCl, generated from Grignard reagent and ZnCl_2_.

With a selection of primary sulfinamides readily available, our attention turned to their conversion into sulfonimidamides. An efficient procedure was established, involving treating the sulfinamide with the desired amine in the presence of 1.5 equiv of PhI(OAc)_2_, using triethylamine as base (Scheme 4). Applying this procedure, using morpholine as the amine component, allowed all of the primary sulfinamides shown in Scheme 3 to be smoothly converted into the corresponding sulfonimidamides. The reactions were performed at ambient temperature, for between 1.5 and 23 h. The *para*-fluoro example (**3d**) was prepared on a gram scale (2.3 g) in an identical yield (92%) to that achieved in the smaller-scale scoping experiments. The *para*-fluorophenyl sulfinamide (**2d**) was then used to explore the range of secondary amines that could be used. Cyclic examples substituted with cyano and ketone groups performed well (**3q**, **3r**). Acyclic amines featuring pyridyl and cyclohexylmethyl groups were also included (**3s**, **3t**). The remainder of the amines used were selected as they feature in marketed pharmaceuticals (Clopidogrel **3u**, Perospirone **3v**, Buspirone **3w**, Amoxapine **3x**), and as can be seen, these more complex, heterocylic scaffolds provided the desired sulfonimidamides in generally excellent yields.

**Scheme 4:**
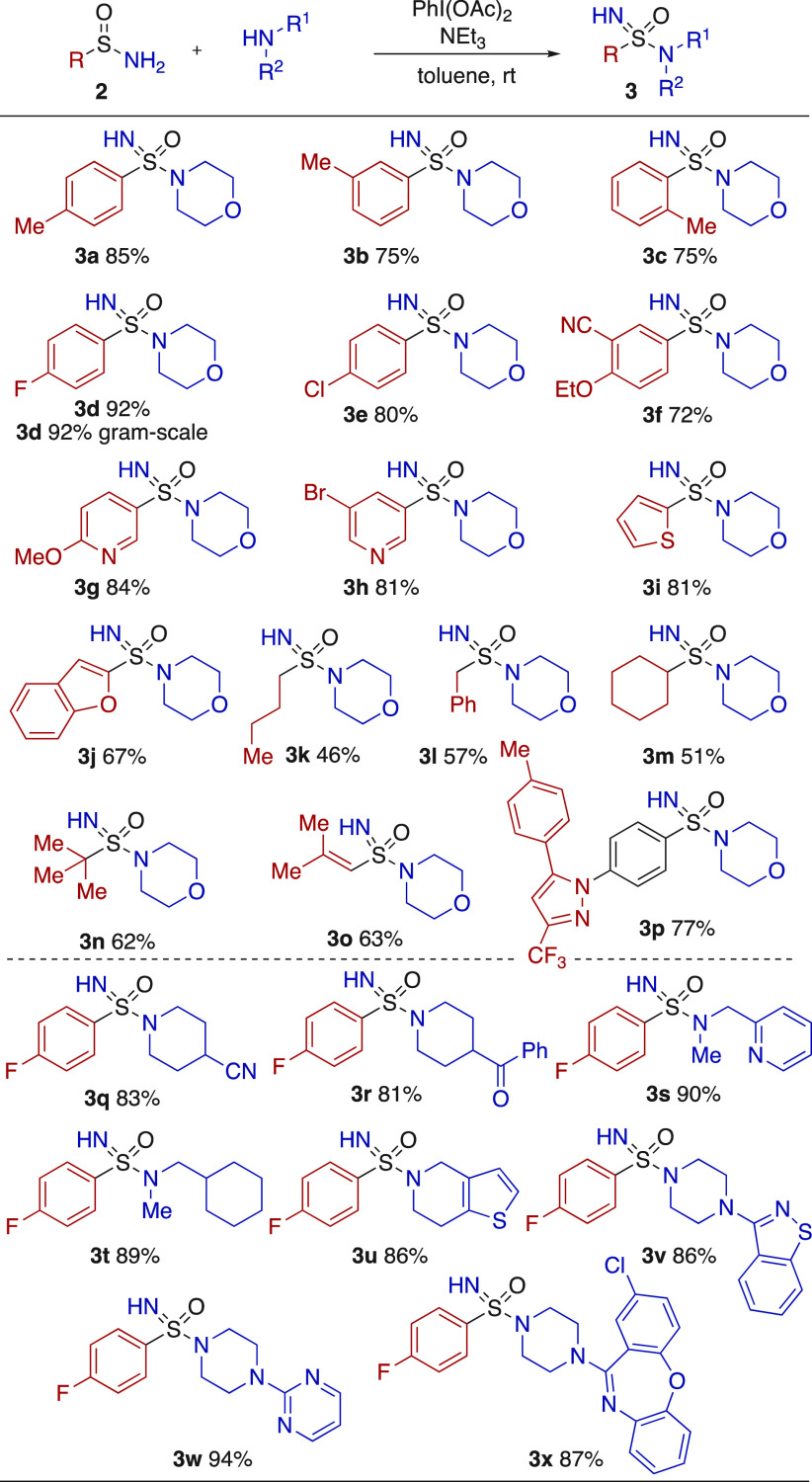
Conversion of Primary Sulfinamides into Sulfonimidamides, Using Secondary Amine Nucleophiles Reaction conditions: sulfinamide **2a**–**p** (1.0 equiv), amine (1.5 equiv), PhI(OAc)_2_ (1.5 equiv), Et_3_N (3.0 equiv), toluene (0.1 M), rt. Isolated yields.

All of the amines used in Scheme 4 are secondary amines; primary amines were poor substrates using the original reaction conditions, generally providing only 5–10% of product. After optimization (see Supporting Information), we were able to identify suitable conditions for primary amine substrates. The new conditions required the use of the more robust hypervalent iodine reagent PhI(OC(O)*t*-Bu)_2_,^18^ a greater excess of triethylamine, and an increased reaction temperature of 60 °C. These modified conditions were successfully applied to a range of primary amines, including primary alkyl (**4a**–**c**) and secondary alkyl (**4d**, **4e**), using the *para*-fluorophenyl sulfinamide (**2d**) as the substrate. Amines featuring alkyne (**4f**, **4g**) and alkene (**4h**) groups could be used (Scheme 5). The final example establishes that these new conditions could also be extended to a heterocyclic sulfinamide substrate (**4i**). The successful use of primary amines to prepare the corresponding sulfonimidamides is notable, as neither the Bull nor Stockman approaches shown in Scheme 1 could accommodate this class of amine.^8,9^

**Scheme 5:**
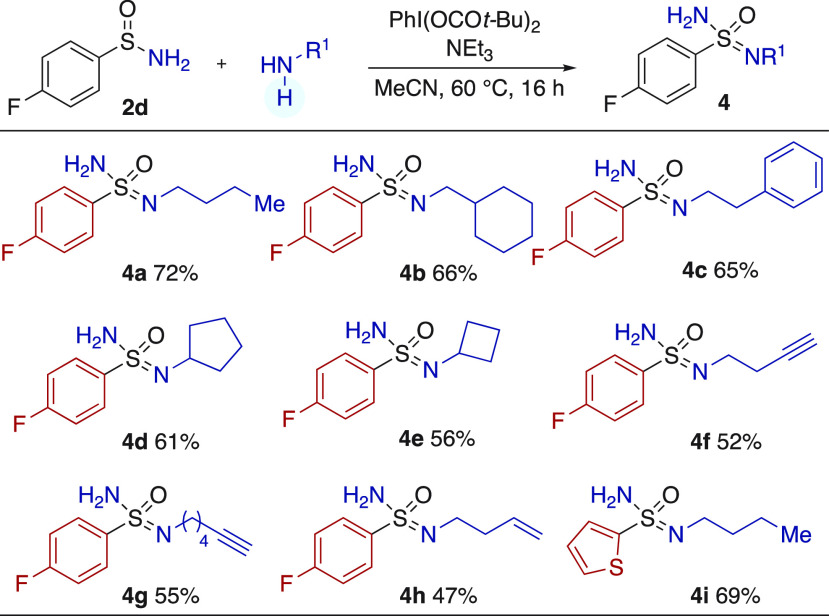
Conversion of Primary Sulfinamides into Sulfonimidamides, Using Primary Amine Nucleophiles Reaction conditions: sulfinamide (1.0 equiv), amine (2.0 equiv), PhI(OC(O)*t*-Bu)_2_ (2.5 equiv), Et_3_N (12.0 equiv), MeCN (0.1 M), 60 °C, 16 h. Isolated yields.

As a final demonstration of the utility of the developed method, we targeted the preparation of a more complex sulfonimidamide derivative (Scheme 6). Pyrimidinone-substituted aryl bromide **5** contains the aryl core of the marketed PDE5-inhibitor Sildenafil. Lithiation of bromide **5** using a combination of MeLi and *n*-BuLi generated an aryl lithium reagent that underwent smooth addition into TIPS-NSO. In situ treatment with TBAF then provided complex primary sulfinamide **6** in an excellent 89% yield. The coupling of sulfinamide **6** with *N*-methyl piperazine required the use of DBU in place of triethylamine and MeCN as solvent; with these modifications, sulfonimidamide **7**,^7a^ the monoaza analogue of Sildenafil, was isolated in 64% yield.

**Scheme 6:**
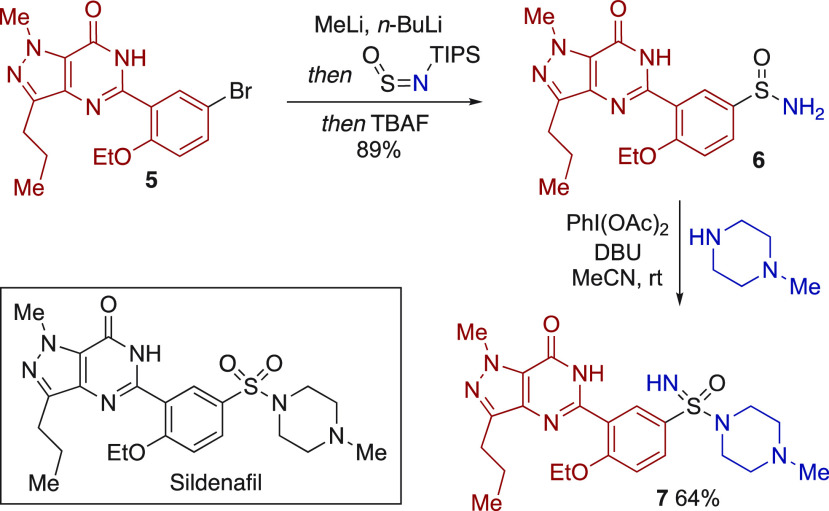
Synthesis of Sildenafil Analogue **7**

In summary, we have developed a modular, two-step synthesis of sulfonimidamides, with organometallics such as Grignard, organolithium, or organozinc reagents, and amines, being the key building blocks. This strategy alleviates the necessity of thiol starting materials. A new *N*-silyl sulfinylamine reagent is introduced that allows ready preparation of a broad range of primary sulfinamides. The convergent nature of this approach should be attractive to medicinal chemists preparing collections of sulfonimidamides or sulfinamides.

## Supporting Information Available

Experimental procedures and supporting characterization data and spectra (PDF)
