# Supplementary material for: A Silyl Sulfinylamine Reagent Enables the Modular Synthesis of Sulfonimidamides via Primary Sulfinamides
Source: Org Lett. 2022 Feb 21;24(8):1711–5. doi: 10.1021/acs.orglett.2c00347 (PMC9084605; doi:10.1021/acs.orglett.2c00347)
Supplement: Supplementary file 1 — ol2c00347_si_001.pdf [file ol2c00347_si_001.pdf]

A Silyl Sulfinylamine Reagent Enables the Modular Synthesis of Sulfonimidamides via Primary Sulfinamides

Mingyan Ding, Ze-Xin Zhang, Thomas Q. Davies, and Michael C. Willis*

Department of Chemistry, University of Oxford, Chemistry Research Laboratory, Mansfield Road, Oxford, OX1 3TA, United Kingdom

michael.willis@chem.ox.ac.uk

Supporting Information

1. General experimental considerations S2

2. Reaction optimisation S4

3. Experimental procedures and characterisation data S6

3.1 Synthesis of triisopropylsilyl amine (TIPS-NH_2) S6

3.2 Synthesis of *N*-sulfinyltriisopropylsilylamine (TIPS-NSO, **1**) S6

3.3 General procedure A - Synthesis of primary sulfinamide **2** S7

3.4 General procedure B - Synthesis of sulfonimidamides **3** and **4** S15

3.4.1 General procedure B1 - Reaction between primary sulfinamide and secondary amine S15

3.4.2 General procedure B2 - Reaction between primary sulfinamide and primary amine S26

3.5 Synthesis of primary sulfinamide **6** S31

3.6 Synthesis of Sildenafil analogue **7** S32

4. References S33

5. NMR Spectra S34

1. General experimental considerations

Handling techniques: Unless otherwise stated, all reactions were conducted under an atmosphere of nitrogen with anhydrous solvents using standard Schlenk techniques. Glassware was dried in an oven (> 100 °C) and allowed to cool to room temperature under a positive pressure of nitrogen before use. Cooling of reaction mixtures to 0 °C was achieved using an ice-water bath. Cooling of reaction mixtures to -78 °C or -15 °C was achieved using an acetone-dry ice bath. 'Room temperature' refers to an ambient temperature of 21 ± 2 °C.

Reagents: Unless otherwise stated, all chemicals were purchased from commercial sources (Sigma-Aldrich, Fluorochem, Fisher Scientific, Alfa-Aesar or Apollo Scientific) and were used without further purification. Anhydrous solvents were purified by filtration through dried alumina columns using the University of Oxford internal solvent drying system (Innovative Technology Inc. PS-400-7) and sparged with nitrogen before use. All inert gases were sourced from the University of Oxford internal supplies and dried through $CaCl_2$ drying columns. 'Petrol' refers to the fraction of petroleum ether which boils in the range 40-60 °C. 'Brine' refers to a saturated aqueous solution of sodium chloride.

Chromatography: Thin-layer chromatography (TLC) was performed on Merck silica gel 60 F_{254} pre-coated aluminium backed TLC sheets with a visualisation under a UV lamp (λ_{max} = 254 nm) and/or by staining with $KMnO_4$ solution. Flash column chromatography (FCC) was performed using Merck silica gel 60 (230-400 mesh) with the solvent system indicated in parenthesis.

NMR Spectroscopy: ^{1}H NMR spectra were recorded on a Bruker AVIII400 spectrometer at 400 MHz. ^{13}C NMR spectra were recorded on a Bruker AVIII400 spectrometer at 101 MHz. ^{19}F NMR spectra were recorded on a Bruker AVIII400 spectrometer at 377 MHz. All reported ^{1}H and ^{13}C chemical shifts (δ_H, δ_C) are referenced to the residual signal of deuterated solvents ($CDCl_3$: δ_H = 7.26 ppm, δ_C = 77.16 ppm; $(CD_3)_2SO$: δ_H = 2.50 ppm, δ_C = 39.52 ppm; C_6D_6: δ_H = 7.16 ppm, δ_C = 128.06 ppm; CD_3OD: δ_H = 3.31 ppm, δ_C = 49.00 ppm). ^{19}F chemical shifts (δ_F) are referenced externally to $CFCl_3$ (δ_F = 0.0 ppm). Chemical shifts (δ) are reported in parts per million (ppm) to the nearest 0.01 ppm for ^{1}H NMR, and 0.1 ppm for ^{13}C and ^{19}F NMR. Coupling constants (*J*) are reported in Hertz (Hz) and rounded to the nearest 0.5 Hz. Multiplicities are reported as followings: s (singlet), d (doublet), t (triplet), q (quartet), pent. (pentet), sext. (sextet), m (multiplet), br. (broad signal), app. (apparent).

IR Spectroscopy: Infrared spectra were recorded on a Bruker Tensor 27 Fourier Transform spectrometer with an internal range 600-4000 cm^{-1} and all absorption maximum (ν_{max}) are given in wavenumbers (cm^{-1}).

Melting point: Melting points were recorded in degrees Celsius (°C) using a STUART scientific hot-stage

microscope apparatus SMP1 or a Reichert melting point apparatus and are reported uncorrected.

Mass Spectroscopy: High resolution mass spectra (HRMS) were recorded on a Bruker Daltonic µTOF spectrometer through electrospray ionisation (ESI) by the mass spectrometry service at Chemistry Research Laboratory, University of Oxford. m/z values are reported in Daltons (Da) and high-resolution values are calculated to four decimal places from the molecular formula. Samples for mass spectra were prepared in 1 mg/mL solution in MeCN or MeOH (HRMS-ESI).

2. Reaction optimisation

Table 1. Screen of temperatures.

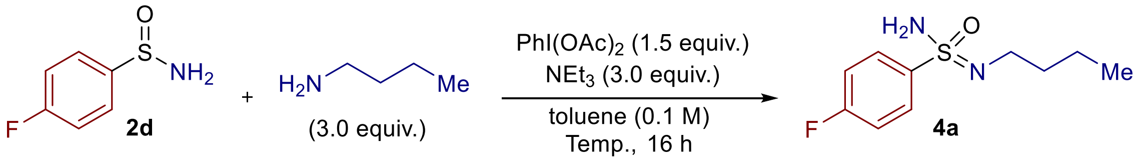

Entry	Temp./°C	NMR yield[b]/%
1	rt	5
2	40	10
3	60	22

(a) Reactions were performed on 0.2 mmol scale. (b) Determined by quantitative ^{19}F NMR spectra of crude reaction mixture using perfluoronaphthalene as an internal standard.

Table 2. Screen of iodine reagents, bases and solvents.

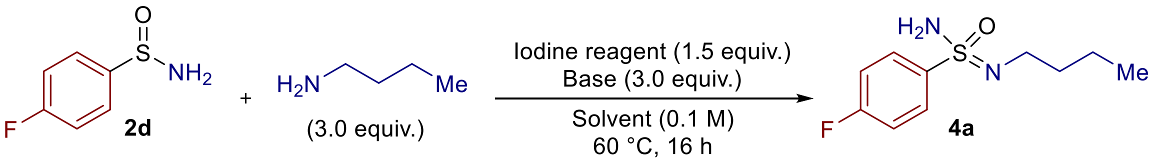

Entry	Iodine reagent	Base	Solvent	NMR yield[b]/%
1	$PhI(OAc)_2$	NEt_3	DCE	40
2	$PhI(OAc)_2$	NEt_3	MeCN	48
3	PhI=O	NEt_3	MeCN	38
4	$PhI(OH)(OSO_2CH_3)$	NEt_3	MeCN	30
5	PhI(OC(O)*t*-Bu)$_2$	NEt_3	MeCN	56
6	PhI(OC(O)*t*-Bu)$_2$	DBU	MeCN	14
7	PhI(OC(O)*t*-Bu)$_2$	DIPEA	MeCN	5
8	PhI(OC(O)*t*-Bu)$_2$	NEt_3	THF	22
9	PhI(OC(O)*t*-Bu)$_2$	NEt_3	1,4-dioxane	23
10	PhI(OC(O)*t*-Bu)$_2$	NEt_3	DMF	49

(a) Reactions were performed on 0.2 mmol scale. (b) Determined by quantitative ^{19}F NMR spectra of crude reaction mixture using perfluoronaphthalene as an internal standard.

Table 3. Stoichiometry screen of *n*-$BuNH_2$, PhI(OC(O)*t*-Bu)$_2$ and NEt_3.

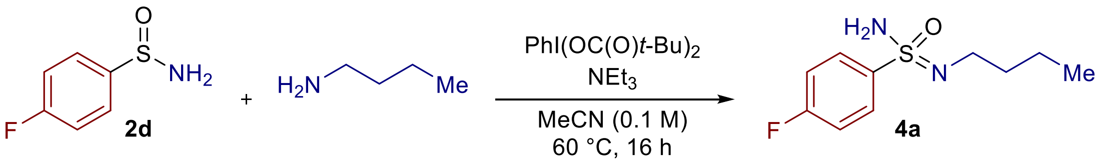

Entry	*n*-$BuNH_2$/equiv.	PhI(OC(O)*t*-Bu)$_2$/equiv.	NEt_3/equiv.	NMR yield[b]/%
1	3	1.5	3	56
2	3	1.5	5	64
3	3	1.5	10	58
4	3	1.8	5	66
5	3	1.8	10	71
6	3	2	10	78
7	3	2	12	78
8	3	2.5	12	82
9	2	2.5	12	84
10	1.5	2.5	12	77

(a) Reactions were performed on 0.2 mmol scale. (b) Determined by quantitative ^{19}F NMR spectra of crude reaction mixture using perfluoronaphthalene as an internal standard.

3. Experimental procedures and characterisation data

3.1 Synthesis of triisopropylsilyl amine (TIPS-NH_2)

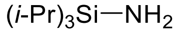

To an oven-dried 1 litre round-bottom flask was dissolved triisopropylsilyl chloride (32.85 g, 170.38 mmol, 1.0 equiv.) in anhydrous diethyl ether (340 mL). The reaction mixture was cooled to -78 °C. Anhydrous ammonia was bubbled through this solution for 2 hours, resulting in the immediate formation of a white precipitate. The reaction was then warmed to 0 °C and stirred for another 3 hours open to air to remove excess ammonia. When bubbling stopped, the reaction slurry was filtered through a pad of anhydrous Na_2SO_4 (3-4 cm), washed with Et_2O (20 mL) and concentrated *in vacuo* to afford triisopropylsilyl amine as a colourless oil (29.14 g, 168.23 mmol, 99%).

IR (ν_{max}, cm^{-1}) 2940, 2864, 1463, 882, 817, 658; $\boldsymbol{\delta_H}$ (400 MHz, C_6D_6) 1.03 (d, *J* = 7.0 Hz, 18H), 0.95-0.84 (m, 3H), -0.13 (br. s, 2H); $\boldsymbol{\delta_C}$ (101 MHz, C_6D_6) 18.4, 12.5; Identification of TIPS-NH_2 using HRMS-ESI and HRMS-EI analyses was not achieved.

Notes:

1) The product may be slightly volatile. For this reason, when removing solvent on a rotary evaporator, the bath temperature should be set to 30 °C or lower.
2) TIPS-NH_2 is stored in the freezer (-20 °C) and can be taken out and used regularly without loss of performance for at least one month.

3.2 Synthesis of *N*-sulfinyltriisopropylsilylamine (TIPS-NSO, 1)

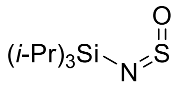

To an oven-dried 1 litre round-bottom flask was added triisopropylsilyl amine (5.23 g, 30.20 mmol, 1.00 equiv.). The vessel was sealed and subjected to three N_2 evacuation/refill cycles before anhydrous diethyl ether (600 mL) was added. The solution was cooled to 0 °C and anhydrous triethylamine (8.67 mL, 62.21 mmol, 2.06 equiv.) was added. Freshly distilled thionyl chloride (2.27 mL, 31.11 mmol, 1.03 equiv.) was then added dropwise. The reaction slurry was then stirred vigorously at 0 °C for 2 hours. Filtration through a pad of anhydrous Na_2SO_4 (3-4 cm) and removal of solvent *in vacuo* afforded TIPS-NSO **1** as a light-yellow oil (6.59 g, 30.03 mmol, 99%).

IR (ν_{max}, cm^{-1}) 2980, 1308, 1128, 882, 682, 664; $\boldsymbol{\delta_H}$ (400 MHz, C_6D_6) 1.12-1.10 (m, 3H), 1.01 (d, *J* = 5.5 Hz, 18H); $\boldsymbol{\delta_C}$ (101 MHz, C_6D_6) 18.0, 12.4; Identification of TIPS-NSO using HRMS-ESI and HRMS-EI analyses was not achieved.

Notes:

1) The product may be slightly volatile. For this reason, when removing solvent on a rotary evaporator, the bath temperature should be set to 30 °C or lower.
2) TIPS-NSO is stored in the freezer (-20 °C) and can be taken out and used regularly without loss of performance for at least one month.
3) **CAUTION: Hydrolysis of sulfinylamines results in the formation of toxic sulfur dioxide gas.** Evolution of SO_2 from TIPS-NSO has not been observed in the normal course of use. But avoidance of contact with water or prolonged storage at room temperature is advised.
4) This reaction has been performed on a range of scales from 1 mmol to 30 mmol with no loss in yield observed.

3.3 General procedure A - Synthesis of primary sulfinamide 2

An oven-dried round-bottom flask containing TIPS-NSO **1** (1.0 equiv.) was sealed and subjected to three N_2 evacuation/refill cycles before pre-sparged anhydrous THF (TIPS-NSO conc. 0.1 M) was added. The solution was cooled to 0 °C and then the corresponding Grignard, organolithium, or organozinc reagent (1.2 equiv.) was added dropwise. The reaction was stirred at 0 °C for 5 minutes prior to the addition of tetrabutylammonium fluoride (TBAF) solution (1.0 M in THF, 2.0 equiv.) at the same temperature. The reaction was warmed to room temperature and stirred for another 10 minutes before being diluted with EtOAc (10 mL) and quenched with saturated aqueous solution of EDTA tetrasodium salt (10 mL). The aqueous phase was extracted with EtOAc (3 × 10 mL). The combined organic layers were then washed with water (10 mL) and brine (10 mL), dried over anhydrous Na_2SO_4, filtered and concentrated *in vacuo*. Primary sulfinamide **2** was then purified by flash column chromatography with the appropriate solvent system.

4-Methylbenzenesulfinamide (2a)

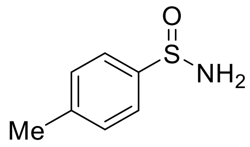

1) Preparation of **2a** using Grignard reagent

Following general procedure A, TIPS-NSO (219.4 mg, 1.00 mmol, 1.0 equiv.), THF (10 mL), 4-methylphenylmagnesium bromide solution (1.22 mL, 1.20 mmol, 0.98 M in THF, 1.2 equiv.) and TBAF solution (2.00 mL, 2.00 mmol, 1.0 M in THF, 2.0 equiv.) were used. Flash column chromatography (SiO_2, CH_2Cl_2/EtOAc = 1:2 to 1:4) afforded the desired sulfinamide **2a** as a white solid (138.5 mg, 0.89 mmol, 89%).

2) Preparation of **2a** using organozinc reagent

4-Methylphenylzinc chloride solution was prepared according to the following procedure. To 4-methylphenylmagnesium bromide solution (1.22 mL, 1.20 mmol, 0.98 M in THF, 1.2 equiv.) in an oven-dried

25 mL round-bottom flask was added a solution of $ZnCl_2$ (163.6 mg, 1.20 mmol, 1.2 equiv.) in THF (2.4 mL) dropwise at 0 °C. The reaction was stirred at the same temperature for 30 minutes.

2a was then prepared following general procedure A using TIPS-NSO (219.4 mg, 1.00 mmol, 1.0 equiv.), THF (10 mL), 4-methylphenylzinc chloride solution (1.20 mmol, 1.2 equiv.) and TBAF solution (2.00 mL, 2.00 mmol, 1.0 M in THF, 2.0 equiv.). Flash column chromatography (SiO_2, CH_2Cl_2/EtOAc = 1:2 to 1:4) afforded the desired sulfinamide **2a** as a white solid (135.8mg, 0.88 mmol, 88%).

M.p. 96-98 °C (*n*-hexane); **IR** (ν_{max}, cm^{-1}) 3275, 3072, 1024, 1010, 812; **δ_H** (400 MHz, $CDCl_3$) 7.60 (d, *J* = 8.0 Hz, 2H), 7.28 (d, *J* = 8.0 Hz, 2H), 4.48 (s, 2H), 2.40 (s, 3H); **δ_C** (101 MHz, $CDCl_3$) 143.6, 141.5, 129.7, 125.5, 21.4; **HRMS** (ESI^+, m/z) calculated for $[C_7H_{10}NOS]^+$ $[M+H]^+$ 156.0478, found 156.0478. Data for this compound was consistent with previous reports.[1]

3-Methylbenzenesulfinamide (2b)

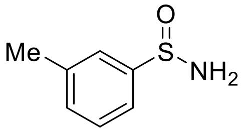

Following general procedure A, TIPS-NSO (219.4 mg, 1.00 mmol, 1.0 equiv.), THF (10 mL), 3-methylphenylmagnesium bromide solution (1.58 mL, 1.20 mmol, 0.76 M in THF, 1.2 equiv.) and TBAF solution (2.00 mL, 2.00 mmol, 1.0 M in THF, 2.0 equiv.) were used. Flash column chromatography (SiO_2, CH_2Cl_2/EtOAc = 1:2 to 1:4) afforded the desired sulfinamide **2b** as a white solid (136.5 mg, 0.88 mmol, 88%).

M.p. 68-70 °C (*n*-hexane); **IR** (ν_{max}, cm^{-1}) 3230, 3098, 1048, 1013, 780, 690; **δ_H** (400 MHz, $CDCl_3$) 7.56 (s, 1H), 7.53 (d, *J* = 7.5 Hz, 1H), 7.38 (app. t, *J* = 7.5 Hz, 1H), 7.29 (d, *J* = 7.5 Hz, 1H), 4.38 (s, 2H), 2.42 (s, 3H); **δ_C** (101 MHz, $CDCl_3$) 146.4, 139.2, 132.0, 128.9, 125.8, 122.6, 21.5; **HRMS** (ESI^+, m/z) calculated for $[C_7H_{10}NOS]^+$ $[M+H]^+$ 156.0478, found 156.0477.

2-Methylbenzenesulfinamide (2c)

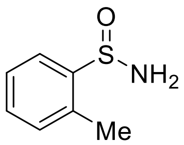

Following general procedure A, TIPS-NSO (219.4 mg, 1.00 mmol, 1.0 equiv.), THF (10 mL), 2-methylphenylmagnesium chloride solution (1.54 mL, 1.20 mmol, 0.78 M in THF, 1.2 equiv.) and TBAF solution (2.00 mL, 2.00 mmol, 1.0 M in THF, 2.0 equiv.) were used. Flash column chromatography (SiO_2, CH_2Cl_2/EtOAc = 1:2 to 1:4) afforded the desired sulfinamide **2c** as a white solid (144.1 mg, 0.93 mmol, 93%).

M.p. 90-92 °C (*n*-hexane); **IR** (ν_{max}, cm^{-1}) 3292, 3185, 1063, 1021, 752; **δ_H** (400 MHz, $CDCl_3$) 7.98 (dd, *J* = 5.5, 3.5 Hz, 1H), 7.38 (dd, *J* = 5.5, 3.5 Hz, 2H), 7.21 (dd, *J* = 5.5, 3.5 Hz, 1H), 4.25 (s, 2H), 2.46 (s, 3H); **δ_C** (101 MHz, $CDCl_3$) 144.5, 136.0, 131.3, 131.1, 126.7, 122.9, 18.7; **HRMS** (ESI^+, m/z) calculated for $[C_7H_{10}NOS]^+$ $[M+H]^+$ 156.0478, found 156.0477. Data for this compound was consistent with previous reports.[2]

4-Fluorobenzenesulfinamide (2d)

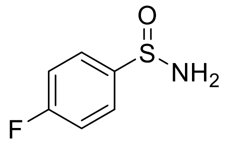

Following general procedure A, TIPS-NSO (219.4 mg, 1.00 mmol, 1.0 equiv.), THF (10 mL), 4-fluorophenylmagnesium bromide solution (1.24 mL, 1.20 mmol, 0.97 M in THF, 1.2 equiv.) and TBAF solution (2.00 mL, 2.00 mmol, 1.0 M in THF, 2.0 equiv.) were used. Flash column chromatography (SiO_2, CH_2Cl_2/EtOAc = 1:2 to 1:3) afforded the desired sulfinamide **2d** as a white crystalline solid (149.5 mg, 0.94 mmol, 94%).

A scale-up of this reaction was performed using TIPS-NSO (1.76 g, 8.00 mmol, 1.0 equiv.), THF (27 mL, TIPS-NSO conc. 0.3 M), 4-fluorophenylmagnesium bromide solution (9.92 mL, 9.60 mmol, 0.97 M in THF, 1.2 equiv.) and TBAF solution (16.00 mL, 16.00 mmol, 1.0 M in THF, 2.0 equiv.). Purification by flash column chromatography (SiO_2, CH_2Cl_2/EtOAc = 1:2 to 1:3) afforded **2d** as a white crystalline solid (1.13 g, 7.08 mmol, 89%).

M.p. 136-138 °C (*n*-hexane); **IR** (ν_{max}, cm^{-1}) 3276, 3070, 1591, 1019, 1009, 836; **δ_H** (400 MHz, $(CD_3)_2SO$) 7.75-7.64 (m, 2H), 7.43-7.32 (m, 2H), 6.30 (s, 2H); **δ_C** (101 MHz, $(CD_3)_2SO$) 163.3 (d, ${}^1J_{C-F}$ = 247.0 Hz), 144.2 (d, ${}^4J_{C-F}$ = 3.0 Hz), 128.0 (d, ${}^3J_{C-F}$ = 9.0 Hz), 115.7 (d, ${}^2J_{C-F}$ = 22.5 Hz); **δ_F** (377 MHz, $(CD_3)_2SO$) -111.4 (tt, *J* = 9.0, 5.5 Hz); **HRMS** (ESI^+, m/z) calculated for $[C_6H_7FNOS]^+$ $[M+H]^+$ 160.0227, found 160.0227. Data for this compound was consistent with previous reports.[3]

4-Chlorobenzenesulfinamide (2e)

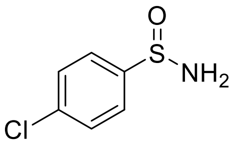

Following general procedure A, TIPS-NSO (219.4 mg, 1.00 mmol, 1.0 equiv.), THF (10 mL), 4-chlorophenylmagnesium bromide solution (1.14 mL, 1.20 mmol, 1.05 M in Et_2O, 1.2 equiv.) and TBAF solution (2.00 mL, 2.00 mmol, 1.0 M in THF, 2.0 equiv.) were used. Flash column chromatography (SiO_2, CH_2Cl_2/EtOAc = 1:2 to 1:4) afforded the desired sulfinamide **2e** as a white solid (164.6 mg, 0.94 mmol, 94%).

M.p. 126-128 °C (*n*-hexane); **IR** (ν_{max}, cm^{-1}) 3277, 3071, 1022, 1006, 828; **δ_H** (400 MHz, $(CD_3)_2SO$) 7.66 (d, *J* =

8.5 Hz, 2H), 7.60 (d, *J* = 8.5 Hz, 2H), 6.35 (s, 2H); **δC** (101 MHz, $(CD_3)_2SO$) 147.2, 135.1, 128.7, 127.4; **HRMS** (ESI^+, m/z) calculated for $[C_6H_7{}^{35}ClNOS]^+$ $[M+H]^+$ 175.9931, found 175.9933. Data for this compound was consistent with previous reports.[4]

3-Cyano-4-ethoxybenzenesulfinamide (2f)

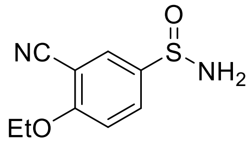

(3-Cyano-4-ethoxyphenyl)lithium solution was prepared according to the following procedure. To a solution of 5-bromo-2-ethoxybenzonitrile (271.3 mg, 1.20 mmol, 1.2 equiv.) in THF (2.4 mL) in an oven-dried 25 mL round-bottom flask was added *n*-butyllithium solution (0.53 mL, 1.20 mmol, 2.25 M in hexanes, 1.2 equiv.) dropwise at -78 °C. The reaction was stirred at the same temperature for 1 hour.

2f was then prepared following general procedure A using TIPS-NSO (219.4 mg, 1.00 mmol, 1.0 equiv.), THF (10 mL), (3-cyano-4-ethoxyphenyl)lithium solution (1.20 mmol, 1.2 equiv.) and TBAF solution (2.00 mL, 2.00 mmol, 1.0 M in THF, 2.0 equiv.). Flash column chromatography (SiO_2, CH_2Cl_2/EtOAc = 1:2 to 1:4) afforded the desired sulfinamide **2f** as a white solid (151.3 mg, 0.72 mmol, 72%).

M.p. 134-136 °C (*n*-hexane); **IR** (ν_{max}, cm^{-1}) 3276, 3089, 2226, 1291, 1054; **δH** (400 MHz, $(CD_3)_2SO$) 7.89 (d, *J* = 2.0 Hz, 1H), 7.86 (dd, *J* = 9.0, 2.0 Hz, 1H), 7.39 (d, *J* = 9.0 Hz, 1H), 6.37 (s, 2H), 4.26 (q, *J* = 7.0 Hz, 2H), 1.39 (t, *J* = 7.0 Hz, 3H); **δC** (101 MHz, $(CD_3)_2SO$) 161.5, 140.4, 132.4, 130.9, 115.8, 113.2, 100.7, 65.2, 14.3; **HRMS** (ESI^+, m/z) calculated for $[C_9H_{11}N_2O_2S]^+$ $[M+H]^+$ 211.0536, found 211.0538.

6-Methoxypyridine-3-sulfinamide (2g)

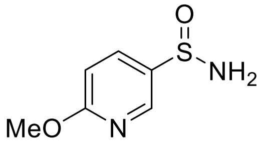

(6-Methoxypyridin-3-yl)lithium solution was prepared according to the following procedure. To a solution of 5-bromo-2-methoxypyridine (225.6 mg, 1.20 mmol, 1.2 equiv.) in THF (2.4 mL) in an oven-dried 25 mL round-bottom flask was added *n*-butyllithium solution (0.53 mL, 1.20 mmol, 2.25 M in hexanes, 1.2 equiv.) dropwise at -78 °C. The reaction was stirred at the same temperature for 10 minutes.

2g was then prepared following general procedure A using TIPS-NSO (219.4 mg, 1.00 mmol, 1.0 equiv.), THF (10 mL), (6-methoxypyridin-3-yl)lithium solution (1.20 mmol, 1.2 equiv.) and TBAF solution (2.00 mL, 2.00 mmol, 1.0 M in THF, 2.0 equiv.). Flash column chromatography (SiO_2, CH_2Cl_2/EtOAc = 1:2 to 1:4) afforded the desired sulfinamide **2g** as a white solid (92.2 mg, 0.54 mmol, 54%).

M.p. 110-112 °C (*n*-hexane); **IR** (ν_{max}, cm^{-1}) 3278, 3096, 1589, 1480, 1372, 1281, 1028; **δ_H** (400 MHz, $(CD_3)_2SO$) 8.36 (dd, *J* = 2.5, 0.5 Hz, 1H), 7.89 (dd, *J* = 8.5, 2.5 Hz, 1H), 6.95 (dd, *J* = 8.5, 0.5 Hz, 1H), 6.37 (s, 2H), 3.90 (s, 3H); **δ_C** (101 MHz, $(CD_3)_2SO$) 164.8, 144.9, 136.9, 136.8, 110.7, 53.7; **HRMS** (ESI$^+$, m/z) calculated for $[C_6H_9N_2O_2S]^+$ $[M+H]^+$ 173.0379, found 173.0380.

5-Bromopyridine-3-sulfinamide (2h)

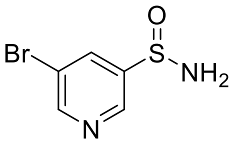

(5-Bromoypyridin-3-yl)magnesium chloride lithium chloride complex solution was prepared according to the following procedure. To a solution of 3,5-dibromopyridine (284.3 mg, 1.20 mmol, 1.2 equiv.) in THF (1.2 mL) in an oven-dried 25 mL round-bottom flask was added isopropylmagnesium chloride lithium chloride complex solution (Turbo Grignard Reagent, 0.96 mL, 1.20 mmol, 1.25 M in THF, 1.2 equiv.) dropwise at -15 °C. The reaction was stirred at the same temperature for 40 minutes.

2h was then prepared following general procedure A using TIPS-NSO (219.4 mg, 1.00 mmol, 1.0 equiv.), THF (10 mL), (5-bromoypyridin-3-yl)magnesium chloride lithium chloride complex solution (1.20 mmol, 1.2 equiv.) and TBAF solution (2.00 mL, 2.00 mmol, 1.0 M in THF, 2.0 equiv.). Flash column chromatography (SiO_2, CH_2Cl_2/EtOAc = 1:2 to 1:4) afforded the desired sulfinamide **2h** as a white solid (192.9 mg, 0.87 mmol, 87%).

M.p. 142-144 °C (*n*-hexane); **IR** (ν_{max}, cm^{-1}) 3322, 3183, 1413, 1027, 1001; **δ_H** (400 MHz, $(CD_3)_2SO$) 8.84 (d, *J* = 2.0 Hz, 1H), 8.75 (d, *J* = 2.0 Hz, 1H), 8.19 (app. t, *J* = 2.0 Hz, 1H), 6.65 (s, 2H); **δ_C** (101 MHz, $(CD_3)_2SO$) 151.7, 146.0, 145.5, 135.7, 120.2; **HRMS** (ESI$^+$, m/z) calculated for $[C_5H_6{}^{79}BrN_2OS]^+$ $[M+H]^+$ 220.9379, found 220.9380.

Thiophene-2-sulfinamide (2i)

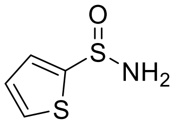

Following general procedure A, TIPS-NSO (219.4 mg, 1.00 mmol, 1.0 equiv.), THF (10 mL), 2-thienylmagnesium bromide solution (1.04 mL, 1.20 mmol, 1.15 M in THF, 1.2 equiv.) and TBAF solution (2.00 mL, 2.00 mmol, 1.0 M in THF, 2.0 equiv.) were used. Flash column chromatography (SiO_2, CH_2Cl_2/EtOAc = 1:2 to 1:4) afforded the desired sulfinamide **2i** as a white solid (120.8 mg, 0.82 mmol, 82%).

M.p. 94-96 °C (*n*-hexane); **IR** (ν_{max}, cm^{-1}) 3158, 1039, 1002, 908, 847, 711; **δ_H** (400 MHz, $(CD_3)_2SO$) 7.83 (dd, *J* = 5.0, 1.5 Hz, 1H), 7.31 (dd, *J* = 3.5, 1.5 Hz, 1H), 7.16 (dd, *J* = 5.0, 3.5 Hz, 1H), 6.59 (s, 2H); **δ_C** (101 MHz, $(CD_3)_2SO$) 151.2, 131.1, 128.7, 127.9; **HRMS** (ESI$^+$, m/z) calculated for $[C_4H_6NOS_2]^+$ $[M+H]^+$ 147.9885, found 147.9886.

Benzofuran-2-sulfinamide (2j)

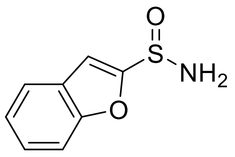

2-Lithiobenzofuran solution was prepared according to the following procedure. To a solution of benzofuran (141.8 mg, 1.20 mmol, 1.2 equiv.) in THF (2.4 mL) in an oven-dried 25 mL round-bottom flask was added *n*-butyllithium solution (0.53 mL, 1.20 mmol, 2.25 M in hexanes, 1.2 equiv.) dropwise at -78 °C. The reaction was stirred at the same temperature for 10 minutes.

2j was then prepared following general procedure A using TIPS-NSO (219.4 mg, 1.00 mmol, 1.0 equiv.), THF (10 mL), 2-lithiobenzofuran solution (1.20 mmol, 1.2 equiv.) and TBAF solution (2.00 mL, 2.00 mmol, 1.0 M in THF, 2.0 equiv.). Flash column chromatography (SiO_2, CH_2Cl_2/EtOAc = 1:1 to 1:2) afforded the desired sulfinamide **2j** as a white solid (151.5 mg, 0.84 mmol, 84%).

M.p. 120-122 °C (*n*-hexane); **IR** (ν_{max}, cm^{-1}) 3317, 3205, 1445, 1043, 734; **δ_H** (400 MHz, $(CD_3)_2SO$) 7.75 (dd, *J* = 7.5, 1.5 Hz, 1H), 7.67 (d, *J* = 8.5 Hz, 1H), 7.42 (ddd, *J* = 8.5, 7.5, 1.5 Hz, 1H), 7.33 (app. td, *J* = 7.5, 1.0 Hz, 1H), 7.27 (s, 1H), 6.83 (s, 2H); **δ_C** (101 MHz, $(CD_3)_2SO$) 159.4, 155.7, 126.9, 126.0, 123.7, 122.3, 111.8, 108.2; **HRMS** (ESI^+, m/z) calculated for $[C_8H_7NO_2SNa]^+$ $[M+Na]^+$ 204.0090, found 204.0092.

Butane-1-sulfinamide (2k)

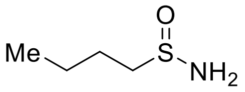

Following general procedure A, TIPS-NSO (219.4 mg, 1.00 mmol, 1.0 equiv.), THF (10 mL), *n*-butylmagnesium chloride solution (0.61 mL, 1.20 mmol, 1.96 M in THF, 1.2 equiv.) and TBAF solution (2.00 mL, 2.00 mmol, 1.0 M in THF, 2.0 equiv.) were used. Flash column chromatography (SiO_2, CH_2Cl_2/EtOAc/MeOH = 1:2:0 to 0:1:0 to 0:12:1) afforded the desired sulfinamide **2k** as a colourless oil (108.1 mg, 0.89 mmol, 89%).

IR (ν_{max}, cm^{-1}) 3220, 3111, 1465, 1030; **δ_H** (400 MHz, $CDCl_3$) 4.54 (s, 2H), 2.81-2.64 (m, 2H), 1.73-1.54 (m, 2H), 1.52-1.30 (m, 2H), 0.90 (t, *J* = 7.5 Hz, 3H); **δ_C** (101 MHz, $CDCl_3$) 57.1, 25.1, 21.8, 13.8; **HRMS** (ESI^+, m/z) calculated for $[C_4H_{12}NOS]^+$ $[M+H]^+$ 122.0634, found 122.0635. Data for this compound was consistent with previous reports.[5]

Phenylmethanesulfinamide (2l)

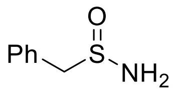

Following general procedure A, TIPS-NSO (219.4 mg, 1.00 mmol, 1.0 equiv.), THF (10 mL), benzylmagnesium chloride solution (1.35 mL, 1.20 mmol, 0.89 M in THF, 1.2 equiv.) and TBAF solution (2.00 mL, 2.00 mmol, 1.0 M in THF, 2.0 equiv.) were used. Flash column chromatography (SiO_2, CH_2Cl_2/EtOAc/MeOH = 1:2:0 to 0:1:0 to 0:10:1) afforded the desired sulfinamide **2l** as a white solid (108.7 mg, 0.70 mmol, 70%).

M.p. 106-108 °C (*n*-hexane); **IR** (ν_{max}, cm^{-1}) 3252, 3088, 1049, 996, 670; δ_H (400 MHz, CD_3OD) 7.38–7.28 (m, 5H), 4.85 (s, 2H), 4.07 (d, *J* = 13.0 Hz, 1H), 3.99 (d, *J* = 13.0 Hz, 1H); δ_C (101 MHz, CD_3OD) 132.0, 131.6, 129.6, 129.1, 63.3; **HRMS** (ESI^+, m/z) calculated for $[C_7H_{10}NOS]^+$ $[M+H]^+$ 156.0478, found 156.0476. Data for this compound was consistent with previous reports.[4]

Cyclohexanesulfinamide (2m)

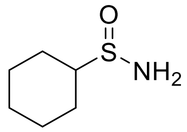

Following general procedure A, TIPS-NSO (219.4 mg, 1.00 mmol, 1.0 equiv.), THF (10 mL), cyclohexylmagnesium chloride solution (1.26 mL, 1.20 mmol, 0.95 M in 2-methyltetrahydrofuran, 1.2 equiv.) and TBAF solution (2.00 mL, 2.00 mmol, 1.0 M in THF, 2.0 equiv.) were used. Flash column chromatography (SiO_2, CH_2Cl_2/EtOAc/ MeOH = 1:2:0 to 0:1:0 to 0:12:1) afforded the desired sulfinamide **2m** as a white solid (134.8 mg, 0.92 mmol, 92%).

M.p. 82-84 °C (*n*-hexane); **IR** (ν_{max}, cm^{-1}) 3271, 3188, 1451, 1043, 1017; δ_H (400 MHz, $CDCl_3$) 4.17 (s, 2H), 2.47 (tt, *J* = 11.0, 3.5 Hz, 1H), 2.09-1.95 (m, 2H), 1.92-1.77 (m, 2H), 1.74-1.60 (m, 1H), 1.49-1.14 (m, 5H); δ_C (101 MHz, $CDCl_3$) 63.5, 25.9, 25.7, 25.4, 25.28, 25.25 (note: for cyclohexane ring, 6 peaks were found instead of 4 due to the loss of symmetry caused by chiral sulfur atom); **HRMS** (ESI^+, m/z) calculated for $[C_6H_{14}NOS]^+$ $[M+H]^+$ 148.0791, found 148.0788. Data for this compound was consistent with previous reports.[6]

2-Methylpropane-2-sulfinamide (2n)

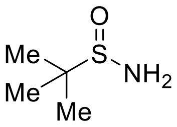

Following general procedure A, TIPS-NSO (219.4 mg, 1.00 mmol, 1.0 equiv.), THF (10 mL), *tert*-butylmagnesium bromide solution (1.26 mL, 1.20 mmol, 0.95 M in THF, 1.2 equiv.) and TBAF solution (2.00 mL, 2.00 mmol, 1.0 M in THF, 2.0 equiv.) were used. Flash column chromatography (SiO_2, CH_2Cl_2/EtOAc/

MeOH = 1:2:0 to 0:1:0 to 0:10:1) afforded the desired sulfinamide **2n** as a white solid (98.6 mg, 0.81 mmol, 81%).

M.p. 80-82 °C (*n*-hexane); **IR** (ν_{max}, cm^{-1}) 3227, 1475, 1364, 1029; δ_H (400 MHz, $CDCl_3$) 3.96 (s, 2H), 1.18 (s, 9H); δ_C (101 MHz, $CDCl_3$) 55.3, 22.2; **HRMS** (ESI$^+$, m/z) calculated for $[C_4H_{12}NOS]^+$ $[M+H]^+$ 122.0634, found 122.0635. Data for this compound was consistent with previous reports.[3]

2-Methylprop-1-ene-1-sulfinamide (2o)

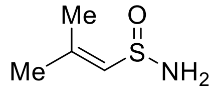

Following general procedure A, TIPS-NSO (438.8 mg, 2.00 mmol, 1.0 equiv.), THF (20 mL), 2-methyl-1-propenylmagnesium bromide solution (12.00 mL, 2.40 mmol, 0.20 M in THF, 1.2 equiv.) and TBAF solution (4.00 mL, 4.00 mmol, 1.0 M in THF, 2.0 equiv.) were used. Flash column chromatography (SiO_2, CH_2Cl_2/EtOAc/MeOH = 1:1:0 to 0:1:0 to 0:10:1) afforded the desired sulfinamide **2o** as a white solid (88.3 mg, 0.74 mmol, 37%).

M.p. 60-62 °C (*n*-hexane); **IR** (ν_{max}, cm^{-1}) 3218, 3085, 1437, 1055; δ_H (400 MHz, $CDCl_3$) 6.07 (app. pent., *J* = 1.5 Hz, 1H), 4.45 (s, 2H), 1.96 (d, *J* = 1.5 Hz, 3H), 1.84 (d, *J* = 1.5 Hz, 3H); δ_C (101 MHz, $CDCl_3$) 145.6, 133.4, 25.3, 20.0; **HRMS** (ESI$^+$, m/z) calculated for $[C_4H_{10}NOS]^+$ $[M+H]^+$ 120.0478, found 120.0479.

4-(5-(*p*-Tolyl)-3-(trifluoromethyl)-1*H*-pyrazol-1-yl)benzenesulfinamide (2p)

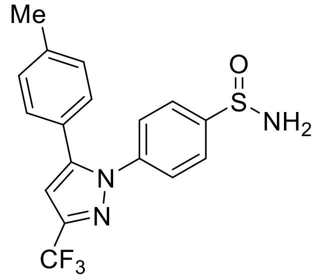

(4-(5-(*p*-tolyl)-3-(trifluoromethyl)-1*H*-pyrazol-1-yl)phenyl)lithium solution was prepared according to the following procedure. To a solution of 1-(4-bromophenyl)-5-(*p*-tolyl)-3-(trifluoromethyl)-1*H*-pyrazole[7] (457.4 mg, 1.20 mmol, 1.2 equiv.) in THF (2.4 mL) in an oven-dried 25 mL round-bottom flask was added *n*-butyllithium solution (0.53 mL, 1.20 mmol, 2.25 M in hexanes, 1.2 equiv.) dropwise at -78 °C. The reaction was stirred at the same temperature for 40 minutes.

2p was then prepared following general procedure A using TIPS-NSO (219.4 mg, 1.00 mmol, 1.0 equiv.), THF (10 mL), (4-(5-(*p*-tolyl)-3-(trifluoromethyl)-1*H*-pyrazol-1-yl)phenyl)lithium solution (1.20 mmol, 1.2 equiv.) and TBAF solution (2.00 mL, 2.00 mmol, 1.0 M in THF, 2.0 equiv.). Flash column chromatography (SiO_2,

CH_2Cl_2/EtOAc = 1:1 to 1:2) afforded the desired sulfinamide **2p** as a colourless oil (272.5 mg, 0.75 mmol, 75%).

IR (ν_{max}, cm^{-1}) 3214, 3102, 1471, 1235, 1161, 1133, 1097, 1051, 976, 755; **δ_H** (400 MHz, $CDCl_3$) 7.67 (d, *J* = 8.5 Hz, 2H), 7.40 (d, *J* = 8.5 Hz, 2H), 7.12 (d, *J* = 8.0 Hz, 2H), 7.08 (d, *J* = 8.0 Hz, 2H), 6.71 (s, 1H), 4.92 (s, 2H), 2.32 (s, 3H); **δ_C** (101 MHz, $CDCl_3$) 146.3, 145.1, 143.7 (q, $^2J_{C-F}$ = 38.5 Hz), 141.4, 139.6, 129.6, 128.7, 126.7, 125.9, 125.6, 121.2 (q, $^1J_{C-F}$ = 269.0 Hz), 106.0, 21.3; **δ_F** (377 MHz, $CDCl_3$) -62.3 (s); **HRMS** (ESI$^+$, m/z) calculated for $[C_{17}H_{15}F_3N_3OS]^+$ $[M+H]^+$ 366.0882, found 366.0885.

3.4 General procedure B - Synthesis of sulfonimidamides 3 and 4

3.4.1 General procedure B1 - Reaction between primary sulfinamide and secondary amine

An oven-dried round-bottom flask containing primary sulfinamide **2** (1.0 equiv.) and $PhI(OAc)_2$ (1.5 equiv.) was sealed and subjected to three N_2 evacuation/refill cycles before pre-sparged anhydrous toluene (sulfinamide conc. 0.1 M) was added. Anhydrous triethylamine (3.0 equiv.) was added to the solution, followed immediately by the addition of secondary amine (1.5 equiv.). The reaction mixture was then stirred at room temperature for the specified time. Once judged complete by TLC, the reaction was diluted with EtOAc (10 mL) and quenched with saturated aqueous solution of $NaHCO_3$ (10 mL). The aqueous phase was extracted with EtOAc (3 × 10 mL). The combined organic layers were then dried over anhydrous Na_2SO_4, filtered and concentrated *in vacuo*. Sulfonimidamide **3** was purified by flash column chromatography with the appropriate solvent system.

4-(4-Methylphenylsulfonimidoyl)morpholine (3a)

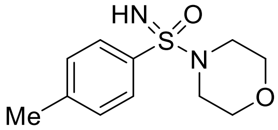

Following general procedure B1, 4-methylbenzenesulfinamide **2a** (96.8 mg, 0.62 mmol, 1.0 equiv.), $PhI(OAc)_2$ (299.6 mg, 0.93 mmol, 1.5 equiv.), toluene (6.2 mL), triethylamine (262 µL, 1.88 mmol, 3.0 equiv.) and morpholine (81 µL, 0.93 mmol, 1.5 equiv.) were combined at room temperature for 1.5 hours. Flash column chromatography (SiO_2, Petrol/EtOAc = 1:1 to 1:2) afforded the desired sulfonimidamide **3a** as a white crystalline solid (126.5 mg, 0.53 mmol, 85%).

M.p. 90-92 °C (CH_2Cl_2); **IR** (ν_{max}, cm^{-1}) 3265, 1254, 1110, 926, 711; **δ_H** (400 MHz, $CDCl_3$) 7.73 (d, *J* = 8.0 Hz, 2H), 7.30 (d, *J* = 8.0 Hz, 2H), 3.68 (t, *J* = 4.5 Hz, 4H), 2.95 (t, *J* = 4.5 Hz, 4H), 2.41 (s, 3H), 2.38 (s, 1H); **δ_C** (101 MHz, $CDCl_3$) 143.5, 132.2, 129.6, 128.3, 66.6, 47.2, 21.5; **HRMS** (ESI$^+$, m/z) calculated for $[C_{11}H_{17}N_2O_2S]^+$ $[M+H]^+$ 241.1005, found 241.1004. Data for this compound was consistent with previous reports.[8]

4-(3-Methylphenylsulfonimidoyl)morpholine (3b)

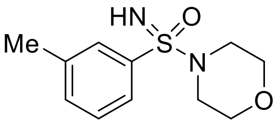

Following general procedure B1, 3-methylbenzenesulfinamide **2b** (67.3 mg, 0.43 mmol, 1.0 equiv.), $PhI(OAc)_2$ (209.4 mg, 0.65 mmol, 1.5 equiv.), toluene (4.3 mL), triethylamine (180 µL, 1.29 mmol, 3.0 equiv.) and morpholine (57 µL, 0.65 mmol, 1.5 equiv.) were combined at room temperature for 8 hours. Flash column chromatography (SiO_2, Petrol/EtOAc = 1:1.2 to 1:1.8) afforded the desired sulfonimidamide **3b** as a colourless oil (77.4 mg, 0.32 mmol, 75%).

IR (ν_{max}, cm^{-1}) 3268, 1256, 1112, 930, 714; $\boldsymbol{\delta_H}$ (400 MHz, $CDCl_3$) 7.71-7.63 (m, 2H), 7.45-7.35 (m, 2H), 3.71 (t, *J* = 4.5 Hz, 4H), 2.98 (t, *J* = 4.5 Hz, 4H), 2.43 (s, 3H), 2.32 (s, 1H); $\boldsymbol{\delta_C}$ (101 MHz, $CDCl_3$) 139.2, 135.0, 133.6, 128.9, 128.5, 125.4, 66.6, 47.2, 21.5; **HRMS** (ESI^+, m/z) calculated for $[C_{11}H_{17}N_2O_2S]^+$ $[M+H]^+$ 241.1005, found 241.1001.

4-(2-Methylphenylsulfonimidoyl)morpholine (3c)

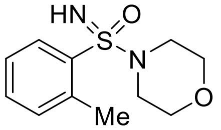

Following general procedure B1, 2-methylbenzenesulfinamide **2c** (134.6 mg, 0.87 mmol, 1.0 equiv.), $PhI(OAc)_2$ (422.0 mg, 1.31 mmol, 1.5 equiv.), toluene (8.7 mL), triethylamine (364 µL, 2.61 mmol, 3.0 equiv.) and morpholine (115 µL, 1.31 mmol, 1.5 equiv.) were combined at room temperature for 1.5 hours. Flash column chromatography (SiO_2, Petrol/EtOAc = 1:3 to 1:3.5) afforded the desired sulfonimidamide **3c** as a colourless oil (157.0 mg, 0.65 mmol, 75%).

IR (ν_{max}, cm^{-1}) 3270, 1256, 1111, 929, 713; $\boldsymbol{\delta_H}$ (400 MHz, $CDCl_3$) 7.95 (dd, *J* = 8.0, 1.5 Hz, 1H), 7.40 (app. td, *J* = 7.5, 1.5 Hz, 1H), 7.31-7.23 (m, 2H), 3.67 (t, *J* = 4.5 Hz, 4H), 3.16-3.05 (m, 4H), 2.83-2.63 (br. s, 1H), 2.68 (s, 3H); $\boldsymbol{\delta_C}$ (101 MHz, $CDCl_3$) 138.2, 136.3, 133.2, 132.5, 130.2, 126.0, 66.6, 46.1, 21.4; **HRMS** (ESI^+, m/z) calculated for $[C_{11}H_{17}N_2O_2S]^+$ $[M+H]^+$ 241.1005, found 241.1004. Data for this compound was consistent with previous reports.[9]

4-(4-Fluorophenylsulfonimidoyl)morpholine (3d)

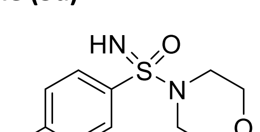

Following general procedure B1, 4-fluorobenzenesulfinamide **2d** (159.2 mg, 1.00 mmol, 1.0 equiv.), $PhI(OAc)_2$ (483.2 mg, 1.50 mmol, 1.5 equiv.), toluene (10.0 mL), triethylamine (418 µL, 3.00 mmol, 3.0 equiv.) and

morpholine (131 µL, 1.50 mmol, 1.5 equiv.) were combined at room temperature for 1.5 hours. Flash column chromatography (SiO_2, Petrol/EtOAc = 1:1 to 1:2) afforded the desired sulfonimidamide **3d** as a white crystalline solid (223.9 mg, 0.92 mmol, 92%).

A scale-up of this reaction was performed using 4-fluorobenzenesulfinamide **2d** (1.59 g, 10.00 mmol, 1.0 equiv.), $PhI(OAc)_2$ (4.83 g, 15.00 mmol, 1.5 equiv.), toluene (33.3 mL, sulfinamide conc. 0.3 M), triethylamine (4.18 mL, 30.00 mmol, 3.0 equiv.) and morpholine (1.31 mL, 15.00 mmol, 1.5 equiv.). Purification by flash column chromatography (SiO_2, Petrol/EtOAc = 1:1 to 1:2) afforded **3d** as a white crystalline solid (2.25 g, 9.22 mmol, 92%).

M.p. 101-103 °C (CH_2Cl_2); **IR** (ν_{max}, cm^{-1}) 3269, 1257, 1112, 931, 842, 715; **δ_H** (400 MHz, $CDCl_3$) 7.93-7.83 (m, 2H), 7.24-7.14 (m, 2H), 3.70 (t, J = 4.5 Hz, 4H), 2.97 (t, J = 4.5 Hz, 4H), 2.56 (s, 1H); **δ_C** (101 MHz, $CDCl_3$) 165.3 (d, $^{1}J_{C-F}$ = 254.5 Hz), 131.2 (d, $^{4}J_{C-F}$ = 3.5 Hz), 130.9 (d, $^{3}J_{C-F}$ = 9.0 Hz), 116.2 (d, $^{2}J_{C-F}$ = 22.5 Hz), 66.5, 47.2; **δ_F** (377 MHz, $CDCl_3$) -105.8 (tt, J = 8.0, 5.0 Hz); **HRMS** (ESI^+, m/z) calculated for $[C_{10}H_{14}FN_2O_2S]^+$ $[M+H]^+$ 245.0755, found 245.0755. Data for this compound was consistent with previous reports.[10]

4-(4-Chlorophenylsulfonimidoyl)morpholine (3e)

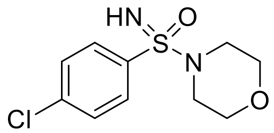

Following general procedure B1, 4-chlorobenzenesulfinamide **2e** (44.8 mg, 0.26 mmol, 1.0 equiv.), $PhI(OAc)_2$ (125.6 mg, 0.39 mmol, 1.5 equiv.), toluene (2.6 mL), triethylamine (109 µL, 0.78 mmol, 3.0 equiv.) and morpholine (34 µL, 0.39 mmol, 1.5 equiv.) were combined at room temperature for 23 hours. Flash column chromatography (SiO_2, Petrol/EtOAc = 1:0.8 to 1:1.2) afforded the desired sulfonimidamide **3e** as a white solid (53.9 mg, 0.21 mmol, 80%).

M.p. 96-98 °C (chloroform); **IR** (ν_{max}, cm^{-1}) 3264, 1257, 1112, 1088, 931, 753; **δ_H** (400 MHz, $CDCl_3$) 7.81 (d, J = 8.5 Hz, 2H), 7.50 (d, J = 8.5 Hz, 2H), 3.71 (t, J = 4.5 Hz, 4H), 2.98 (t, J = 4.5 Hz, 4H), 2.33 (s, 1H); **δ_C** (101 MHz, $CDCl_3$) 139.5, 133.8, 129.7, 129.3, 66.6, 47.2; **HRMS** (ESI^+, m/z) calculated for $[C_{10}H_{14}{}^{35}ClN_2O_2S]^+$ $[M+H]^+$ 261.0459, found 261.0460.

2-Ethoxy-5-(morpholine-4-sulfonimidoyl)benzonitrile (3f)

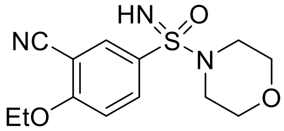

Following general procedure B1, 3-cyano-4-ethoxybenzenesulfinamide **2f** (55.4 mg, 0.26 mmol, 1.0 equiv.),

$PhI(OAc)_2$ (125.6 mg, 0.39 mmol, 1.5 equiv.), toluene (2.6 mL), triethylamine (109 µL, 0.78 mmol, 3.0 equiv.) and morpholine (34 µL, 0.39 mmol, 1.5 equiv.) were combined at room temperature for 1 hour. Flash column chromatography (SiO_2, Petrol/EtOAc = 1:3 to 1:6 to 0:1) afforded the desired sulfonimidamide **3f** as a colourless oil (55.0 mg, 0.19 mmol, 72%).

IR (ν_{max}, cm^{-1}) 3278, 2231, 1288, 1257, 1112, 931; **δ_H** (400 MHz, $(CD_3)_2SO$) 8.03 (d, *J* = 2.5 Hz, 1H), 7.98 (dd, *J* = 9.0, 2.5 Hz, 1H), 7.43 (d, *J* = 9.0 Hz, 1H), 4.69 (s, 1H), 4.30 (q, *J* = 7.0 Hz, 2H), 3.59 (t, *J* = 4.5 Hz, 4H), 2.88-2.76 (m, 4H), 1.40 (t, *J* = 7.0 Hz, 3H)； **δ_C** (101 MHz, $(CD_3)_2SO$) 162.6, 134.7, 133.5, 128.3, 115.3, 113.2, 100.9, 65.6, 65.5, 46.7, 14.2; **HRMS** (ESI^+, m/z) calculated for $[C_{13}H_{18}N_3O_3S]^+$ $[M+H]^+$ 296.1063, found 296.1064.

4-(6-Methoxypyridine-3-sulfonimidoyl)morpholine (3g)

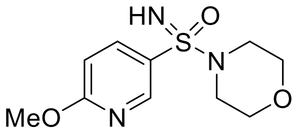

Following general procedure B1, 6-methoxypyridine-3-sulfinamide **2g** (44.6 mg, 0.26 mmol, 1.0 equiv.), $PhI(OAc)_2$ (125.6 mg, 0.39 mmol, 1.5 equiv.), toluene (2.6 mL), triethylamine (109 µL, 0.78 mmol, 3.0 equiv.) and morpholine (34 µL, 0.39 mmol, 1.5 equiv.) were combined at room temperature for 3 hours. Flash column chromatography (SiO_2, Petrol/EtOAc = 1:2.5 to 1:3.5) afforded the desired sulfonimidamide **3g** as a white solid (55.5 mg, 0.22 mmol, 84%).

M.p. 86-88 °C (*n*-hexane); **IR** (ν_{max}, cm^{-1}) 3271, 1589, 1480, 1255, 1112, 929, 716; **δ_H** (400 MHz, $(CD_3)_2SO$) 8.53 (dd, *J* = 2.5, 0.5 Hz, 1H), 7.99 (dd, *J* = 8.5, 2.5 Hz, 1H), 7.00 (dd, *J* = 8.5, 0.5 Hz, 1H), 4.65 (s, 1H), 3.94 (s, 3H), 3.60 (t, *J* = 4.5 Hz, 4H), 2.89-2.76 (m, 4H); **δ_C** (101 MHz, $(CD_3)_2SO$) 165.7, 147.6, 138.9, 125.5, 110.7, 65.6, 54.0, 46.7; **HRMS** (ESI^+, m/z) calculated for $[C_{10}H_{16}N_3O_3S]^+$ $[M+H]^+$ 258.0907, found 258.0908. Data for this compound was consistent with previous reports.[9]

4-(5-Bromopyridine-3-sulfonimidoyl)morpholine (3h)

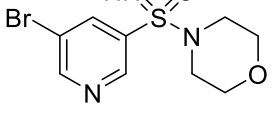

Following general procedure B1, 5-bromopyridine-3-sulfinamide **2h** (76.4 mg, 0.35 mmol, 1.0 equiv.), $PhI(OAc)_2$ (170.7 mg, 0.53 mmol, 1.5 equiv.), toluene (3.5 mL), triethylamine (146 µL, 1.05 mmol, 3.0 equiv.) and morpholine (46 µL, 0.53 mmol, 1.5 equiv.) were combined at room temperature for 22 hours. Flash column chromatography (SiO_2, Petrol/EtOAc = 1:3 to 1:3.5) afforded the desired sulfonimidamide **3h** as a white solid (86.3 mg, 0.28 mmol, 81%).

M.p. 116-118 °C (*n*-hexane); **IR** (ν_{max}, cm^{-1}) 3270, 1273, 1258, 1111, 931, 696; **δ_H** (400 MHz, $(CD_3)_2SO$) 8.99 (d, *J* = 2.0 Hz, 1H), 8.88 (d, *J* = 2.0 Hz, 1H), 8.29 (app. t, *J* = 2.0 Hz, 1H), 5.08 (s, 1H), 3.60 (t, *J* = 4.5 Hz, 4H), 3.00-2.82 (m, 4H); **δ_C** (101 MHz, $(CD_3)_2SO$) 153.7, 146.7, 137.7, 134.3, 120.4, 65.6, 46.6; **HRMS** (ESI^+, m/z) calculated for $[C_9H_{13}{}^{79}BrN_3O_2S]^+$ $[M+H]^+$ 305.9906, found 305.9908. Data for this compound was consistent with previous reports.[9]

4-(Thiophene-2-sulfonimidoyl)morpholine (3i)

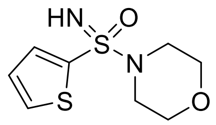

Following general procedure B1, thiophene-2-sulfinamide **2i** (53.6 mg, 0.36 mmol, 1.0 equiv.), $PhI(OAc)_2$ (173.9 mg, 0.54 mmol, 1.5 equiv.), toluene (3.6 mL), triethylamine (151 µL, 1.08 mmol, 3.0 equiv.) and morpholine (47 µL, 0.54 mmol, 1.5 equiv.) were combined at room temperature for 1 hour. Flash column chromatography (SiO_2, Petrol/EtOAc = 1:2 to 1:3) afforded the desired sulfonimidamide **3i** as a white solid (67.5 mg, 0.29 mmol, 81%).

M.p. 122-124 °C (chloroform); **IR** (ν_{max}, cm^{-1}) 3269, 1258, 1112, 931, 713; **δ_H** (400 MHz, $CDCl_3$) 7.60 (dd, *J* = 5.0, 1.5 Hz, 1H), 7.54 (dd, *J* = 3.5, 1.5 Hz, 1H), 7.14 (dd, *J* = 5.0, 3.5 Hz, 1H), 3.75 (t, *J* = 4.5 Hz, 4H), 3.07 (t, *J* = 4.5 Hz, 4H), 2.63 (s, 1H); **δ_C** (101 MHz, $CDCl_3$) 136.6, 132.7, 132.6, 127.9, 66.5, 47.2; **HRMS** (ESI^+, m/z) calculated for $[C_8H_{13}N_2O_2S_2]^+$ $[M+H]^+$ 233.0413, found 233.0414.

4-(Benzofuran-2-sulfonimidoyl)morpholine (3j)

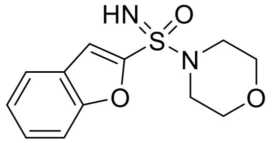

Following general procedure B1, benzofuran-2-sulfinamide **2j** (95.0 mg, 0.52 mmol, 1.0 equiv.), $PhI(OAc)_2$ (251.2 mg, 0.78 mmol, 1.5 equiv.), toluene (5.2 mL), triethylamine (217 µL, 1.56 mmol, 3.0 equiv.) and morpholine (68 µL, 0.78 mmol, 1.5 equiv.) were combined at room temperature for 3 hours. Flash column chromatography (SiO_2, Petrol/EtOAc = 1:1.5 to 1:2) afforded the desired sulfonimidamide **3j** as a white solid (92.4 mg, 0.35 mmol, 67%).

M.p. 88-90 °C (Et_2O); **IR** (ν_{max}, cm^{-1}) 3270, 1282, 1258, 1111, 1078, 939; **δ_H** (400 MHz, $CDCl_3$) 7.67 (app. dt, *J* = 8.0, 1.0 Hz, 1H), 7.56 (dd, *J* = 8.5, 1.0 Hz, 1H), 7.45 (ddd, *J* = 8.5, 7.0, 1.0 Hz, 1H), 7.37 (d, *J* = 1.0 Hz, 1H), 7.33 (ddd, *J* = 8.0, 7.0, 1.0 Hz, 1H), 3.73 (t, *J* = 4.5 Hz, 4H), 3.27 (t, *J* = 4.5 Hz, 4H), 2.74 (s, 1H); **δ_C** (101 MHz, $CDCl_3$) 156.1, 149.2, 127.6, 126.3, 124.3, 122.9, 113.3, 112.4, 66.5, 47.0; **HRMS** (ESI^+, m/z) calculated for $[C_{12}H_{15}N_2O_3S]^+$ $[M+H]^+$ 267.0798, found 267.0797. Data for this compound was consistent with previous

reports.[9]

4-(Butylsulfonimidoyl)morpholine (3k)

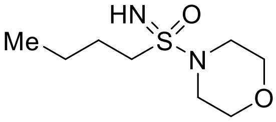

Following general procedure B1, butane-1-sulfinamide **2k** (109.8 mg, 0.91 mmol, 1.0 equiv.), PhI(OAc)$_2$ (441.3 mg, 1.37 mmol, 1.5 equiv.), toluene (9.1 mL), triethylamine (381 µL, 2.73 mmol, 3.0 equiv.) and morpholine (120 µL, 1.37 mmol, 1.5 equiv.) were combined at room temperature for 3 hours. Flash column chromatography (SiO$_2$, Petrol/EtOAc/MeOH = 1:1:0 to 0:1:0 to 0:13:1) afforded the desired sulfonimidamide **3k** as a colourless oil (85.8 mg, 0.42 mmol, 46%).

IR (ν_{max}, cm^{-1}) 3271, 1247, 1110, 931; **δ_H** (400 MHz, CDCl$_3$) 3.72 (t, *J* = 4.5 Hz, 4H), 3.34-3.19 (m, 4H), 2.97 (ddd, *J* = 13.5, 10.0, 6.5 Hz, 1H), 2.81 (ddd, *J* = 13.5, 10.0, 6.5 Hz, 1H), 2.20 (s, 1H), 1.93-1.75 (m, 2H), 1.51-1.37 (m, 2H), 0.94 (t, *J* = 7.5 Hz, 3H); **δ_C** (101 MHz, CDCl$_3$) 67.0, 48.5, 46.9, 25.5, 21.9, 13.7; **HRMS** (ESI$^+$, m/z) calculated for $[C_8H_{19}N_2O_2S]^+$ $[M+H]^+$ 207.1162, found 207.1162.

4-(S-Benzylsulfonimidoyl)morpholine (3l)

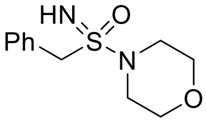

Following general procedure B1, phenylmethanesulfinamide **2l** (77.7 mg, 0.50 mmol, 1.0 equiv.), PhI(OAc)$_2$ (241.6 mg, 0.75 mmol, 1.5 equiv.), toluene (5.0 mL), triethylamine (209 µL, 1.50 mmol, 3.0 equiv.) and morpholine (66 µL, 0.75 mmol, 1.5 equiv.) were combined at room temperature for 2 hours. Flash column chromatography (SiO$_2$, Petrol/EtOAc/MeOH = 1:2:0 to 0:1:0 to 0:15:1) afforded the desired sulfonimidamide **3l** as a white solid (68.3 mg, 0.28 mmol, 57%).

M.p. 134-136 °C (*n*-hexane); **IR** (ν_{max}, cm^{-1}) 3278, 1255, 1111, 935; **δ_H** (400 MHz, CDCl$_3$) 7.45–7.38 (m, 2H), 7.38–7.28 (m, 3H), 4.25 (d, *J* = 13.5 Hz, 1H), 4.21 (d, *J* = 13.5 Hz, 1H), 3.66–3.52 (m, 4H), 3.21–3.06 (m, 4H), 2.25 (s, 1H); **δ_C** (101 MHz, CDCl$_3$) 131.1, 129.1, 128.82, 128.75, 67.0, 56.6, 46.9; **HRMS** (ESI$^+$, m/z) calculated for $[C_{11}H_{17}N_2O_2S]^+$ $[M+H]^+$ 241.1005, found 241.1004.

4-(Cyclohexanesulfonimidoyl)morpholine (3m)

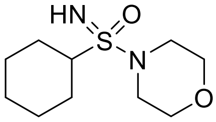

Following general procedure B1, cyclohexanesulfinamide **2m** (61.9 mg, 0.42 mmol, 1.0 equiv.), PhI(OAc)$_2$

(202.9 mg, 0.63 mmol, 1.5 equiv.), toluene (4.2 mL), triethylamine (176 µL, 1.26 mmol, 3.0 equiv.) and morpholine (55 µL, 0.63 mmol, 1.5 equiv.) were combined at room temperature for 3 hours. Flash column chromatography (SiO_2, *n*-hexane/EtOAc = 1:5) afforded the desired sulfonimidamide **3m** as a yellow oil (50.1 mg, 0.22 mmol, 51%).

IR (v_{max}, cm^{-1}) 3270, 1242, 1112, 939; **δ_H** (400 MHz, $CDCl_3$) 3.66 (t, *J* = 4.5 Hz, 4H), 3.33 (t, *J* = 4.5 Hz, 4H), 2.93 (tt, *J* = 12.0, 3.5 Hz, 1H), 2.32-2.11 (m, 2H), 2.11-2.01 (m, 1H), 1.91-1.79 (m, 2H), 1.66 (dtt, *J* = 11.0, 3.5, 1.5 Hz, 1H), 1.60-1.42 (m, 2H), 1.32-1.08 (m, 3H); **δ_C** (101 MHz, $CDCl_3$) 67.5, 61.9, 47.2, 27.4, 26.9, 25.5, 25.4, 25.2 (note: for cyclohexane ring, 6 peaks were found instead of 4 due to the loss of symmetry caused by chiral sulfur atom); **HRMS** (ESI^+, m/z) calculated for $[C_{10}H_{21}N_2O_2S]^+$ $[M+H]^+$ 233.1318, found 233.1319.

4-(2-Methylpropan-2-ylsulfonimidoyl)morpholine (3n)

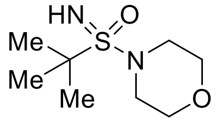

Following general procedure B1, 2-methylpropane-2-sulfinamide **2n** (242.4 mg, 2.00 mmol, 1.0 equiv.), $PhI(OAc)_2$ (966.3 mg, 3.00 mmol, 1.5 equiv.), toluene (20.0 mL), triethylamine (836 µL, 6.00 mmol, 3.0 equiv.) and morpholine (262 µL, 3.00 mmol, 1.5 equiv.) were combined at room temperature for 13 hours. Flash column chromatography (SiO_2, Petrol/EtOAc = 1:4) afforded the desired sulfonimidamide **3n** as a light-yellow crystalline solid (254.1 mg, 1.23 mmol, 62%).

M.p. 53-55 °C (chloroform); **IR** (v_{max}, cm^{-1}) 3271, 1237, 1112, 938; **δ_H** (400 MHz, $CDCl_3$) 3.62 (t, *J* = 4.5 Hz, 4H), 3.36 (t, *J* = 4.5 Hz, 4H), 2.36-1.81 (br. s, 1H), 1.34 (s, 9H); **δ_C** (101 MHz, $CDCl_3$) 67.6, 62.3, 48.3, 25.1; **HRMS** (ESI^+, m/z) calculated for $[C_8H_{19}N_2O_2S]^+$ $[M+H]^+$ 207.1162, found 207.1165.

4-(2-Methylprop-1-en-1-ylsulfonimidoyl)morpholine (3o)

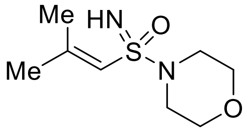

Following general procedure B1, 2-methylprop-1-ene-1-sulfinamide **2o** (57.6 mg, 0.48 mmol, 1.0 equiv.), $PhI(OAc)_2$ (231.9 mg, 0.72 mmol, 1.5 equiv.), toluene (4.8 mL), triethylamine (201 µL, 1.44 mmol, 3.0 equiv.) and morpholine (63 µL, 0.72 mmol, 1.5 equiv.) were combined at room temperature for 2 hours. Flash column chromatography (SiO_2, Petrol/EtOAc/MeOH = 1:1:0 to 0:1:0 to 0:15:1) afforded the desired sulfonimidamide **3o** as a light-yellow oil (61.5 mg, 0.30 mmol, 63%).

IR (v_{max}, cm^{-1}) 3268, 1254, 1111, 929; **δ_H** (400 MHz, $CDCl_3$) 5.93-5.87 (m, 1H), 3.68 (t, *J* = 4.5 Hz, 4H), 3.11 (t, *J*

= 4.5 Hz, 4H), 2.34 (s, 1H), 2.12 (d, *J* = 1.0 Hz, 3 H), 1.89 (d, *J* = 1.0 Hz, 3 H); **δC** (101 MHz, $CDCl_3$) 153.8, 119.0, 66.6, 46.9, 27.3, 19.3; **HRMS** (ESI^+, m/z) calculated for $[C_8H_{17}N_2O_2S]^+$ $[M+H]^+$ 205.1005, found 205.1005.

4-(4-(5-(*p*-Tolyl)-3-(trifluoromethyl)-1*H*-pyrazol-1-yl)phenylsulfonimidoyl)morpholine (3p)

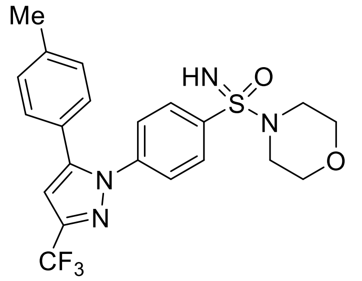

Following general procedure B1, 4-(5-(*p*-tolyl)-3-(trifluoromethyl)-1*H*-pyrazol-1-yl)benzenesulfinamide **2p** (255.6 mg, 0.70 mmol, 1.0 equiv.), $PhI(OAc)_2$ (338.2 mg, 1.05 mmol, 1.5 equiv.), toluene (7.0 mL), triethylamine (293 µL, 2.10 mmol, 3.0 equiv.) and morpholine (92 µL, 1.05 mmol, 1.5 equiv.) were combined at room temperature for 1.5 hours. Flash column chromatography (SiO_2, Petrol/EtOAc = 1:1 to 1:2) afforded the desired sulfonimidamide **3p** as a light-yellow oil (243.3 mg, 0.54 mmol, 77%).

IR (ν_{max}, cm^{-1}) 3280, 1259, 1236, 1161, 1135, 1113, 976, 932, 759; **δH** (400 MHz, $CDCl_3$) 7.81 (d, *J* = 8.5 Hz, 2H), 7.44 (d, *J* = 8.5 Hz, 2H), 7.10 (d, *J* = 8.0 Hz, 2H), 7.04 (d, *J* = 8.0 Hz, 2H), 6.69 (s, 1H), 3.63 (t, *J* = 4.5 Hz, 4H), 3.72-3.42 (br. s, 1H), 2.91 (t, *J* = 4.5 Hz, 4H), 2.30 (s, 3H); **δC** (101 MHz, $CDCl_3$) 145.2, 143.9 (q, $^2J_{C-F}$ = 38.5 Hz), 142.4, 139.6, 134.5, 129.6, 129.0, 128.6, 125.6, 125.2, 121.0 (q, $^1J_{C-F}$ = 269.0 Hz), 106.1, 66.2, 47.0, 21.1; **δF** (377 MHz, $CDCl_3$) -62.3 (s); **HRMS** (ESI^+, m/z) calculated for $[C_{21}H_{22}F_3N_4O_2S]^+$ $[M+H]^+$ 451.1410, found 451.1407. Data for this compound was consistent with previous reports.[10]

1-(4-Fluorophenylsulfonimidoyl)piperidine-4-carbonitrile (3q)

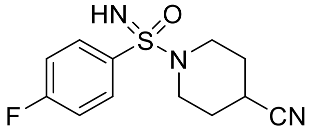

Following general procedure B1, 4-fluorobenzenesulfinamide **2d** (159.2 mg, 1.00 mmol, 1.0 equiv.), $PhI(OAc)_2$ (483.2 mg, 1.50 mmol, 1.5 equiv.), toluene (10.0 mL), triethylamine (418 µL, 3.00 mmol, 3.0 equiv.) and piperidine-4-carbonitrile (167 µL, 1.50 mmol, 1.5 equiv.) were combined at room temperature for 4 hours. Flash column chromatography (SiO_2, Petrol/EtOAc = 1:1 to 1:1.8) afforded the desired sulfonimidamide **3q** as a light-yellow solid (222.7 mg, 0.83 mmol, 83%).

M.p. 116-118 °C (CH_2Cl_2); **IR** (ν_{max}, cm^{-1}) 3280, 1589, 1491, 1258, 1137, 923; **δH** (400 MHz, $CDCl_3$) 7.86-7.76 (m, 2H), 7.19-7.09 (m, 2H), 3.13 (ddd, *J* = 11.5, 7.5, 3.5 Hz, 2H), 3.03-2.90 (m, 2H), 2.65-2.55 (m, 2H), 1.97-1.77 (m, 4H); **δC** (101 MHz, $CDCl_3$) 165.1 (d, $^1J_{C-F}$ = 254.5 Hz), 132.0 (d, $^4J_{C-F}$ = 3.0 Hz), 130.4 (d, $^3J_{C-F}$ = 9.0 Hz), 120.5, 116.1 (d, $^2J_{C-F}$ = 22.5 Hz), 44.9, 44.8, 28.3, 25.3 (note: for 2 secondary carbons attached to nitrogen in

piperidine ring, 2 peaks were found instead of 1 due to the loss of symmetry caused by chiral sulfur atom); **δF** (377 MHz, $CDCl_3$) -105.7 (tt, *J* = 8.5, 5.0 Hz); **HRMS** (ESI$^+$, m/z) calculated for $[C_{12}H_{15}FN_3OS]^+$ $[M+H]^+$ 268.0914, found 268.0913.

(1-(4-Fluorophenylsulfonimidoyl)piperidin-4-yl)(phenyl)methanone (3r)

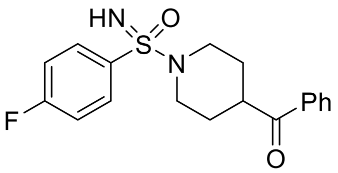

An oven-dried round-bottom flask containing 4-fluorobenzenesulfinamide **2d** (79.6 mg, 0.50 mmol, 1.0 equiv.), $PhI(OAc)_2$ (241.6 mg, 0.75 mmol, 1.5 equiv.) and 4-benzoylpiperidine hydrochloride (169.3 mg, 0.75 mmol, 1.5 equiv.) was sealed and subjected to three N_2 evacuation/refill cycles before pre-sparged anhydrous toluene (5.0 mL) was added. Triethylamine (314 µL, 2.25 mmol, 4.5 equiv.) was added to the solution. The reaction mixture was then stirred at room temperature for 6 hours. Flash column chromatography (SiO_2, Petrol/EtOAc = 1:0.8 to 1:1.2) afforded the desired sulfonimidamide **3r** as a yellow oil (140.5 mg, 0.41 mmol, 81%).

IR (v_{max}, cm^{-1}) 3280, 1677, 1256, 1133, 699; **δH** (400 MHz, $CDCl_3$) 7.88 (dd, *J* = 5.0, 2.0 Hz, 1H), 7.85 (dd, *J* = 5.0, 2.0 Hz, 1H), 7.80 (d, *J* = 8.0 Hz, 2H), 7.55-7.44 (m, 1H), 7.44-7.33 (m, 2H), 7.20-7.09 (m, 2H), 3.82 (dt, *J* = 12.0, 4.0 Hz, 2H), 3.13 (tt, *J* = 11.0, 4.0 Hz, 1H), 2.60 (s, 1H), 2.44-2.31 (m, 2H), 1.93-1.70 (m, 4H); **δC** (101 MHz, $CDCl_3$) δ 201.5, 165.0 (d, $^1J_{C-F}$ = 254.0 Hz), 135.5, 133.2, 132.1 (d, $^4J_{C-F}$ = 3.0 Hz), 130.6 (d, $^3J_{C-F}$ = 9.0 Hz), 128.7, 128.1, 115.9 (d, $^2J_{C-F}$ = 22.5 Hz), 46.7, 46.4, 42.3, 28.30, 28.26 (note: for 4 secondary carbons in piperidine ring, 4 peaks were found instead of 2 due to the loss of symmetry caused by chiral sulfur atom); **δF** (377 MHz, $CDCl_3$) -106.3 (tt, *J* = 8.0, 5.0 Hz); **HRMS** (ESI$^+$, m/z) calculated for $[C_{18}H_{20}FN_2O_2S]^+$ $[M+H]^+$ 347.1224, found 347.1227.

4-Fluoro-*N*-methyl-*N*-(pyridin-2-ylmethyl)benzenesulfonimidamide (3s)

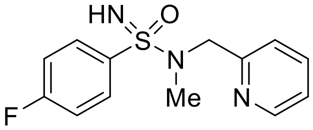

Following general procedure B1, 4-fluorobenzenesulfinamide **2d** (159.2 mg, 1.00 mmol, 1.0 equiv.), $PhI(OAc)_2$ (483.2 mg, 1.50 mmol, 1.5 equiv.), toluene (10.0 mL), triethylamine (418 µL, 3.00 mmol, 3.0 equiv.) and *N*-methyl-1-(pyridin-2-yl)methanamine (184 µL, 1.50 mmol, 1.5 equiv.) were combined at room temperature for 7 hours. Flash column chromatography (SiO_2, Petrol/EtOAc = 1:1.5 to 1:1.8) afforded the desired sulfonimidamide **3s** as a brown oil (251.3 mg, 0.90 mmol, 90%).

IR (v_{max}, cm^{-1}) 3280, 1589, 1490, 1258, 1227, 1137, 999, 921, 840; **δH** (400 MHz, $CDCl_3$) 8.49 (ddd, *J* = 5.0, 2.0,

1.0 Hz, 1H), 8.00-7.92 (m, 2H), 7.67 (app. td, J = 8.0, 2.0 Hz, 1H), 7.42 (d, J = 8.0 Hz, 1H), 7.22-7.13 (m, 3H), 4.35 (d, J = 15.0 Hz, 1H), 4.25 (d, J = 15.0 Hz, 1H), 2.80 (s, 1H), 2.69 (s, 3H); **δ_C** (101 MHz, $CDCl_3$) 165.1 (d, $^1J_{C\text{-}F}$ = 254.0 Hz), 156.9, 149.4, 137.0, 133.6 (d, $^4J_{C\text{-}F}$ = 3.0 Hz), 130.5 (d, $^3J_{C\text{-}F}$ = 9.0 Hz), 122.7, 122.2, 116.1 (d, $^2J_{C\text{-}F}$ = 22.5 Hz), 57.0, 36.4; **δ_F** (377 MHz, $CDCl_3$) -106.4 (tt, J = 8.5, 4.0 Hz); **HRMS** (ESI^+, m/z) calculated for $[C_{13}H_{15}FN_3OS]^+$ $[M+H]^+$ 280.0914, found 280.0915.

N-(Cyclohexylmethyl)-4-fluoro-N-methylbenzenesulfonimidamide (3t)

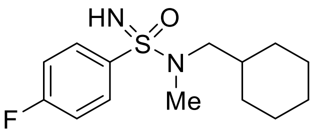

Following general procedure B1, 4-fluorobenzenesulfinamide **2d** (159.2 mg, 1.00 mmol, 1.0 equiv.), $PhI(OAc)_2$ (483.2 mg, 1.50 mmol, 1.5 equiv.), toluene (10.0 mL), triethylamine (418 µL, 3.00 mmol, 3.0 equiv.) and 1-cyclohexyl-*N*-methylmethanamine (229 µL, 1.50 mmol, 1.5 equiv.) were combined at room temperature for 3 hours. Flash column chromatography (SiO_2, Petrol/EtOAc = 6:1 to 3:1) afforded the desired sulfonimidamide **3t** as a yellow oil (253.9 mg, 0.89 mmol, 89%).

IR (ν_{max}, cm^{-1}) 3280, 1490, 1255, 976, 838, 722; **δ_H** (400 MHz, $CDCl_3$) 7.88-7.80 (m, 2H), 7.14-7.06 (m, 2H), 2.76 (dd, J = 13.0, 8.0 Hz, 1H), 2.64 (dd, J = 13.0, 8.0 Hz, 1H), 2.62 (s, 3H), 2.41 (s, 1H), 1.74-1.54 (m, 5H), 1.42 (ttt, J = 11.0, 7.0, 3.5 Hz, 1H), 1.23-1.01 (m, 3H), 0.92-0.73 (m, 2H); **δ_C** (101 MHz, $CDCl_3$) 164.8 (d, $^1J_{C\text{-}F}$ = 253.5 Hz), 133.6 (d, $^4J_{C\text{-}F}$ = 3.0 Hz), 130.2 (d, $^3J_{C\text{-}F}$ = 9.0 Hz), 115.8 (d, $^2J_{C\text{-}F}$ = 22.0 Hz), 57.2, 36.3, 36.0, 30.8, 26.4, 25.7; **δ_F** (377 MHz, $CDCl_3$) -107.0 (tt, J = 8.5, 4.0 Hz); **HRMS** (ESI^+, m/z) calculated for $[C_{14}H_{22}FN_2OS]^+$ $[M+H]^+$ 285.1431, found 285.1430.

5-(4-Fluorophenylsulfonimidoyl)-4,5,6,7-tetrahydrothieno[3,2-*c*]pyridine (3u)

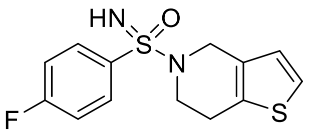

Following general procedure B1, 4-fluorobenzenesulfinamide **2d** (159.2 mg, 1.00 mmol, 1.0 equiv.), $PhI(OAc)_2$ (483.2 mg, 1.50 mmol, 1.5 equiv.), toluene (10.0 mL), triethylamine (418 µL, 3.00 mmol, 3.0 equiv.) and 4,5,6,7-tetrahydrothieno[3,2-*c*]pyridine (183 µL, 1.50 mmol, 1.5 equiv.) were combined at room temperature for 12 hours. Flash column chromatography (SiO_2, Petrol/EtOAc = 1.5:1 to 1:1) afforded the desired sulfonimidamide **3u** as a light-yellow solid (255.7 mg, 0.86 mmol, 86%).

M.p. 106-108 °C (chloroform); **IR** (ν_{max}, cm^{-1}) 3281, 1258, 1234, 750, 715; **δ_H** (400 MHz, $CDCl_3$) 7.99-7.89 (m, 2H), 7.19 -7.10 (m, 2H), 7.07 (d, J = 5.0 Hz, 1H), 6.69 (d, J = 5.0 Hz, 1H), 4.20 (d, J = 14.5 Hz, 1H), 4.16 (d, J = 14.5 Hz, 1H), 3.49-3.33 (m, 2H), 2.86 (t, J = 6.0 Hz, 2H), 2.71 (s, 1H); **δ_C** (101 MHz, $CDCl_3$) 165.1 (d, $^1J_{C\text{-}F}$ =

254.5 Hz), 132.9 (d, $^4J_{C-F}$ = 3.5 Hz), 132.8, 131.3, 130.5 (d, $^3J_{C-F}$ = 9.0 Hz), 124.8, 123.8, 116.1 (d, $^2J_{C-F}$ = 22.5 Hz), 47.0, 44.9, 25.6; **δF** (377 MHz, $CDCl_3$) -106.1 (tt, *J* = 8.5, 5.0 Hz); **HRMS** (ESI^+, m/z) calculated for $[C_{13}H_{14}FN_2OS_2]^+$ $[M+H]^+$ 297.0526, found 297.0527.

3-(4-(4-Fluorophenylsulfonimidoyl)piperazin-1-yl)benzo[*d*]isothiazole (3v)

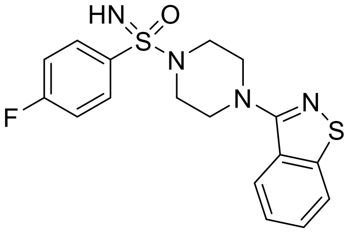

Following general procedure B1, 4-fluorobenzenesulfinamide **2d** (79.6 mg, 0.50 mmol, 1.0 equiv.), $PhI(OAc)_2$ (241.6 mg, 0.75 mmol, 1.5 equiv.), toluene (5.0 mL), triethylamine (209 µL, 1.50 mmol, 3.0 equiv.) and 3-(piperazin-1-yl)benzo[*d*]isothiazole (164.5 mg, 0.75 mmol, 1.5 equiv.) were combined at room temperature for 3 hours. Flash column chromatography (SiO_2, Petrol/EtOAc = 1:0.6 to 1:0.8) afforded the desired sulfonimidamide **3v** as a light-yellow solid (163.0 mg, 0.43 mmol, 86%).

M.p. 50-52 °C (*n*-hexane); **IR** (v_{max}, cm^{-1}) 3273, 1490, 1259, 1135, 931, 740; **δH** (400 MHz, $CDCl_3$) 7.98-7.88 (m, 2H), 7.81-7.73 (m, 2H), 7.44 (ddd, *J* = 8.0, 7.0, 1.0 Hz, 1H), 7.31 (ddd, *J* = 8.0, 7.0, 1.0 Hz, 1H), 7.25-7.15 (m, 2H), 3.58 (t, *J* = 4.5 Hz, 4H), 3.23 (t, *J* = 4.5 Hz, 4H), 2.66 (s, 1H); **δC** (101 MHz, $CDCl_3$) 165.3 (d, $^1J_{C-F}$ = 254.5 Hz), 163.1, 152.9, 131.7 (d, $^4J_{C-F}$ = 3.0 Hz), 130.8 (d, $^3J_{C-F}$ = 9.0 Hz), 127.8, 127.7, 124.2, 123.5, 120.7, 116.2 (d, $^2J_{C-F}$ = 22.5 Hz), 49.8, 46.9; **δF** (377 MHz, $CDCl_3$) -105.8 (tt, *J* = 8.5, 5.0 Hz); **HRMS** (ESI^+, m/z) calculated for $[C_{17}H_{18}FN_4OS_2]^+$ $[M+H]^+$ 377.0901, found 377.0905.

2-(4-(4-Fluorophenylsulfonimidoyl)piperazin-1-yl)pyrimidine (3w)

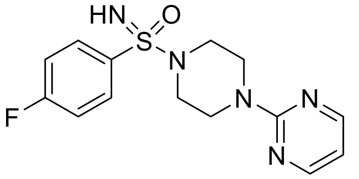

Following general procedure B1, 4-fluorobenzenesulfinamide **2d** (79.6 mg, 0.50 mmol, 1.0 equiv.), $PhI(OAc)_2$ (241.6 mg, 0.75 mmol, 1.5 equiv.), toluene (5.0 mL), triethylamine (209 µL, 1.50 mmol, 3.0 equiv.) and 2-(piperazin-1-yl)pyrimidine (106 µL, 0.75 mmol, 1.5 equiv.) were combined at room temperature for 2 hours. Flash column chromatography (SiO_2, Petrol/EtOAc = 1:2 to 1:4) afforded the desired sulfonimidamide **3w** as a white solid (150.2 mg, 0.47 mmol, 94%).

M.p. 138-140 °C (*n*-hexane); **IR** (v_{max}, cm^{-1}) 3267, 1584, 1490, 1260, 953; **δH** (400 MHz, $CDCl_3$) 8.23 (d, *J* = 4.5 Hz, 2H), 7.91-7.81 (m, 2H), 7.19-7.09 (m, 2H), 6.45 (t, *J* = 4.5 Hz, 1H), 3.87 (t, *J* = 5.0 Hz, 4H), 3.02 (t, *J* = 5.0 Hz, 4H), 2.77 (s, 1H); **δC** (101 MHz, $CDCl_3$) 165.2 (d, $^1J_{C-F}$ = 254.5 Hz), 161.1, 157.8, 131.5 (d, $^4J_{C-F}$ = 3.0 Hz),

130.8 (d, $^{3}J_{C\text{-}F}$ = 9.0 Hz), 116.1 (d, $^{2}J_{C\text{-}F}$ = 22.5 Hz), 110.5, 47.0, 43.4; **δF** (377 MHz, $CDCl_3$) -106.0 (tt, *J* = 8.0, 5.0 Hz); **HRMS** (ESI^+, m/z) calculated for $[C_{14}H_{16}FN_5OSNa]^+$ $[M+Na]^+$ 344.0952, found 344.0954.

2-Chloro-11-(4-(4-fluorophenylsulfonimidoyl)piperazin-1-yl)dibenzo[*b*,*f*][1,4]oxazepine (3x)

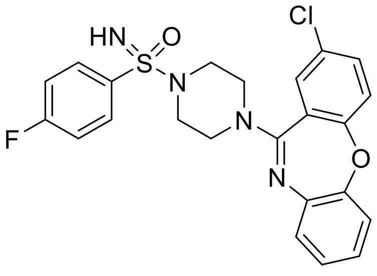

Following general procedure B1, 4-fluorobenzenesulfinamide **2d** (47.8 mg, 0.30 mmol, 1.0 equiv.), $PhI(OAc)_2$ (144.9 mg, 0.45 mmol, 1.5 equiv.), toluene (3.0 mL), triethylamine (125 µL, 0.90 mmol, 3.0 equiv.) and Amoxapine (141.2 mg, 0.45 mmol, 1.5 equiv.) were combined at room temperature for 5 hours. Flash column chromatography (SiO_2, Petrol/EtOAc = 1:0.5 to 1:0.7) afforded the desired sulfonimidamide **3x** as a white solid (124.2 mg, 0.26 mmol, 87%).

M.p. 162-164 °C (*n*-hexane); **IR** (ν_{max}, cm^{-1}) 3329, 1588, 1254, 1137, 1107, 936, 909, 733; **δH** (400 MHz, $(CD_3)_2SO$) 7.91-7.82 (m, 2H), 7.58 (dd, *J* = 8.5, 2.5 Hz, 1H), 7.48-7.32 (m, 4H), 7.16 (dd, *J* = 8.0, 1.5 Hz, 1H), 7.11-6.95 (m, 3H), 4.64 (s, 1H), 3.51 (app. br. s, 4H), 3.00 (app. br. s, 4H); **δC** (101 MHz, $(CD_3)_2SO$) 164.2 (d, $^{1}J_{C\text{-}F}$ = 250.5 Hz), 158.6, 157.8, 151.1, 139.6, 133.1, 132.8 (d, $^{4}J_{C\text{-}F}$ = 3.0 Hz), 130.7 (d, $^{3}J_{C\text{-}F}$ = 9.5 Hz), 129.6, 128.7, 126.5, 125.8, 124.5, 124.2, 123.0, 120.2, 116.0 (d, $^{2}J_{C\text{-}F}$ = 22.5 Hz), 46.5, 46.2; **δF** (377 MHz, $(CD_3)_2SO$) -107.6 (tt, *J* = 9.0, 5.0 Hz); **HRMS** (ESI^+, m/z) calculated for $[C_{23}H_{21}{}^{35}ClFN_4O_2S]^+$ $[M+H]^+$ 471.1052, found 471.1045. Data for this compound closely matched previous reports.[10]

3.4.2 General procedure B2 - Reaction between primary sulfinamide and primary amine

An oven-dried round-bottom flask containing primary sulfinamide **2** (1.0 equiv.) and $PhI(OC(O)t\text{-}Bu)_2$ (2.5 equiv.) was sealed and subjected to three N_2 evacuation/refill cycles before pre-sparged anhydrous MeCN (sulfinamide conc. 0.1 M) was added. Triethylamine (12.0 equiv.) was added to the solution, followed immediately by the addition of primary amine (2.0 equiv.). The reaction was heated at 60 °C in an aluminium heating block for 16 hours and monitored by TLC. The reaction mixture was then diluted with EtOAc (10 mL) and quenched with saturated aqueous solution of $NaHCO_3$ (10 mL). The aqueous phase was extracted with EtOAc (3 × 10 mL). The combined organic layers were then dried over anhydrous Na_2SO_4, filtered and concentrated *in vacuo*. Sulfonimidamide **4** was purified by flash column chromatography with the appropriate solvent system.

N'-Butyl-4-fluorobenzenesulfonimidamide (4a)

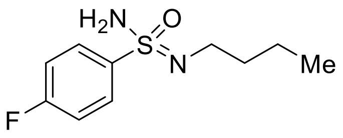

Following general procedure B2, 4-fluorobenzenesulfinamide **2d** (79.6 mg, 0.50 mmol, 1.0 equiv.), PhI(OC(O)*t*-Bu)$_2$ (507.8 mg, 1.25 mmol, 2.5 equiv.), MeCN (5.0 mL), triethylamine (836 µL, 6.00 mmol, 12.0 equiv.) and *n*-butyl amine (99 µL, 1.00 mmol, 2.0 equiv.) were heated at 60 °C for 16 hours. Flash column chromatography (SiO_2, Petrol/EtOAc = 1:1 to 1:1.3) afforded the desired sulfonimidamide **4a** as a light-yellow oil (82.3 mg, 0.36 mmol, 72%).

IR (ν_{max}, cm^{-1}) 3259, 1590, 1492, 1234, 1008, 838; **δ_H** (400 MHz, $CDCl_3$) 8.02-7.92 (m, 2H), 7.18-7.08 (m, 2H), 5.51-2.71 (br. s, 2H), 2.89 (dt, *J* = 12.5, 7.0 Hz, 1H), 2.82 (dt, *J* = 12.5, 7.0 Hz, 1H), 1.43-1.31 (m, 2H), 1.31-1.16 (m, 2H), 0.80 (t, *J* = 7.5 Hz, 3H); **δ_C** (101 MHz, $CDCl_3$) 164.9 (d, $^1J_{C-F}$ = 253.5 Hz), 137.6 (d, $^4J_{C-F}$ = 3.0 Hz), 130.0 (d, $^3J_{C-F}$ = 9.0 Hz), 116.1 (d, $^2J_{C-F}$ = 22.5 Hz), 43.3, 31.7, 19.9, 13.6; **δ_F** (377 MHz, $CDCl_3$) -106.8 (tt, *J* = 8.5, 5.0 Hz); **HRMS** (ESI^+, m/z) calculated for $[C_{10}H_{16}FN_2OS]^+$ $[M+H]^+$ 231.0962, found 231.0960.

N'-(Cyclohexylmethyl)-4-fluorobenzenesulfonimidamide (4b)

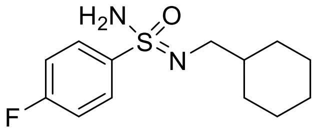

Following general procedure B2, 4-fluorobenzenesulfinamide **2d** (79.6 mg, 0.50 mmol, 1.0 equiv.), PhI(OC(O)*t*-Bu)$_2$ (507.8 mg, 1.25 mmol, 2.5 equiv.), MeCN (5.0 mL), triethylamine (836 µL, 6.00 mmol, 12.0 equiv.) and cyclohexylmethanamine (130 µL, 1.00 mmol, 2.0 equiv.) were heated at 60 °C for 16 hours. Flash column chromatography (SiO_2, CH_2Cl_2/EtOAc = 2.5:1 to 2:1) afforded the desired sulfonimidamide **4b** as a yellow oil (89.9 mg, 0.33 mmol, 66%).

IR (ν_{max}, cm^{-1}) 3259, 1590, 1492, 1233, 1009, 836; **δ_H** (400 MHz, $CDCl_3$) 8.02-7.90 (m, 2H), 7.19-7.08 (m, 2H), 5.97-1.75 (br. s, 2H), 2.72 (dd, *J* = 12.5, 6.5 Hz, 1H), 2.66 (dd, *J* = 12.5, 6.5 Hz, 1H), 1.74-1.54 (m, 5H), 1.32 (dddd, *J* = 11.5, 6.5, 5.0, 3.5, 1H), 1.21-0.99 (m, 3H), 0.91-0.72 (m, 2H); **δ_C** (101 MHz, $CDCl_3$) 164.9 (d, $^1J_{C-F}$ = 253.5 Hz), 137.6 (d, $^4J_{C-F}$ = 3.0 Hz), 130.0 (d, $^3J_{C-F}$ = 9.0 Hz), 116.0 (d, $^2J_{C-F}$ = 22.5 Hz), 49.9, 37.9, 30.8, 30.7, 26.4, 25.8 (note: for 2 secondary carbons attached to tertiary carbon in cyclohexane ring, 2 peaks were found instead of 1 due to the loss of symmetry caused by chiral sulfur atom); **δ_F** (377 MHz, $CDCl_3$) -106.8 (tt, *J* = 8.5, 5.0 Hz); **HRMS** (ESI^+, m/z) calculated for $[C_{13}H_{20}FN_2OS]^+$ $[M+H]^+$ 271.1275, found 271.1271.

4-Fluoro-*N*'-phenethylbenzenesulfonimidamide (4c)

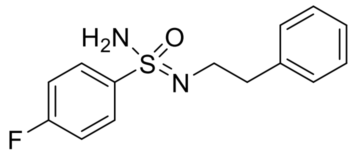

Following general procedure B2, 4-fluorobenzenesulfinamide **2d** (31.8 mg, 0.20 mmol, 1.0 equiv.), PhI(OC(O)*t*-Bu)$_2$ (203.1 mg, 0.50 mmol, 2.5 equiv.), MeCN (2.0 mL), triethylamine (335 µL, 2.40 mmol, 12.0 equiv.) and 2-phenylethan-1-amine (50 µL, 0.40 mmol, 2.0 equiv.) were heated at 60 °C for 16 hours. Flash column chromatography (SiO$_2$, Petrol/EtOAc = 1:1 to 1:2) afforded the desired sulfonimidamide **4c** as a light-yellow oil (36.3 mg, 0.13 mmol, 65%).

IR (ν_{max}, cm^{-1}) 3259, 1590, 1492, 1233, 1008; **δ$_H$** (400 MHz, CDCl$_3$) 7.97-7.87 (m, 2H), 7.29-7.17 (m, 3H), 7.16-7.09 (m, 2H), 7.09-7.01 (m, 2H), 3.91 (br. s, 2H), 3.22 (dt, *J* = 12.5, 7.0 Hz, 1H), 3.14 (dt, *J* = 12.5, 7.0 Hz, 1H), 2.73 (app. td, *J* = 7.0, 2.0 Hz, 2H); **δ$_C$** (101 MHz, CDCl$_3$) 164.9 (d, $^1J_{C-F}$ = 254.0 Hz), 138.1, 137.3 (d, $^4J_{C-F}$ = 3.0 Hz), 130.0 (d, $^3J_{C-F}$ = 9.0 Hz), 128.81, 128.79, 126.8, 116.2 (d, $^2J_{C-F}$ = 22.5 Hz), 44.8, 36.0; **δ$_F$** (377 MHz, CDCl$_3$) -106.3 (tt, *J* = 8.5, 5.0 Hz); **HRMS** (ESI$^+$, m/z) calculated for [$C_{14}H_{16}FN_2OS$]$^+$ [M+H]$^+$ 279.0962, found 279.0960.

***N*'-Cyclopentyl-4-fluorobenzenesulfonimidamide (4d)**

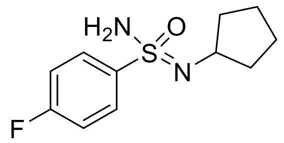

Following general procedure B2, 4-fluorobenzenesulfinamide **2d** (79.6 mg, 0.50 mmol, 1.0 equiv.), PhI(OC(O)*t*-Bu)$_2$ (507.8 mg, 1.25 mmol, 2.5 equiv.), MeCN (5.0 mL), triethylamine (836 µL, 6.00 mmol, 12.0 equiv.) and cyclopentylamine (99 µL, 1.00 mmol, 2.0 equiv.) were heated at 60 °C for 16 hours. Flash column chromatography (SiO$_2$, Petrol/EtOAc = 2:1 to 1.5:1) afforded the desired sulfonimidamide **4d** as a yellow oil (73.8 mg, 0.30 mmol, 61%).

IR (ν_{max}, cm^{-1}) 3252, 1590, 1492, 1232, 1010; **δ$_H$** (400 MHz, CDCl$_3$) 8.05-7.94 (m, 2H), 7.19-7.08 (m, 2H), 5.43-2.64 (br. s, 2H), 3.54 (pent., *J* = 7.0 Hz, 1H), 1.83-1.71 (m, 1H), 1.71-1.15 (m, 7H); **δ$_C$** (101 MHz, CDCl$_3$) 164.9 (d, $^1J_{C-F}$ = 253.5 Hz), 138.5 (d, $^4J_{C-F}$ = 2.5 Hz), 130.1 (d, $^3J_{C-F}$ = 9.0 Hz), 116.1 (d, $^2J_{C-F}$ = 22.5 Hz), 55.5, 33.6, 33.3, 23.3, 23.2; (note: for 4 secondary carbons in cyclopentane ring, 4 peaks were found instead of 2 due to the loss of symmetry caused by chiral sulfur atom); **δ$_F$** (377 MHz, CDCl$_3$) -106.8 (tt, *J* = 8.0, 5.0 Hz); **HRMS** (ESI$^+$, m/z) calculated for [$C_{11}H_{16}FN_2OS$]$^+$ [M+H]$^+$ 243.0962, found 243.0959.

***N*'-Cyclobutyl-4-fluorobenzenesulfonimidamide (4e)**

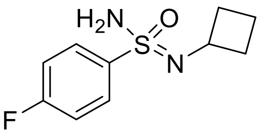

Following general procedure B2, 4-fluorobenzenesulfinamide **2d** (79.6 mg, 0.50 mmol, 1.0 equiv.), PhI(OC(O)*t*-Bu)$_2$ (507.8 mg, 1.25 mmol, 2.5 equiv.), MeCN (5.0 mL), triethylamine (836 µL, 6.00 mmol, 12.0 equiv.) and cyclobutylamine (85 µL, 1.00 mmol, 2.0 equiv.) were heated at 60 °C for 16 hours. Flash column chromatography (SiO_2, Petrol/EtOAc = 1:0.8 to 1:1.2) afforded the desired sulfonimidamide **4e** as a light-yellow oil (64.3 mg, 0.28 mmol, 56%).

IR (ν_{max}, cm^{-1}) 3256, 1590, 1491, 1234, 1009, 838; **δ_H** (400 MHz, $CDCl_3$) 8.04-7.93 (m, 2H), 7.18-7.07 (m, 2H), 6.04-2.52 (br. s, 2H), 3.80-3.68 (m, 1H), 2.12-2.01 (m, 1H), 2.00-1.90 (m, 1H), 1.82-1.61 (m, 2H), 1.60-1.42 (m, 2H); **δ_C** (101 MHz, $CDCl_3$) 164.9 (d, $^1J_{C-F}$ = 253.5 Hz), 138.5 (d, $^4J_{C-F}$ = 3.0 Hz), 130.0 (d, $^3J_{C-F}$ = 9.0 Hz), 116.0 (d, $^2J_{C-F}$ = 22.5 Hz), 48.5, 31.8, 31.7, 15.1 (note: for 2 secondary carbons attached to tertiary carbon in cyclobutane ring, 2 peaks were found instead of 1 due to the loss of symmetry caused by chiral sulfur atom); **δ_F** (377 MHz, $CDCl_3$) -106.7 (tt, *J* = 8.5, 5.0 Hz); **HRMS** (ESI$^+$, m/z) calculated for $[C_{10}H_{14}FN_2OS]^+$ $[M+H]^+$ 229.0805, found 229.0808. Data for this compound was consistent with previous reports.[10]

***N*'-(But-3-yn-1-yl)-4-fluorobenzenesulfonimidamide (4f)**

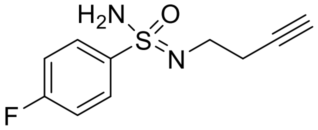

An oven-dried round-bottom flask containing 4-fluorobenzenesulfinamide **2d** (79.6 mg, 0.50 mmol, 1.0 equiv.), PhI(OC(O)*t*-Bu)$_2$ (507.8 mg, 1.25 mmol, 2.5 equiv.) and but-3-yn-1-amine hydrochloride (105.6 mg, 1.00 mmol, 2.0 equiv.) was sealed and subjected to three N_2 evacuation/refill cycles before pre-sparged anhydrous MeCN (5.0 mL) was added. Triethylamine (836 µL, 6.00 mmol, 12.0 equiv.) was added to the solution. The reaction mixture was then heated at 60 °C in an aluminium heating block for 16 hours. Flash column chromatography (SiO_2, Petrol/EtOAc = 2:1 to 1:1) afforded the desired sulfonimidamide **4f** as a light-yellow oil (58.9 mg, 0.26 mmol, 52%).

IR (ν_{max}, cm^{-1}) 3287, 1590, 1492, 1233, 1007, 838; **δ_H** (400 MHz, $CDCl_3$) 8.05-7.95 (m, 2H), 7.21-7.10 (m, 2H), 4.11 (br. s, 2H), 3.15-2.99 (m, 2H), 2.30 (td, *J* = 6.5, 2.5 Hz, 2H), 1.97 (t, *J* = 2.5 Hz, 1H); **δ_C** (101 MHz, $CDCl_3$) 165.1 (d, $^1J_{C-F}$ = 254.0 Hz), 137.4 (d, $^4J_{C-F}$ = 3.0 Hz), 130.0 (d, $^3J_{C-F}$ = 9.0 Hz), 116.3 (d, $^2J_{C-F}$ = 22.5 Hz), 80.8, 70.8, 42.3, 20.0; **δ_F** (377 MHz, $CDCl_3$) -106.3 (tt, *J* = 8.5, 5.0 Hz); **HRMS** (ESI$^+$, m/z) calculated for $[C_{10}H_{12}FN_2OS]^+$ $[M+H]^+$ 227.0649, found 227.0650.

4-Fluoro-*N*'-(hex-5-yn-1-yl)benzenesulfonimidamide (4g)

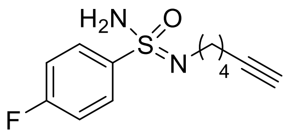

Following general procedure B2, 4-fluorobenzenesulfinamide **2d** (79.6 mg, 0.50 mmol, 1.0 equiv.), PhI(OC(O)*t*-Bu)$_2$ (507.8 mg, 1.25 mmol, 2.5 equiv.), MeCN (5.0 mL), triethylamine (836 µL, 6.00 mmol, 12.0 equiv.) and hex-5-yn-1-amine (115 µL, 1.00 mmol, 2.0 equiv.) were heated at 60 °C for 16 hours. Flash column chromatography (SiO$_2$, Petrol/EtOAc = 2:1 to 1:1) afforded the desired sulfonimidamide **4g** as a light-yellow oil (70.0 mg, 0.28 mmol, 55%).

IR (ν_{max}, cm^{-1}) 3289, 1590, 1492, 1233, 1008, 839; **δ_H** (400 MHz, CDCl$_3$) 8.02-7.92 (m, 2H), 7.20-7.10 (m, 2H), 4.13 (s, 2H), 2.98-2.80 (m, 2H), 2.11 (td, *J* = 6.5, 2.5 Hz, 2H), 1.90 (t, *J* = 2.5 Hz, 1 H), 1.59-1.40 (m, 4H); **δ_C** (101 MHz, CDCl$_3$) 165.0 (d, $^1J_{C\text{-}F}$ = 254.0 Hz), 137.4 (d, $^4J_{C\text{-}F}$ = 3.0 Hz), 130.1 (d, $^3J_{C\text{-}F}$ = 9.0 Hz), 116.2 (d, $^2J_{C\text{-}F}$ = 22.5 Hz), 83.8, 68.9, 43.0, 28.6, 25.5, 18.0; **δ_F** (377 MHz, CDCl$_3$) -106.5 (tt, *J* = 8.5, 5.0 Hz); **HRMS** (ESI$^+$, m/z) calculated for $[C_{12}H_{16}FN_2OS]^+$ $[M+H]^+$ 255.0962, found 255.0962.

***N*'-(But-3-en-1-yl)-4-fluorobenzenesulfonimidamide (4h)**

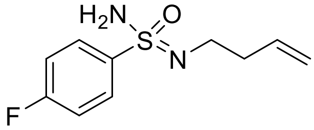

Following general procedure B2, 4-fluorobenzenesulfinamide **2d** (79.6 mg, 0.50 mmol, 1.0 equiv.), PhI(OC(O)*t*-Bu)$_2$ (507.8 mg, 1.25 mmol, 2.5 equiv.), MeCN (5.0 mL), triethylamine (836 µL, 6.00 mmol, 12.0 equiv.) and but-3-en-1-amine (92 µL, 1.00 mmol, 2.0 equiv.) were heated at 60 °C for 16 hours. Flash column chromatography (SiO$_2$, Petrol/EtOAc = 1:0.8 to 1:1.2) afforded the desired sulfonimidamide **4h** as a colourless oil (53.1 mg, 0.23 mmol, 47%).

IR (ν_{max}, cm^{-1}) 3257, 1590, 1492, 1233, 994, 838; **δ_H** (400 MHz, CDCl$_3$) 8.02-7.91 (m, 2H), 7.20-7.09 (m, 2H), 5.60 (ddt, *J* = 17.0, 10.5, 7.0 Hz, 1H), 5.07-4.93 (m, 2H), 4.90-3.14 (br. s, 2H), 3.00 (dt, *J* = 12.5, 7.0 Hz, 1H), 2.93 (dt, *J* = 12.5, 7.0 Hz, 1H), 2.17 (dt, *J* = 7.0, 1.5 Hz, 1H), 2.13 (dt, *J* = 7.0, 1.5 Hz, 1H); **δ_C** (101 MHz, CDCl$_3$) 165.0 (d, $J^1_{C\text{-}F}$ = 254.0 Hz), 137.4 (d, $J^4_{C\text{-}F}$ = 3.5 Hz), 134.6, 130.1 (d, $J^3_{C\text{-}F}$ = 9.0 Hz), 117.8, 116.1 (d, $J^2_{C\text{-}F}$ = 22.5 Hz), 42.8, 33.8; **δ_F** (377 MHz, CDCl$_3$) -106.5 (tt, *J* = 8.5, 5.0 Hz); **HRMS** (ESI$^+$, m/z) calculated for $[C_{10}H_{14}FN_2OS]^+$ $[M+H]^+$ 229.0805, found 229.0806.

N'-Butylthiophene-2-sulfonimidamide (4i)

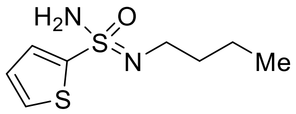

Following general procedure B2, thiophene-2-sulfinamide **2i** (52.3 mg, 0.36 mmol, 1.0 equiv.), PhI(OC(O)*t*-Bu)$_2$ (365.6 mg, 090 mmol, 2.5 equiv.), MeCN (3.6 mL), triethylamine (602 µL, 4.32 mmol, 12.0 equiv.) and *n*-butyl amine (71 µL, 0.72 mmol, 2.0 equiv.) were heated at 60 °C for 16 hours. Flash column chromatography (SiO_2, Petrol/EtOAc = 1:1 to 1:1.5) afforded the desired sulfonimidamide **4i** as a light-yellow oil (53.7 mg, 0.25 mmol, 69%).

IR (ν_{max}, cm^{-1}) 3259, 1246, 1012, 712; **δ_H** (400 MHz, $CDCl_3$) 7.60 (dd, *J* = 3.5, 1.5 Hz, 1H), 7.52 (dd, *J* = 5.0, 1.5 Hz, 1H), 7.05 (dd, *J* = 5.0, 3.5 Hz, 1H), 3.88 (br. s, 2H), 3.08-2.91 (m, 2H), 1.49-1.37 (m, 2H), 1.35-1.22 (m, 2H), 0.84 (t, *J* = 7.5 Hz, 3H); **δ_C** (101 MHz, $CDCl_3$) 143.4, 131.8, 131.7, 127.5, 43.7, 31.6, 19.9, 13.7; **HRMS** (ESI$^+$, m/z) calculated for $[C_8H_{15}N_2OS_2]^+$ $[M+H]^+$ 219.0620, found 219.0620.

3.5 Synthesis of 4-ethoxy-3-(1-methyl-7-oxo-3-propyl-6,7-dihydro-1*H*-pyrazolo[4,3-*d*]pyrimidin-5-yl)benzenesulfinamide (6)

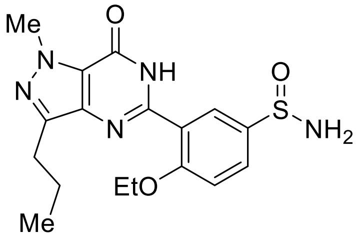

To a solution of 5-(5-bromo-2-ethoxyphenyl)-1-methyl-3-propyl-1,6-dihydro-7*H*-pyrazolo[4,3-*d*]pyrimidin-7-one **5**[11] (266.0 mg, 0.68 mmol, 1.0 equiv.) in THF (3.4 mL, **5** conc. 0.2 M) in an oven-dried 50 mL round-bottom flask was added methyllithium solution (0.51 mL, 0.82 mmol, 1.60 M in Et_2O, 1.2 equiv.) dropwise at -78 °C. The reaction was stirred at -78 °C for 10 minutes. *n*-Butyllithium solution (0.45 mL, 1.02 mmol, 2.25 M in hexanes, 1.5 equiv.) was added dropwise. The reaction mixture was stirred at the same temperature for 50 minutes. Then a solution of TIPS-NSO (223.8 mg, 1.02 mmol, 1.5 equiv.) in THF (2.6 mL) was added. After being stirred at -78 °C for 30 minutes, the reaction was stirred at 0 °C for 30 minutes and then at room temperature for another 30 minutes. The reaction was cooled to 0 °C, prior to the addition of tetrabutylammonium fluoride (TBAF) solution (1.36 mL, 1.36 mmol, 1.0 M in THF, 2.0 equiv.). The reaction was then stirred at the same temperature for 10 minutes before being diluted with EtOAc (10 mL) and quenched with saturated aqueous solution of EDTA tetrasodium salt (10 mL). The aqueous phase was extracted with EtOAc (3 × 10 mL). The combined organic layers were then washed with water (10 mL) and brine (10 mL), dried over anhydrous Na_2SO_4, filtered, and concentrated *in vacuo*. Flash column chromatography (SiO_2, EtOAc/EtOH = 1:0 to 15:1 to 10:1) afforded the desired sulfinamide **6** as a white solid (227.4 mg, 0.61 mmol, 89%).

M.p. 184-186 °C (*n*-hexane); **IR** (ν_{max}, cm^{-1}) 3226, 1693, 1465, 1166, 1039; **δ_H** (400 MHz, $(CD_3)_2SO$) 11.98 (s, 1H), 7.88 (d, *J* = 2.5 Hz, 1H), 7.71 (dd, *J* = 9.0, 2.5 Hz, 1H), 7.29 (d, *J* = 9.0 Hz, 1H), 6.27 (s, 2H), 4.17 (q, *J* = 7.0 Hz, 2H), 4.16 (s, 3H), 2.78 (t, *J* = 7.5 Hz, 2H), 1.74 (sext., *J* = 7.5 Hz, 2H), 1.34 (t, *J* = 7.0 Hz, 3H), 0.93 (t, *J* = 7.5 Hz, 3H); **δ_C** (101 MHz, $(CD_3)_2SO$) 158.0, 153.7, 149.0, 144.9, 139.5, 137.9, 129.1, 127.7, 124.3, 122.8, 112.9, 64.5, 37.8, 27.1, 21.7, 14.4, 13.8; **HRMS** (ESI$^+$, m/z) calculated for $[C_{17}H_{22}N_5O_3S]^+$ $[M+H]^+$ 376.1438, found 376.1437.

3.6 Synthesis of 5-(2-ethoxy-5-(4-methylpiperazine-1-sulfonimidoyl)phenyl)-1-methyl-3-propyl-1,6-dihydro-7*H*-pyrazolo[4,3-*d*]pyrimidin-7-one (7)

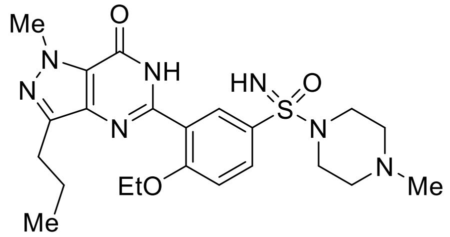

An oven-dried round-bottom flask containing 4-ethoxy-3-(1-methyl-7-oxo-3-propyl-6,7-dihydro-1*H*-pyrazolo[4,3-*d*]pyrimidin-5-yl)benzenesulfinamide **6** (112.6 mg, 0.30 mmol, 1.00 equiv.) and $PhI(OAc)_2$ (193.3 mg, 0.60 mmol, 2.00 equiv.) was sealed and subjected to three N_2 evacuation/refill cycles before pre-sparged anhydrous MeCN (1.5 mL) was added. DBU (179 µL, 1.20 mmol, 4.00 equiv.) and subsequently 1-methylpiperazine (67 µL, 0.60 mmol, 2.00 equiv.) were added dropwise to the solution. The reaction mixture was then stirred at room temperature for 3 hours before being diluted with EtOAc (10 mL) and quenched with saturated aqueous solution of $NaHCO_3$ (10 mL). The aqueous phase was extracted with EtOAc (3 × 10 mL). The combined organic layers were then dried over anhydrous Na_2SO_4, filtered, and concentrated *in vacuo*. Flash column chromatography (SiO_2, EtOAc/EtOH/NEt_3 = 1:0:0 to 1:3:0.04 to 1:4:0.05) afforded the desired sulfonimidamide **7** as a light-yellow solid (91.0 mg, 0.19 mmol, 64%).

M.p. 148-150 °C (Et_2O); **IR** (ν_{max}, cm^{-1}) 3299, 1693, 1264, 1140; **δ_H** (400 MHz, $CDCl_3$) 10.85 (s, 1H), 8.83 (d, *J* = 2.5 Hz, 1H), 7.92 (dd, *J* = 9.0, 2.5 Hz, 1H), 7.09 (d, *J* = 9.0 Hz, 1H), 4.33 (q, *J* = 7.0 Hz, 2H), 4.24 (s, 3H), 3.08 (app. s, 4H), 2.90 (t, *J* = 7.5 Hz, 2H), 2.62 (br. s, 1H), 2.46 (t, *J* = 5.0 Hz, 4H), 2.25 (s, 3H), 1.83 (sext., *J* = 7.5 Hz, 2H), 1.60 (t, *J* = 7.0 Hz, 3H), 0.99 (t, *J* = 7.5 Hz, 3H); **δ_C** (101 MHz, $CDCl_3$) 159.2, 153.8, 147.0, 146.8, 138.6, 132.2, 131.5, 129.0, 124.6, 121.0, 112.9, 66.1, 54.6, 47.0, 45.7, 38.3, 27.8, 22.3, 14.6, 14.1; **HRMS** (ESI$^+$, m/z) calculated for $[C_{22}H_{32}N_7O_3S]^+$ $[M+H]^+$ 474.2282, found 474.2281. Data for this compound was consistent with previous reports.[12]

4. References

[1] Savile, C. K.; Magloire. V. P.; Kazlauskas, R. J. Subtilisin-Catalyzed Resolution of *N*-Acyl Arylsulfinamides. *J. Am. Chem. Soc.* **2005**, *127*(7), 2104–2113.

[2] Ma, L.; Li, G.; Huang, J.; Zhu, J.; Tang, Z. Synthesis of asymmetrical thioethers with sulfinamides as the sulfenylation agent under metal-free conditions. *Tetrahedron Lett.* **2018**, *59*(48), 4255–4258.

[3] Lo, P.K.T.; Oliver, G. A.; Willis, M. C. Sulfinamide Synthesis Using Organometallic Reagents, DABSO, and Amines. *J. Org. Chem.* **2020**, *85*(9), 5753–5760.

[4] Chatterjee, S.; Makai, S.; Morandi, B. Hydroxylamine-Derived Reagent as a Dual Oxidant and Amino Group Donor for the Iron-Catalyzed Preparation of Unprotected Sulfinamides from Thiols. *Angew. Chem. Int. Ed.* **2021**, *60*(2), 758-765.

[5] Di, J.; He, H.; Wang, F.; Xue, F.; Liu, X. Y; Qin, Y. Regiospecific alkyl addition of (hetero)arene-fused thiophenes enabled by a visible-light-mediated photocatalytic desulfuration approach. *Chem. Commun.* **2018**, *54*(37), 4692–4695.

[6] Xue, F.; Wang, F.; Liu, J.; Di, J.; Liao, Q.; Lu, H.; Zhu, M.; He, L.; He, H.; Zhang, D.; Song, H.; Liu, X. Y.; Qin, Y. A Desulfurative Strategy for the Generation of Alkyl Radicals Enabled by Visible-Light Photoredox Catalysis. *Angew. Chem. Int. Ed.* **2018**, *57*(22), 6667–6671.

[7] Uddin, M. J.; Rao, P. N. P.; Knaus, E. E. Design and synthesis of novel celecoxib analogues as selective cyclooxygenase-2 (COX-2) inhibitors: replacement of the sulfonamide pharmacophore by a sulfonylazide bioisostere. *Bioorg. Med. Chem.* **2003**, *11*(23), 5273–5280.

[8] Chen, Y.; Gibson, J. A convenient synthetic route to sulfonimidamides from sulfonamides. *RSC Adv.* **2015**, *5*(6), 4171–4174.

[9] Davies, T. Q.; Tilby, M. J.; Ren, J.; Parker, N. A.; Skolc, D.; Hall, A.; Duarte, F.; Willis, M. C. Harnessing Sulfinyl Nitrenes: A Unified One-Pot Synthesis of Sulfoximines and Sulfonimidamides. *J. Am. Chem. Soc.* **2020**, *142*(36), 15445–15453.

[10] Davies, T. Q.; Hall, A.; Willis, M. C. One-Pot, Three-Component Sulfonimidamide Synthesis Exploiting the Sulfinylamine Reagent *N*-Sulfinyltritylamine, TrNSO. *Angew. Chem. Int. Ed.* **2017**, *56*(47), 14937–14941.

[11] Rabal, O.; Sanchez-Arias, J. A.; Cuadrado-Tejedor, M.; de Miguel, I.; Perez-Gonzalez, M.; Garcia-Barroso, C.; Ugarte, A.; Estella-Hermoso de Mendoza, A.; Saez, E.; Espelosin, M.; Ursua, S.; Tan, H.; Wu, W.; Xu, M.; Garcia-Osta, A.; Oyarzabal, J. Design, Synthesis, and Biological Evaluation of First-in-Class Dual Acting Histone Deacetylases (HDACs) and Phosphodiesterase 5 (PDE5) Inhibitors for the Treatment of Alzheimer's Disease. *J. Med. Chem.* **2016**, *59*(19), 8967-9004.

[12] Bremerich, M.; Conrads, C. M.; Langletz, T.; Bolm, C. Additions to *N*-Sulfinylamines as an Approach for the Metal-free Synthesis of Sulfonimidamides: *O*-Benzotriazolyl Sulfonimidates as Activated Intermediates. *Angew. Chem. Int. Ed.* **2019**, *58*(52), 19014-19020.

5. NMR spectra

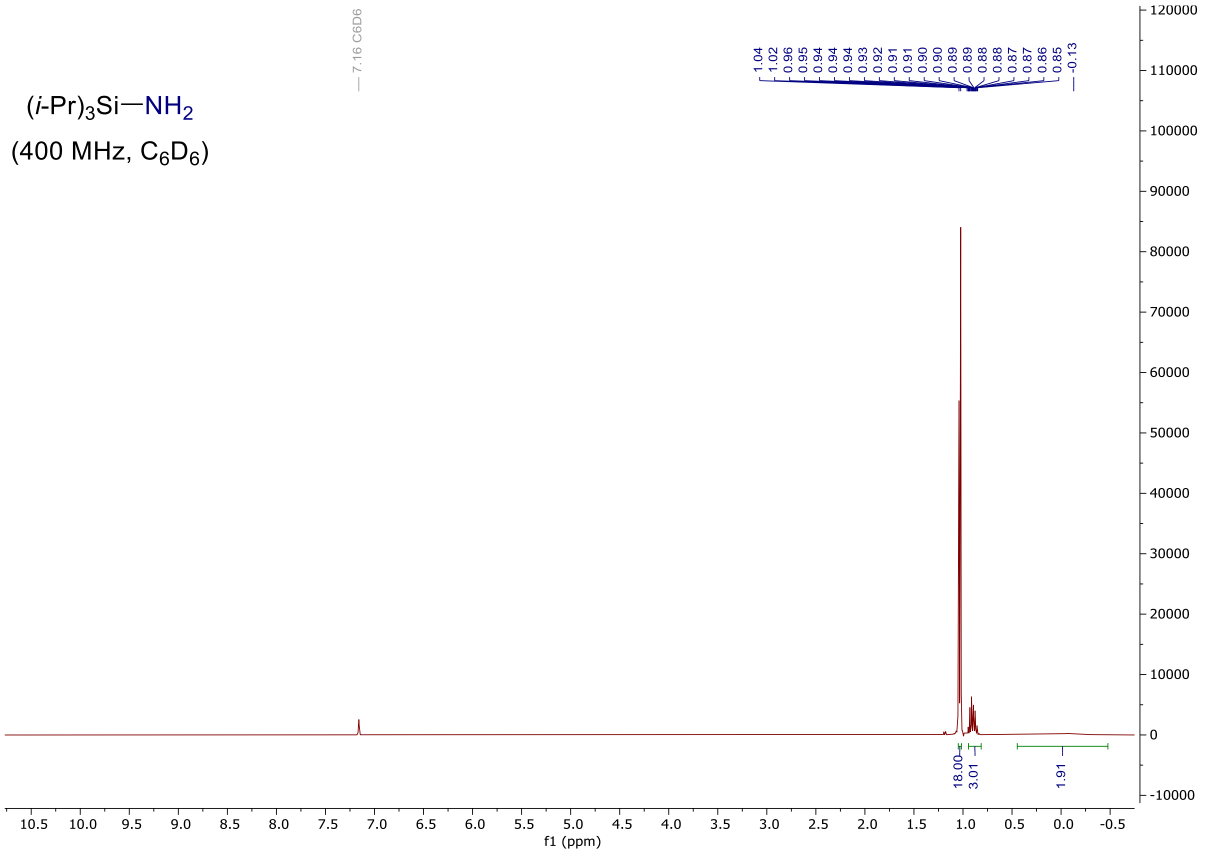

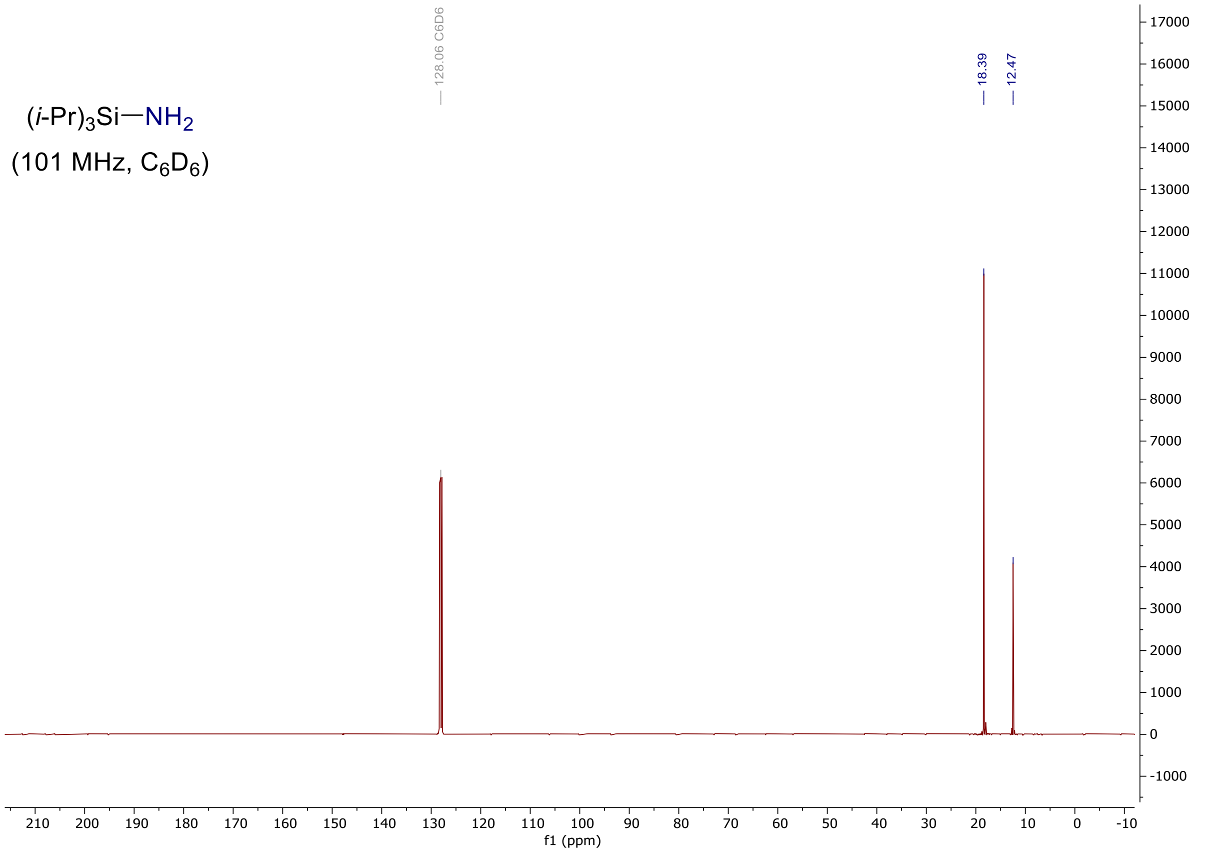

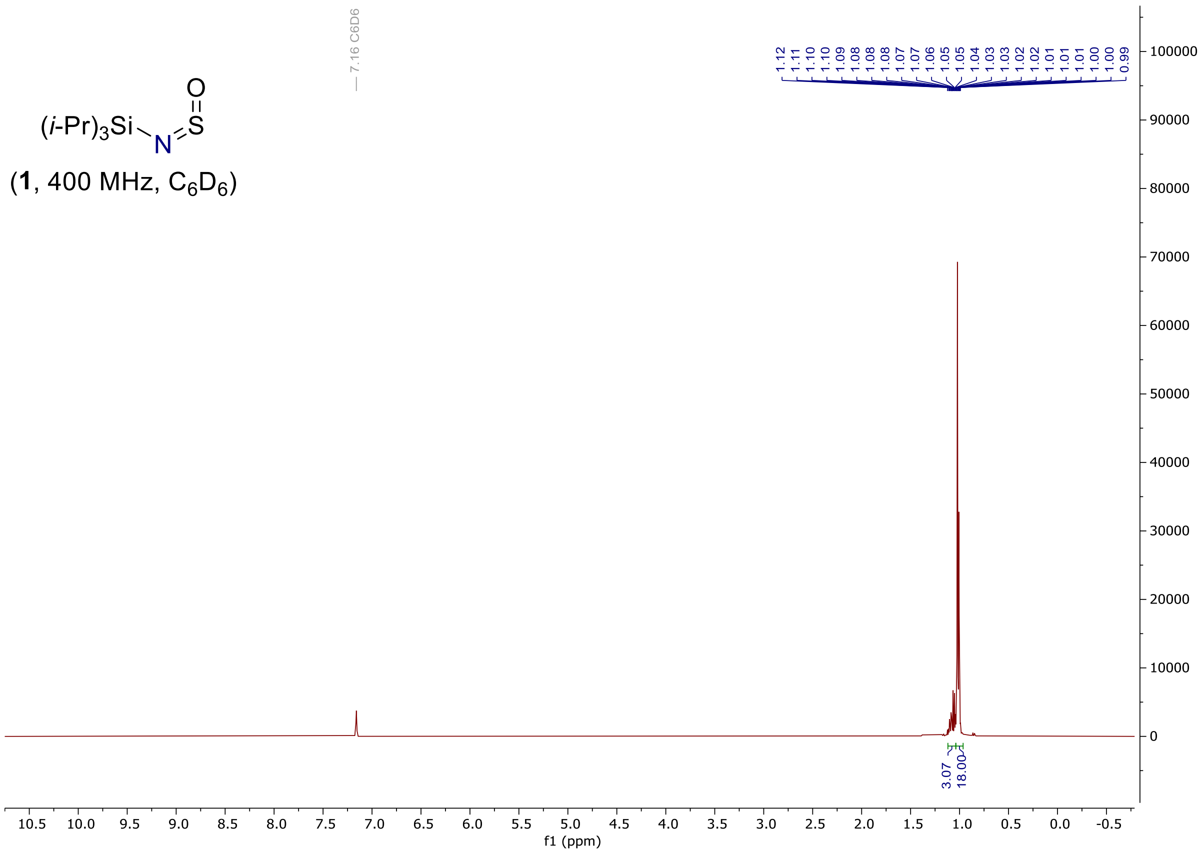
(i-Pr)3Si N S O
(1, 400 MHz, C6D6)
7.16 C6D6
1.12 1.11 1.10 1.10 1.09 1.08 1.08 1.08 1.07 1.07 1.06 1.05 1.05 1.04 1.03 1.03 1.02 1.02 1.01 1.01 1.00 1.00 0.99
3.07
18.00
f1 (ppm)

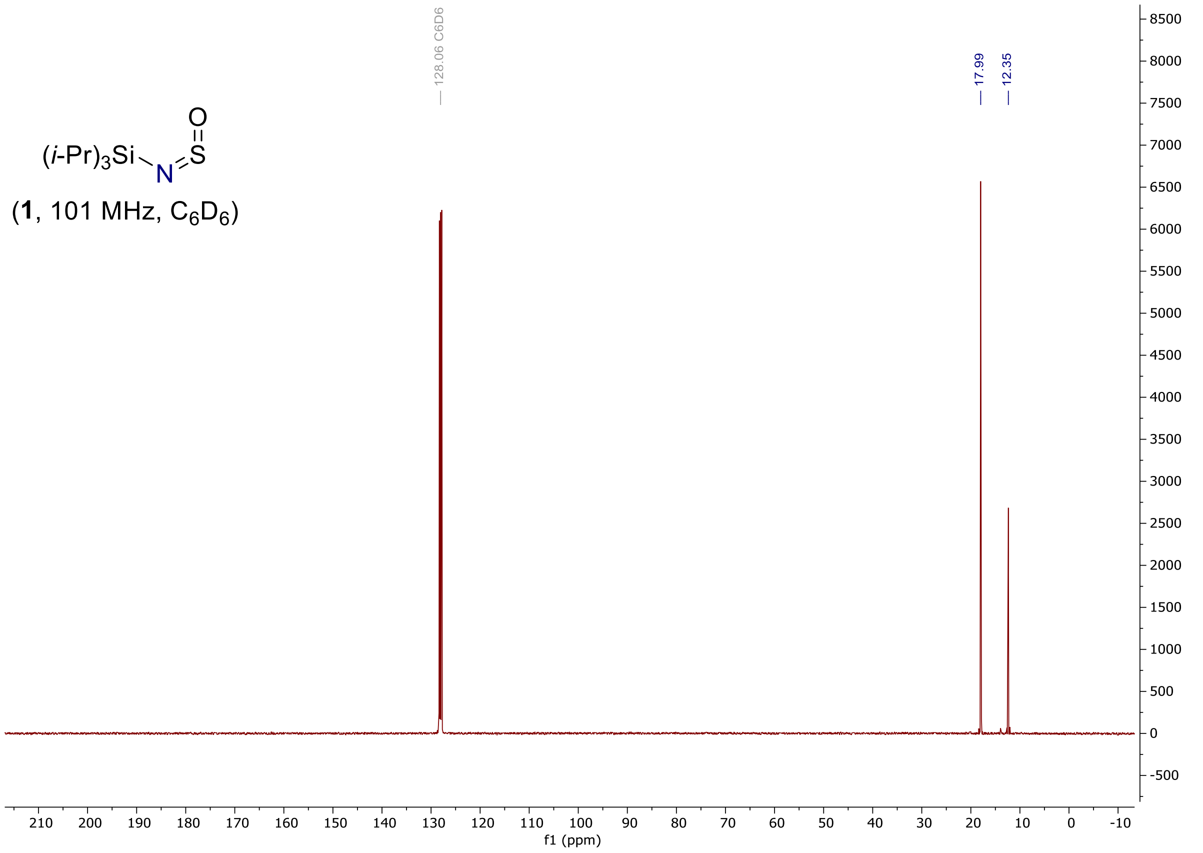
(i-Pr)3Si N S O
(1, 101 MHz, C6D6)
128.06 C6D6
17.99
12.35
f1 (ppm)

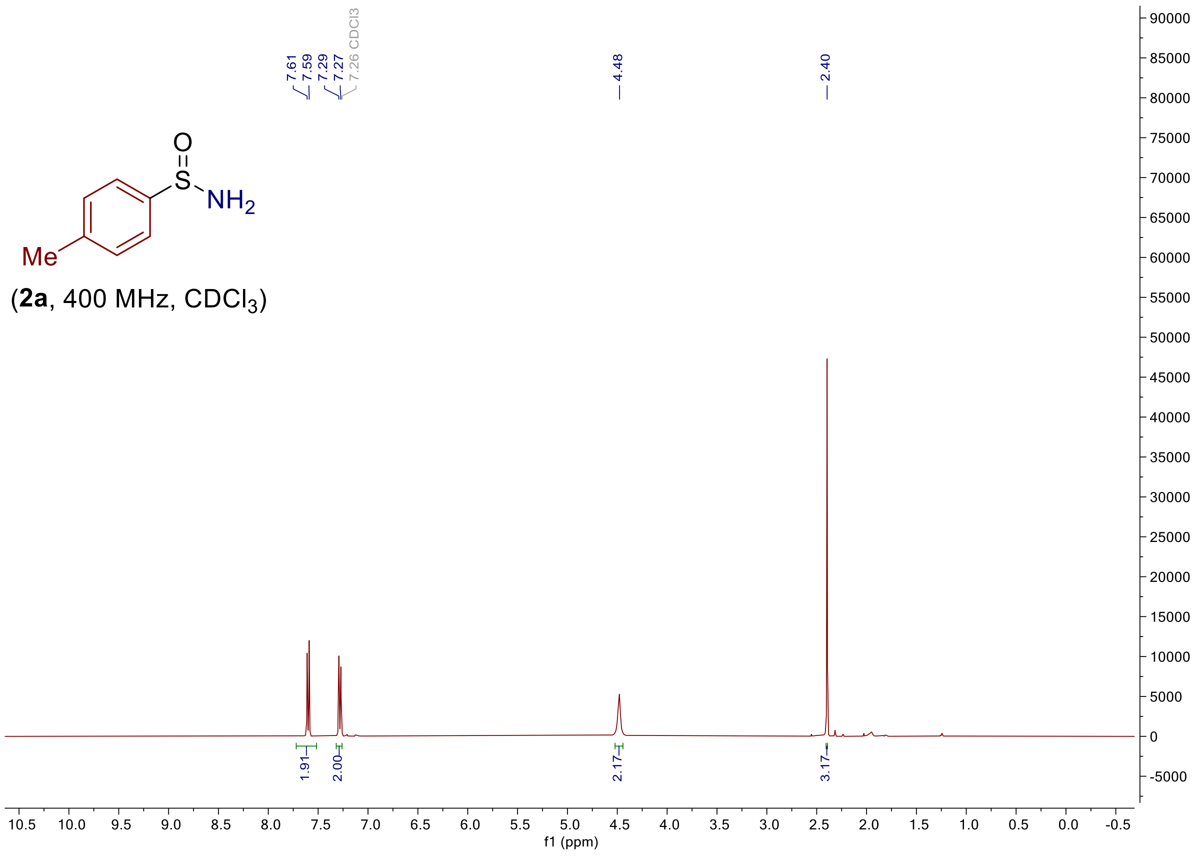

(**2a**, 400 MHz, $CDCl_3$)

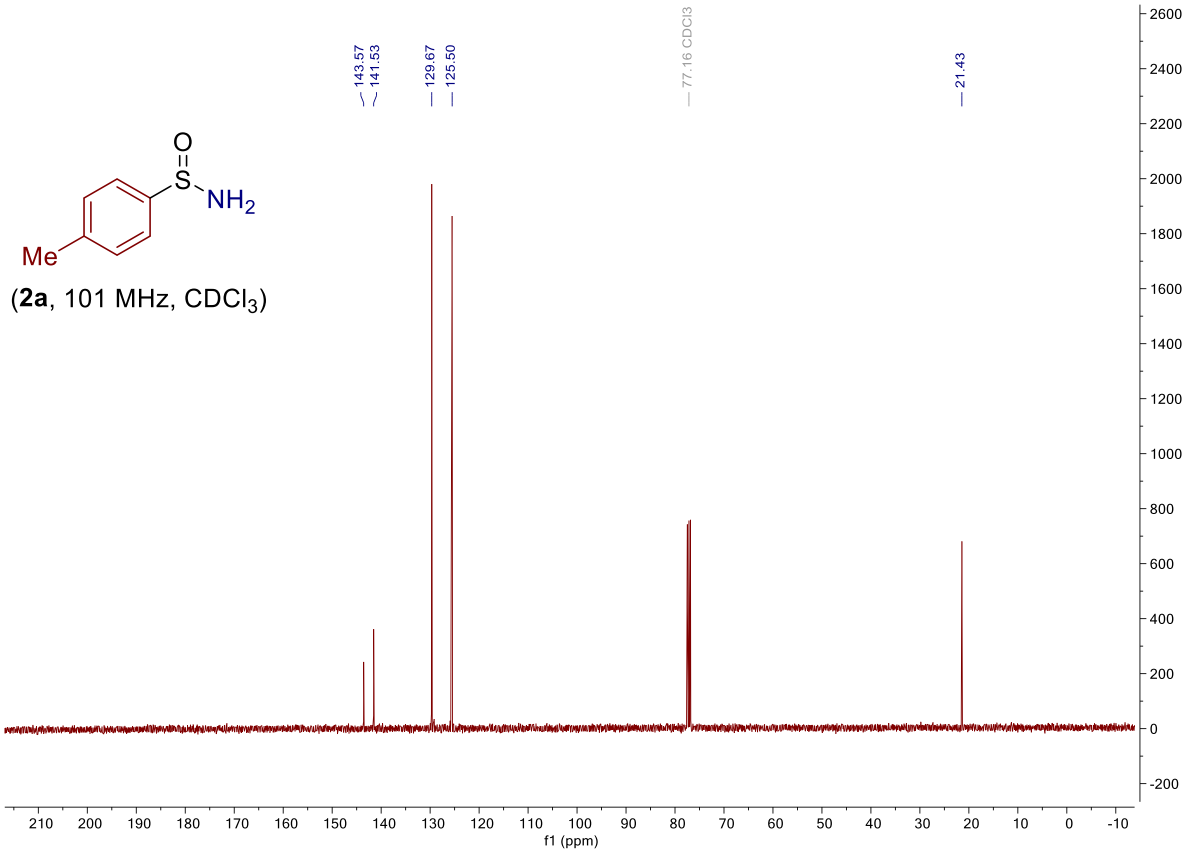

(**2a**, 101 MHz, $CDCl_3$)

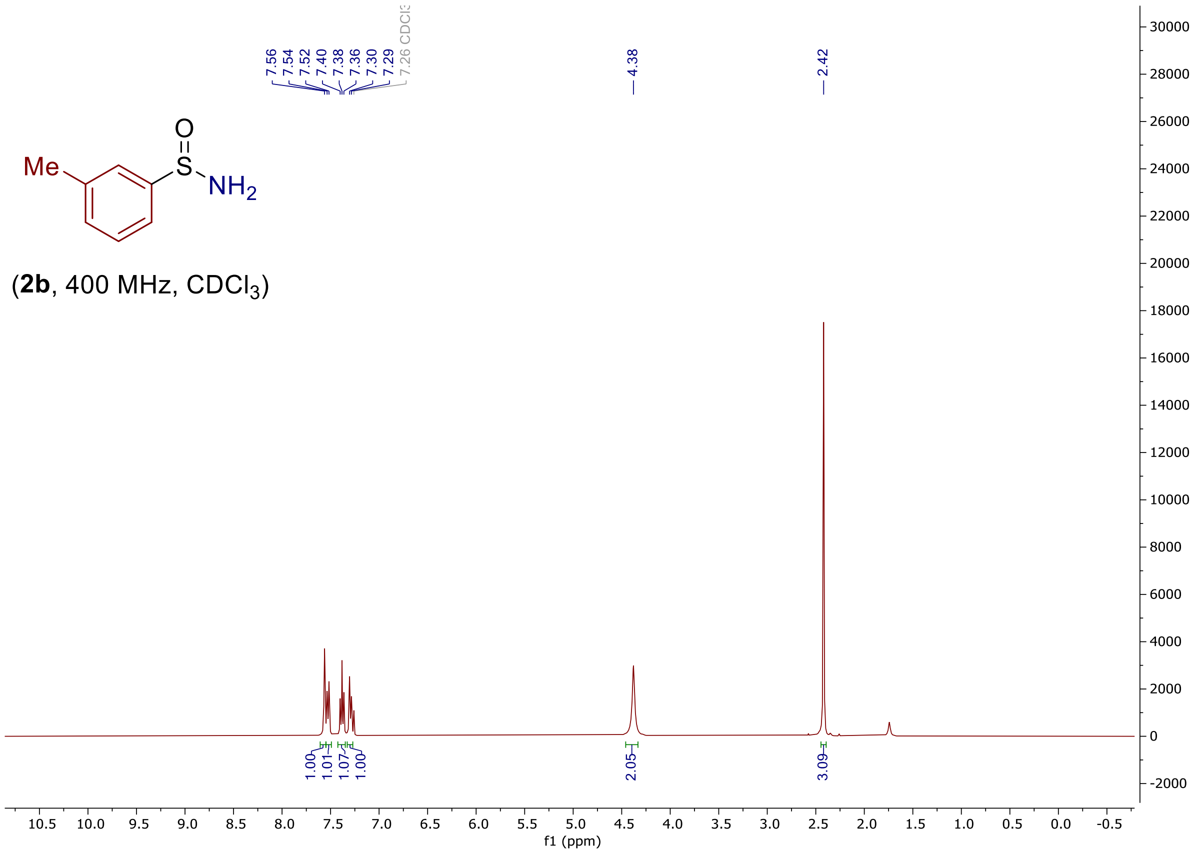
7.56
7.54
7.52
7.40
7.38
7.36
7.30
7.29
7.26 CDCl3
4.38
2.42
Me
O
S
NH2
(2b, 400 MHz, CDCl3)
1.00
1.01
1.07
1.00
2.05
3.09
f1 (ppm)

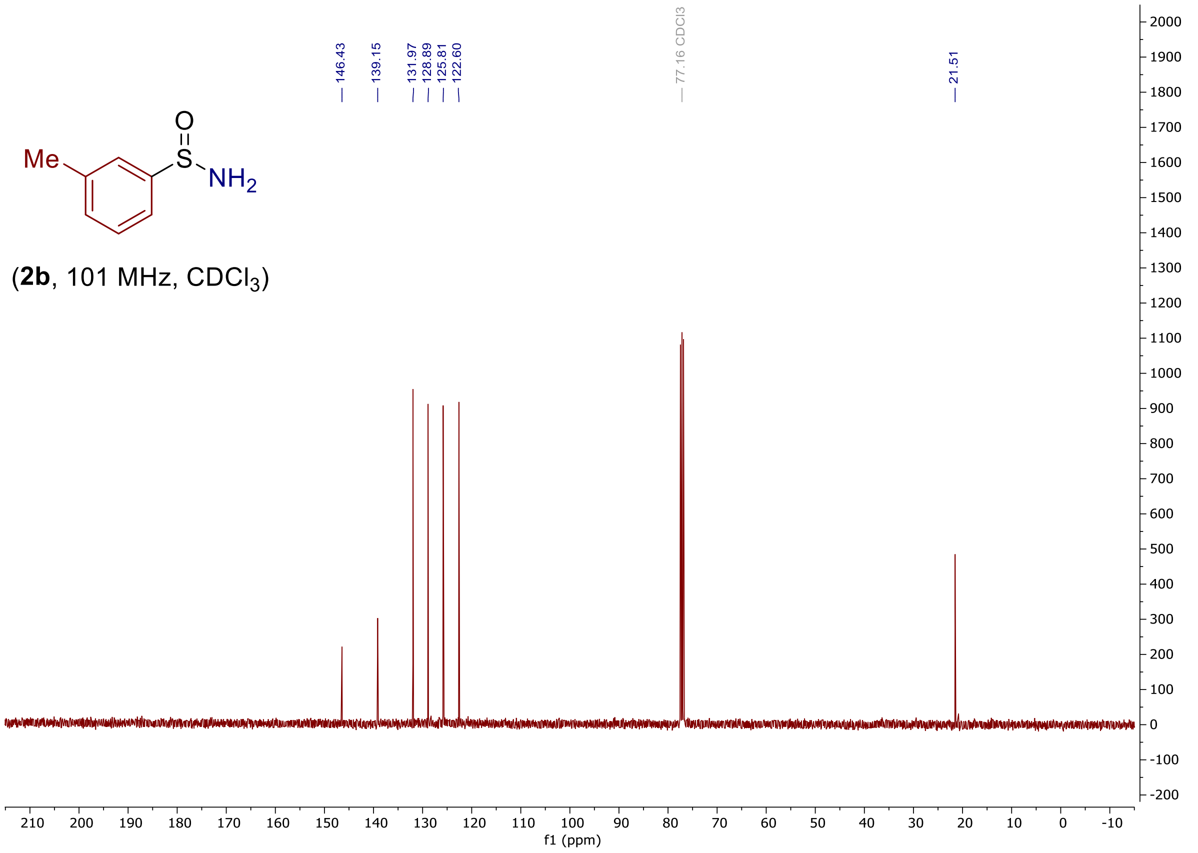
146.43
139.15
131.97
128.89
125.81
122.60
77.16 CDCl3
21.51
Me
O
S
NH2
(2b, 101 MHz, CDCl3)
f1 (ppm)

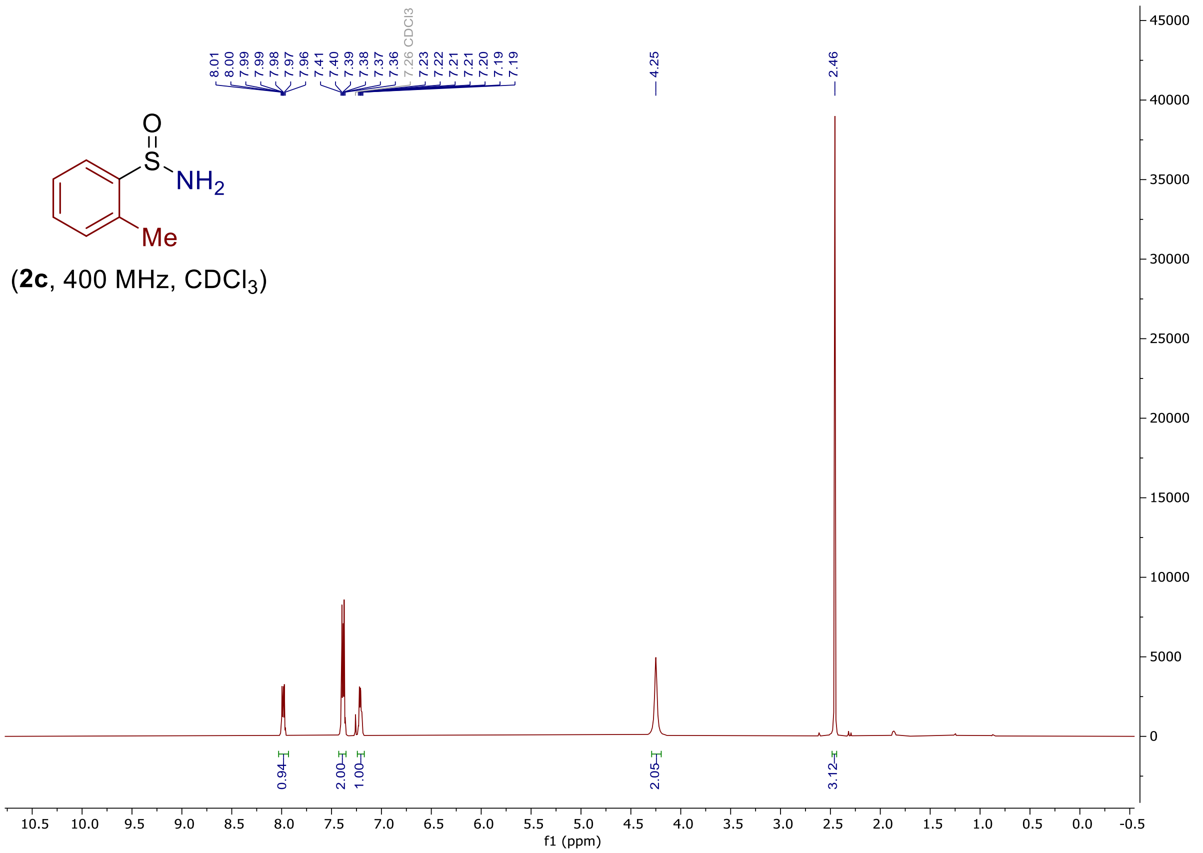

(**2c**, 400 MHz, $CDCl_3$)

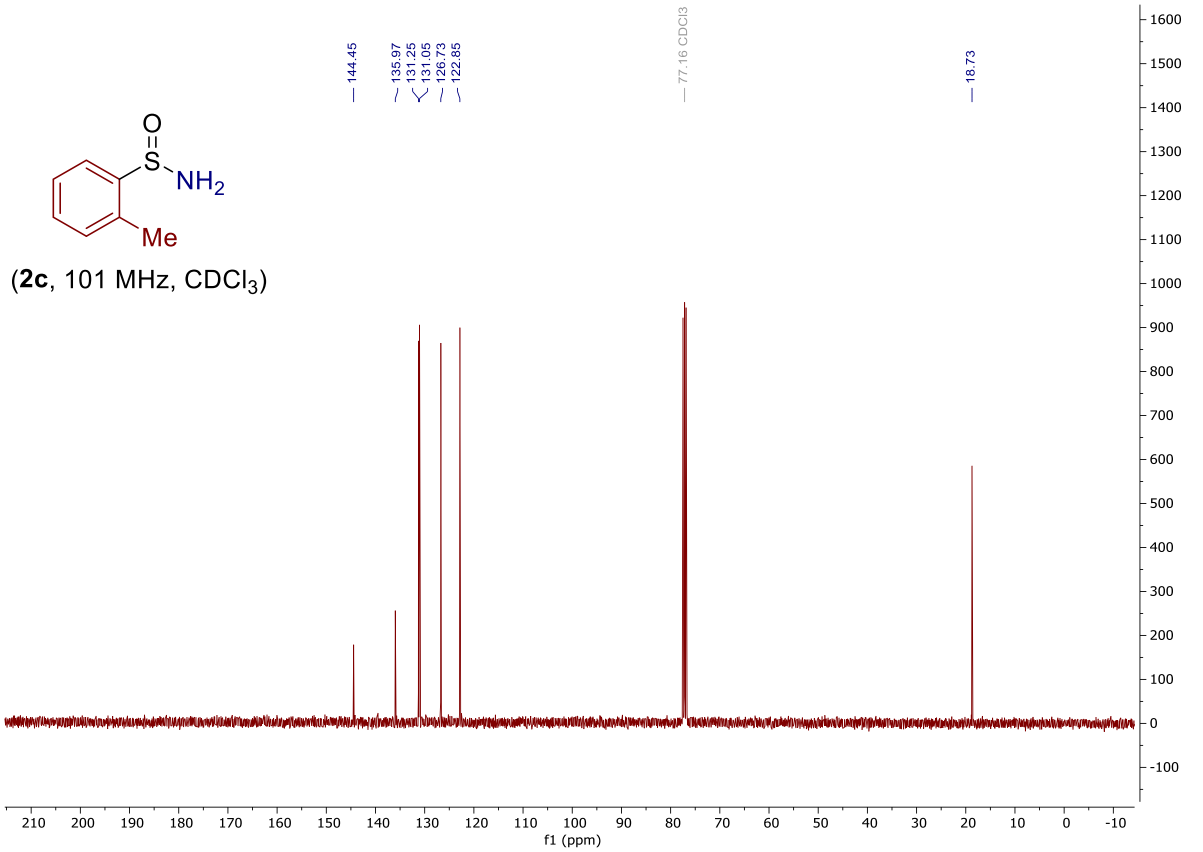

(**2c**, 101 MHz, $CDCl_3$)

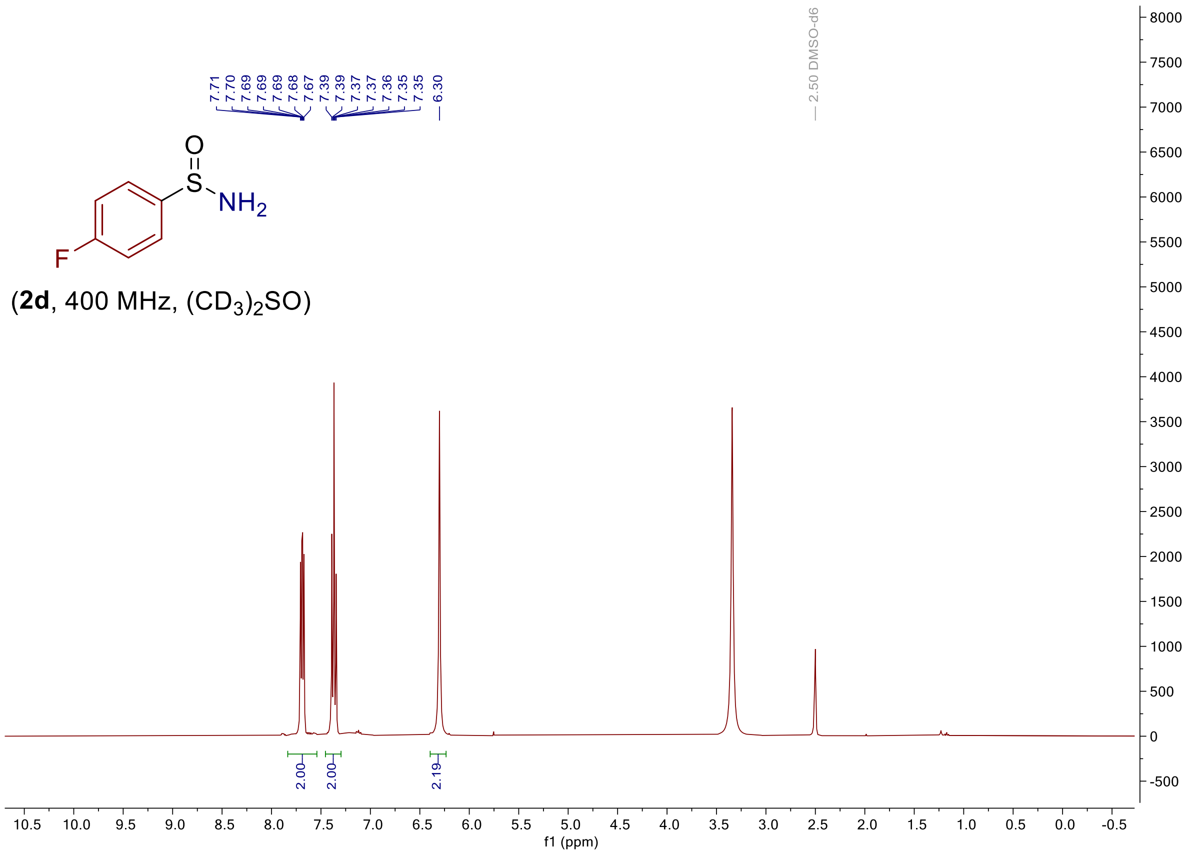

(**2d**, 400 MHz, $(CD_3)_2SO$)

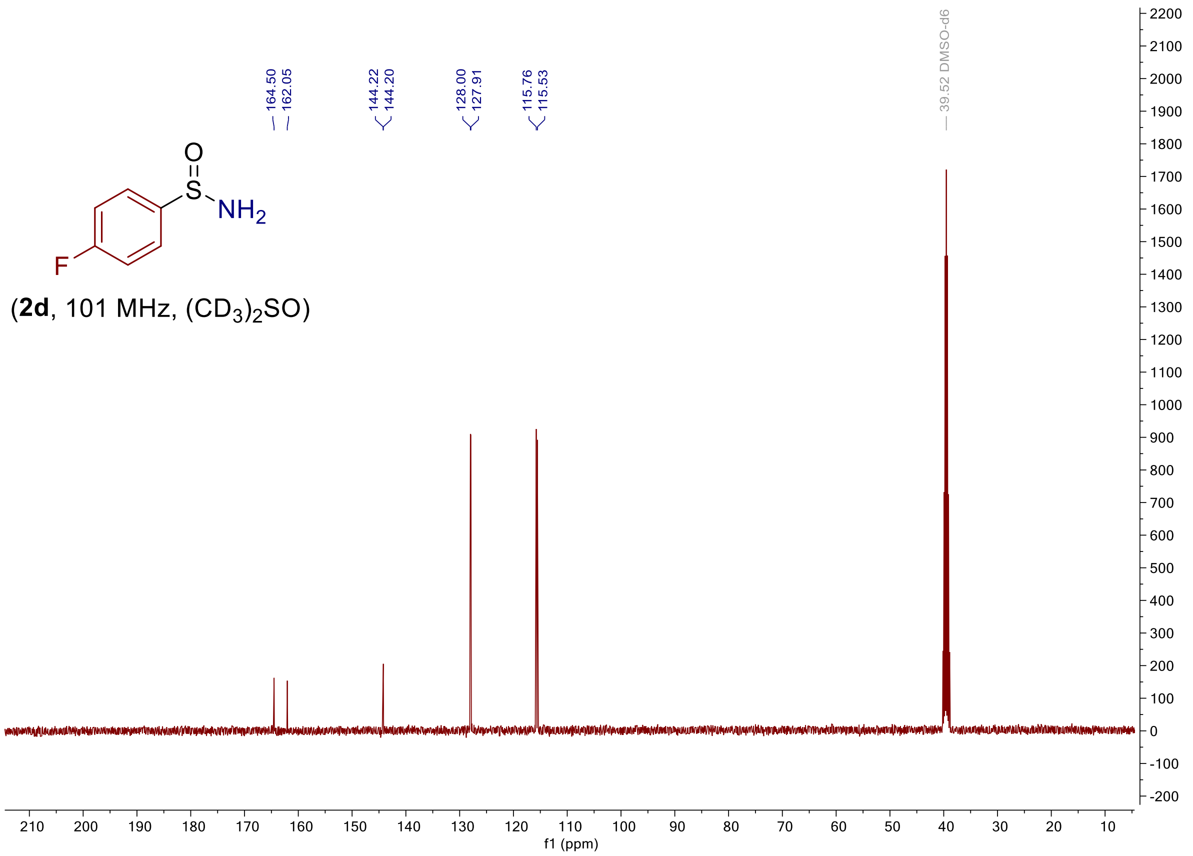

(**2d**, 101 MHz, $(CD_3)_2SO$)

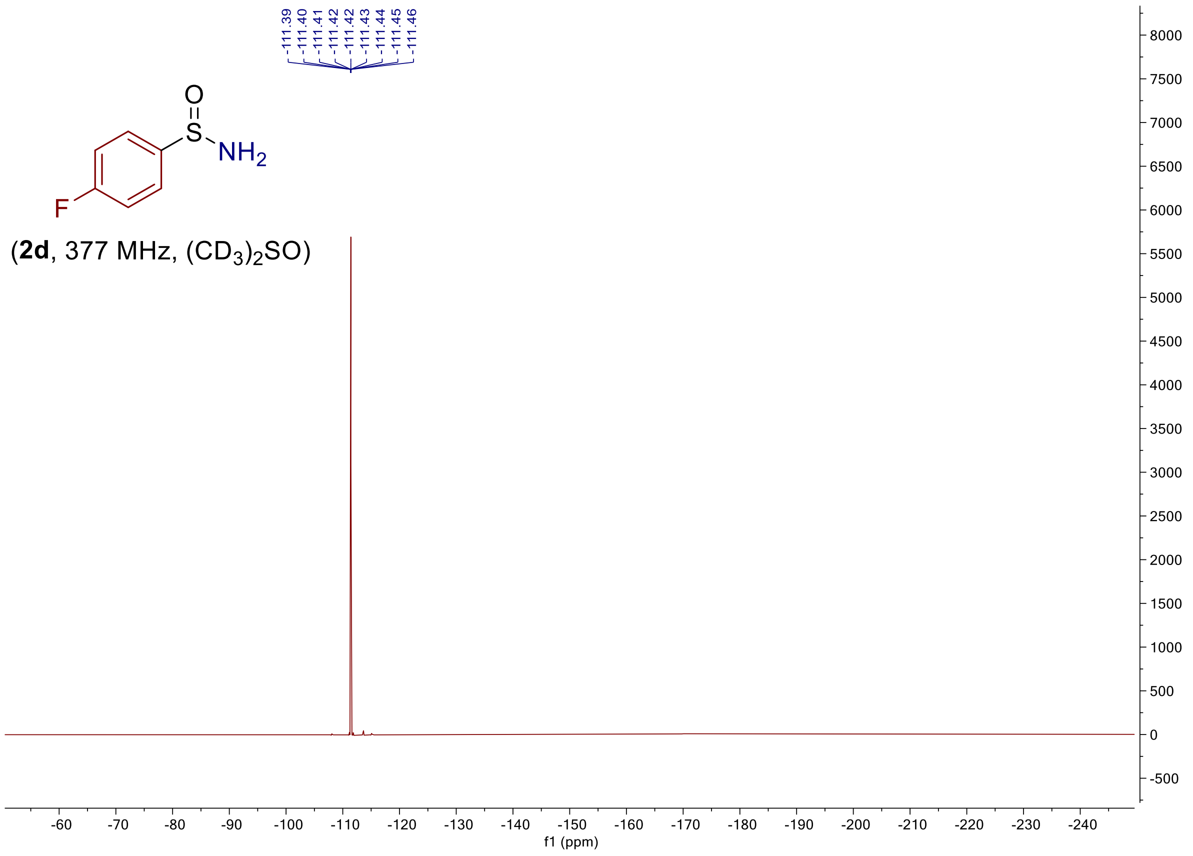
-111.39
-111.40
-111.41
-111.42
-111.42
-111.43
-111.44
-111.45
-111.46
O
S
NH2
F
(2d, 377 MHz, (CD3)2SO)
f1 (ppm)

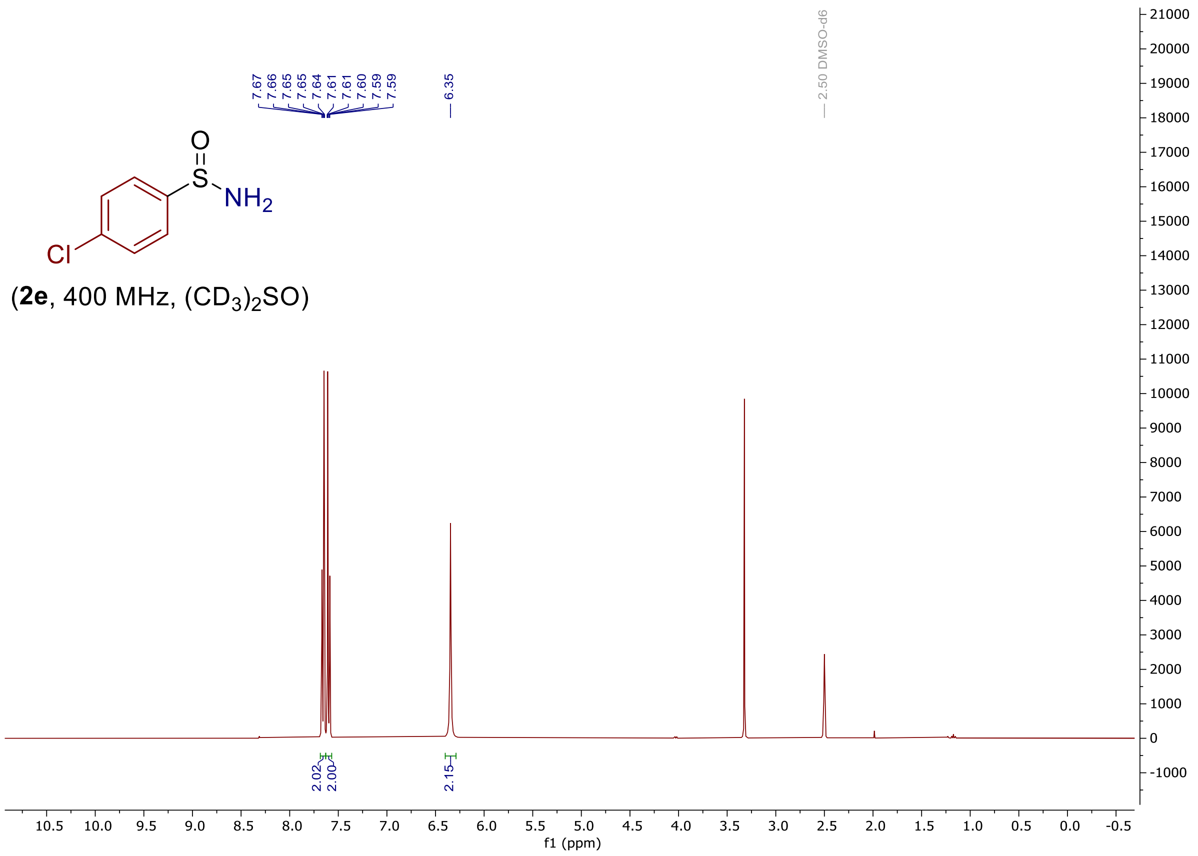
7.67
7.66
7.65
7.65
7.64
7.61
7.61
7.60
7.59
7.59
6.35
2.50 DMSO-d6
O
S
NH2
Cl
(2e, 400 MHz, (CD3)2SO)
2.02
2.00
2.15
f1 (ppm)

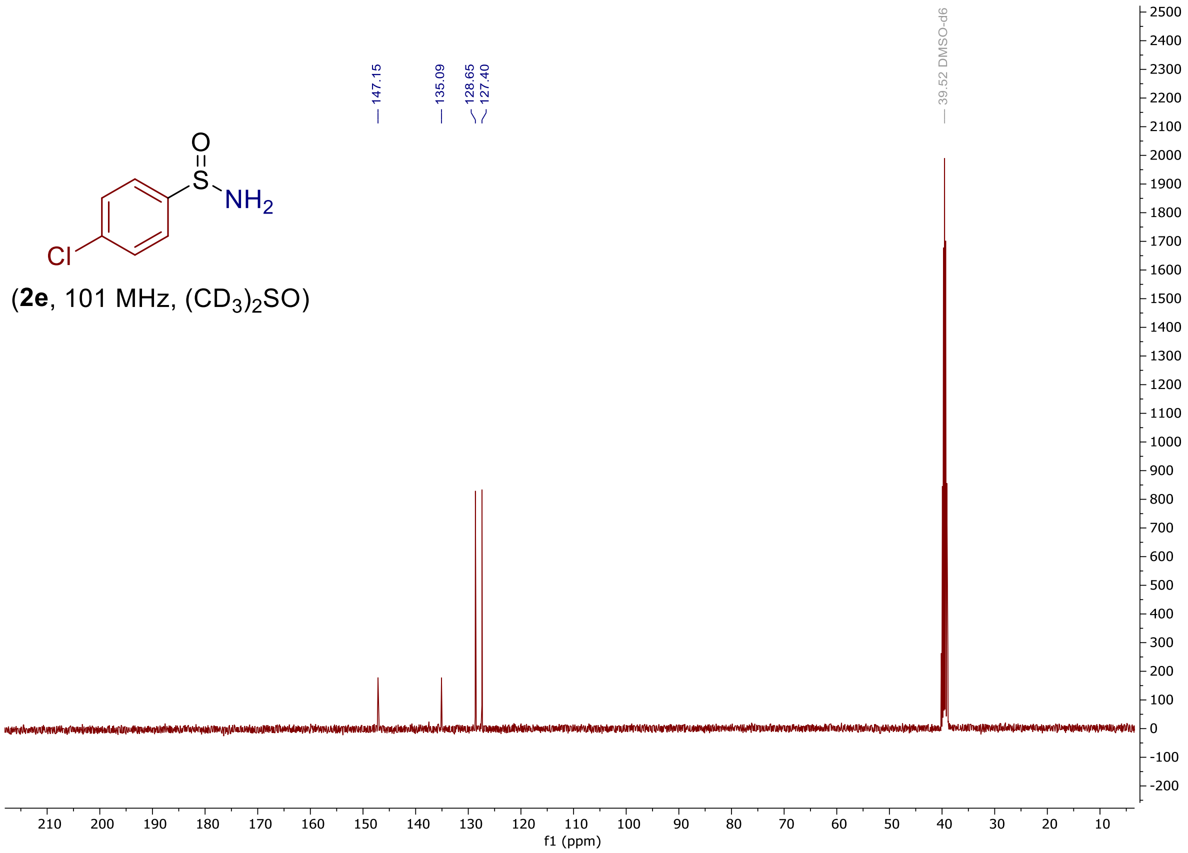

(**2e**, 101 MHz, $(CD_3)_2SO$)

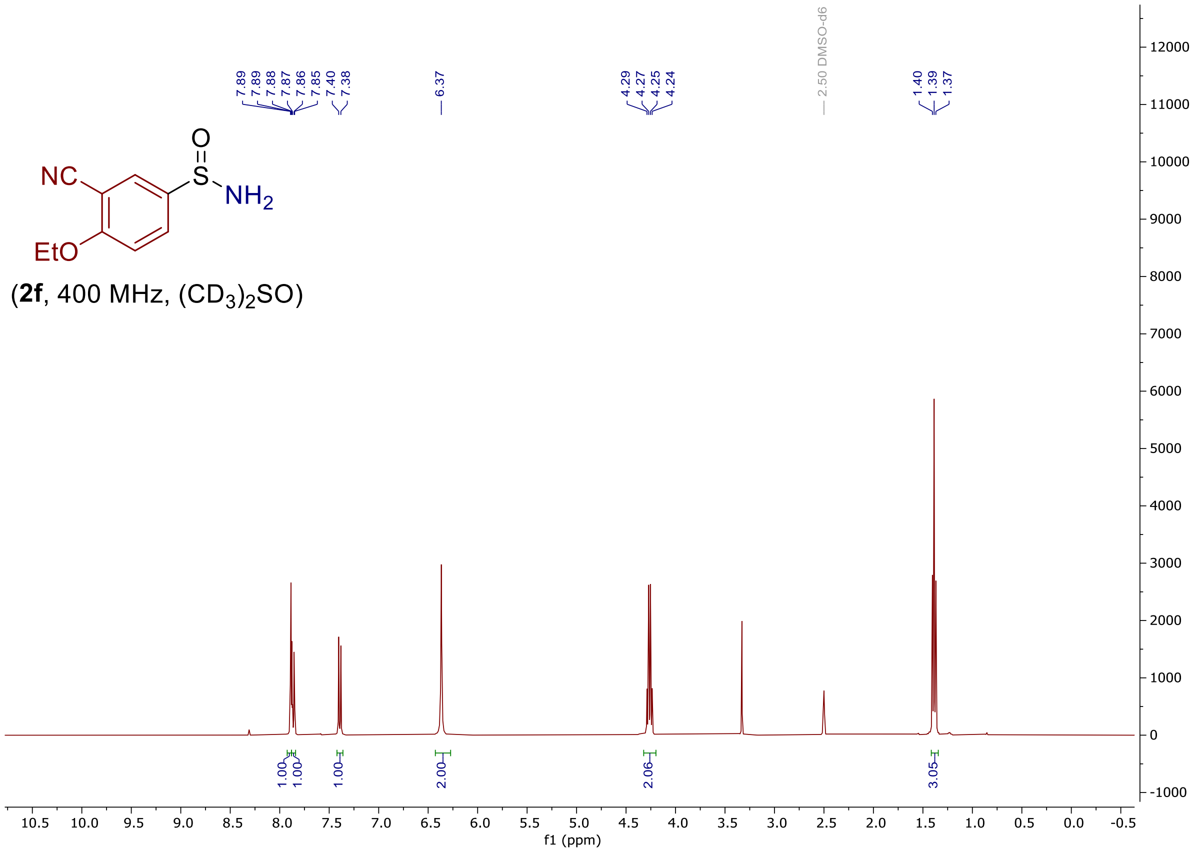

(**2f**, 400 MHz, $(CD_3)_2SO$)

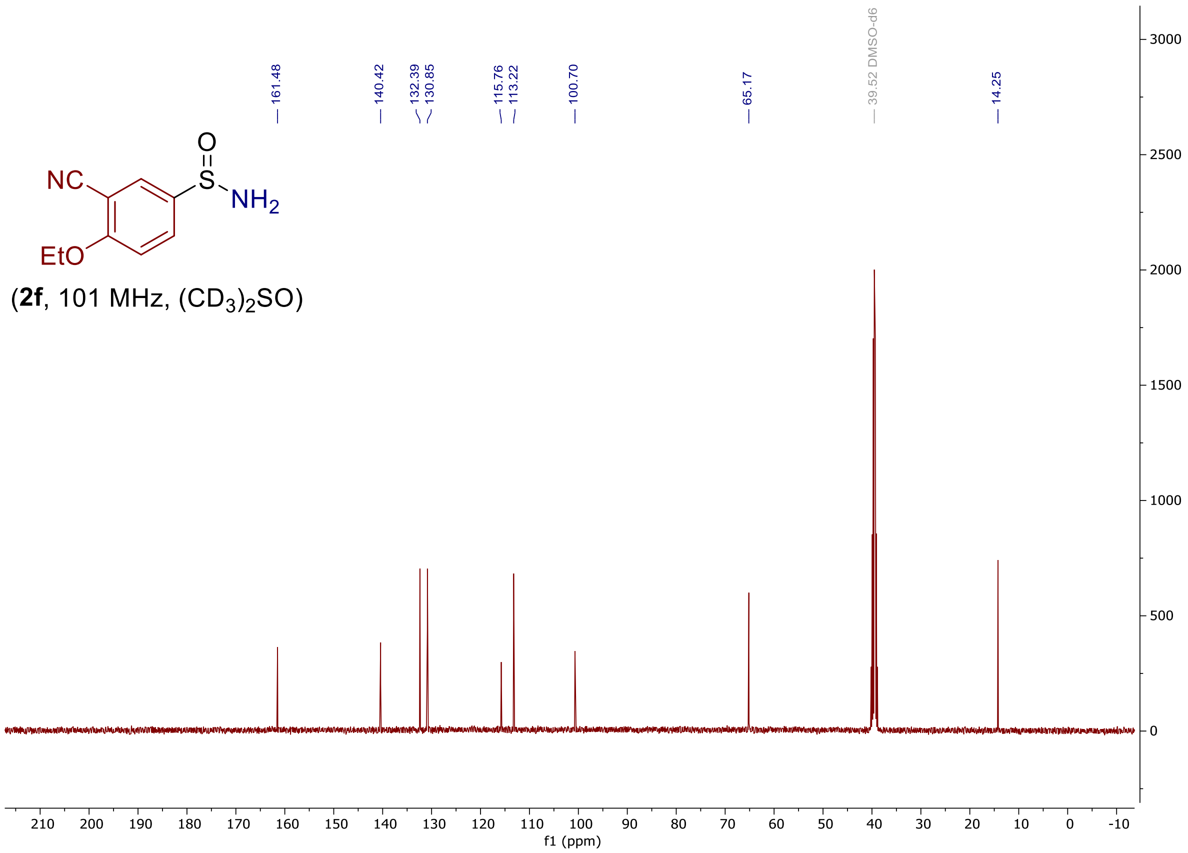

(**2f**, 101 MHz, $(CD_3)_2SO$)

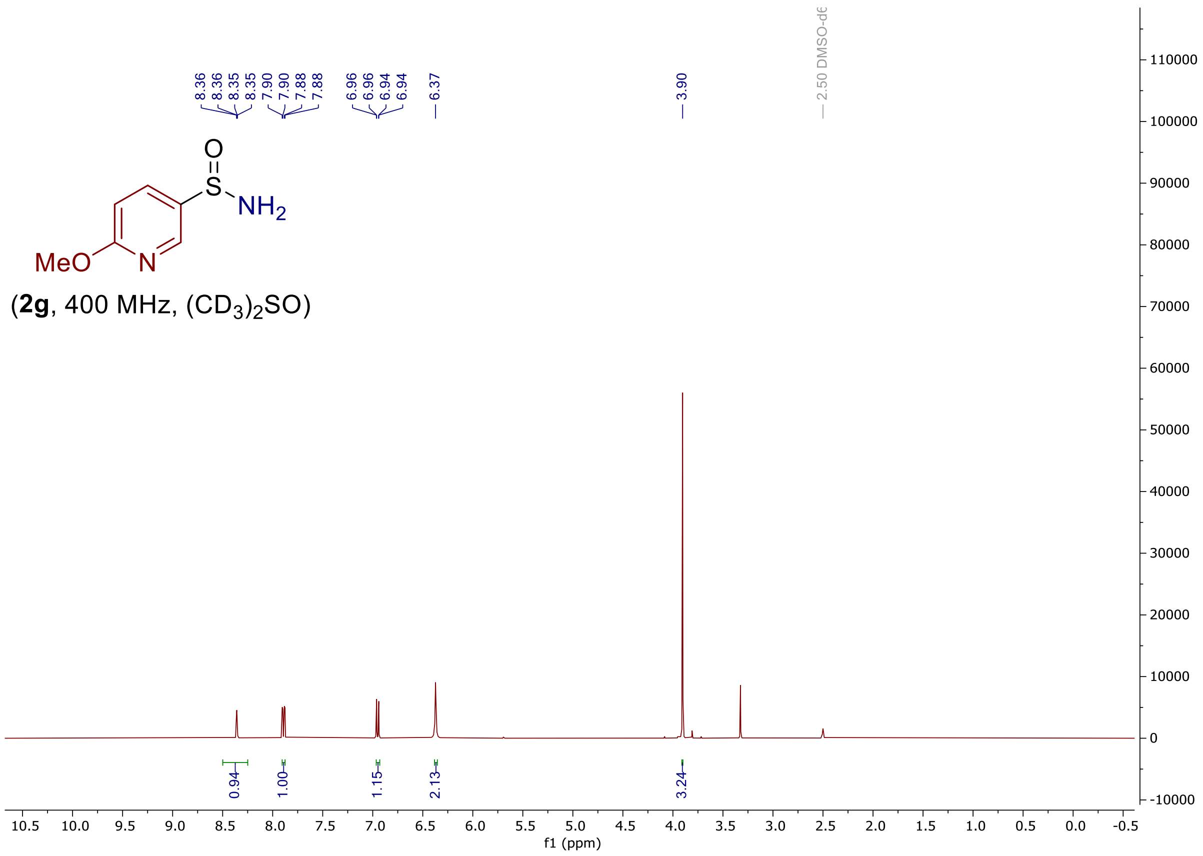

(**2g**, 400 MHz, $(CD_3)_2SO$)

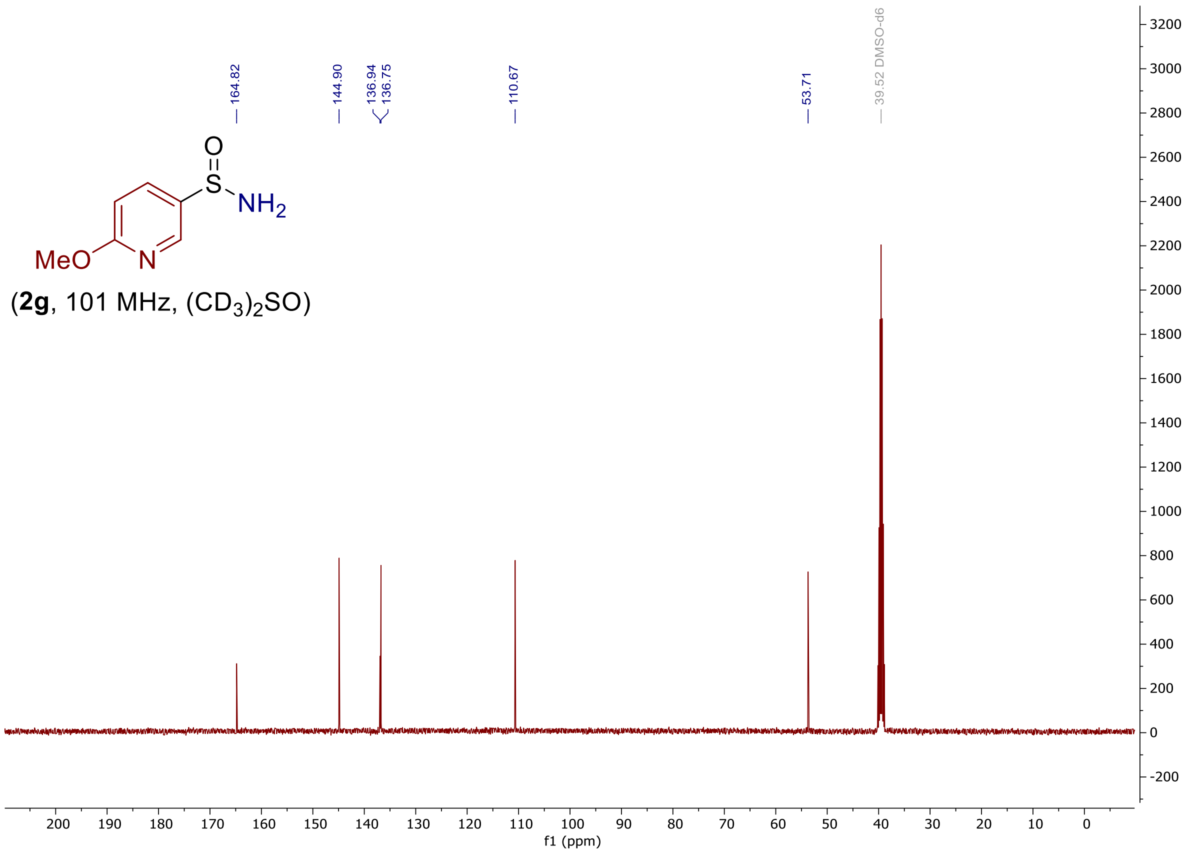
164.82
144.90
136.94
136.75
110.67
53.71
39.52 DMSO-d6
MeO
N
S
O
NH2
(2g, 101 MHz, (CD3)2SO)
f1 (ppm)

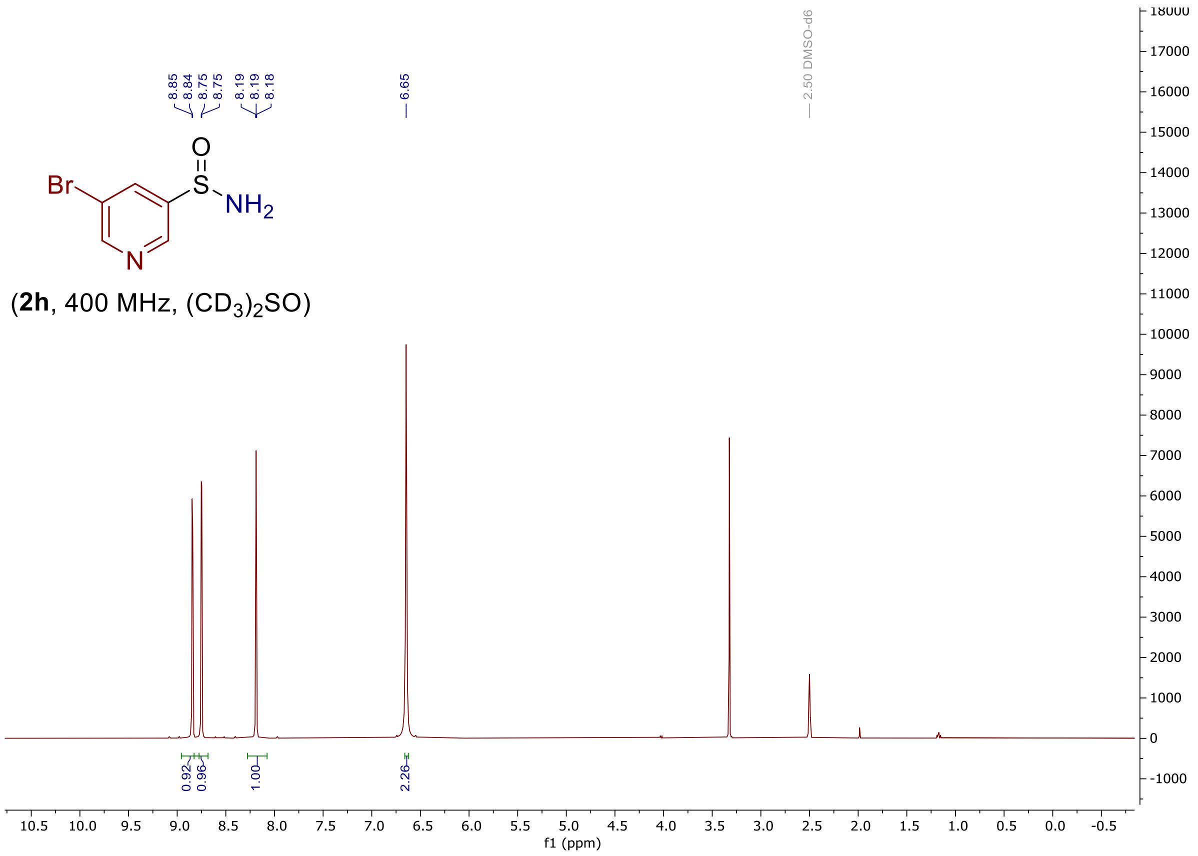
8.85
8.84
8.75
8.75
8.19
8.19
8.18
6.65
2.50 DMSO-d6
Br
N
S
O
NH2
(2h, 400 MHz, (CD3)2SO)
0.92
0.96
1.00
2.26
f1 (ppm)

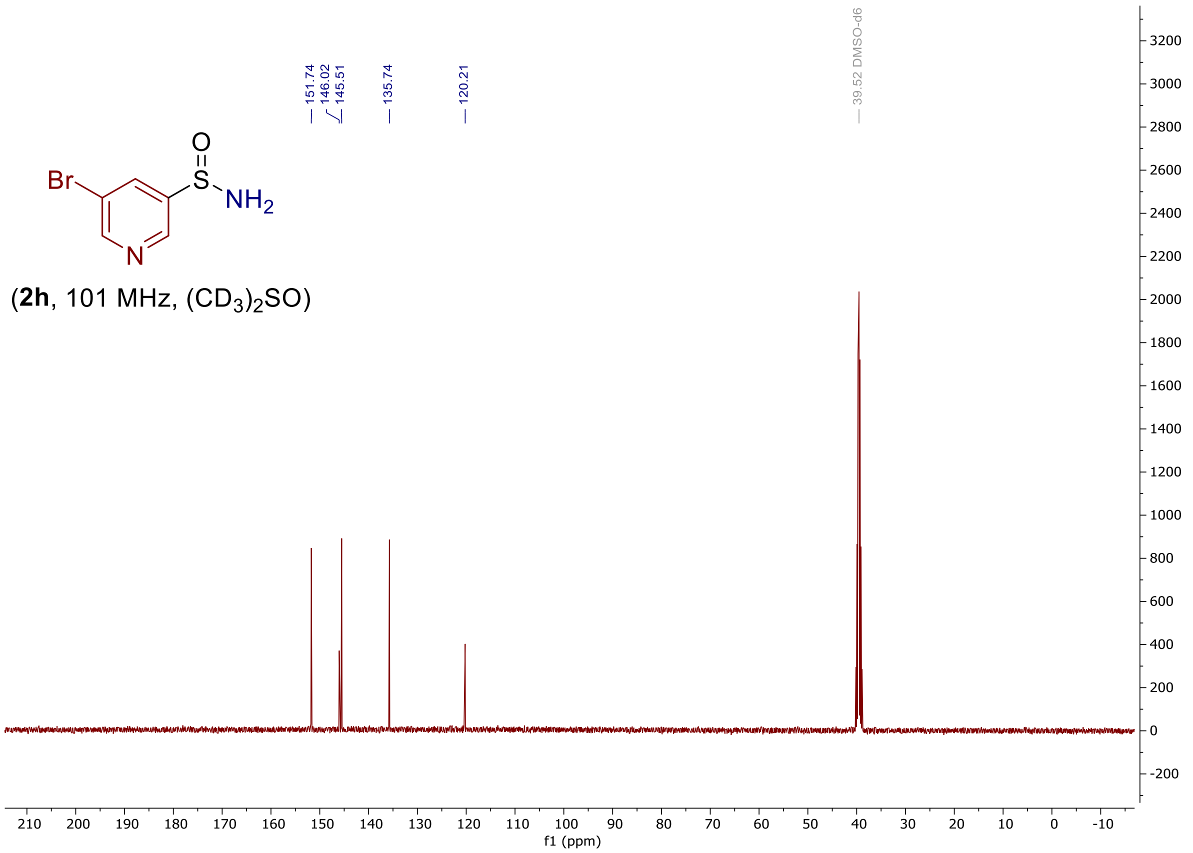
151.74
146.02
145.51
135.74
120.21
39.52 DMSO-d6
(2h, 101 MHz, (CD3)2SO)
f1 (ppm)

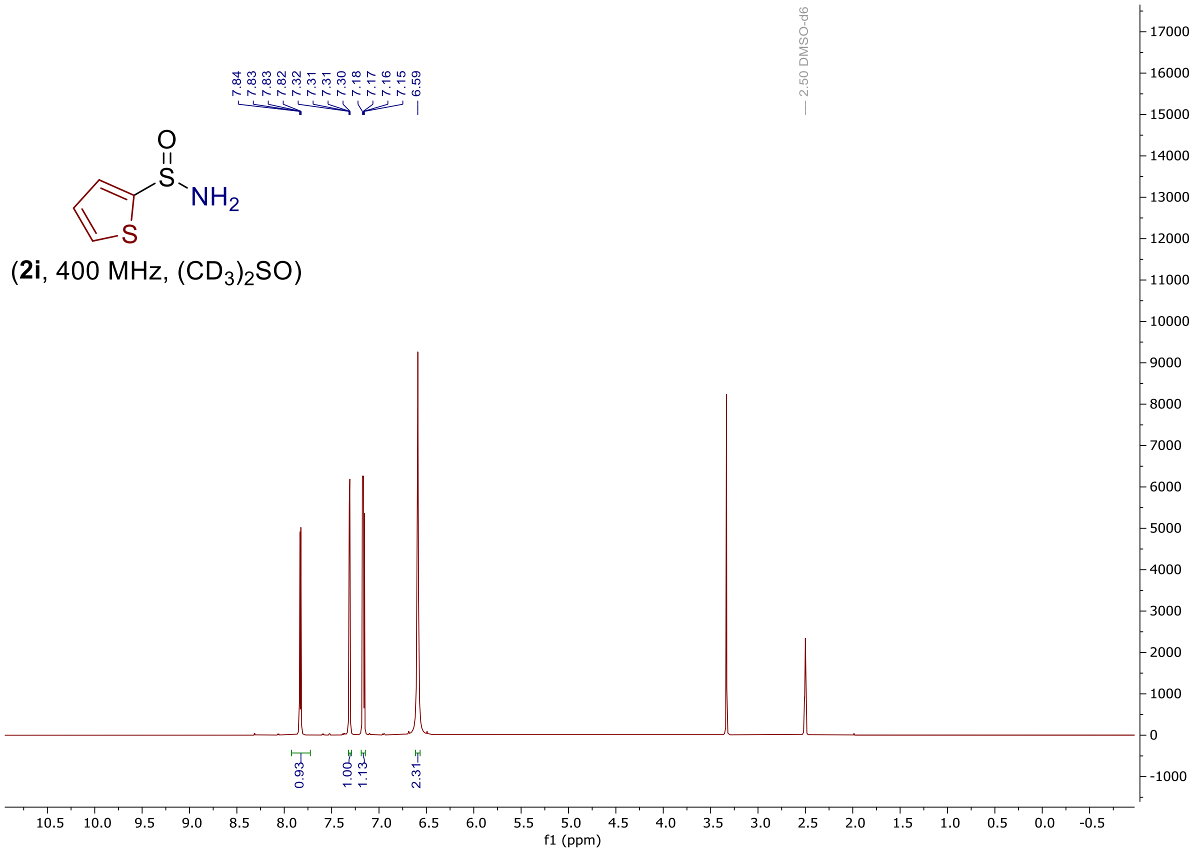
7.84
7.83
7.83
7.82
7.32
7.31
7.31
7.30
7.18
7.17
7.16
7.15
6.59
2.50 DMSO-d6
(2i, 400 MHz, (CD3)2SO)
0.93
1.00
1.13
2.31
f1 (ppm)

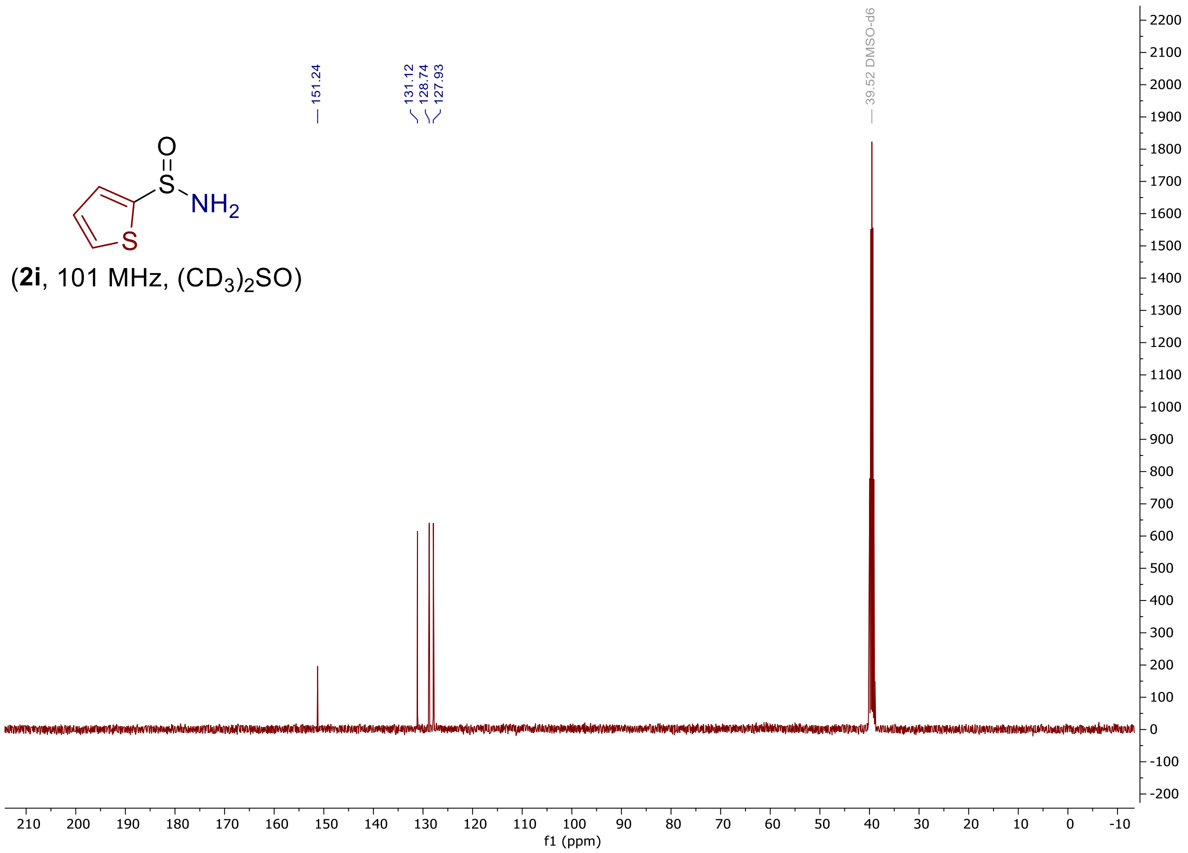

(**2i**, 101 MHz, $(CD_3)_2SO$)

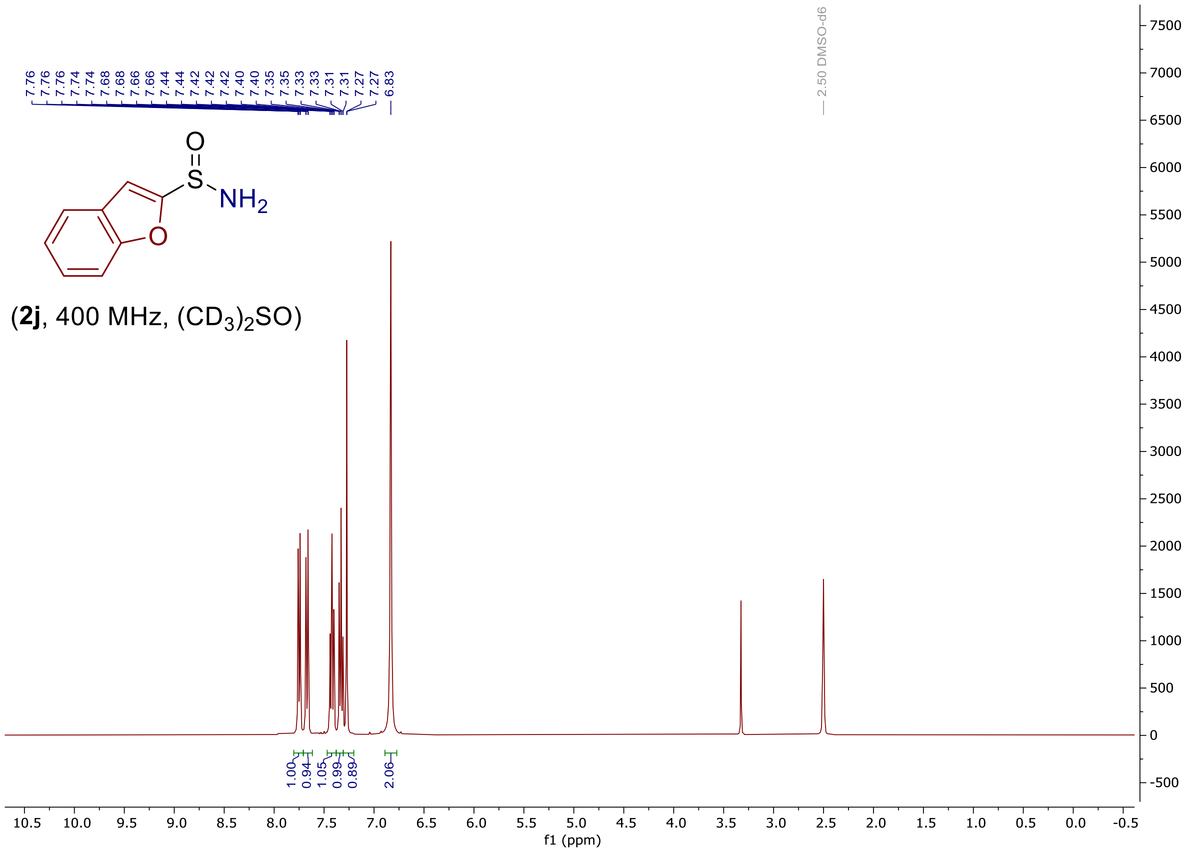

(**2j**, 400 MHz, $(CD_3)_2SO$)

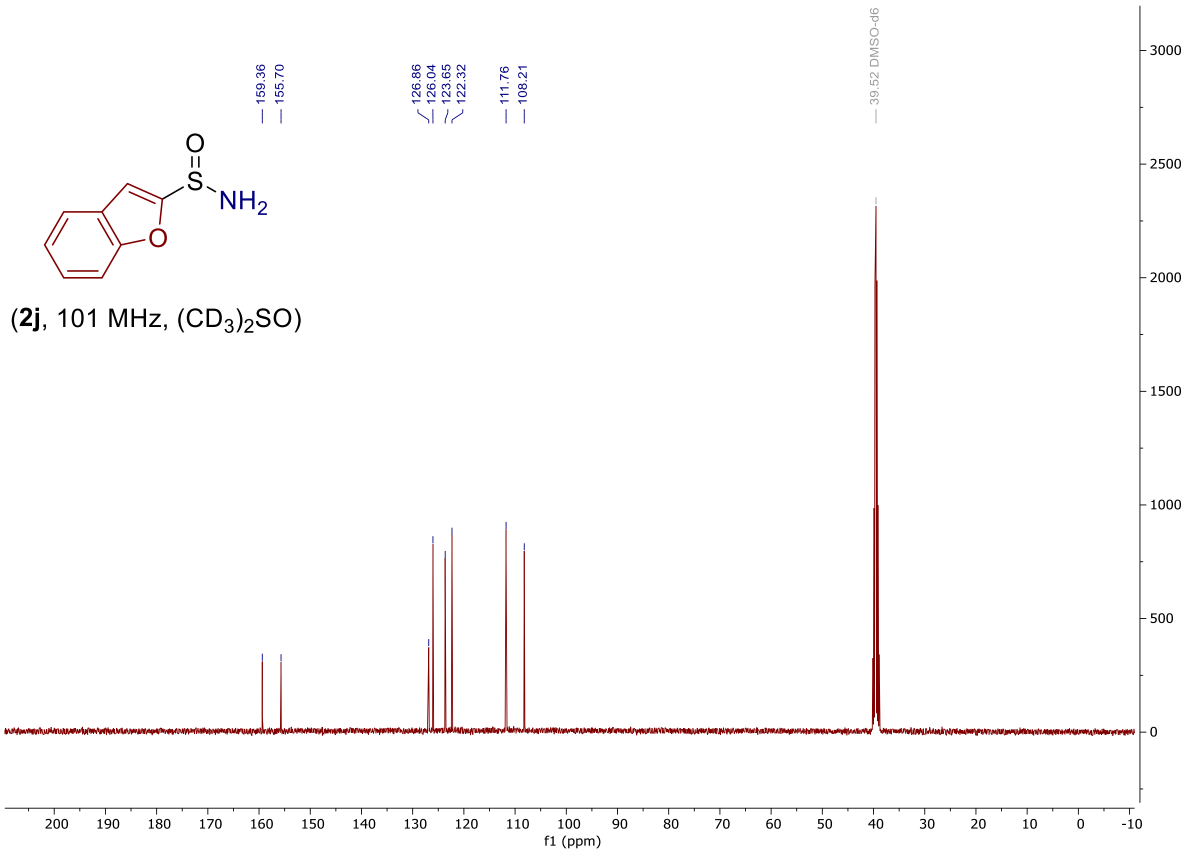

(**2j**, 101 MHz, $(CD_3)_2SO$)

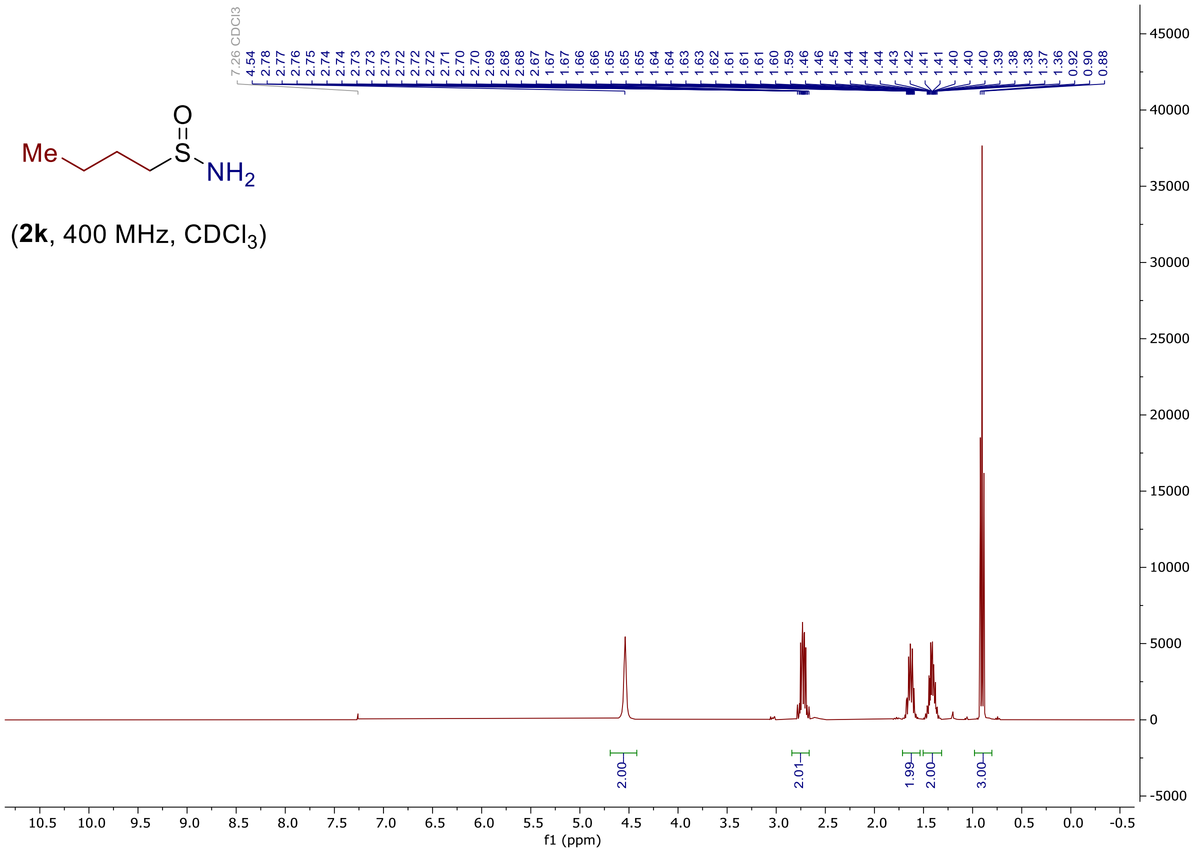

(**2k**, 400 MHz, $CDCl_3$)

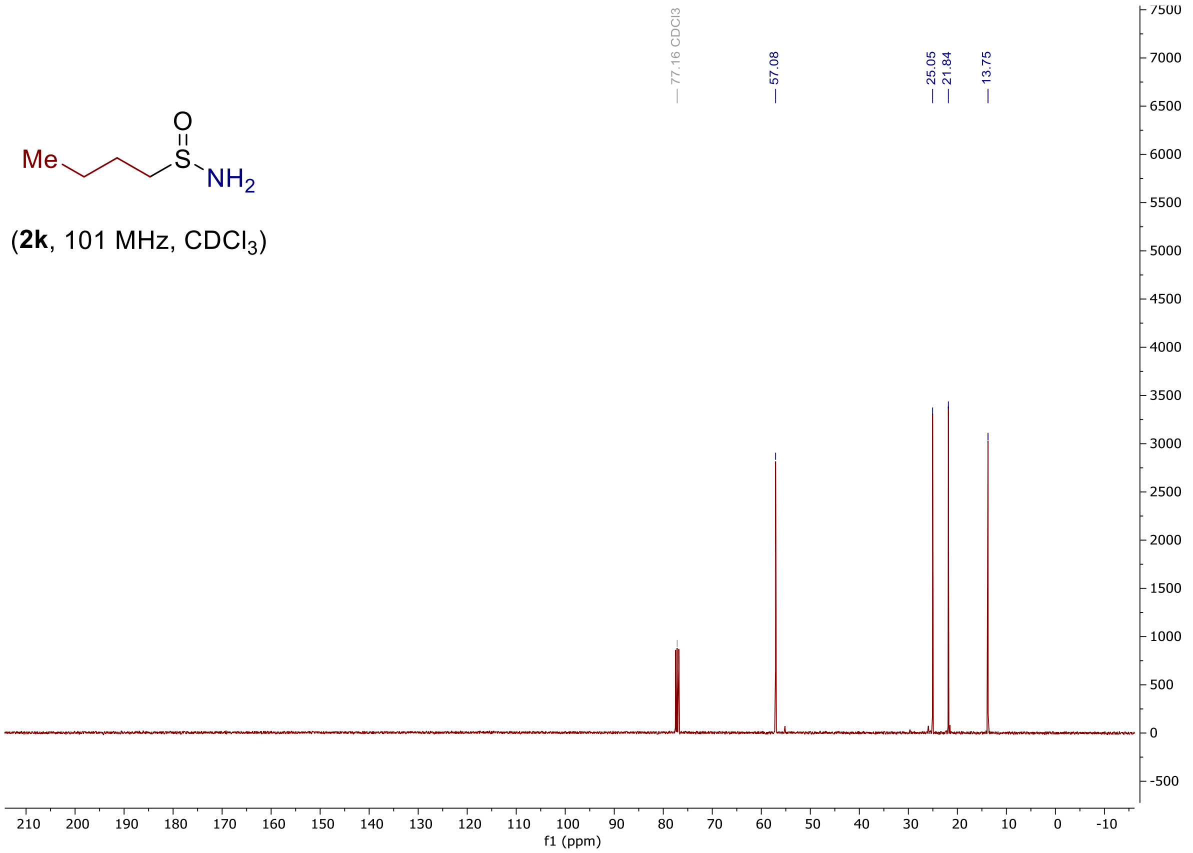

(**2k**, 101 MHz, $CDCl_3$)

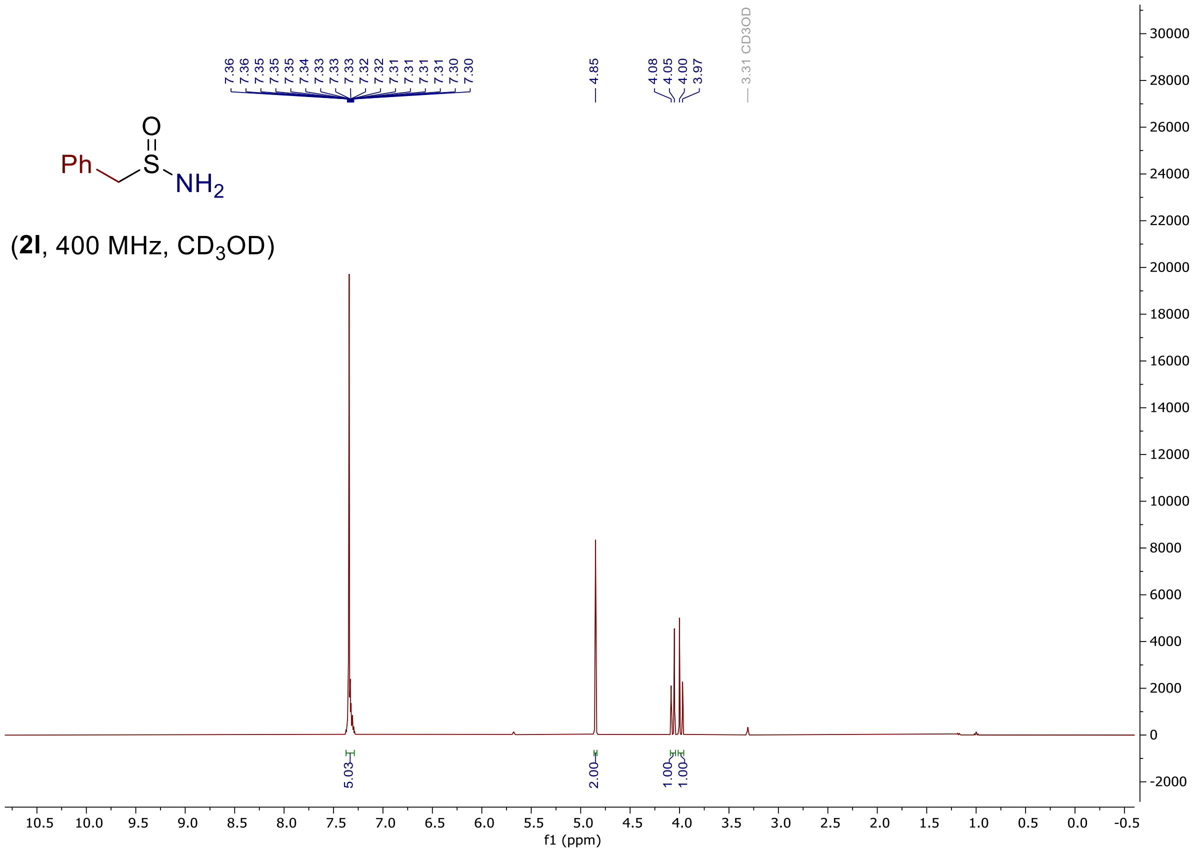

(**2l**, 400 MHz, CD_3OD)

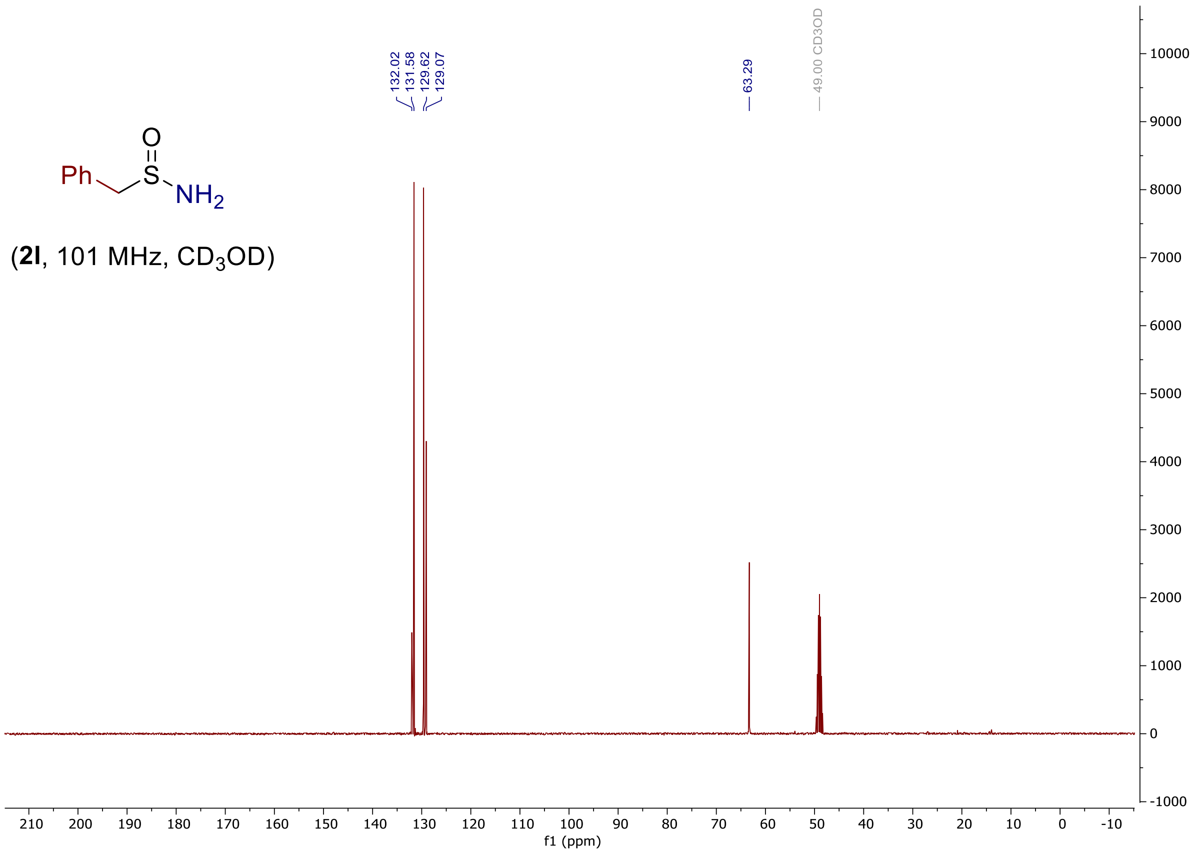
132.02
131.58
129.62
129.07
63.29
49.00 CD3OD
Ph
S
O
NH2
(2l, 101 MHz, CD3OD)
f1 (ppm)

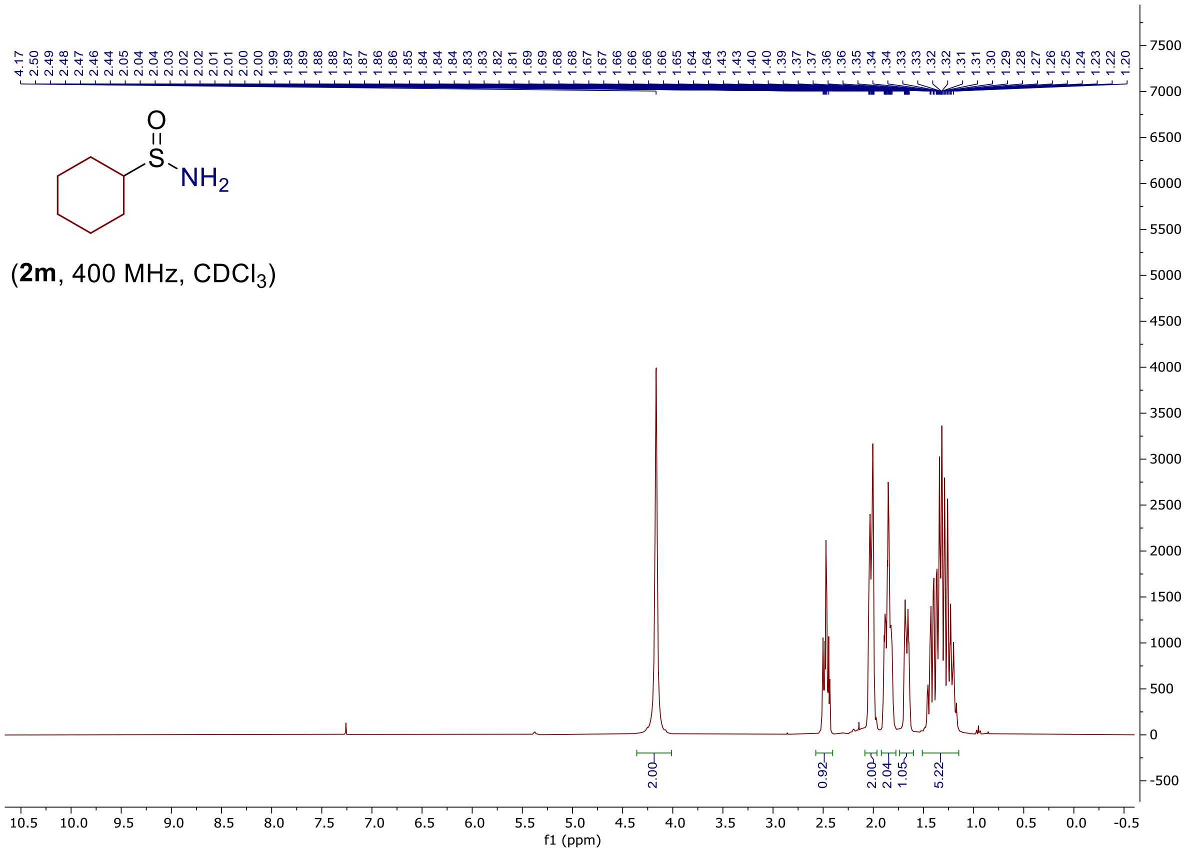
4.17
2.50
2.49
2.48
2.47
2.46
2.44
2.05
2.04
2.04
2.03
2.02
2.02
2.01
2.00
2.00
1.99
1.89
1.88
1.88
1.87
1.87
1.86
1.86
1.85
1.84
1.84
1.83
1.83
1.82
1.81
1.69
1.69
1.68
1.67
1.67
1.66
1.66
1.66
1.65
1.64
1.64
1.43
1.43
1.40
1.40
1.39
1.37
1.37
1.36
1.36
1.35
1.34
1.34
1.33
1.33
1.32
1.32
1.31
1.31
1.30
1.29
1.28
1.27
1.26
1.25
1.24
1.23
1.22
1.20
S
O
NH2
(2m, 400 MHz, CDCl3)
2.00
0.92
2.00
2.04
1.05
5.22
f1 (ppm)

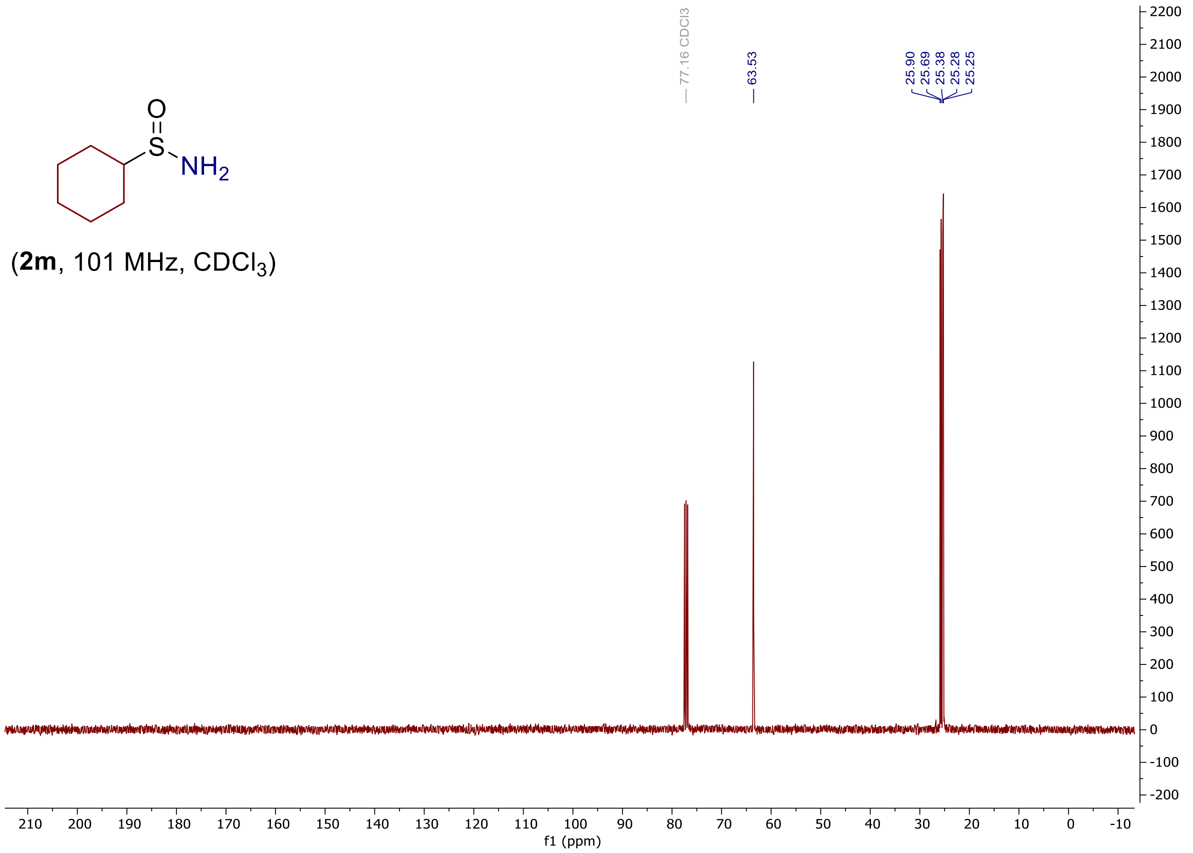

(**2m**, 101 MHz, $CDCl_3$)

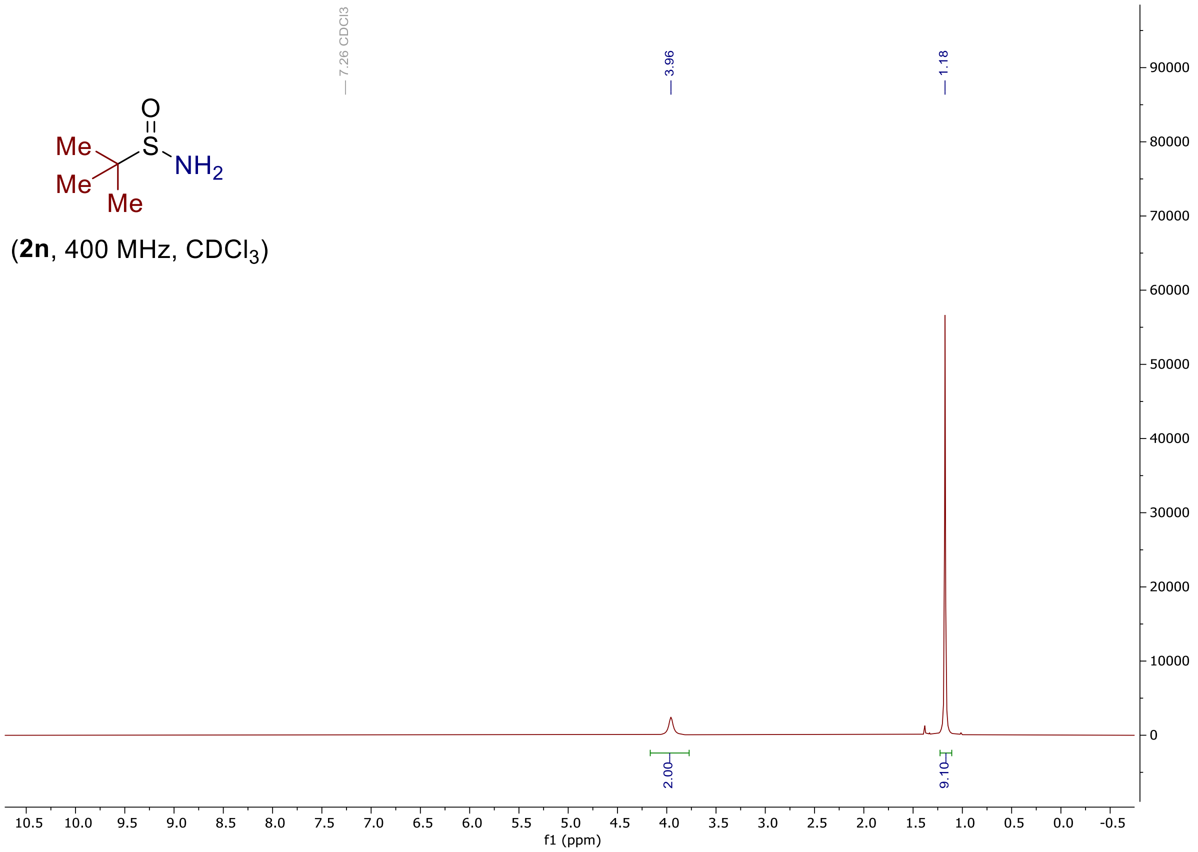

(**2n**, 400 MHz, $CDCl_3$)

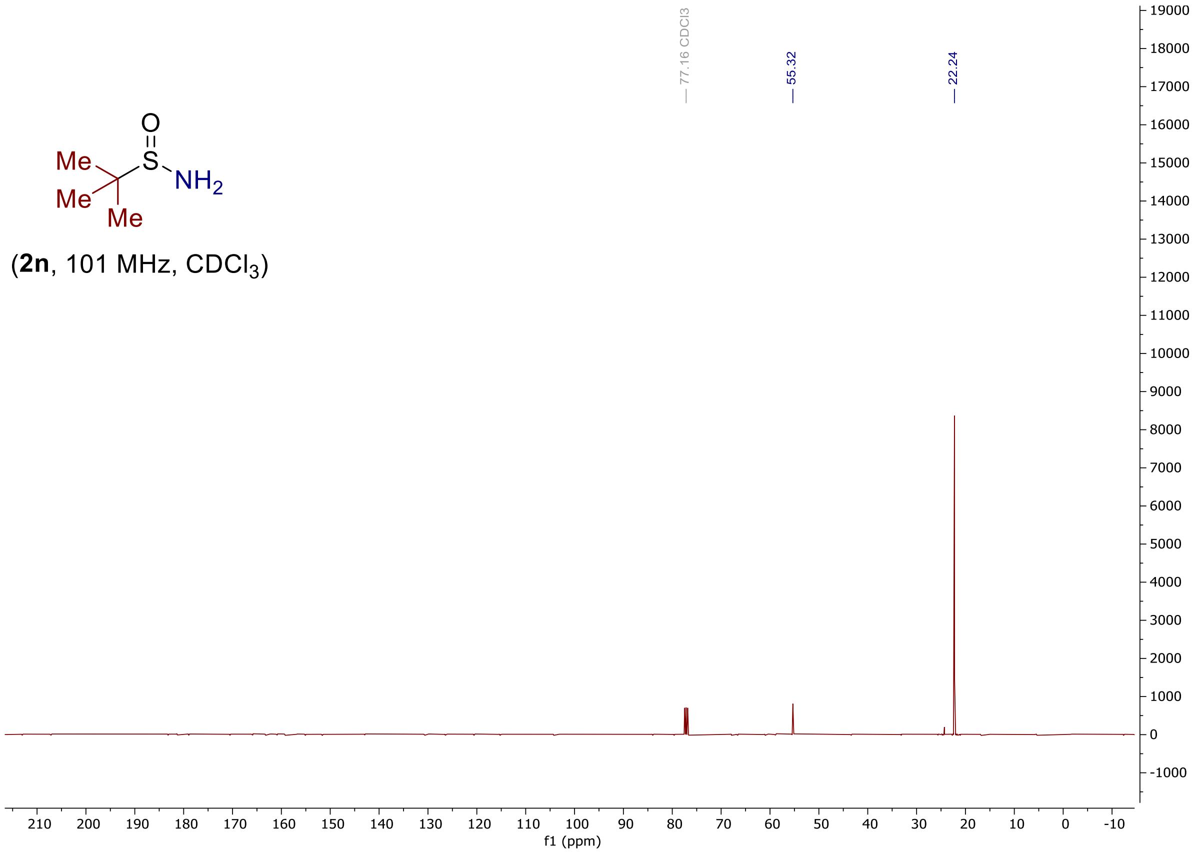

(**2n**, 101 MHz, $CDCl_3$)

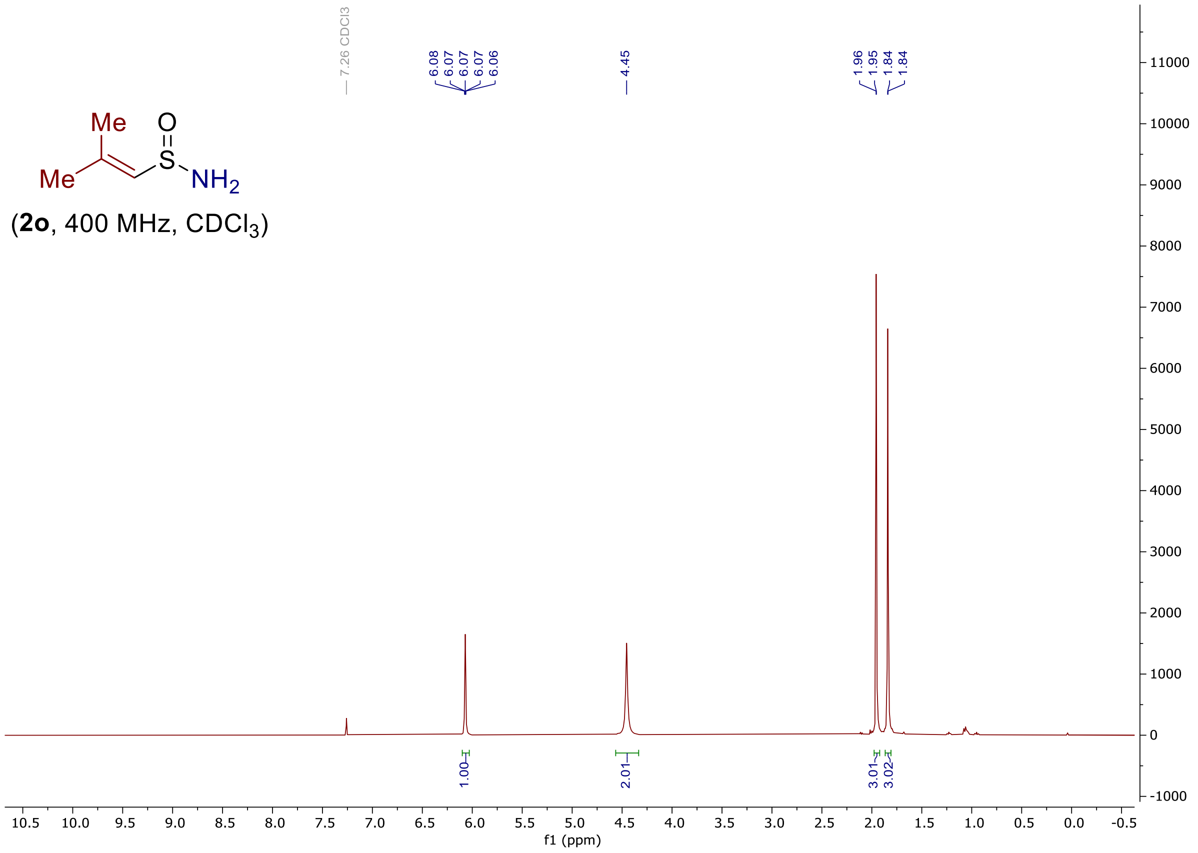

(**2o**, 400 MHz, $CDCl_3$)

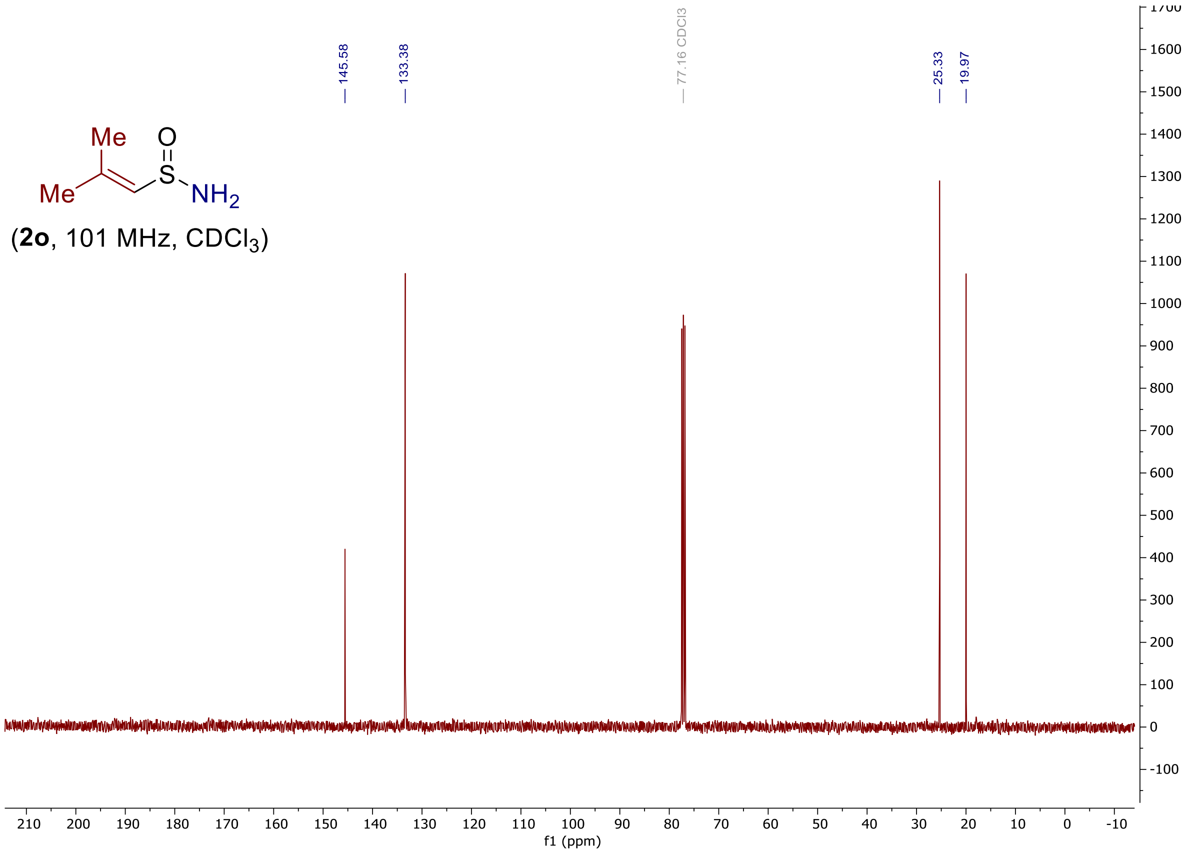

145.58
133.38
77.16 CDCl3
25.33
19.97
Me
O
S
NH2
Me
(2o, 101 MHz, CDCl3)
f1 (ppm)

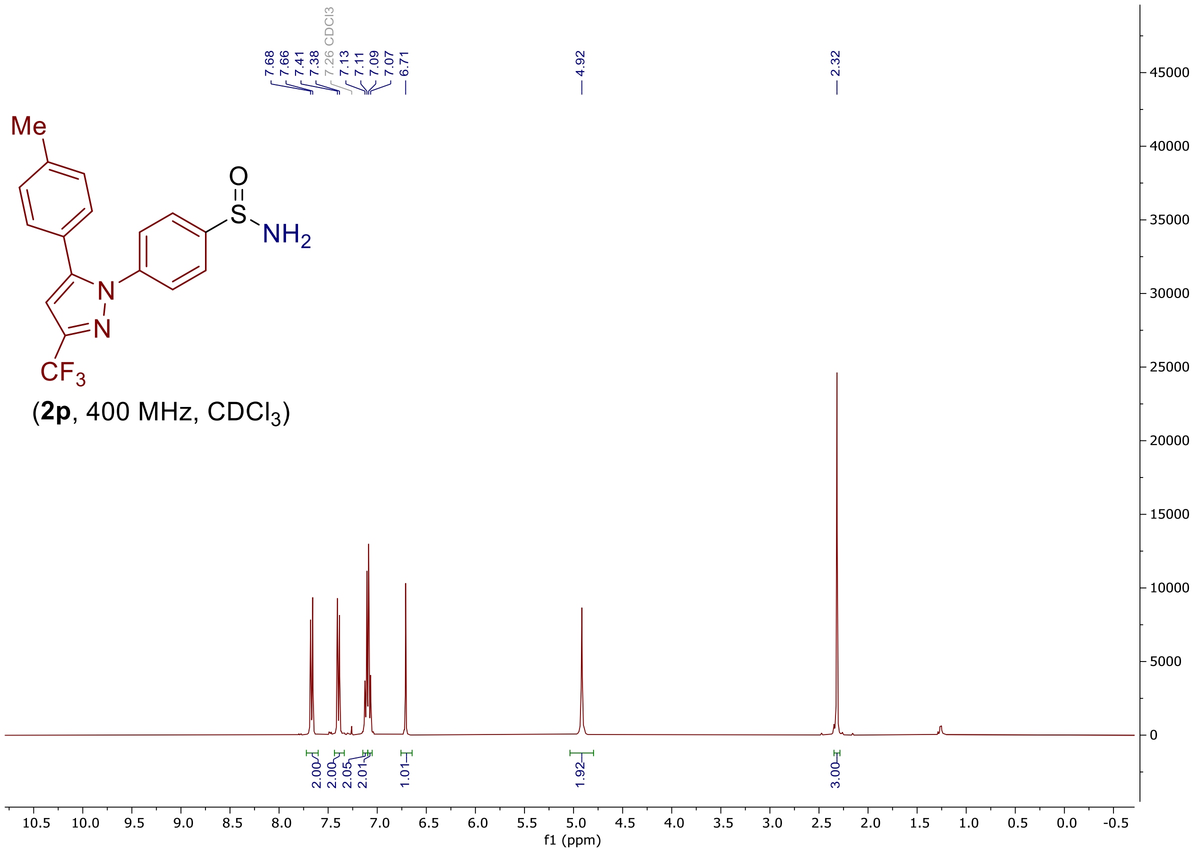

7.68
7.66
7.41
7.38
7.26 CDCl3
7.13
7.11
7.09
7.07
6.71
4.92
2.32
Me
O
S
NH2
N
N
CF3
(2p, 400 MHz, CDCl3)
2.00
2.00
2.05
2.01
1.01
1.92
3.00
f1 (ppm)

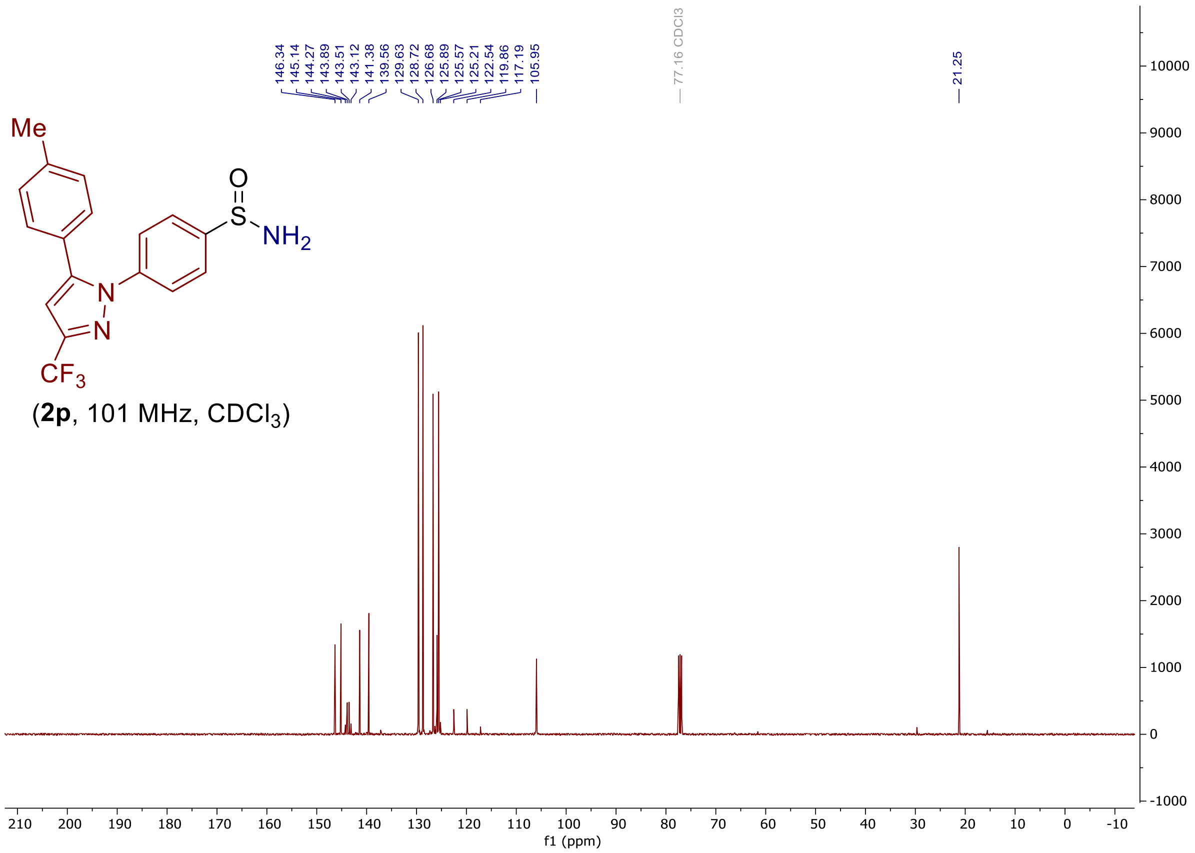
146.34
145.14
144.27
143.89
143.51
143.12
141.38
139.56
129.63
128.72
126.68
125.89
125.57
125.21
122.54
119.86
117.19
105.95
77.16 CDCl3
21.25
Me
O
S
NH2
N
N
CF3
(2p, 101 MHz, CDCl3)
f1 (ppm)

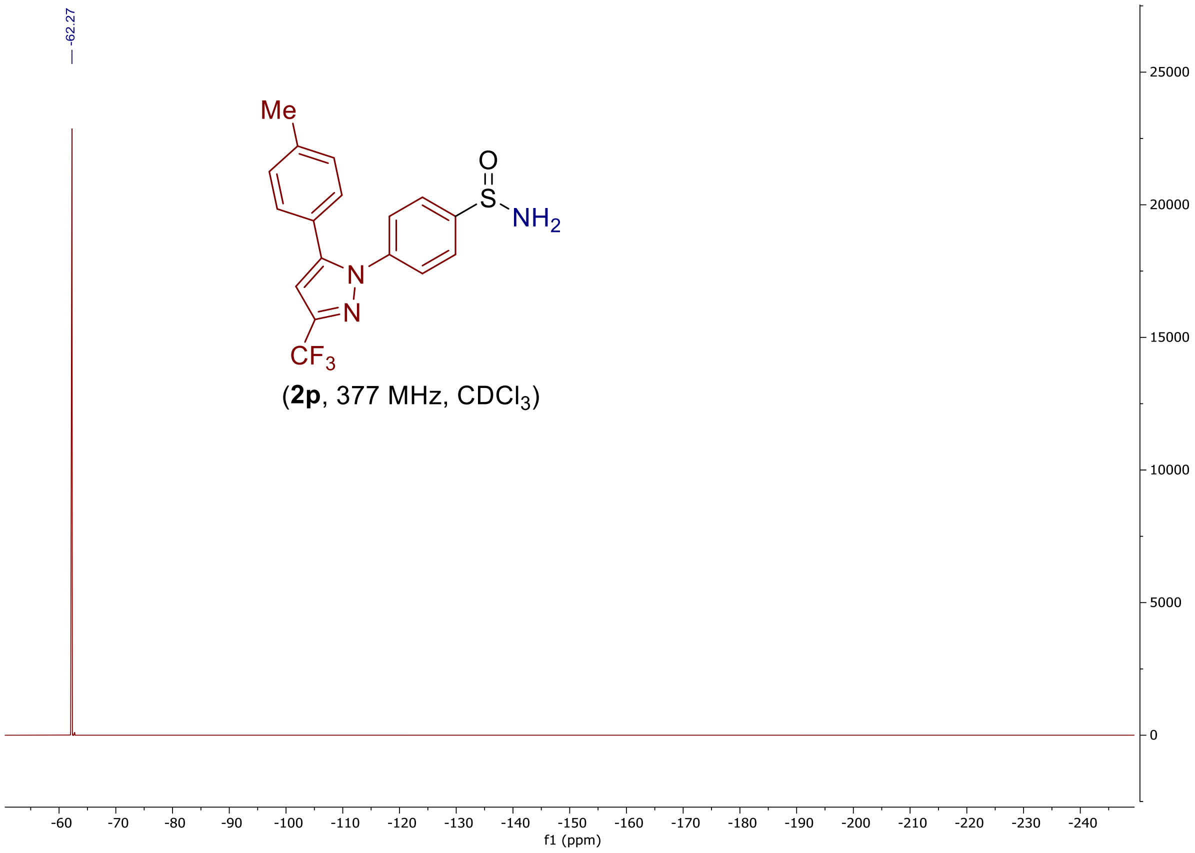
-62.27
Me
O
S
NH2
N
N
CF3
(2p, 377 MHz, CDCl3)
f1 (ppm)

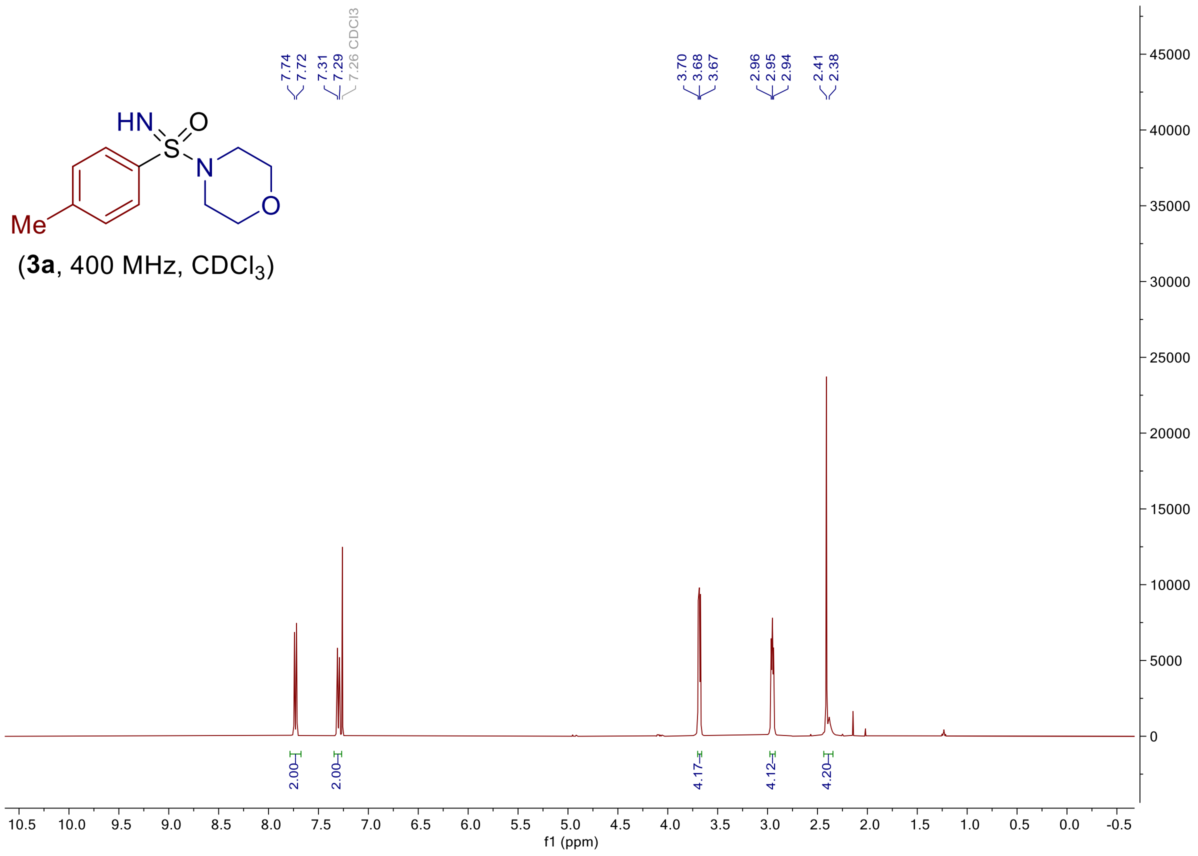
7.74
7.72
7.31
7.29
7.26 CDCl3
3.70
3.68
3.67
2.96
2.95
2.94
2.41
2.38
HN
S
O
N
O
Me
(3a, 400 MHz, CDCl3)
2.00
2.00
4.17
4.12
4.20
f1 (ppm)

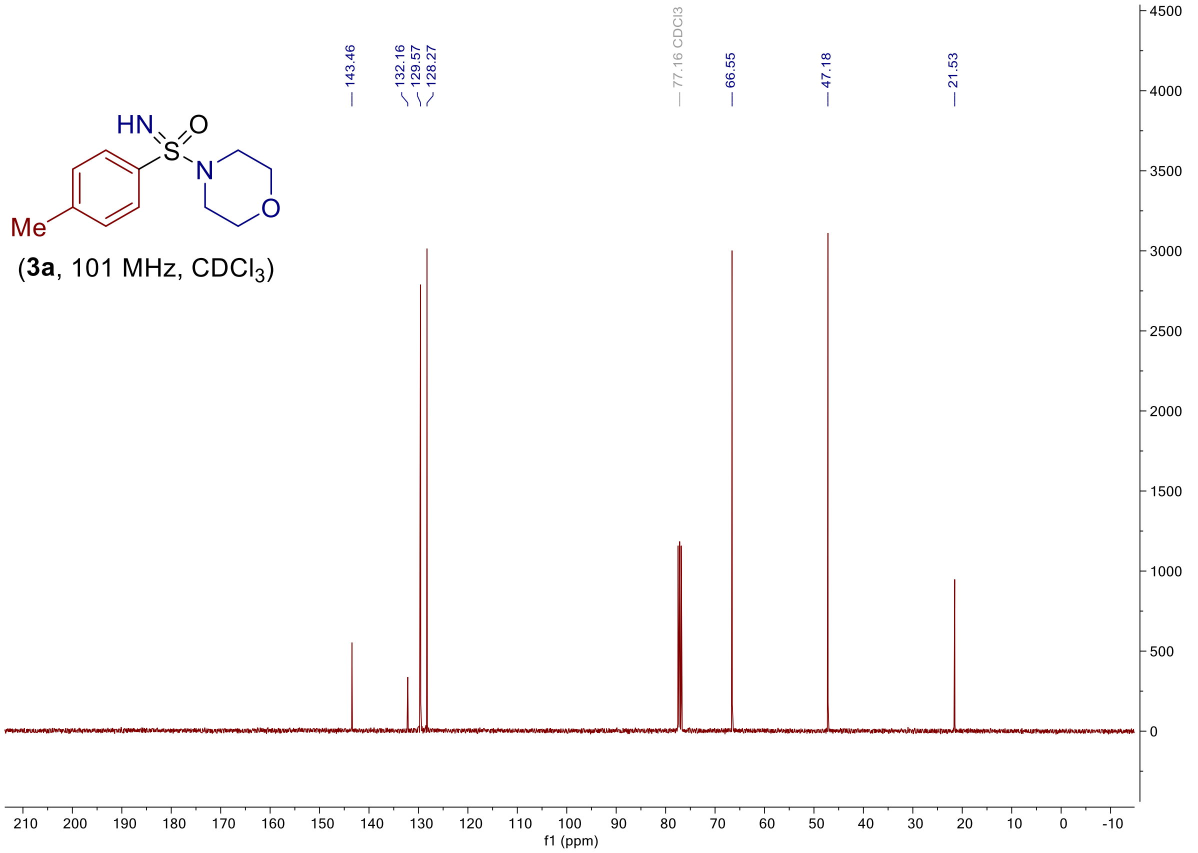
143.46
132.16
129.57
128.27
77.16 CDCl3
66.55
47.18
21.53
HN
S
O
N
O
Me
(3a, 101 MHz, CDCl3)
f1 (ppm)

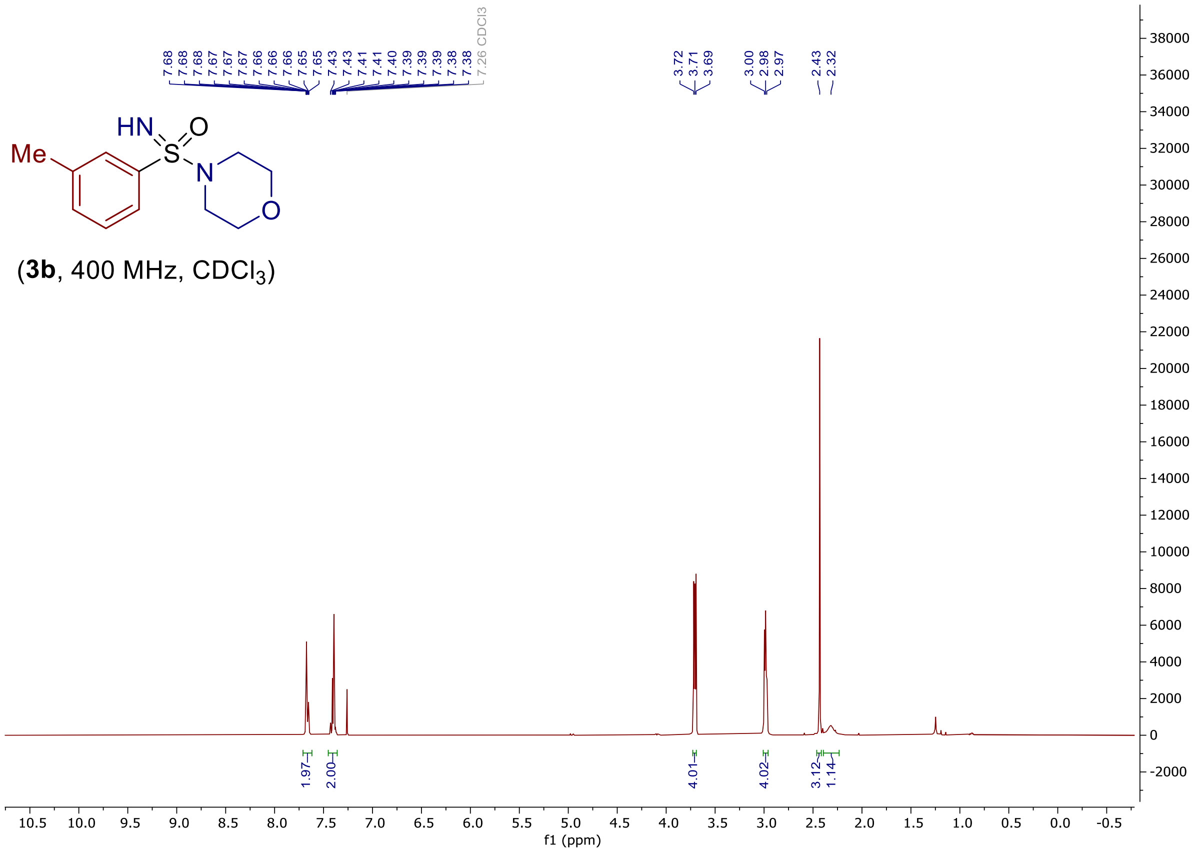
7.68
7.68
7.68
7.67
7.67
7.67
7.66
7.66
7.65
7.65
7.43
7.43
7.41
7.41
7.40
7.39
7.39
7.39
7.38
7.38
7.26 CDCl3
3.72
3.71
3.69
3.00
2.98
2.97
2.43
2.32
Me
HN
S
O
N
O
(3b, 400 MHz, CDCl3)
1.97
2.00
4.01
4.02
3.12
1.14
f1 (ppm)

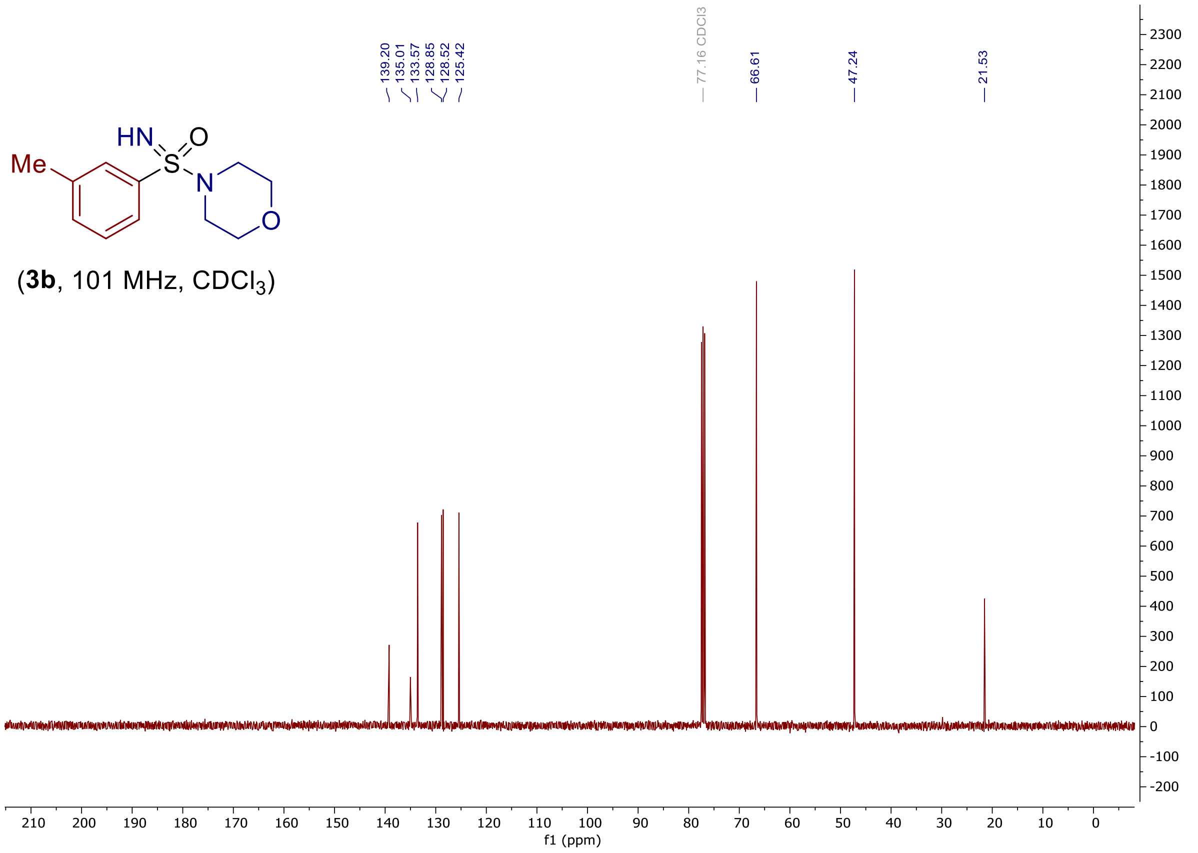
139.20
135.01
133.57
128.85
128.52
125.42
77.16 CDCl3
66.61
47.24
21.53
Me
HN
S
O
N
O
(3b, 101 MHz, CDCl3)
f1 (ppm)

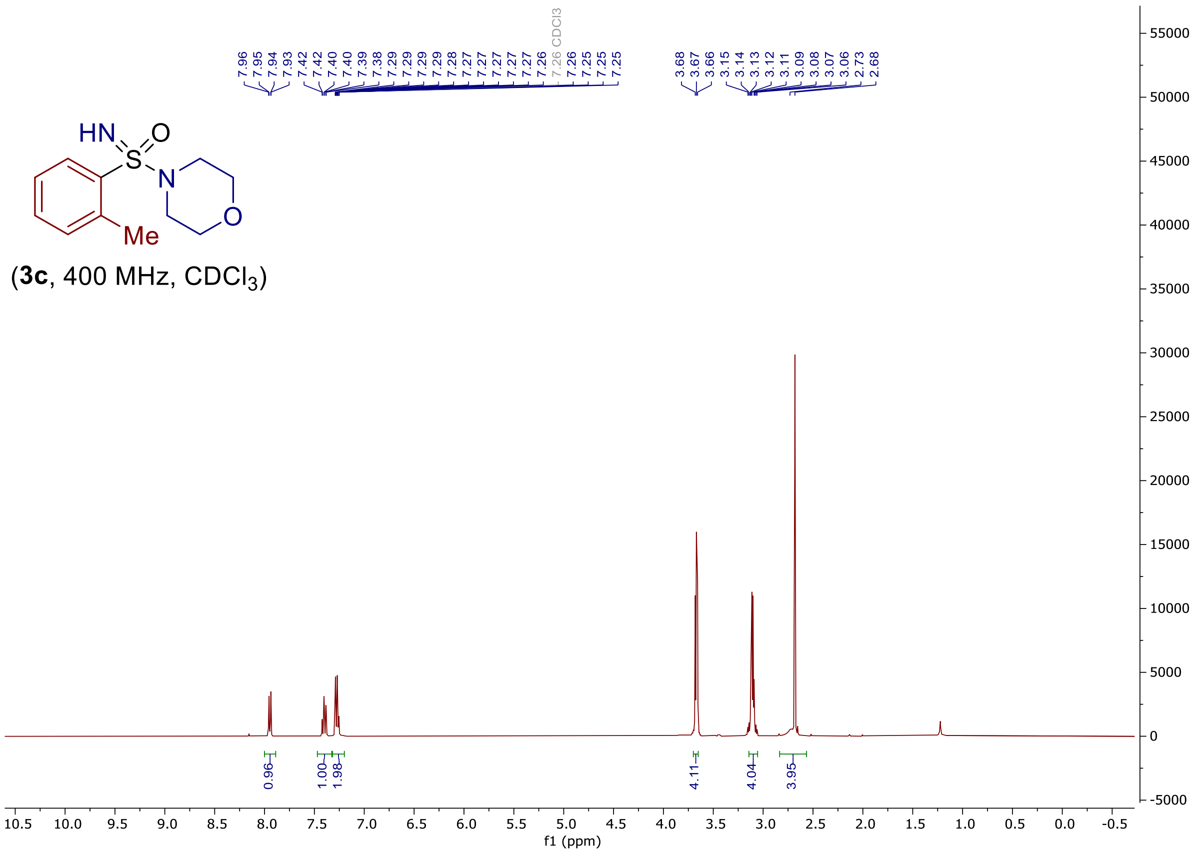
HN
O
S
N
O
Me
(3c, 400 MHz, CDCl3)
7.96 7.95 7.94 7.93 7.42 7.42 7.40 7.40 7.39 7.38 7.29 7.29 7.29 7.29 7.28 7.27 7.27 7.27 7.27 7.27 7.26 7.26 CDCl3 7.26 7.25 7.25 7.25
3.68 3.67 3.66 3.15 3.14 3.13 3.12 3.11 3.09 3.08 3.07 3.06 2.73 2.68
0.96 1.00 1.98 4.11 4.04 3.95
f1 (ppm)

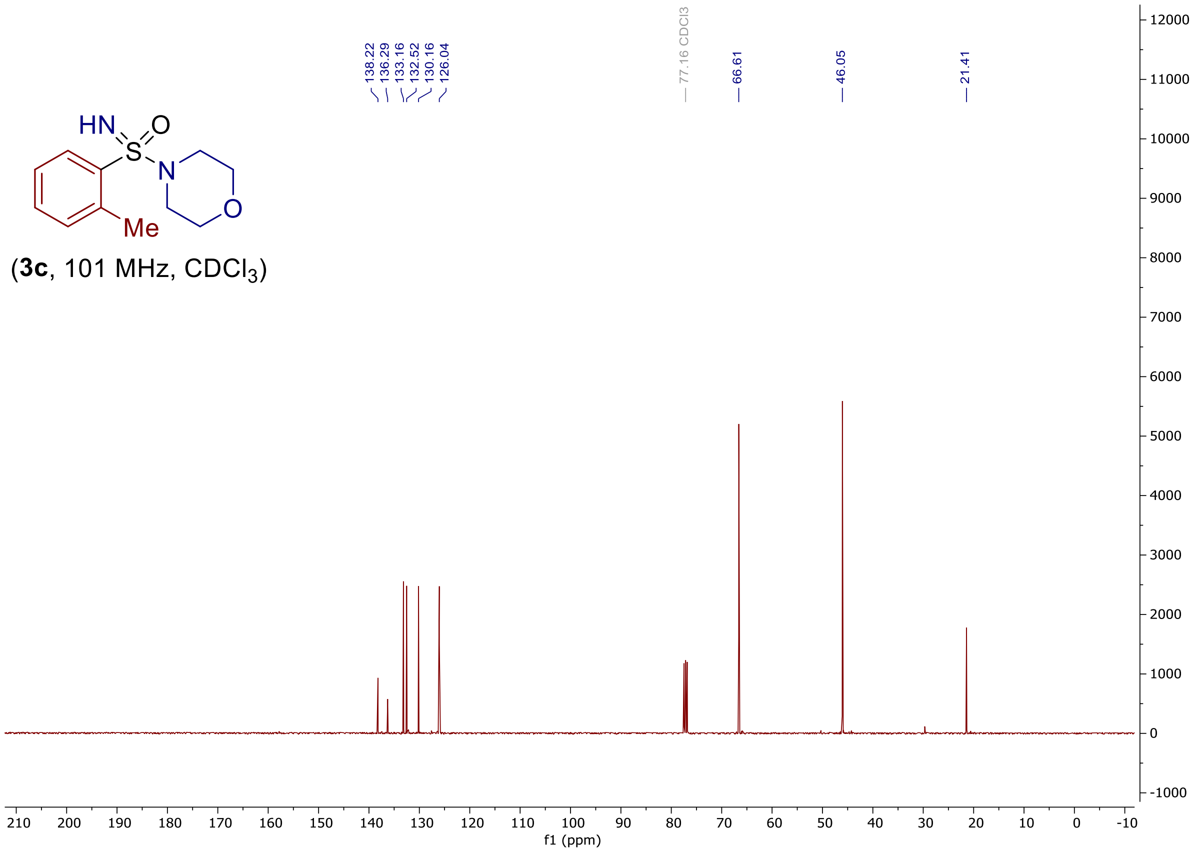
HN
O
S
N
O
Me
(3c, 101 MHz, CDCl3)
138.22 136.29 133.16 132.52 130.16 126.04
77.16 CDCl3
66.61
46.05
21.41
f1 (ppm)

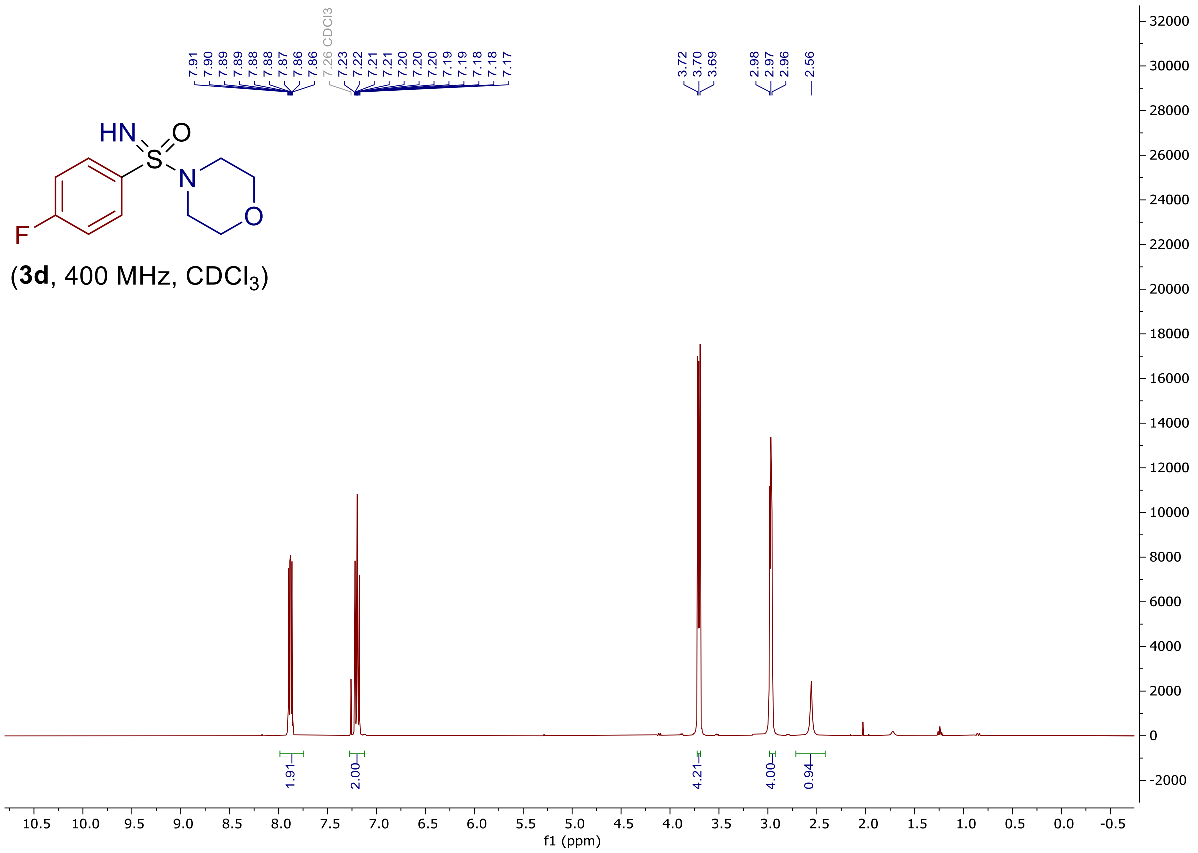

(**3d**, 400 MHz, $CDCl_3$)

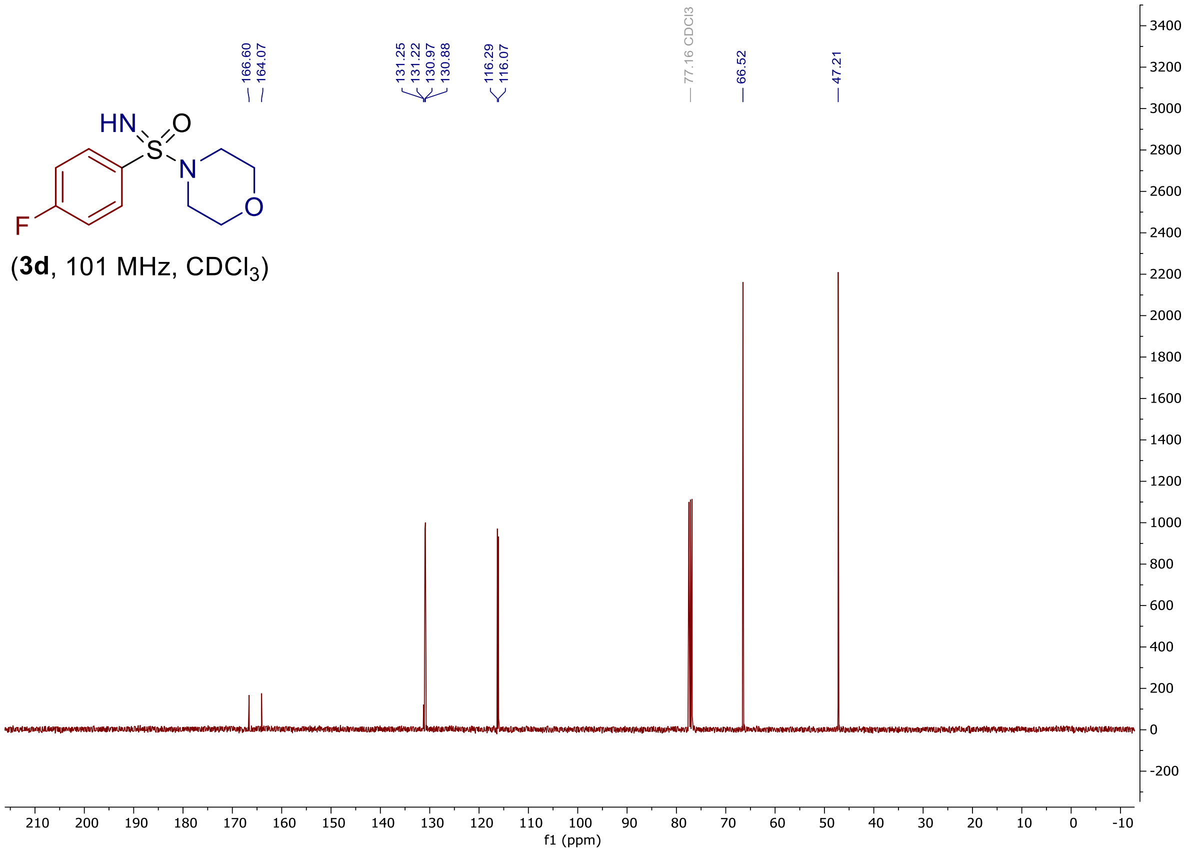

(**3d**, 101 MHz, $CDCl_3$)

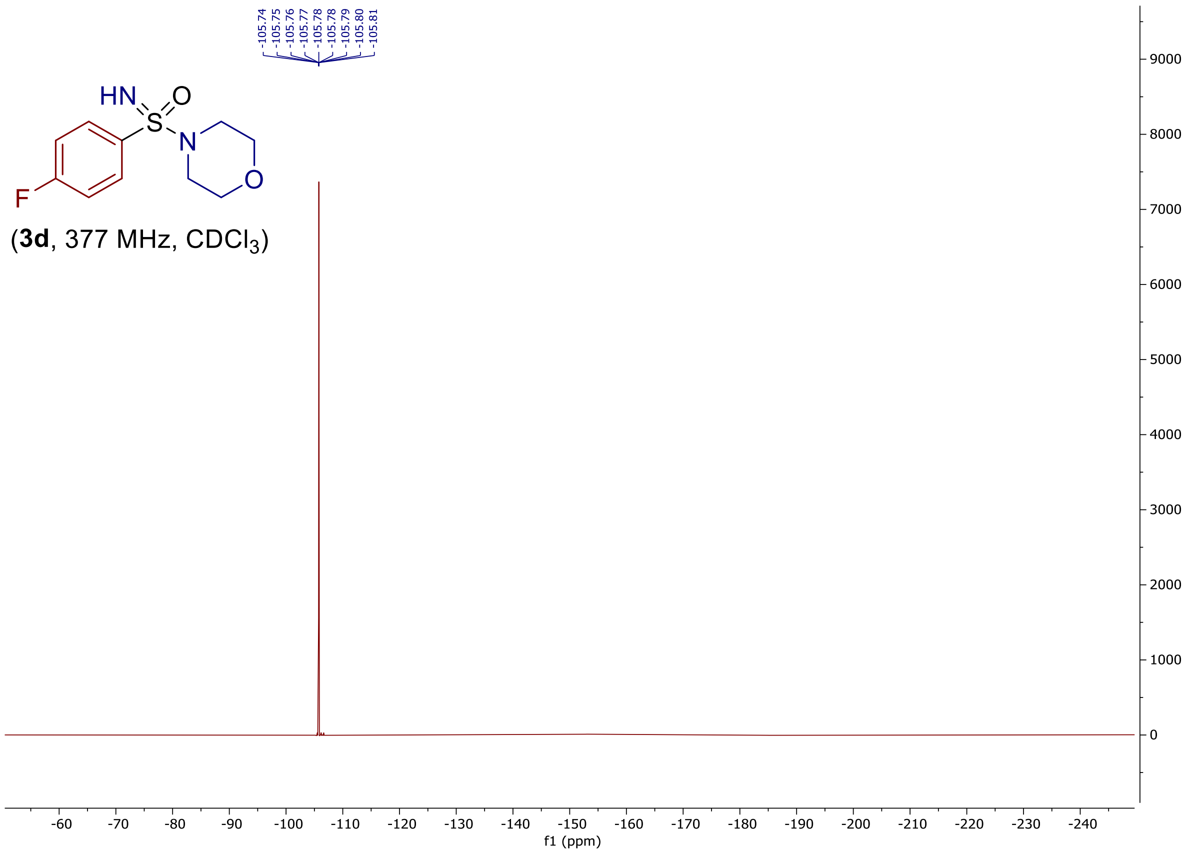

(**3d**, 377 MHz, $CDCl_3$)

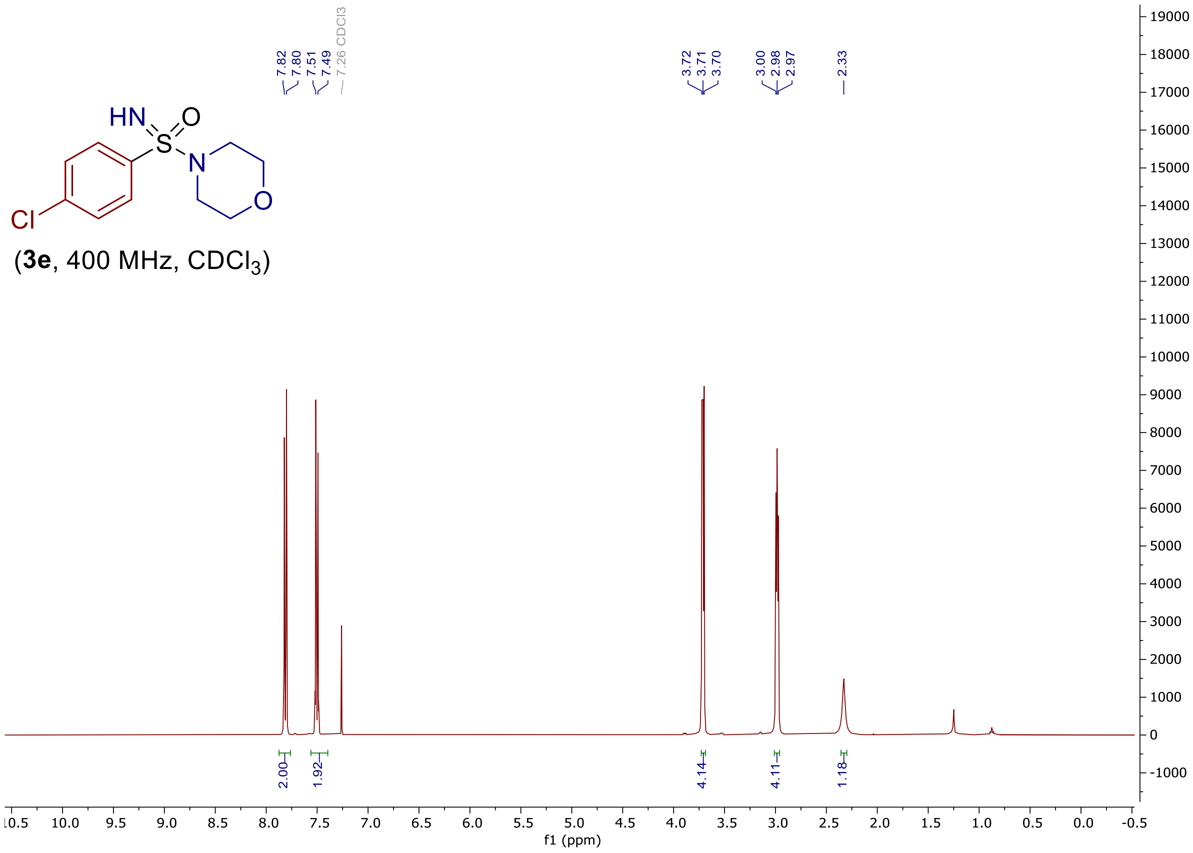

(**3e**, 400 MHz, $CDCl_3$)

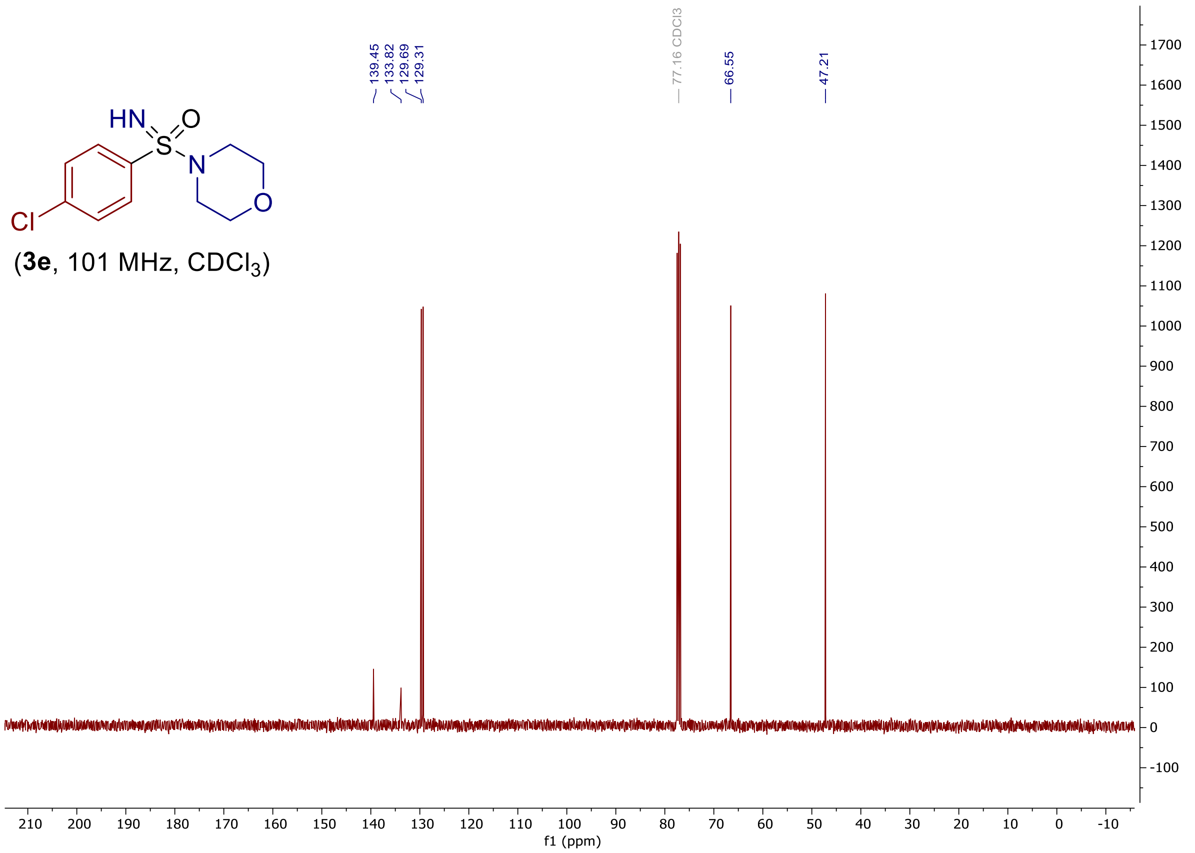
(3e, 101 MHz, CDCl3)
139.45
133.82
129.69
129.31
77.16 CDCl3
66.55
47.21
f1 (ppm)

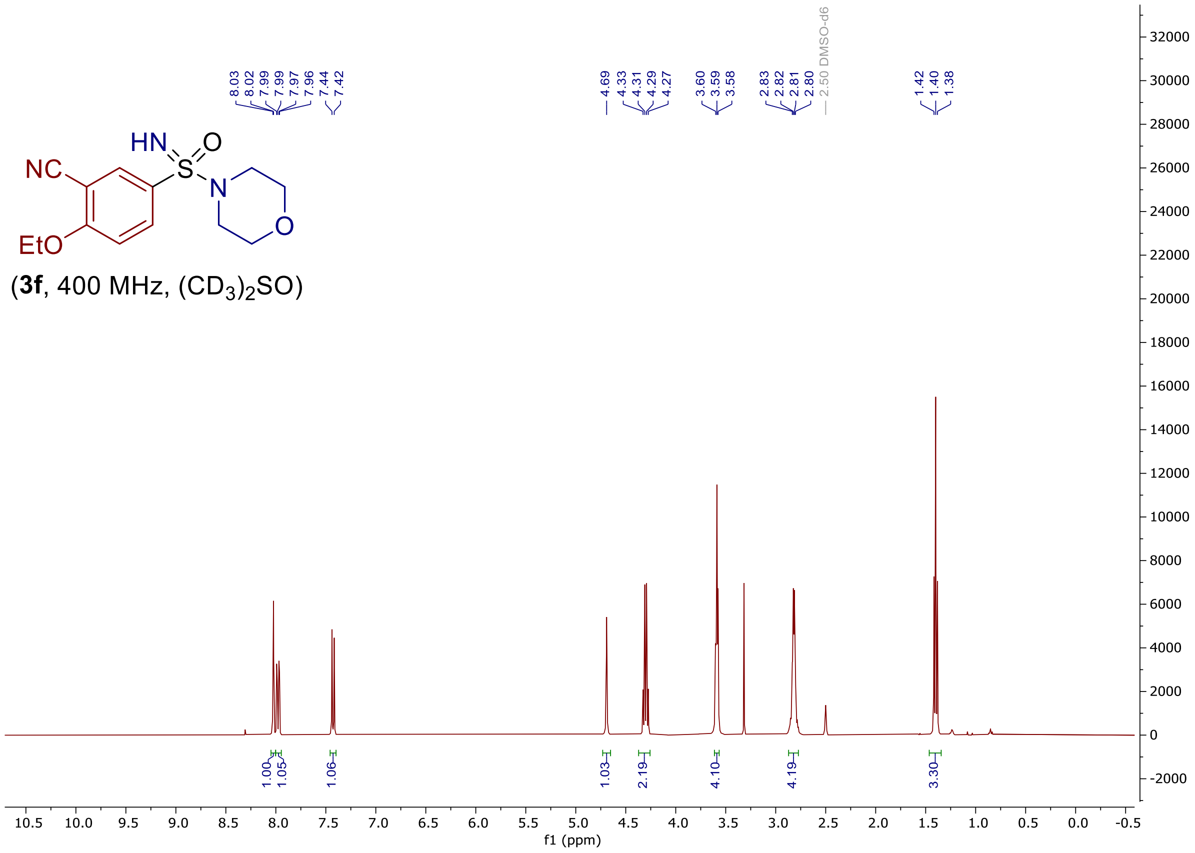
(3f, 400 MHz, (CD3)2SO)
8.03
8.02
7.99
7.99
7.97
7.96
7.44
7.42
4.69
4.33
4.31
4.29
4.27
3.60
3.59
3.58
2.83
2.82
2.81
2.80
2.50 DMSO-d6
1.42
1.40
1.38
1.00
1.05
1.06
1.03
2.19
4.10
4.19
3.30
f1 (ppm)

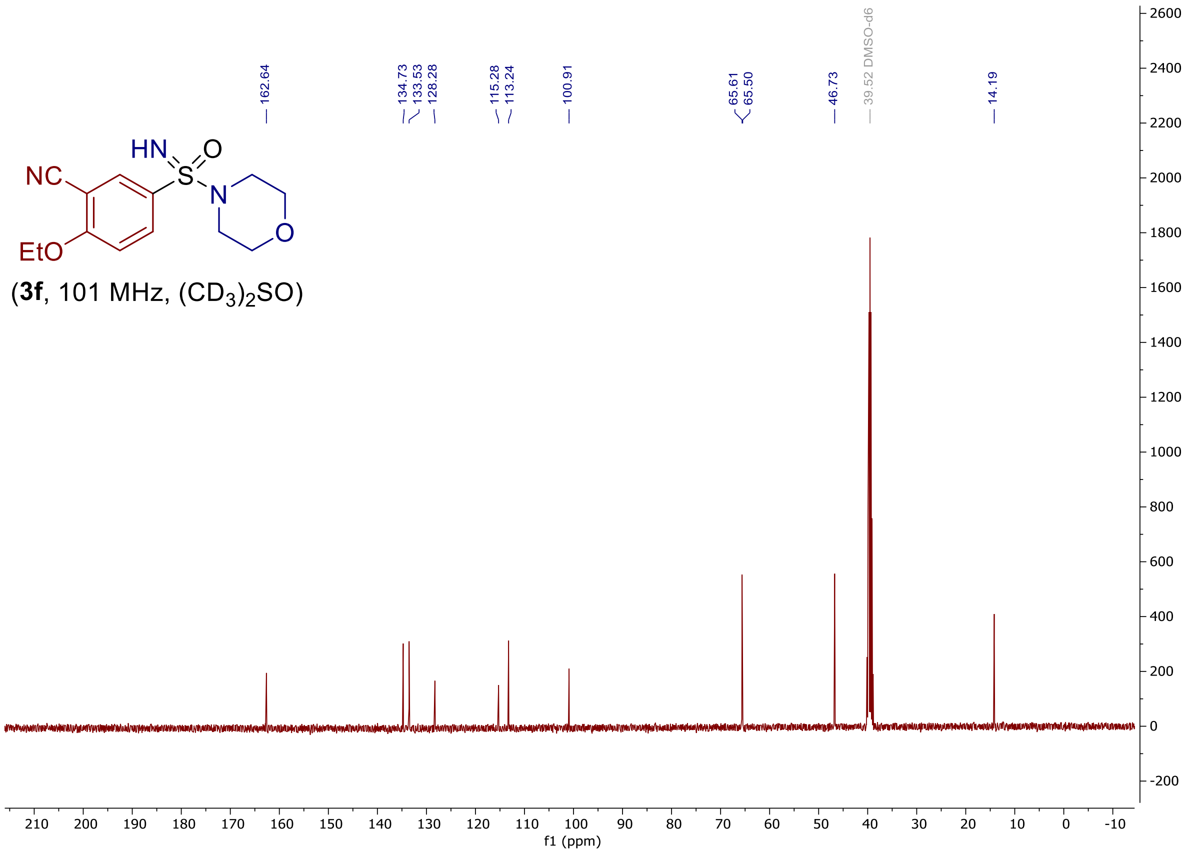
162.64
134.73
133.53
128.28
115.28
113.24
100.91
65.61
65.50
46.73
39.52 DMSO-d6
14.19
NC
HN
S
O
N
O
EtO
(3f, 101 MHz, (CD3)2SO)
f1 (ppm)

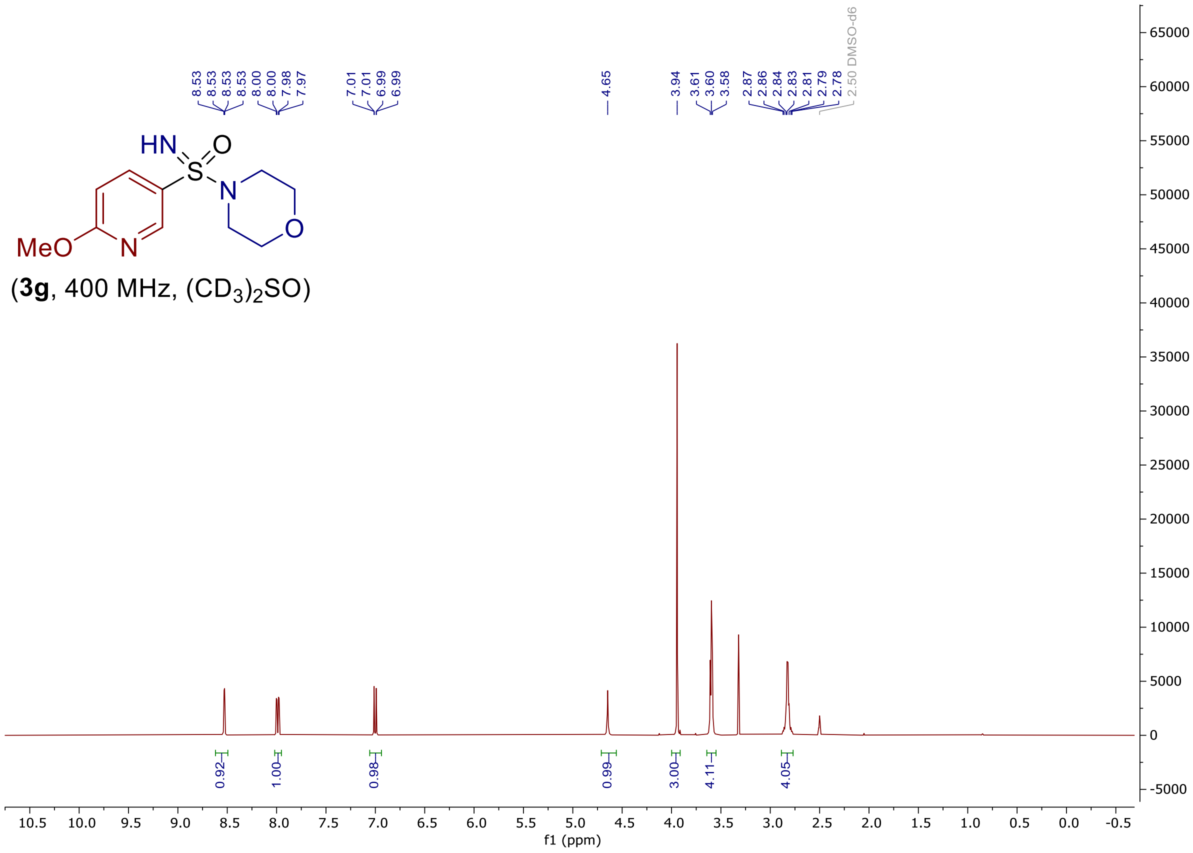
8.53
8.53
8.53
8.53
8.00
8.00
7.98
7.97
7.01
7.01
6.99
6.99
4.65
3.94
3.61
3.60
3.58
2.87
2.86
2.84
2.83
2.81
2.79
2.78
2.50 DMSO-d6
HN
S
O
N
O
MeO
N
(3g, 400 MHz, (CD3)2SO)
0.92
1.00
0.98
0.99
3.00
4.11
4.05
f1 (ppm)

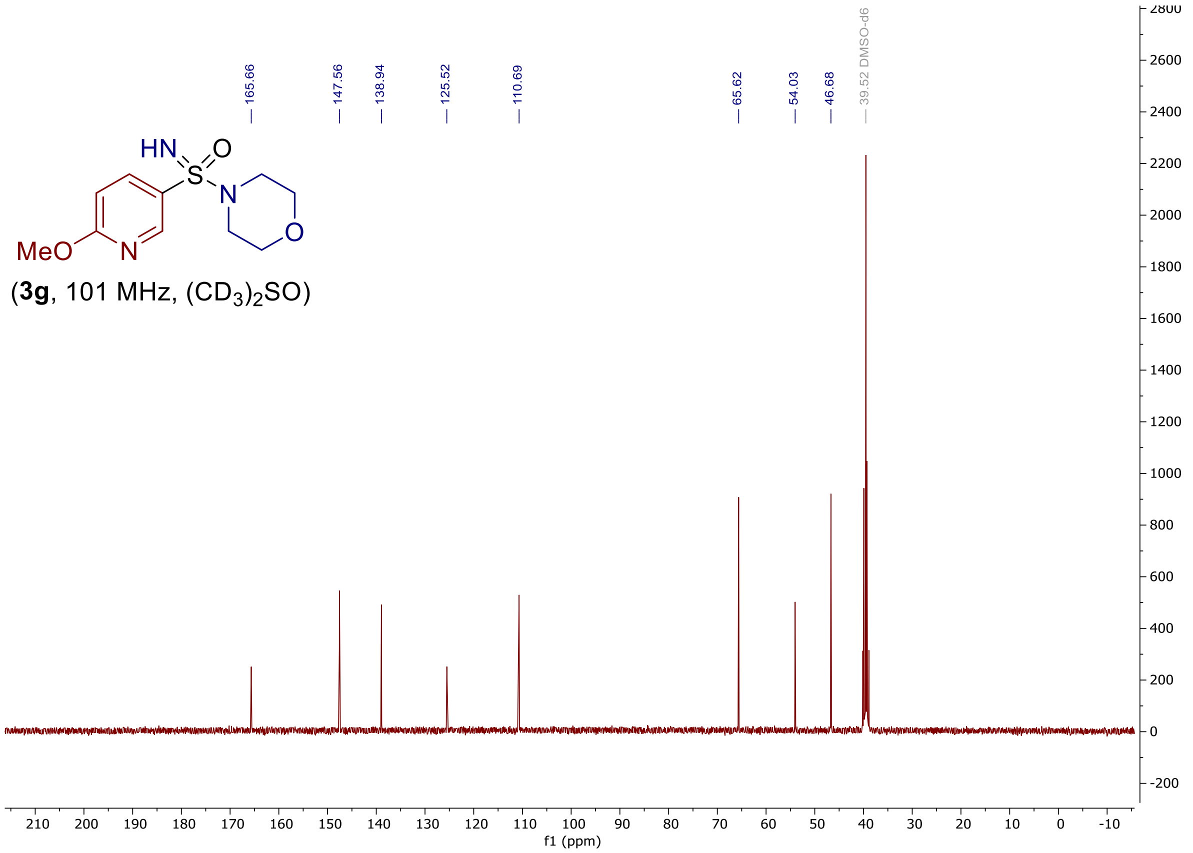

(**3g**, 101 MHz, $(CD_3)_2SO$)

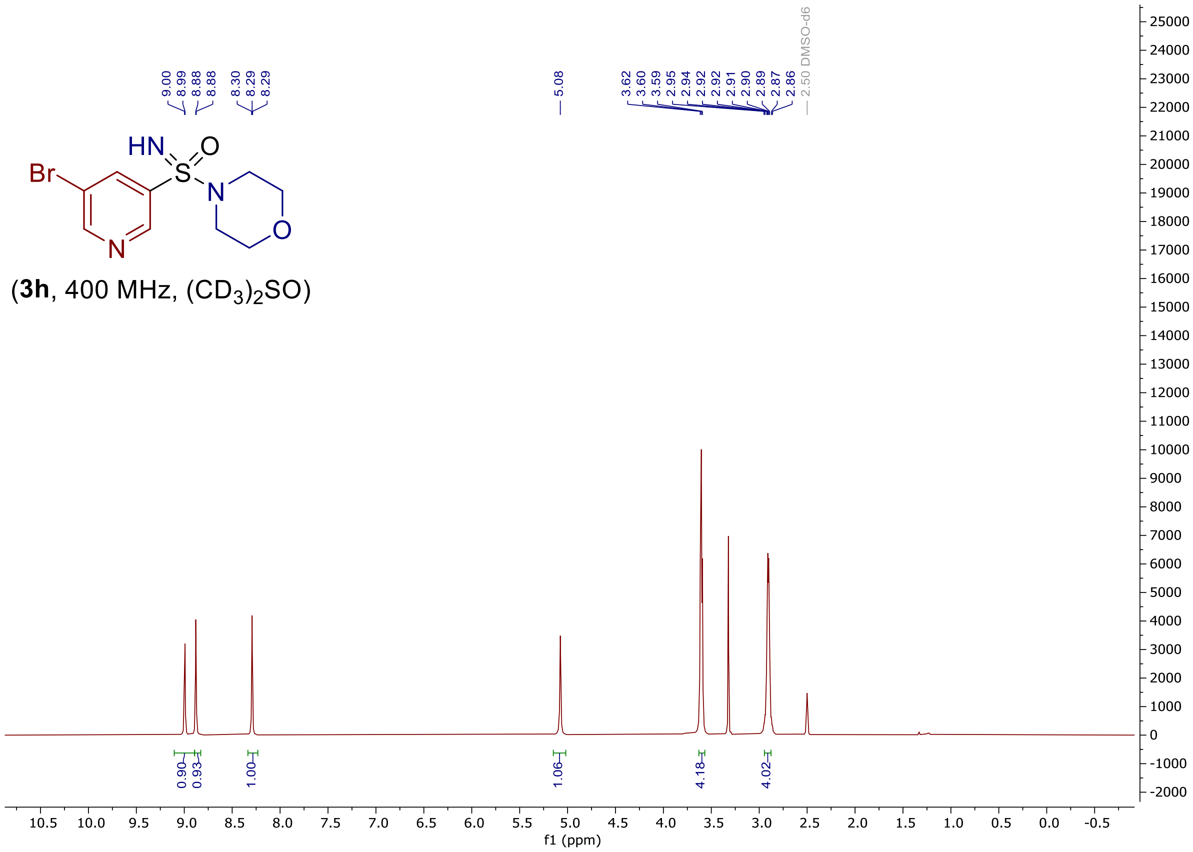

(**3h**, 400 MHz, $(CD_3)_2SO$)

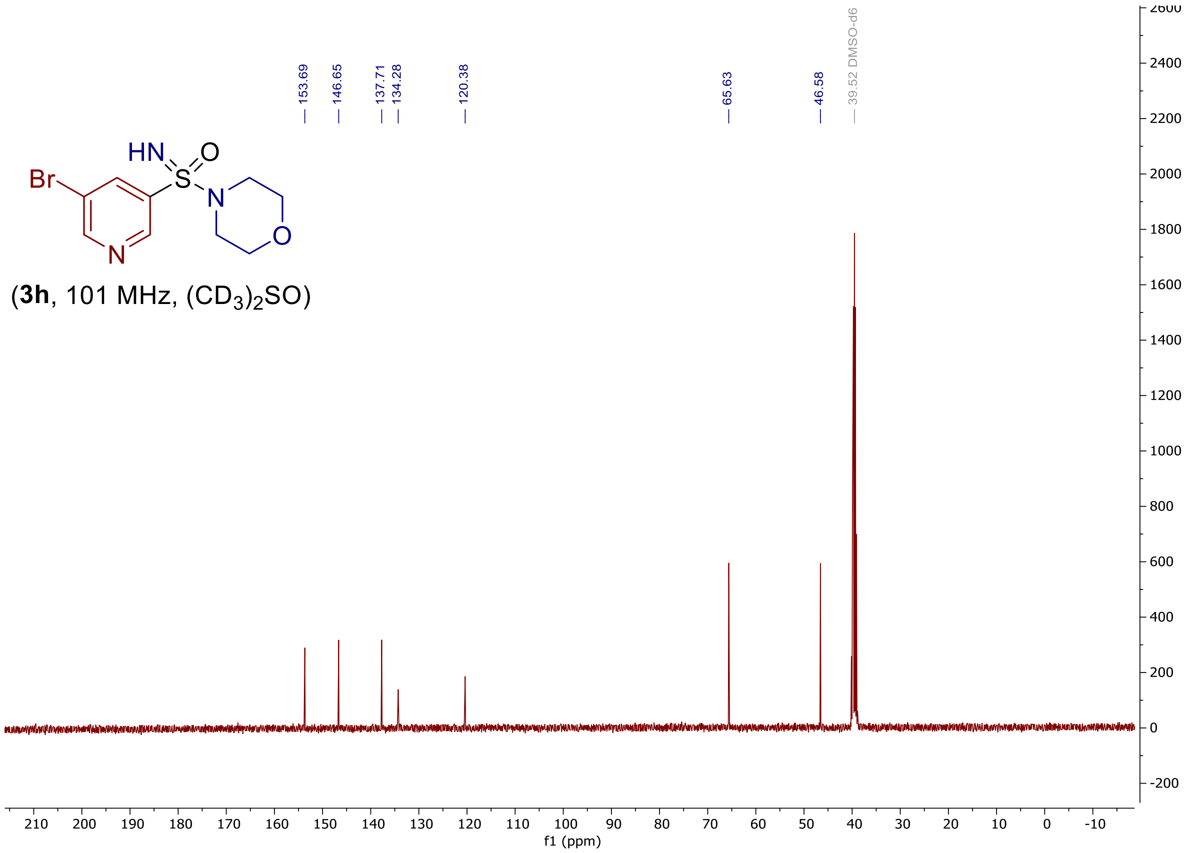

(**3h**, 101 MHz, $(CD_3)_2SO$)

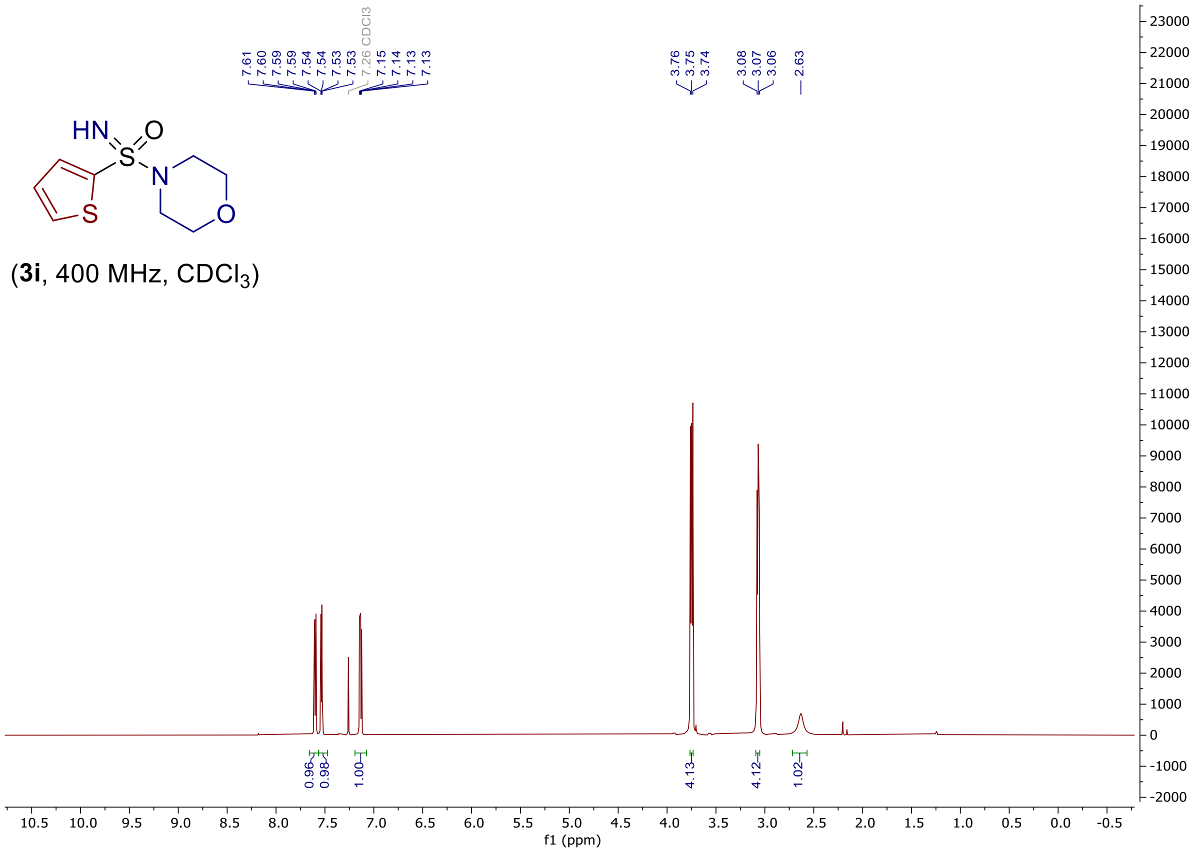

(**3i**, 400 MHz, $CDCl_3$)

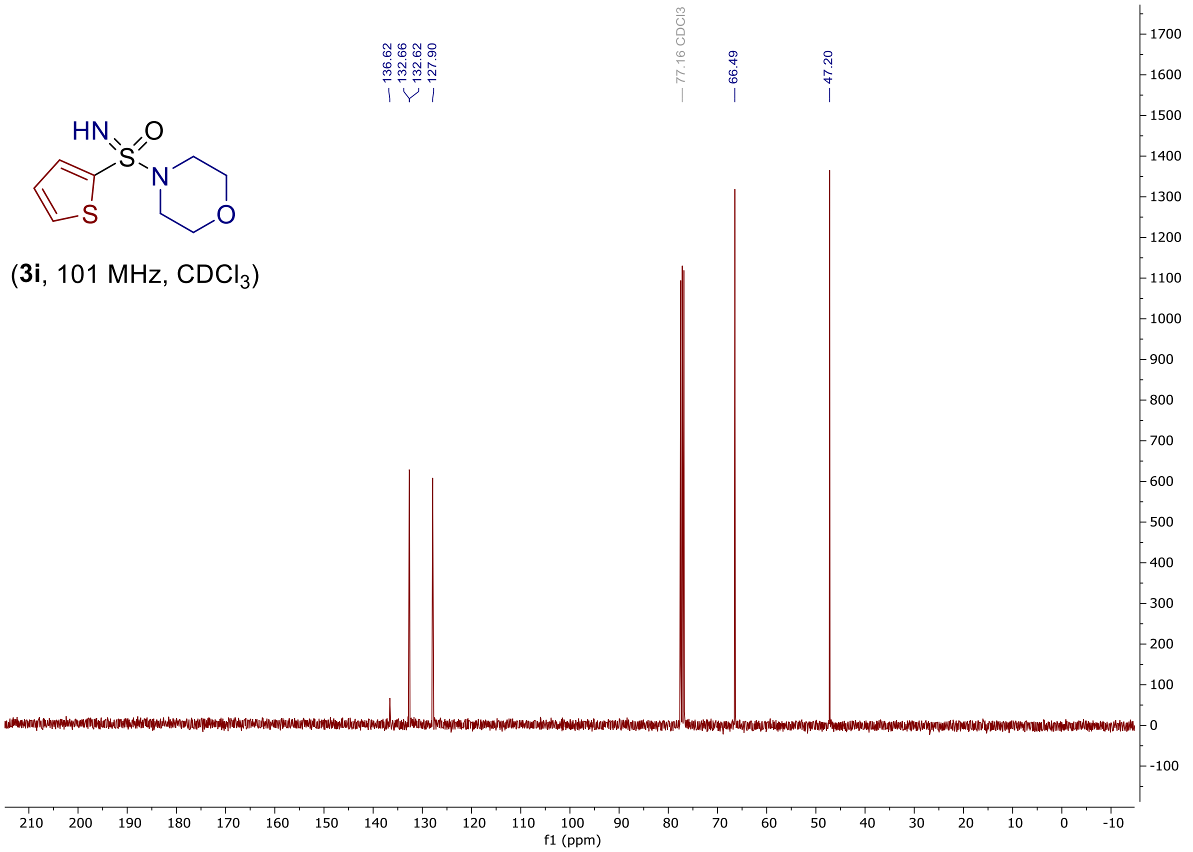

(**3i**, 101 MHz, $CDCl_3$)

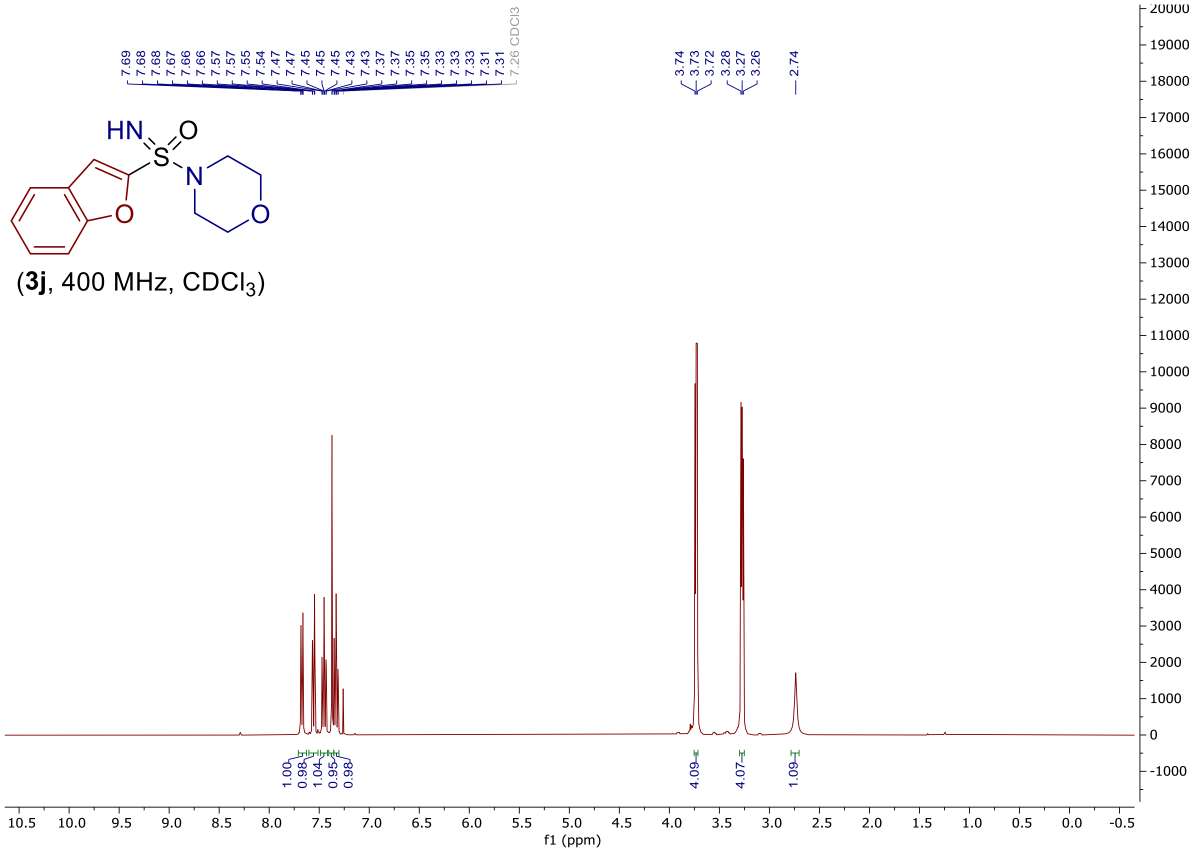

(**3j**, 400 MHz, $CDCl_3$)

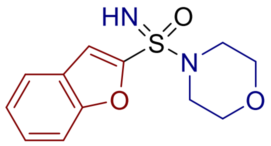

(**3j**, 101 MHz, $CDCl_3$)

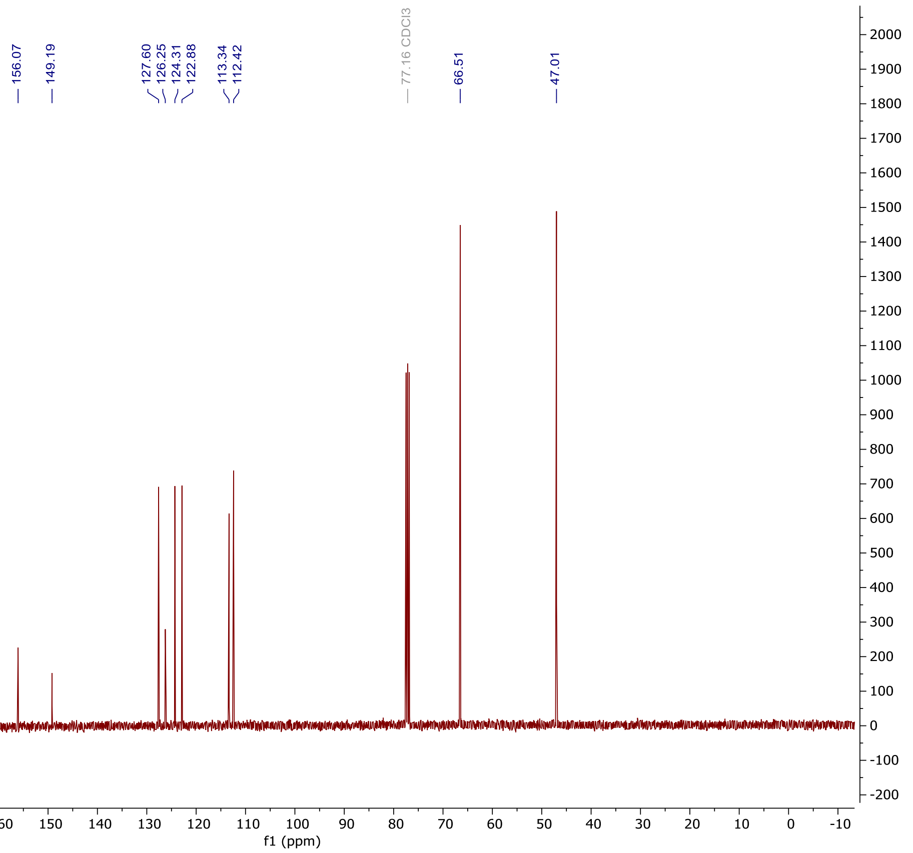

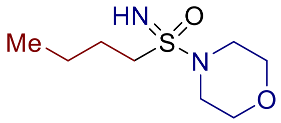

(**3k**, 400 MHz, $CDCl_3$)

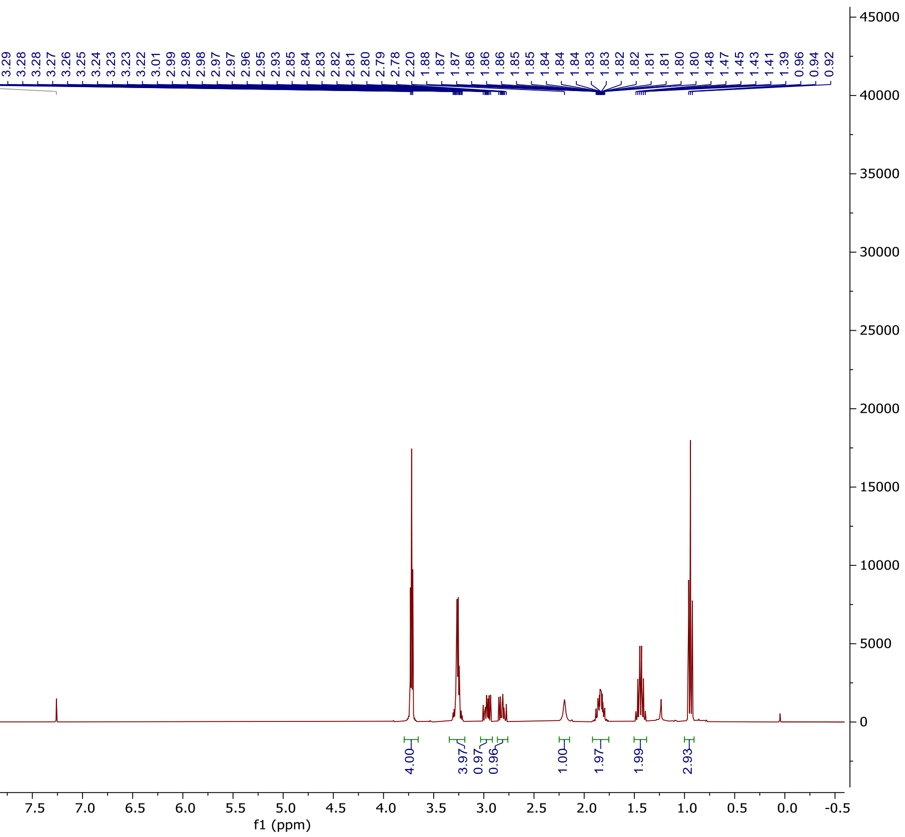

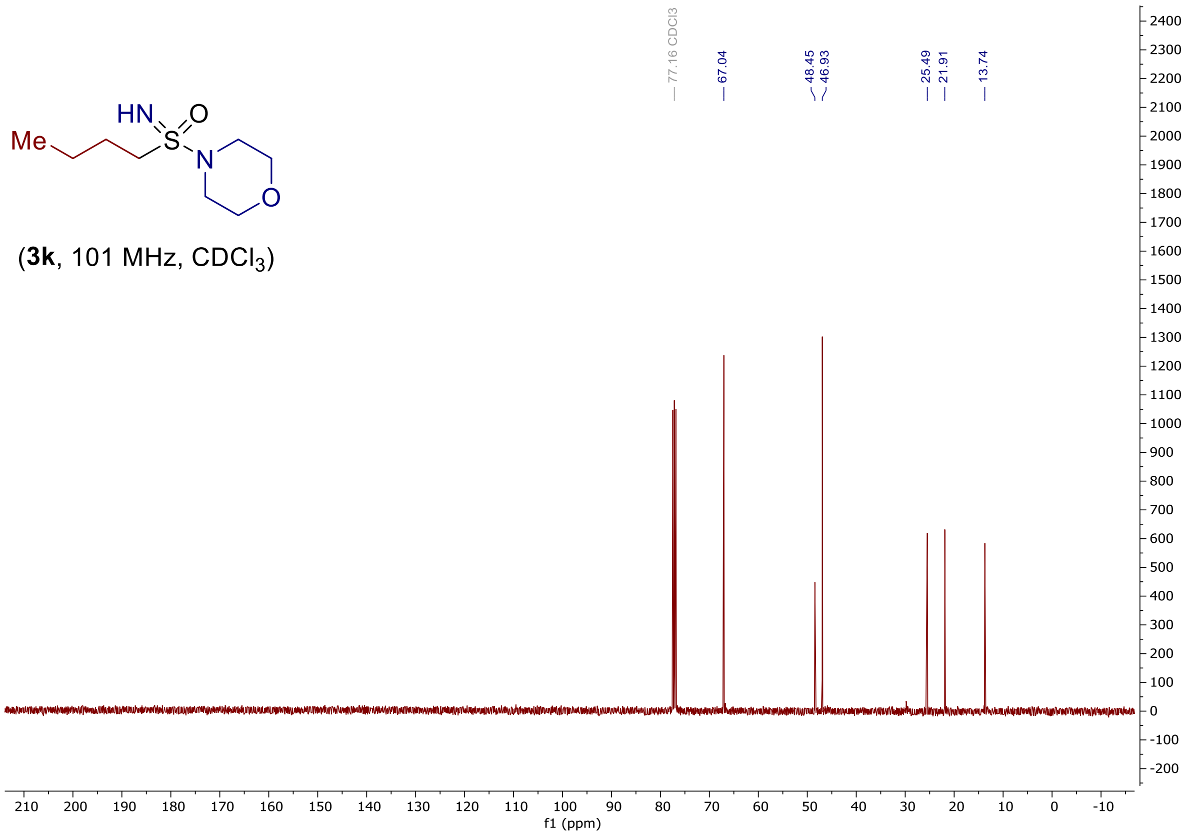

(3k, 101 MHz, CDCl3)
77.16 CDCl3
67.04
48.45
46.93
25.49
21.91
13.74
f1 (ppm)

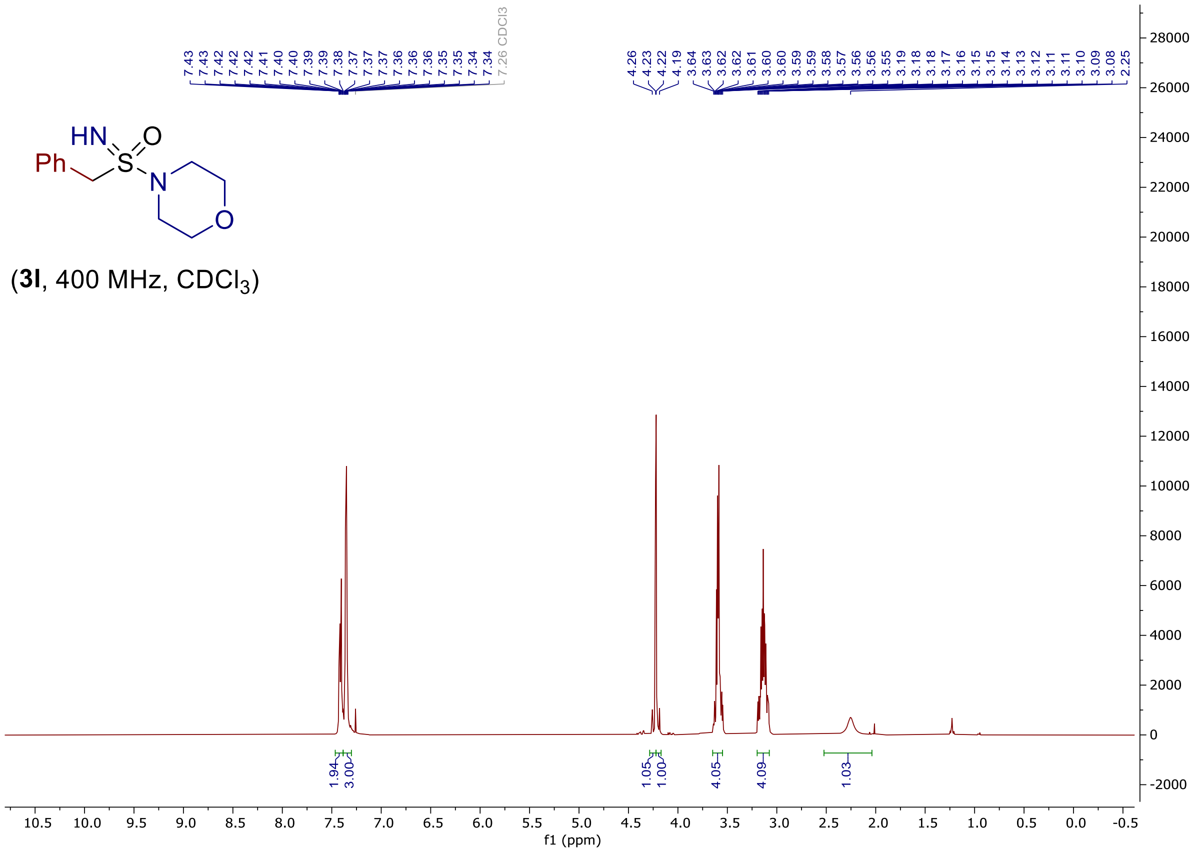

(3l, 400 MHz, CDCl3)
7.43
7.43
7.42
7.42
7.42
7.41
7.40
7.40
7.39
7.39
7.38
7.37
7.37
7.37
7.36
7.36
7.35
7.35
7.34
7.34
7.26 CDCl3
4.26
4.23
4.22
4.19
3.64
3.63
3.62
3.62
3.61
3.60
3.60
3.59
3.59
3.58
3.57
3.56
3.56
3.55
3.19
3.18
3.18
3.17
3.16
3.15
3.15
3.14
3.13
3.12
3.11
3.11
3.10
3.09
3.08
2.25
1.94
3.00
1.05
1.00
4.05
4.09
1.03
f1 (ppm)

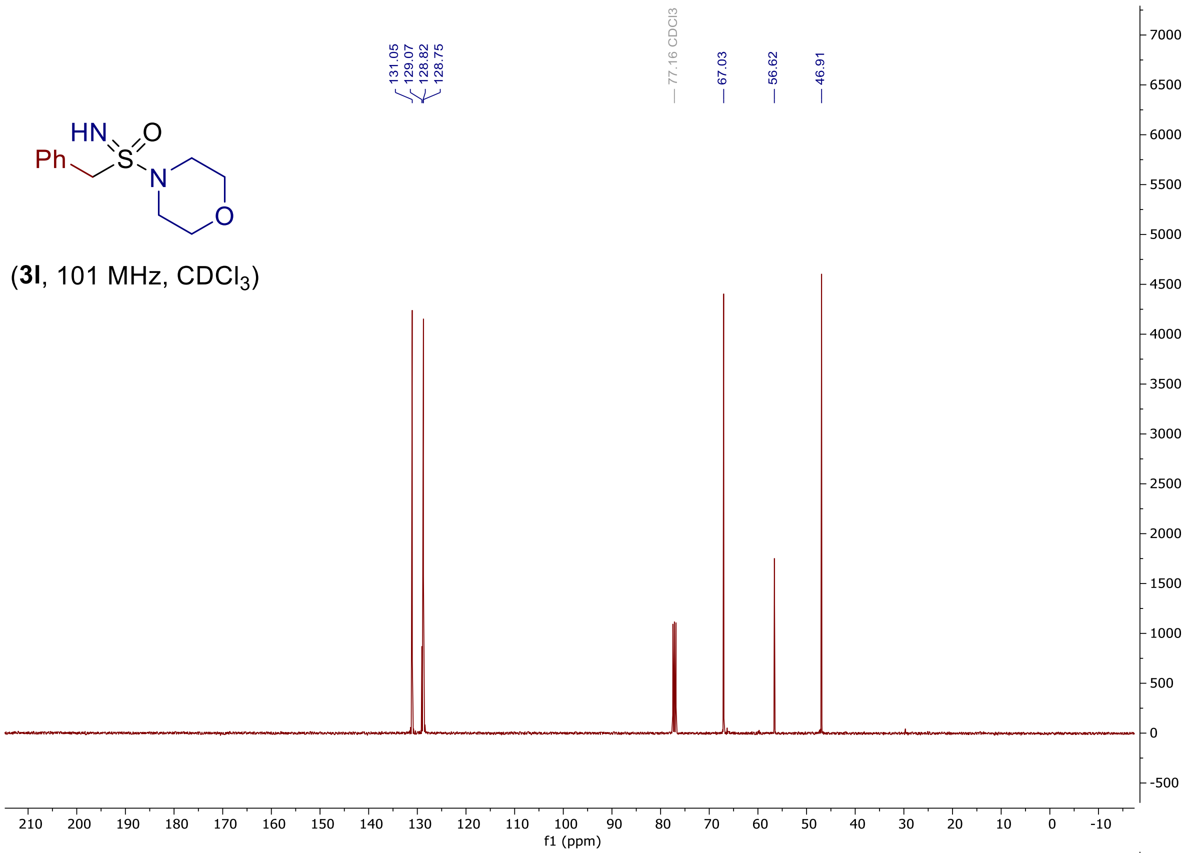
131.05
129.07
128.82
128.75
77.16 CDCl3
67.03
56.62
46.91
HN
O
Ph
S
N
O
(3l, 101 MHz, CDCl$_3$)
f1 (ppm)

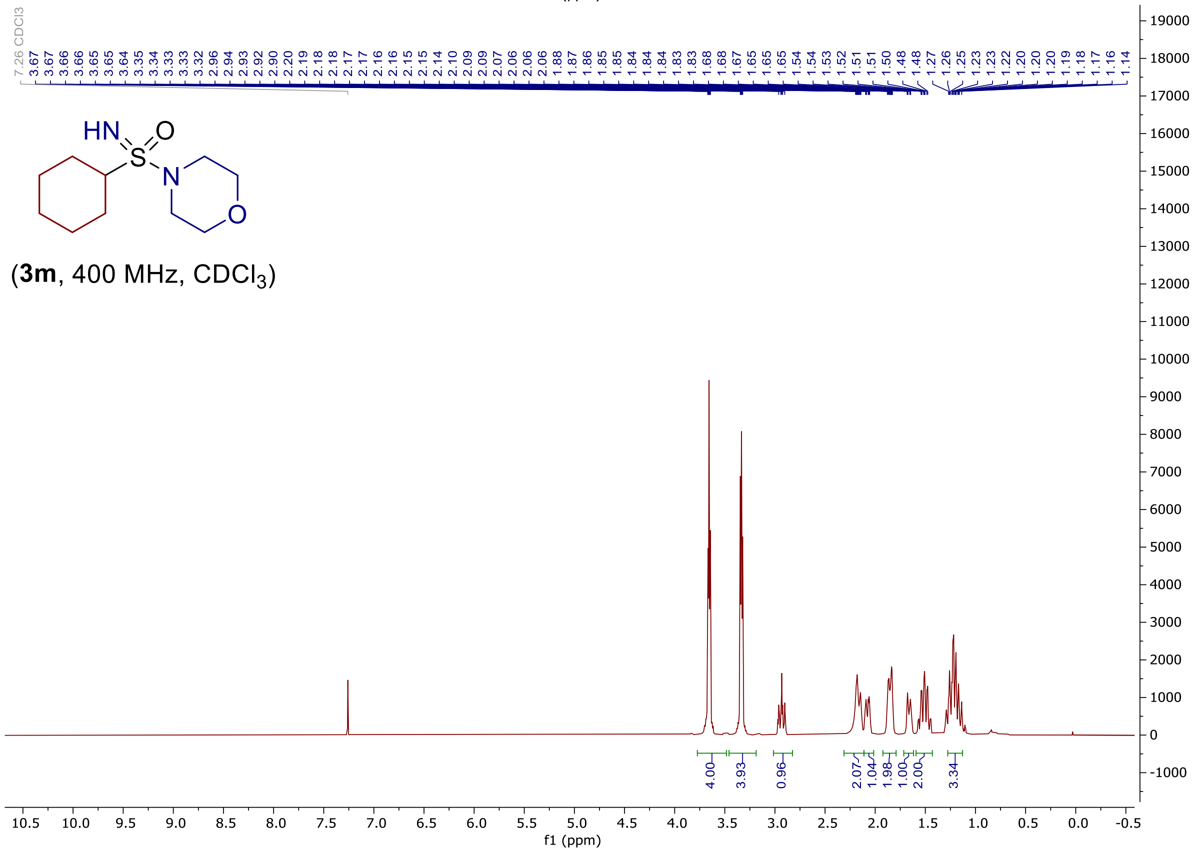
7.26 CDCl3
3.67 3.67 3.66 3.66 3.65 3.65 3.64 3.35 3.34 3.33 3.33 3.32 2.96 2.94 2.93 2.92 2.90 2.20 2.19 2.18 2.18 2.17 2.17 2.16 2.16 2.15 2.15 2.14 2.10 2.09 2.09 2.07 2.06 2.06 2.06 1.88 1.87 1.86 1.85 1.85 1.84 1.84 1.84 1.83 1.83 1.68 1.68 1.67 1.65 1.65 1.54 1.54 1.53 1.52 1.51 1.51 1.50 1.48 1.48 1.27 1.26 1.25 1.23 1.23 1.22 1.22 1.20 1.20 1.19 1.18 1.17 1.16 1.14
HN
O
S
N
O
(3m, 400 MHz, CDCl$_3$)
4.00
3.93
0.96
2.07
1.04
1.98
1.00
2.00
3.34
f1 (ppm)

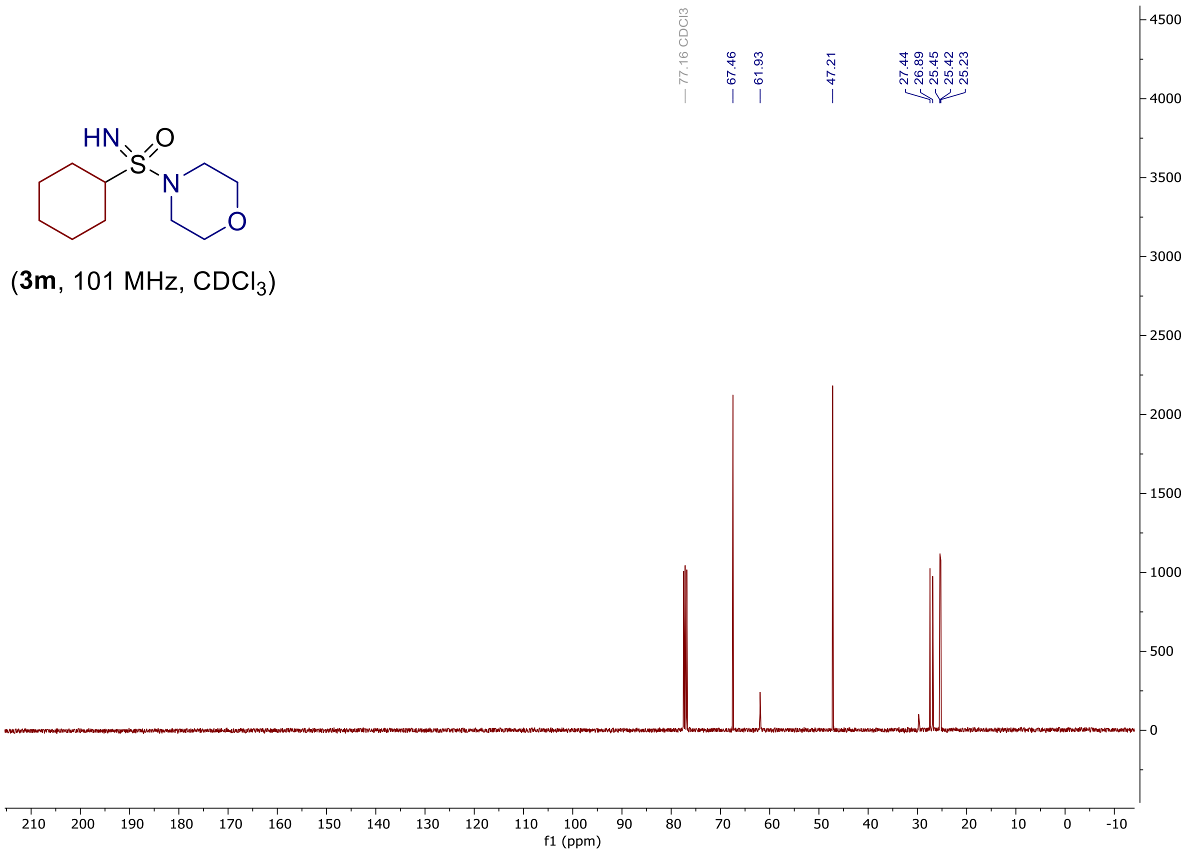

(**3m**, 101 MHz, $CDCl_3$)

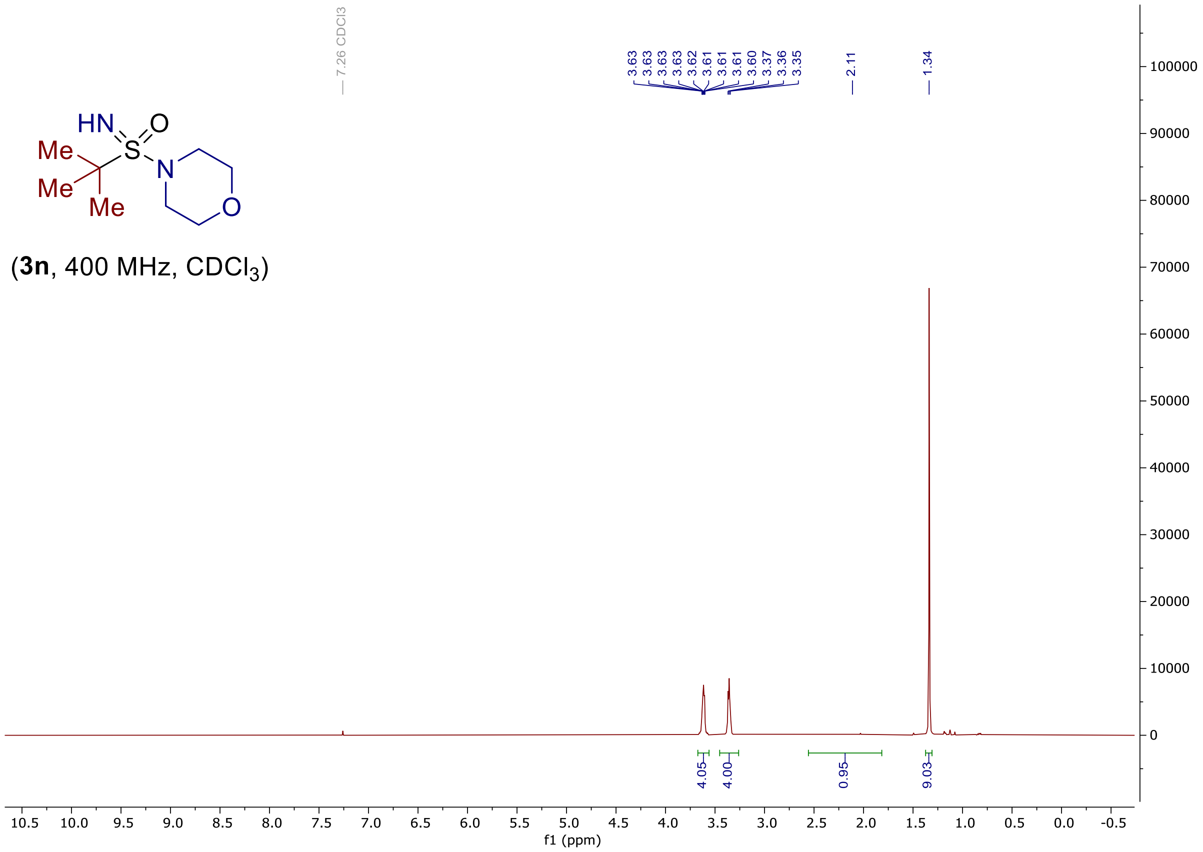

(**3n**, 400 MHz, $CDCl_3$)

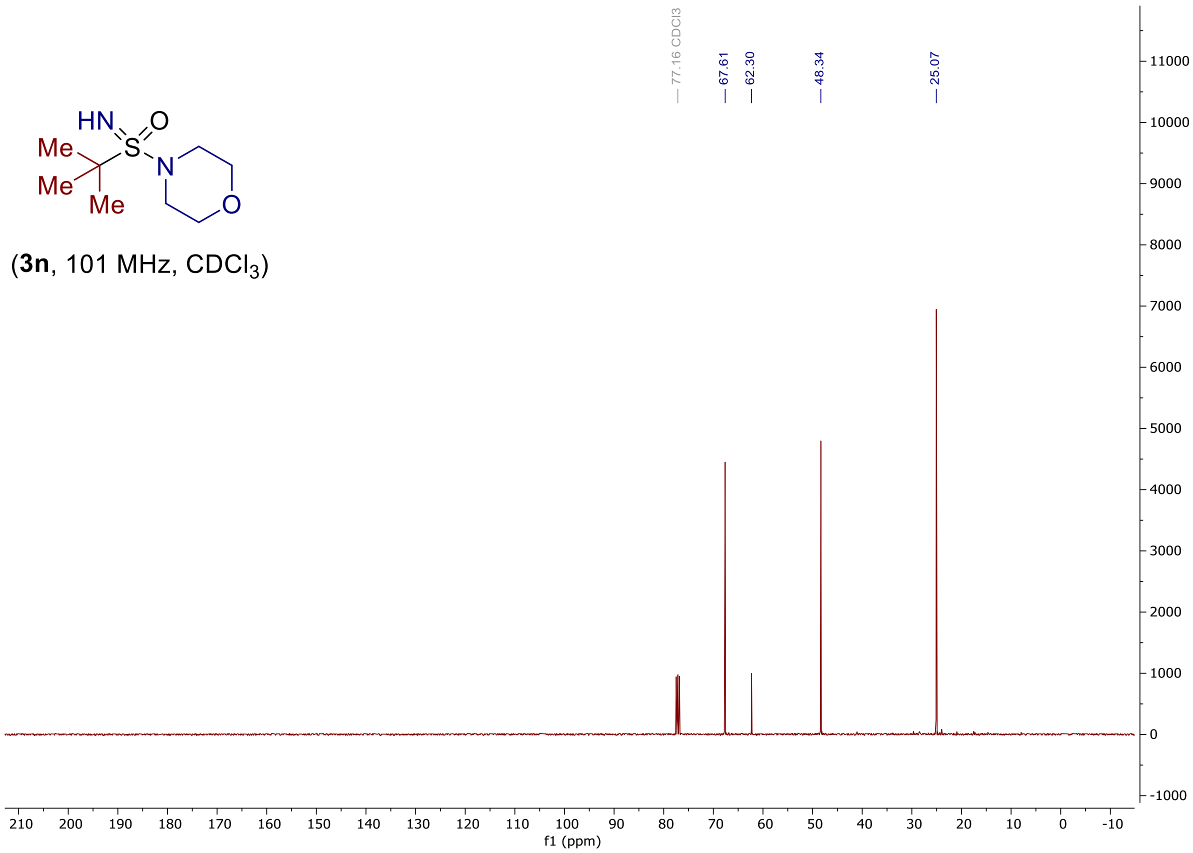

(**3n**, 101 MHz, $CDCl_3$)

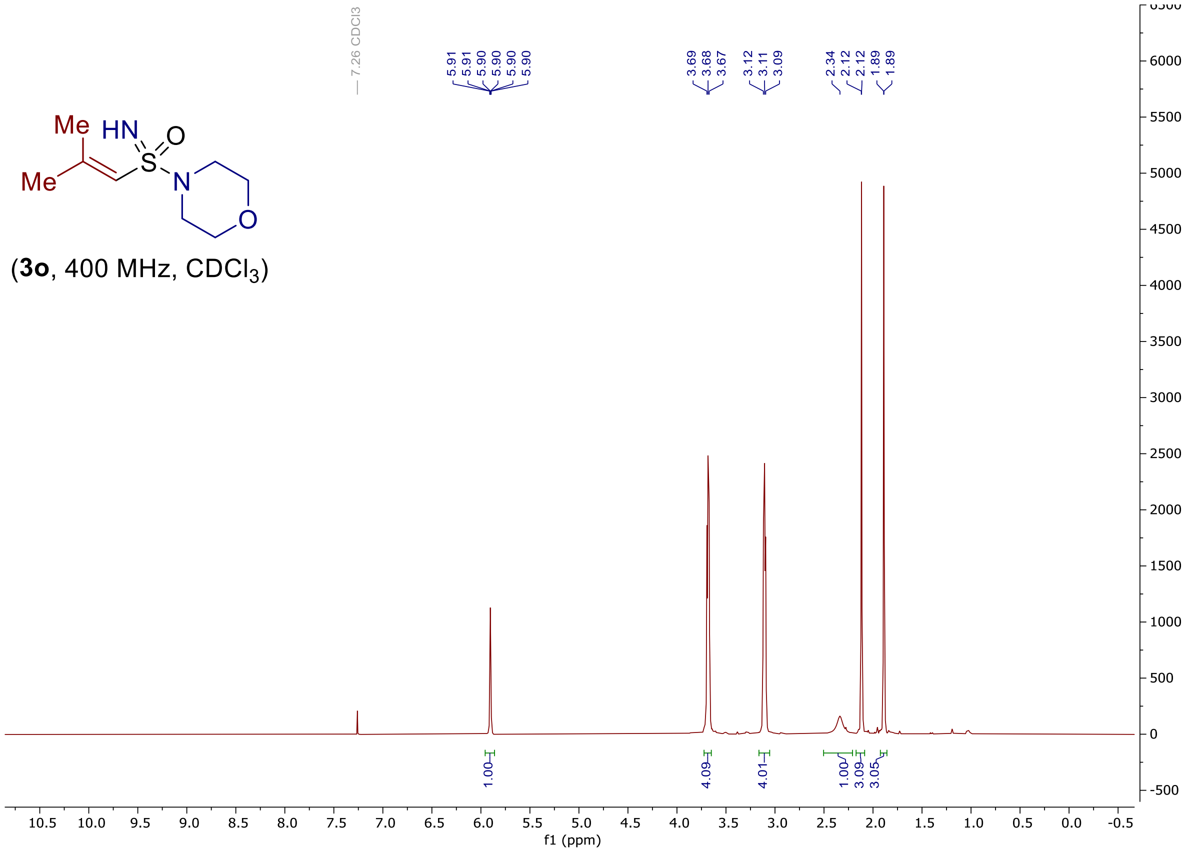

(**3o**, 400 MHz, $CDCl_3$)

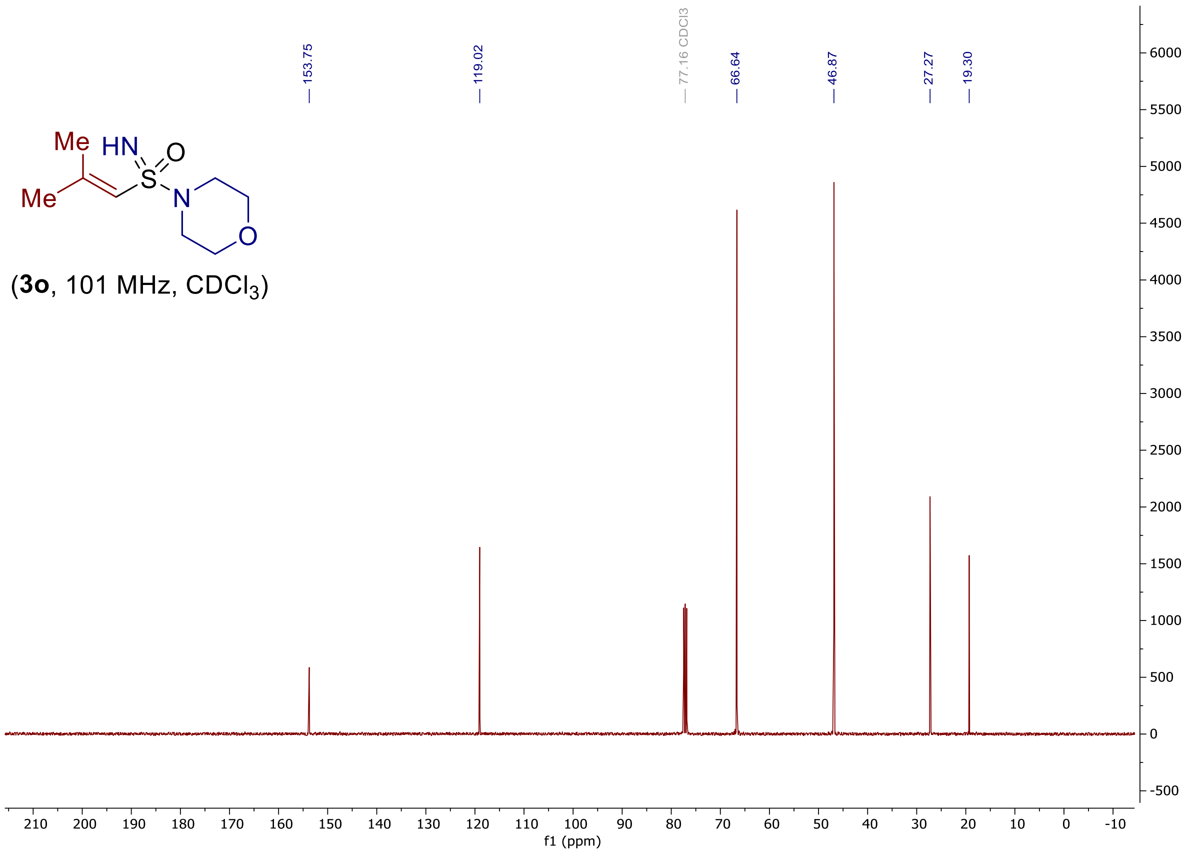

(**3o**, 101 MHz, $CDCl_3$)

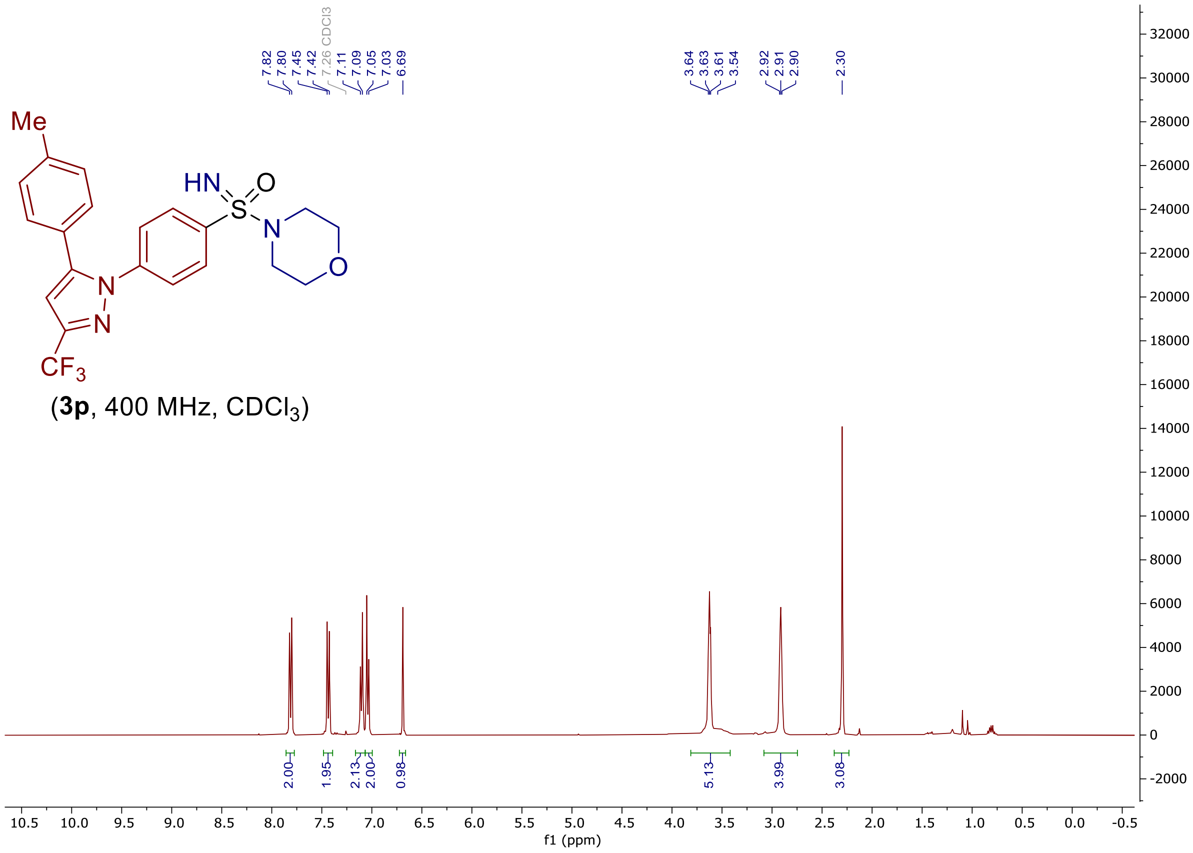

(**3p**, 400 MHz, $CDCl_3$)

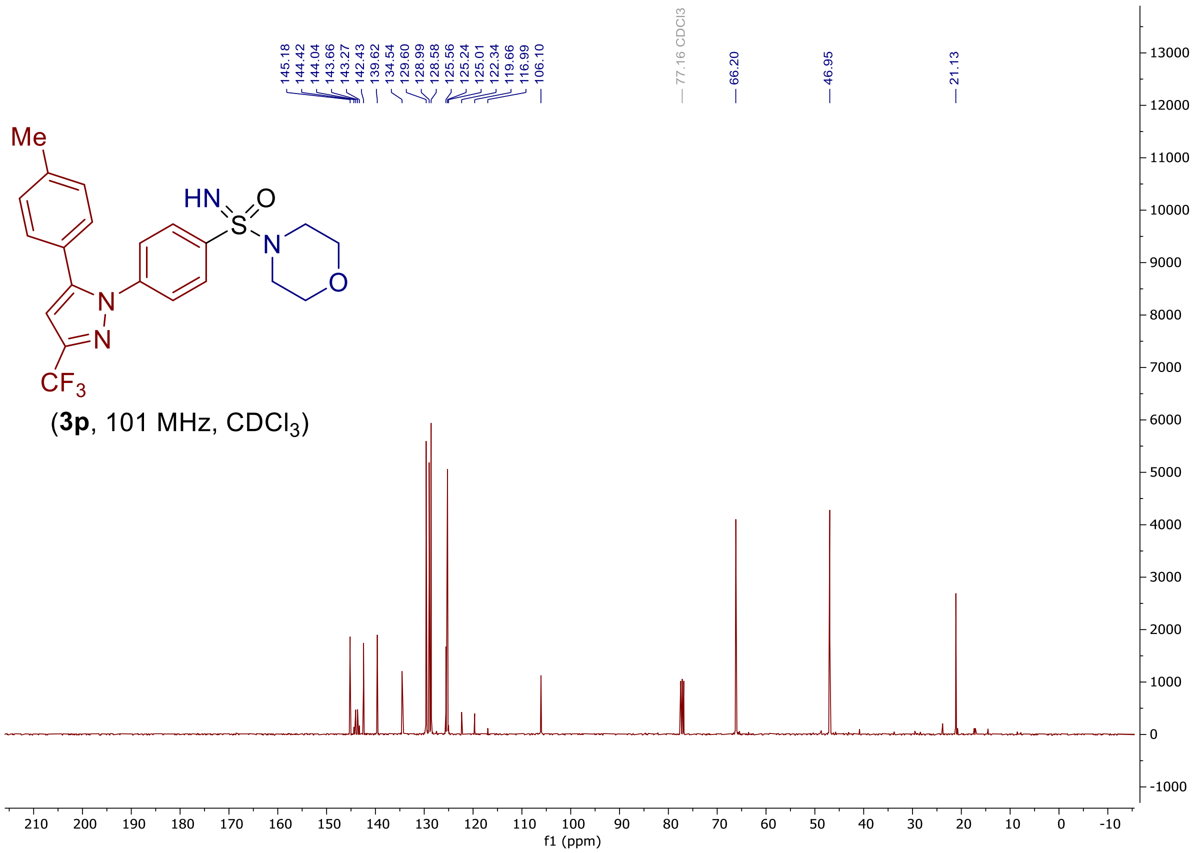

(**3p**, 101 MHz, $CDCl_3$)

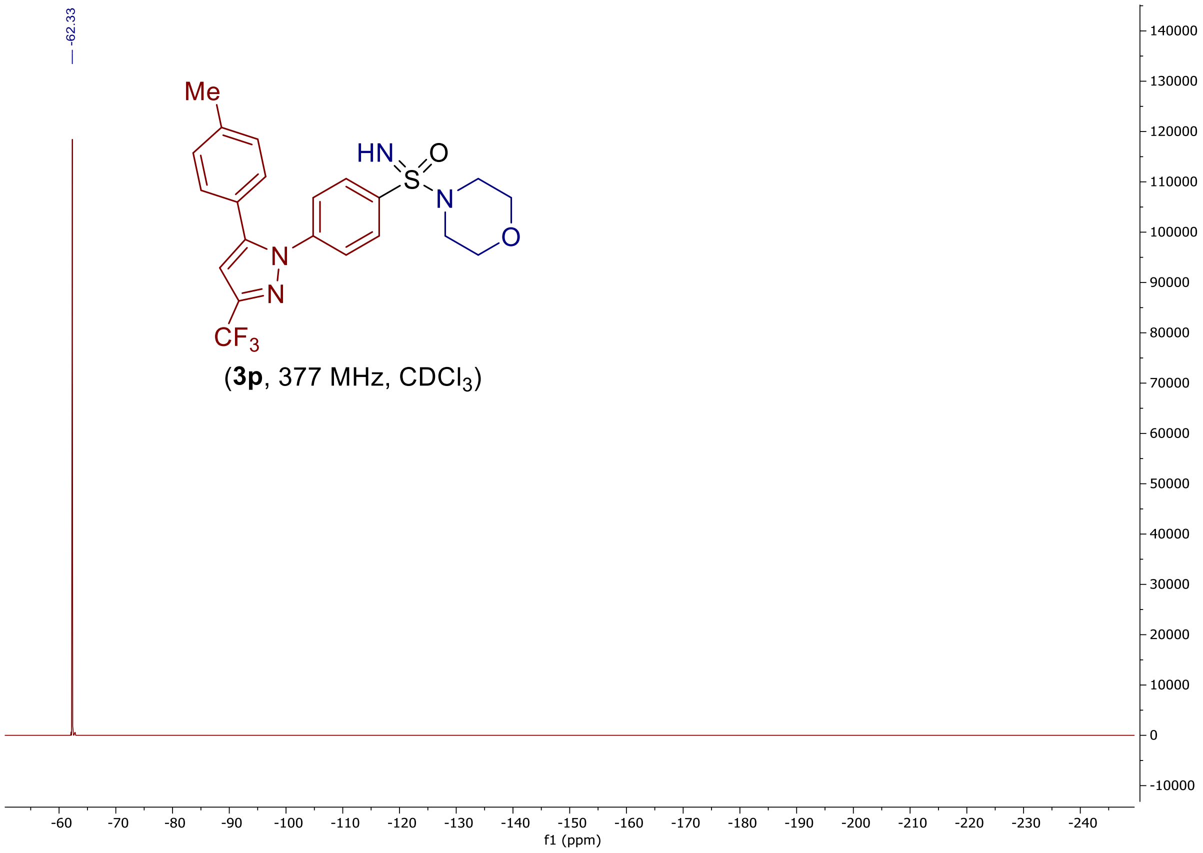

(**3p**, 377 MHz, $CDCl_3$)

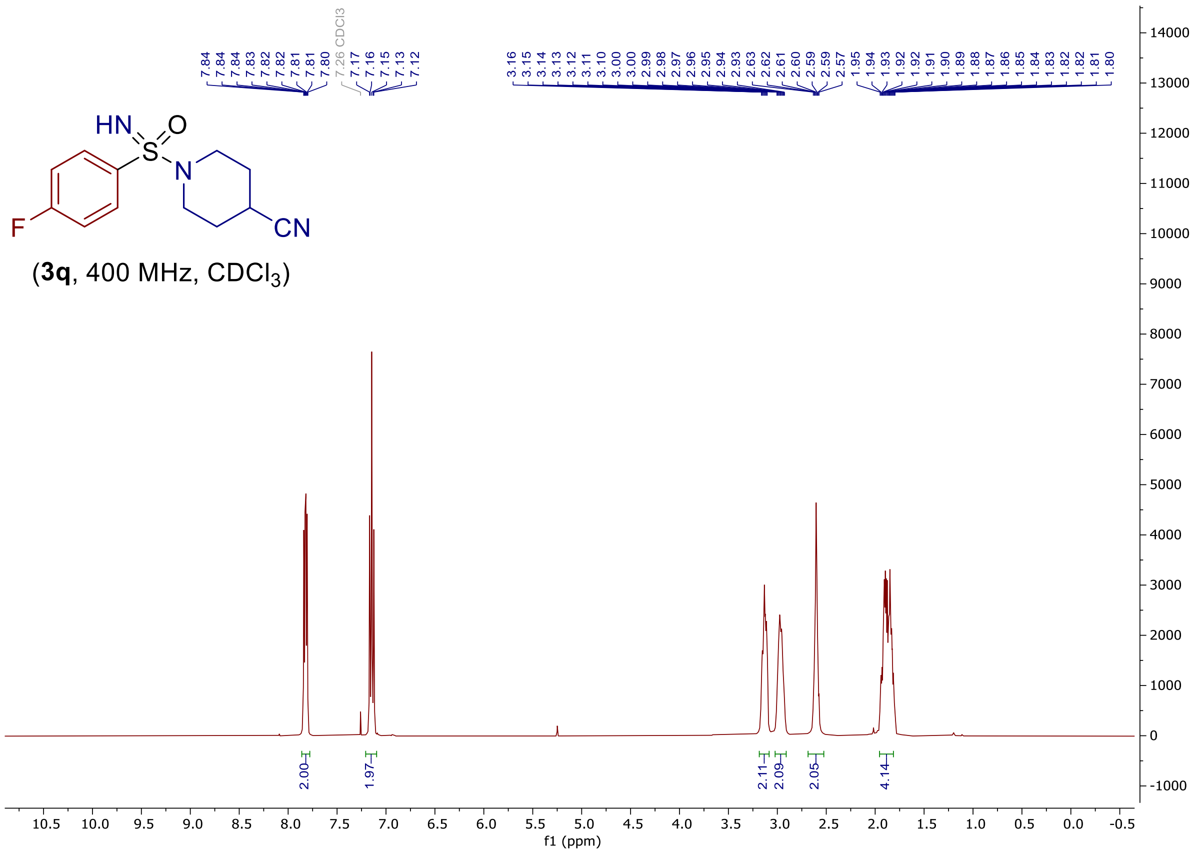
(3q, 400 MHz, CDCl3)
7.84
7.84
7.83
7.82
7.82
7.81
7.81
7.80
7.26 CDCl3
7.17
7.16
7.15
7.13
7.12
3.16
3.15
3.14
3.13
3.12
3.11
3.10
3.00
3.00
2.99
2.98
2.97
2.96
2.95
2.94
2.93
2.63
2.62
2.61
2.60
2.59
2.59
2.57
1.95
1.94
1.93
1.92
1.92
1.91
1.90
1.89
1.88
1.87
1.86
1.85
1.84
1.83
1.82
1.82
1.81
1.80
2.00
1.97
2.11
2.09
2.05
4.14
f1 (ppm)

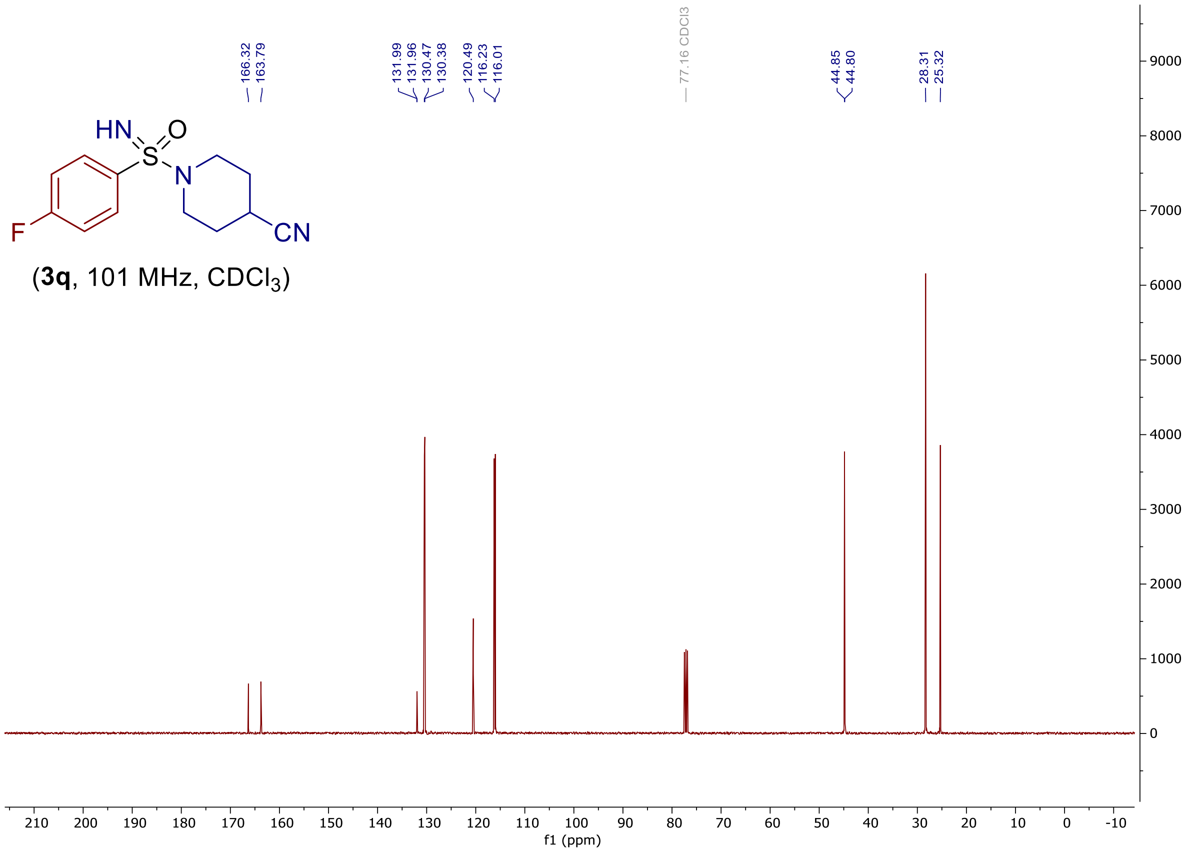
(3q, 101 MHz, CDCl3)
166.32
163.79
131.99
131.96
130.47
130.38
120.49
116.23
116.01
77.16 CDCl3
44.85
44.80
28.31
25.32
f1 (ppm)

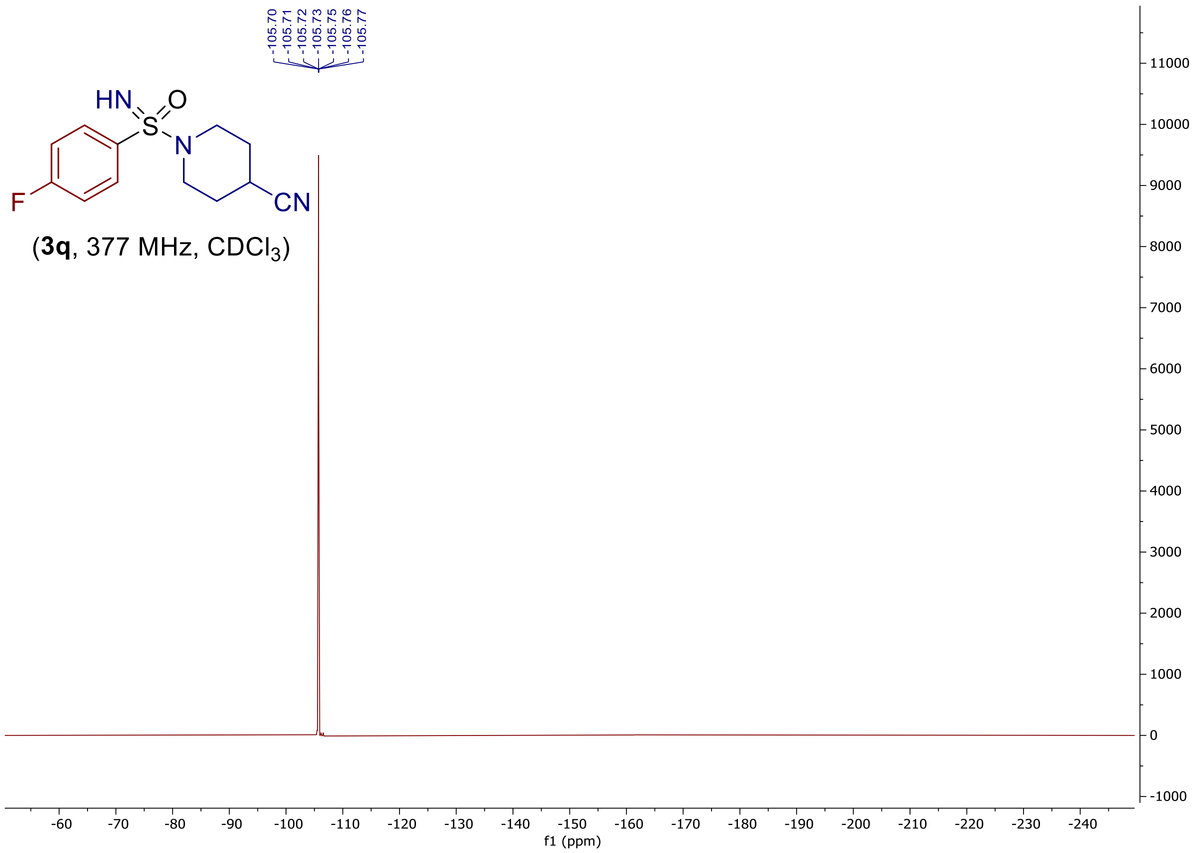
(3q, 377 MHz, CDCl3)
f1 (ppm)

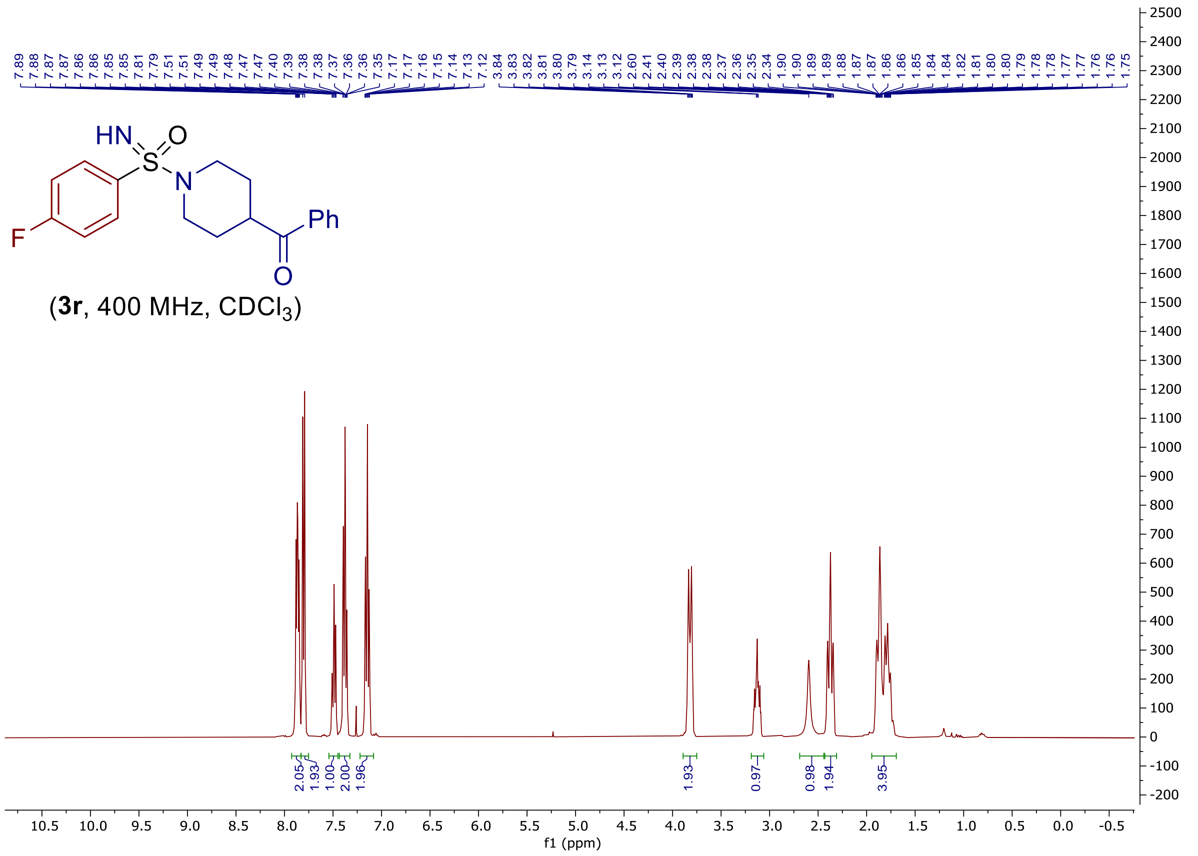
(3r, 400 MHz, CDCl3)
f1 (ppm)

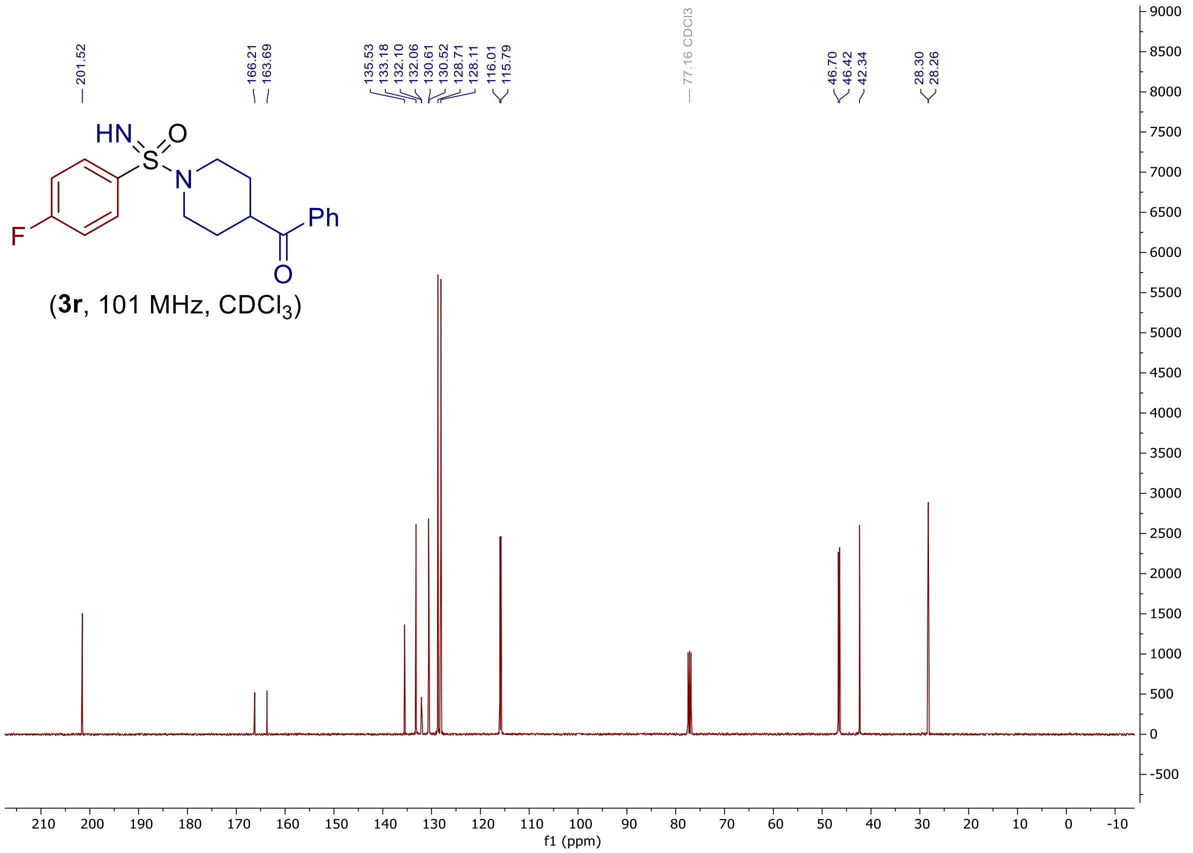

(**3r**, 101 MHz, $CDCl_3$)

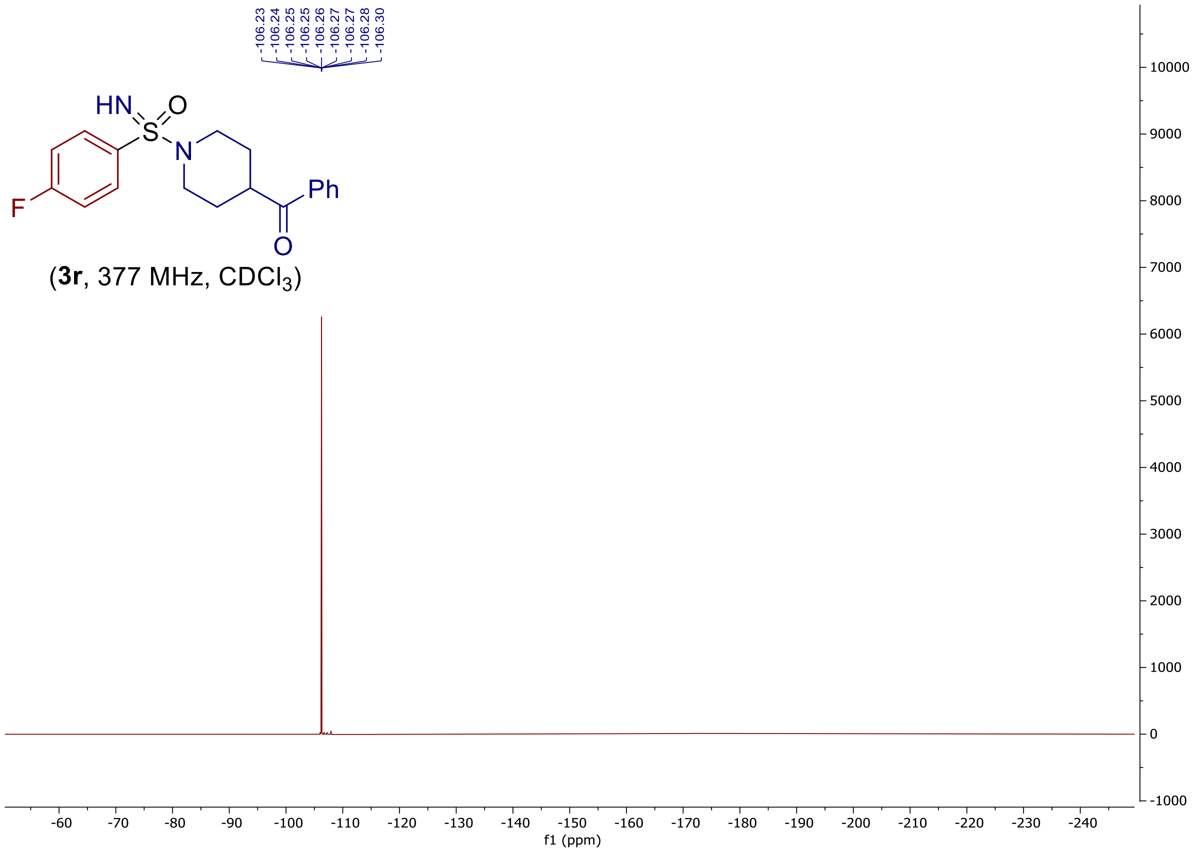

(**3r**, 377 MHz, $CDCl_3$)

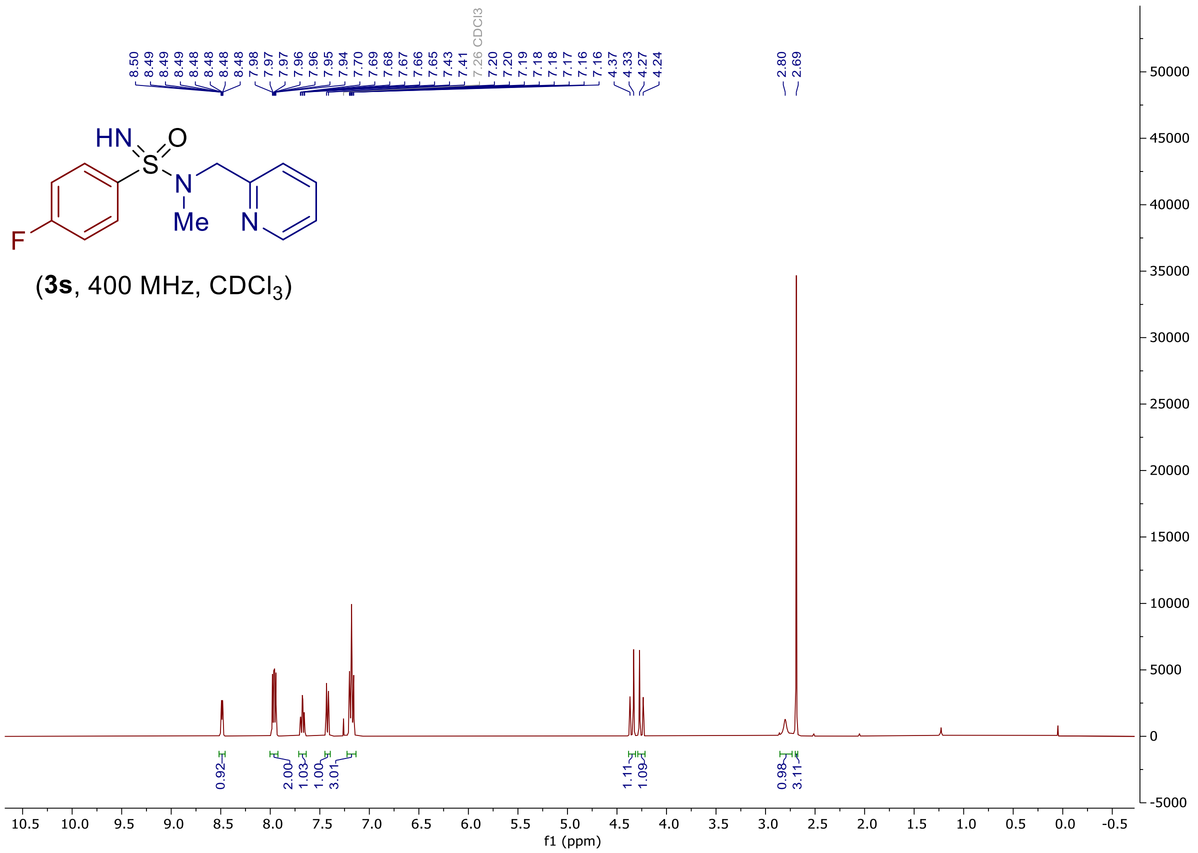

8.50 8.49 8.49 8.49 8.48 8.48 8.48 8.48 7.98 7.97 7.97 7.96 7.96 7.95 7.94 7.70 7.69 7.68 7.67 7.66 7.65 7.43 7.41 7.26 CDCl3 7.20 7.20 7.19 7.18 7.18 7.17 7.16 7.16 4.37 4.33 4.27 4.24
2.80 2.69
HN
S
O
N
Me
N
F
(3s, 400 MHz, CDCl3)
0.92 2.00 1.03 1.00 3.01 1.11 1.09 0.98 3.11
f1 (ppm)

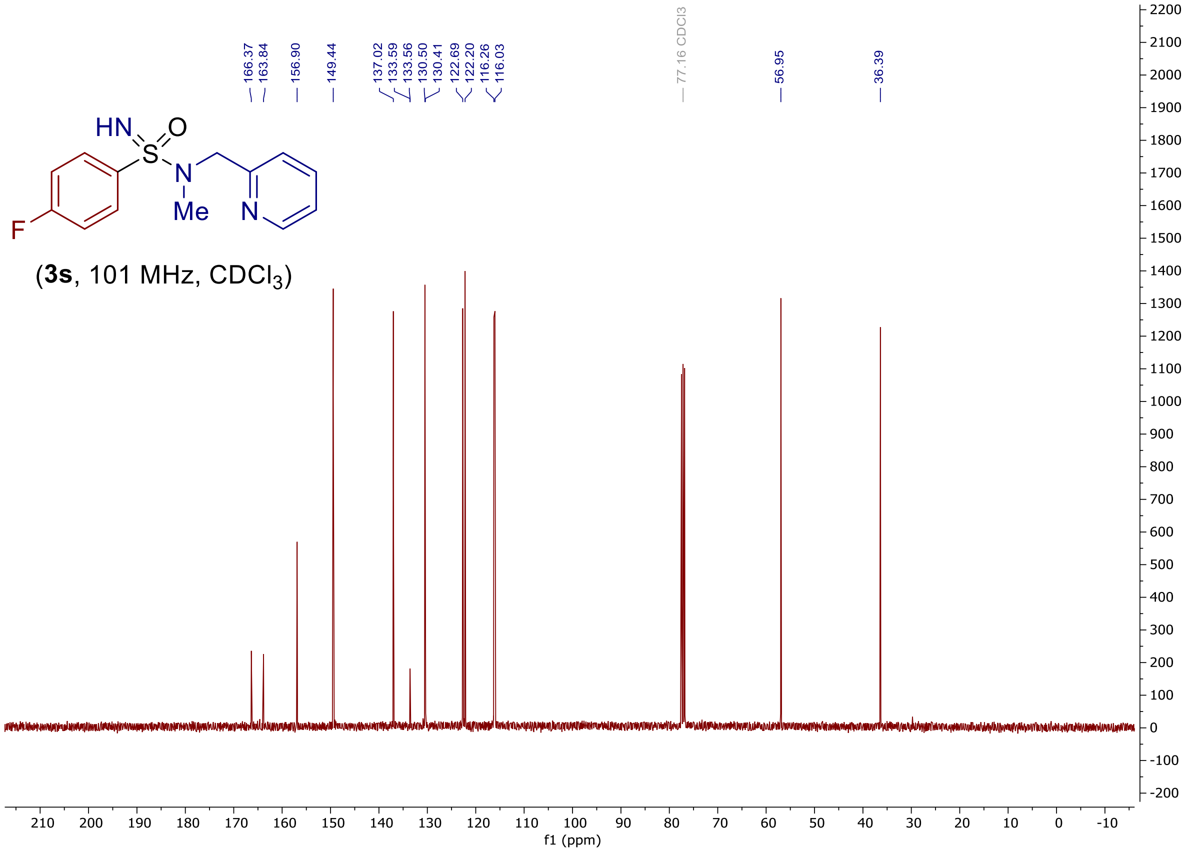

166.37 163.84 156.90 149.44 137.02 133.59 133.56 130.50 130.41 122.69 122.20 116.26 116.03
77.16 CDCl3
56.95
36.39
HN
S
O
N
Me
N
F
(3s, 101 MHz, CDCl3)
f1 (ppm)

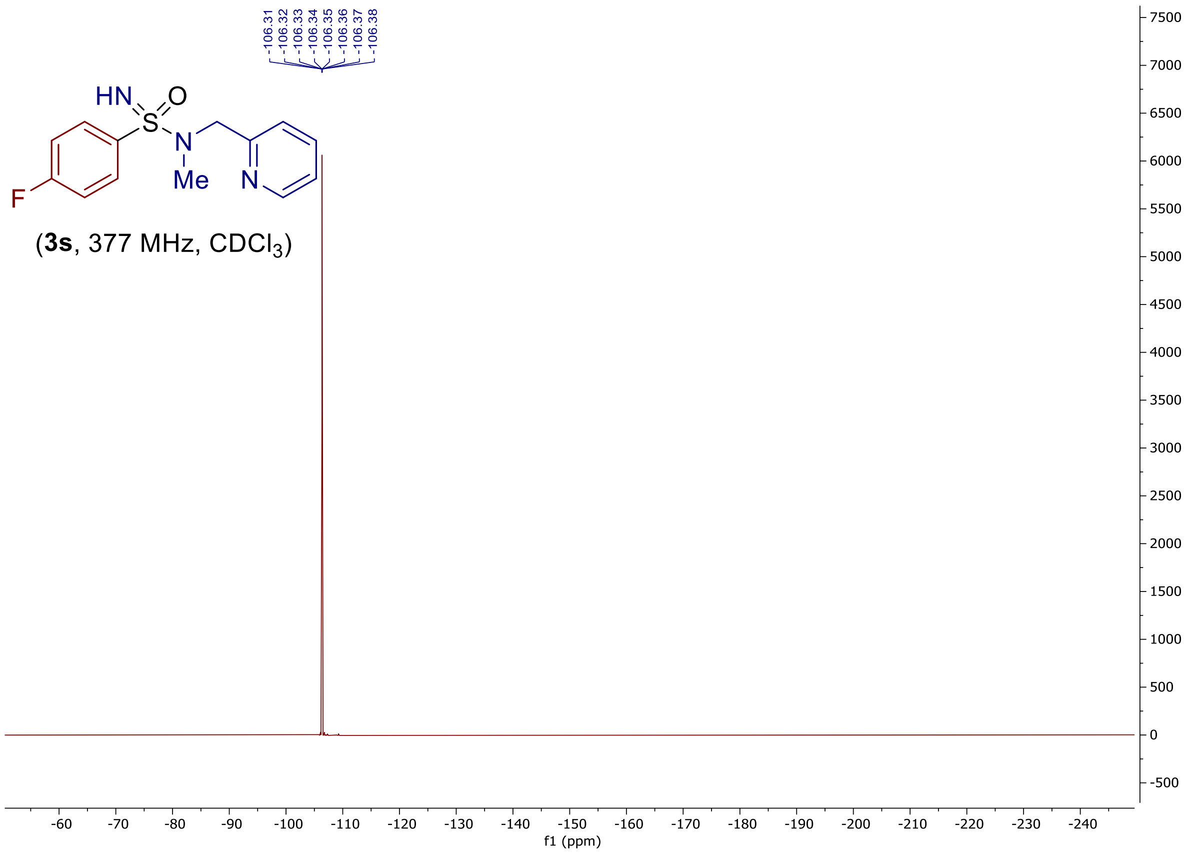

(3s, 377 MHz, CDCl3)
f1 (ppm)

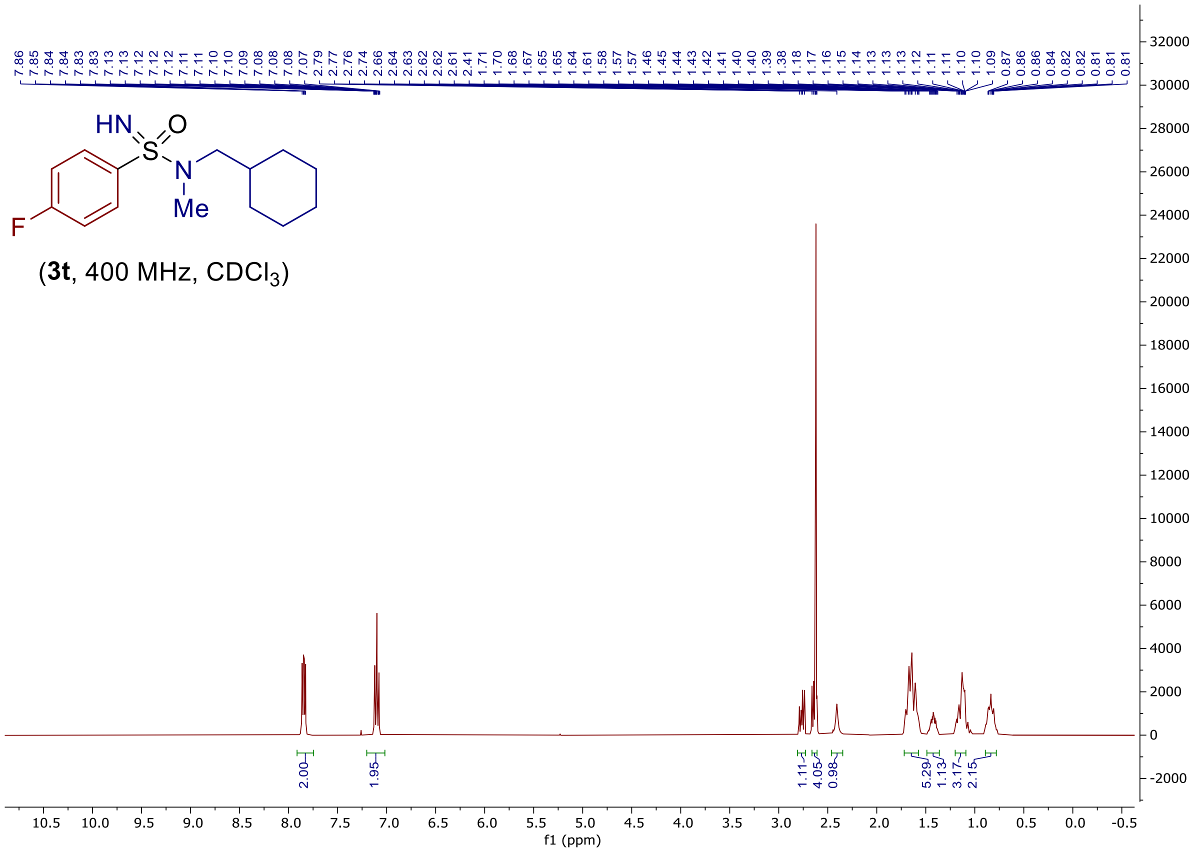

(3t, 400 MHz, CDCl3)
f1 (ppm)

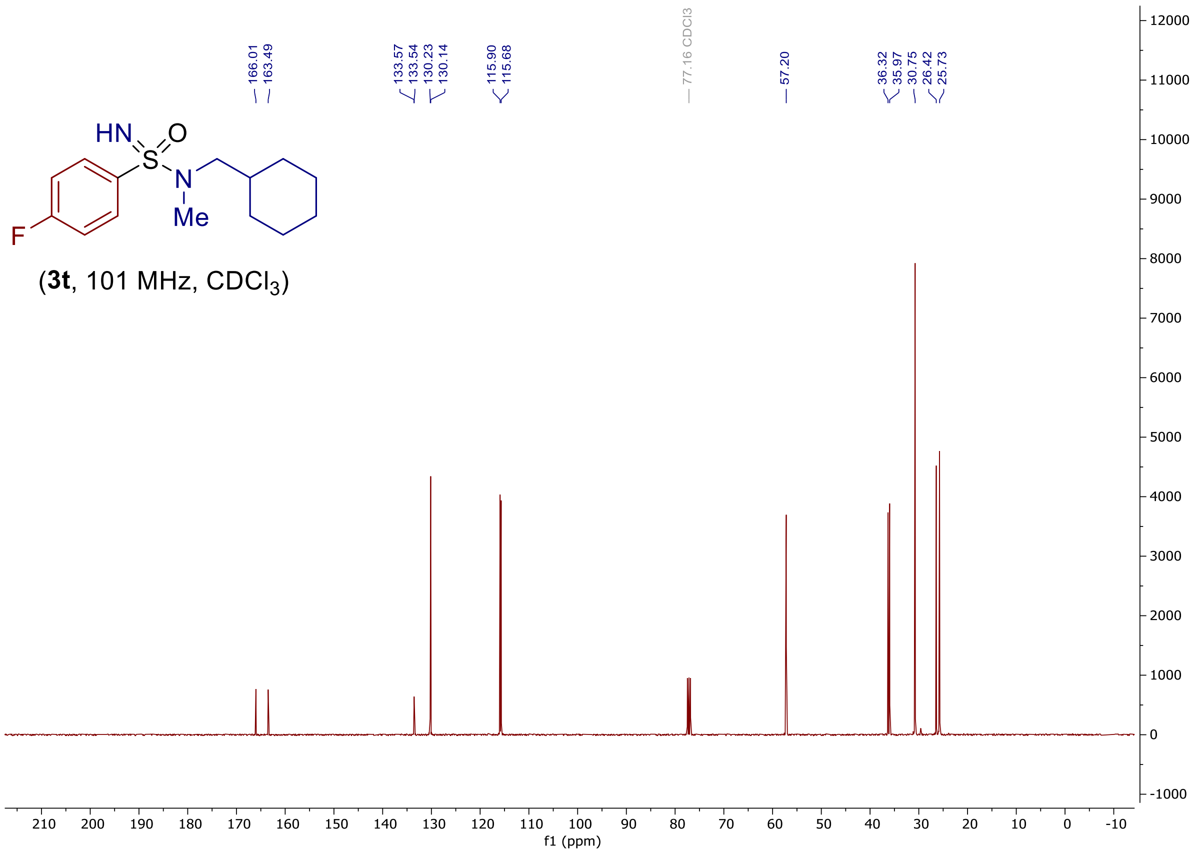

(**3t**, 101 MHz, $CDCl_3$)

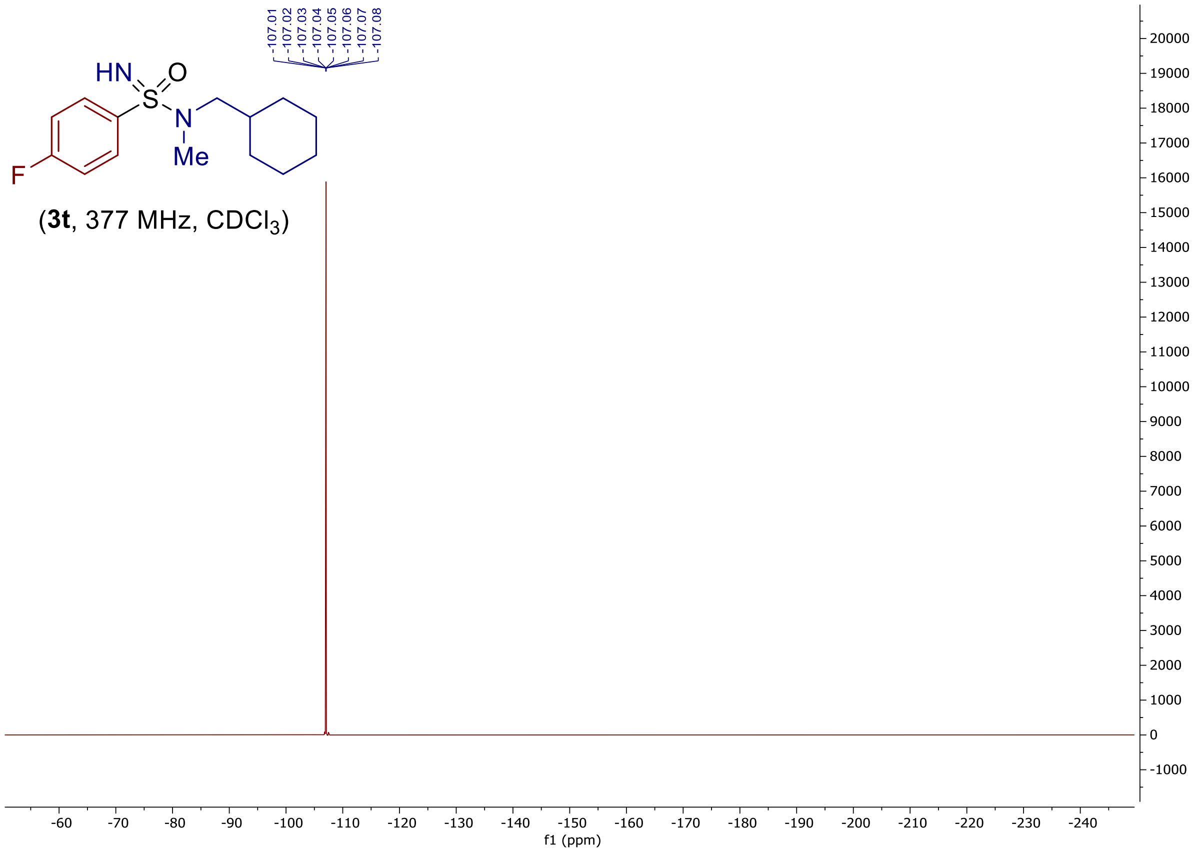

(**3t**, 377 MHz, $CDCl_3$)

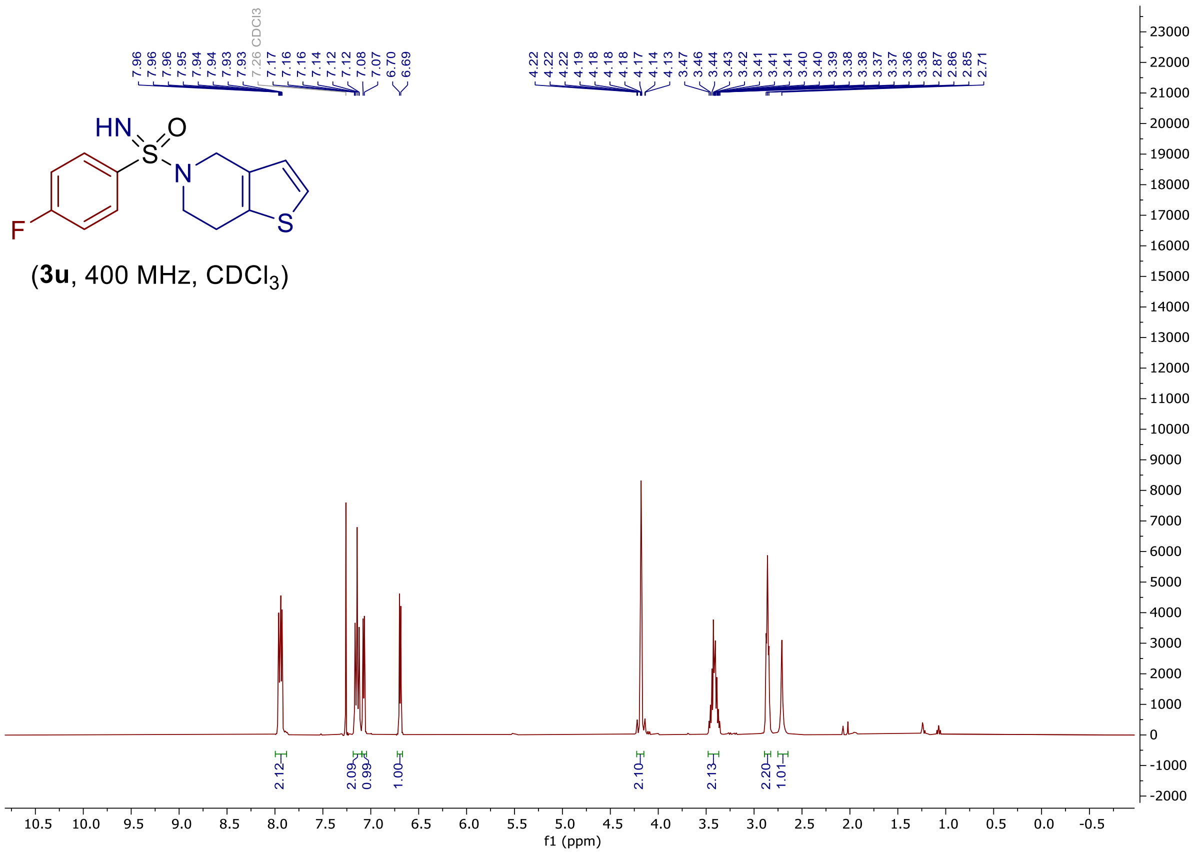
(3u, 400 MHz, CDCl3)
f1 (ppm)

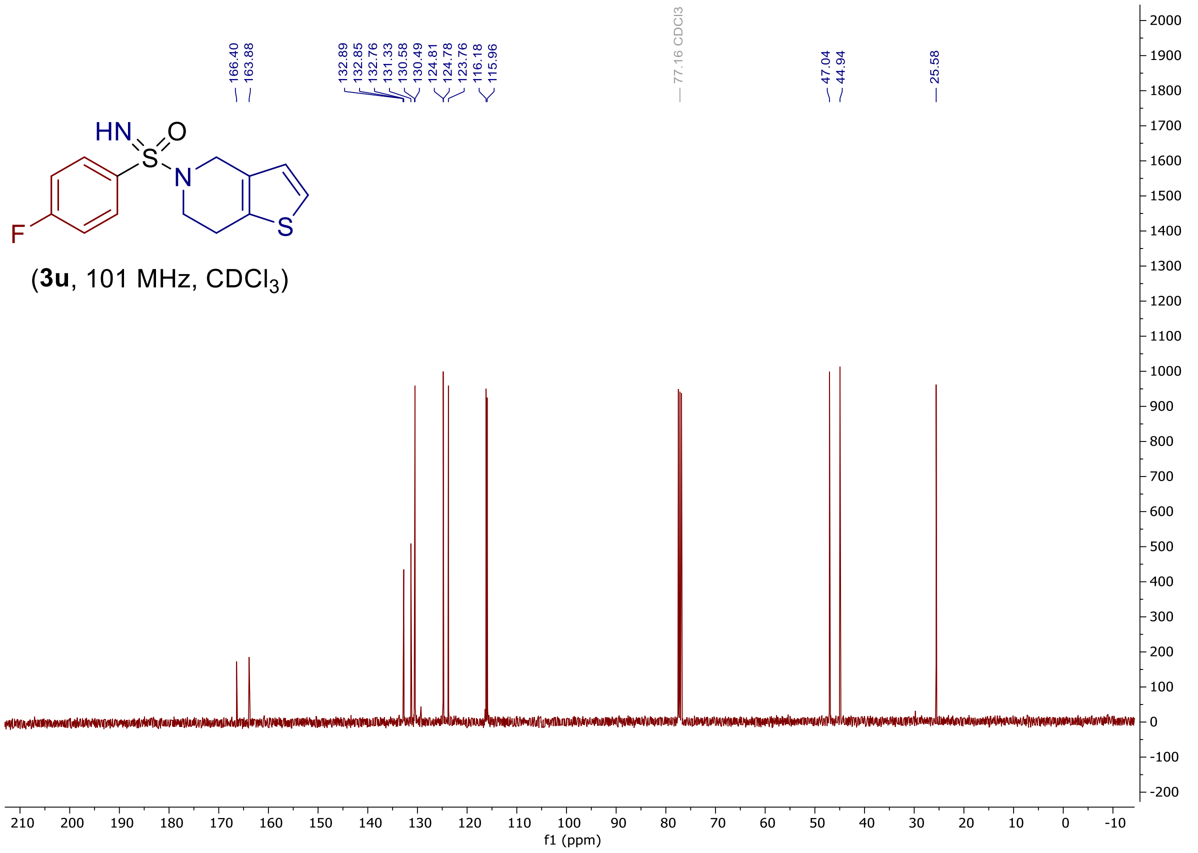
(3u, 101 MHz, CDCl3)
f1 (ppm)

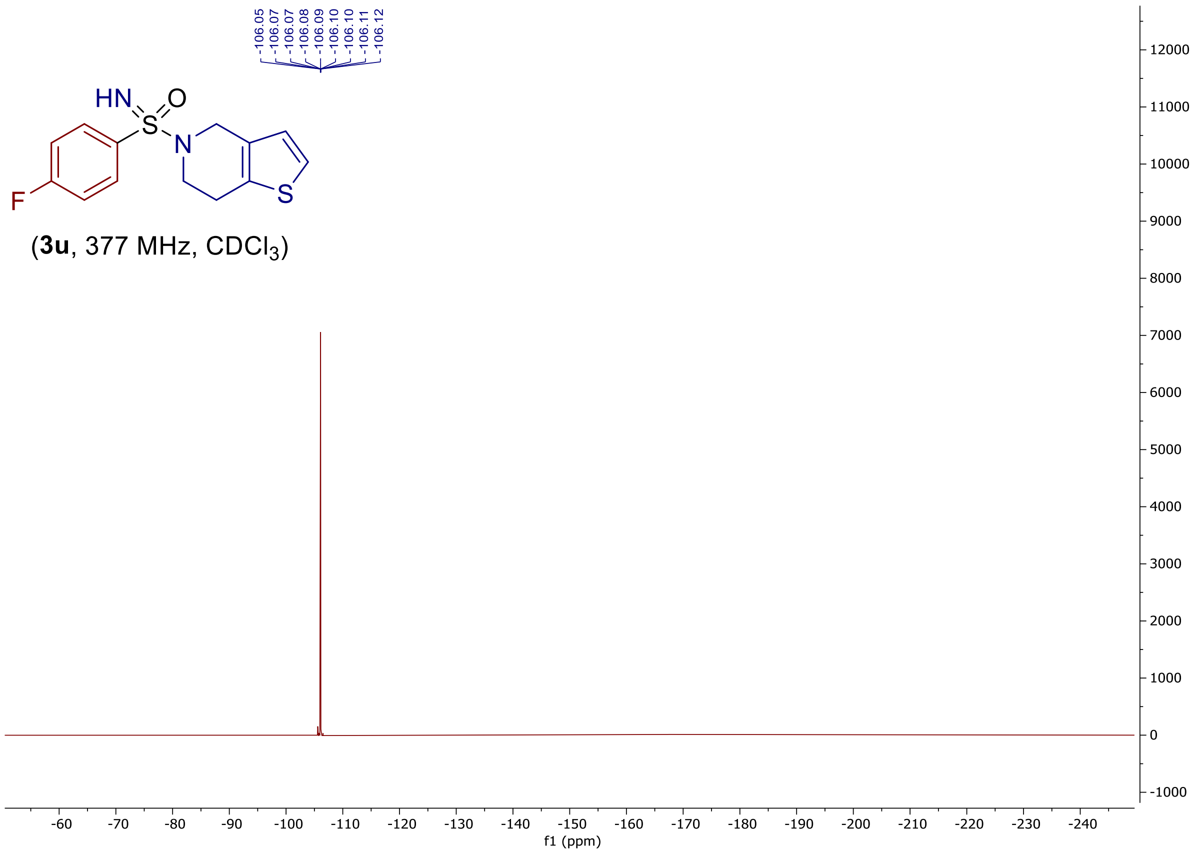

-106.05
-106.07
-106.07
-106.08
-106.09
-106.10
-106.10
-106.11
-106.12
HN
O
S
N
S
F
(3u, 377 MHz, CDCl3)
f1 (ppm)

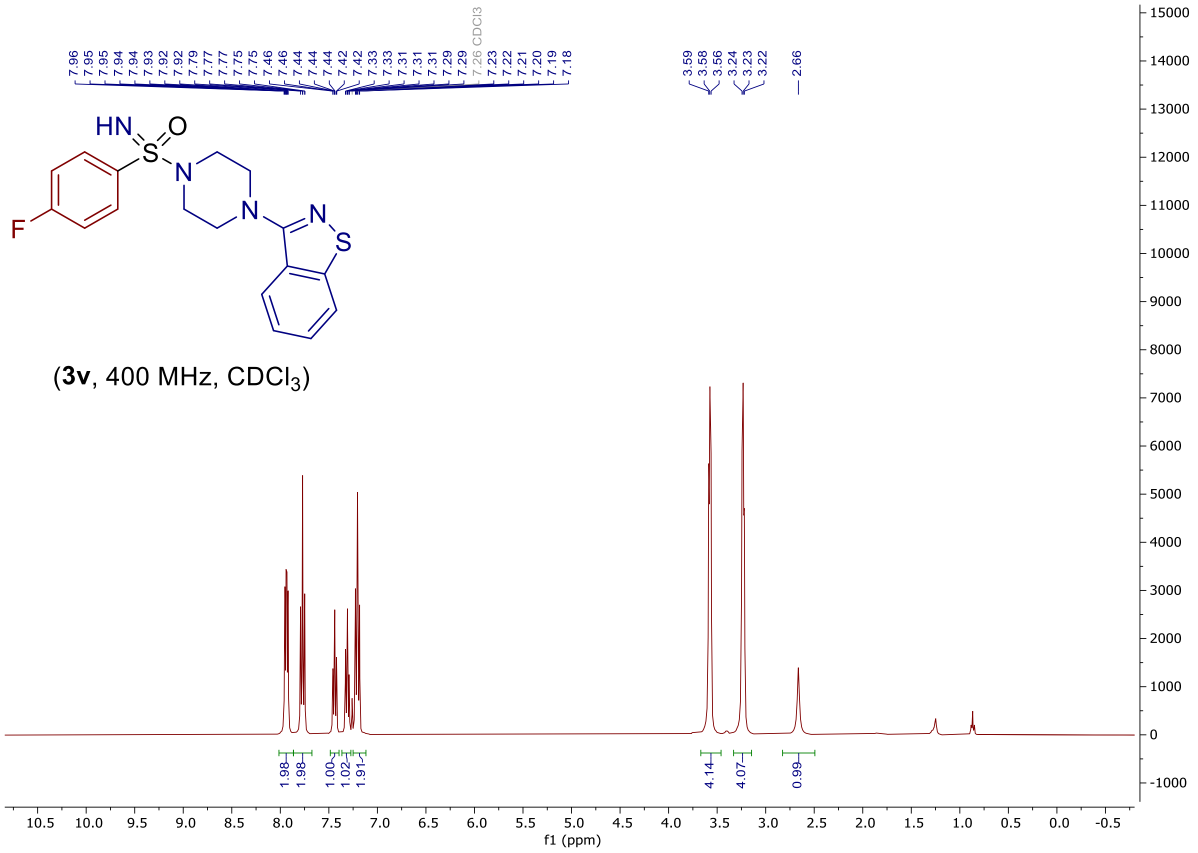

7.26 CDCl3
3.59
3.58
3.56
3.24
3.23
3.22
2.66
HN
O
S
N
N
N
S
F
(3v, 400 MHz, CDCl3)
1.98
1.98
1.00
1.02
1.91
4.14
4.07
0.99
f1 (ppm)

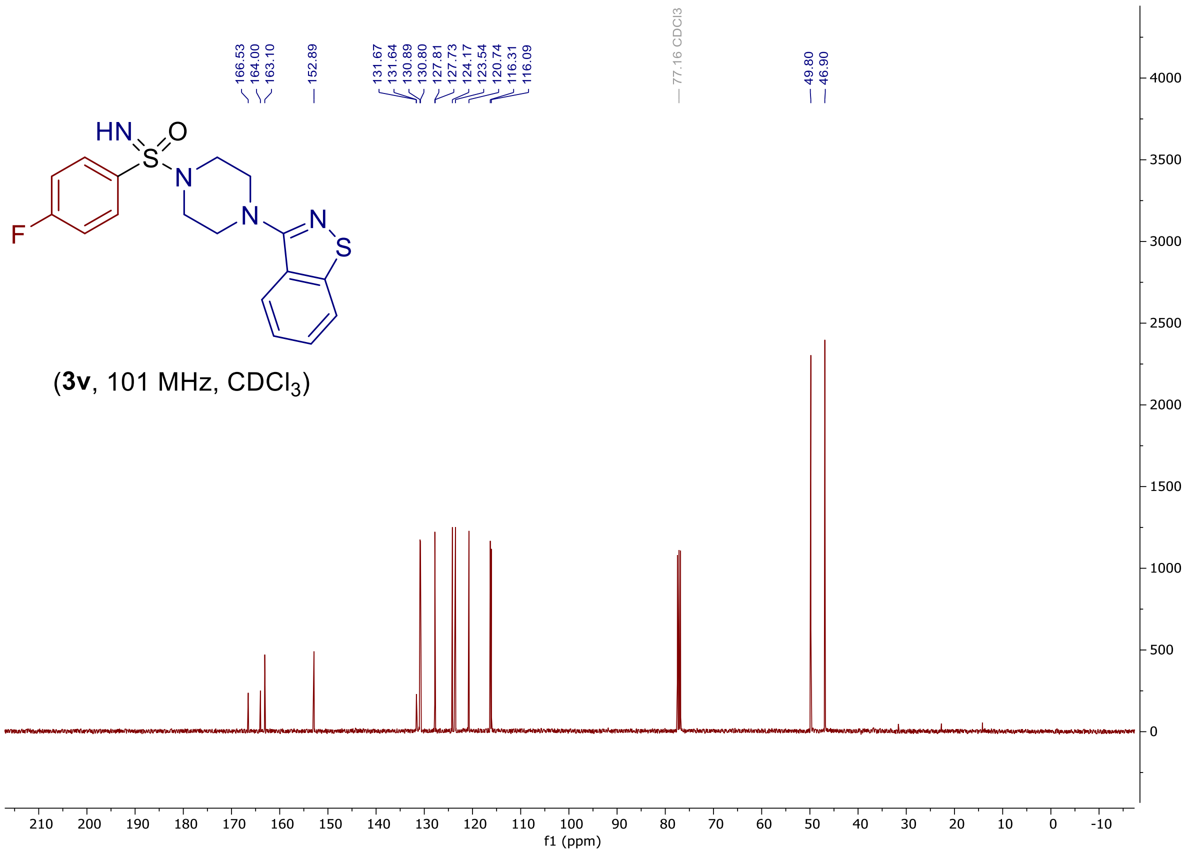

(**3v**, 101 MHz, $CDCl_3$)

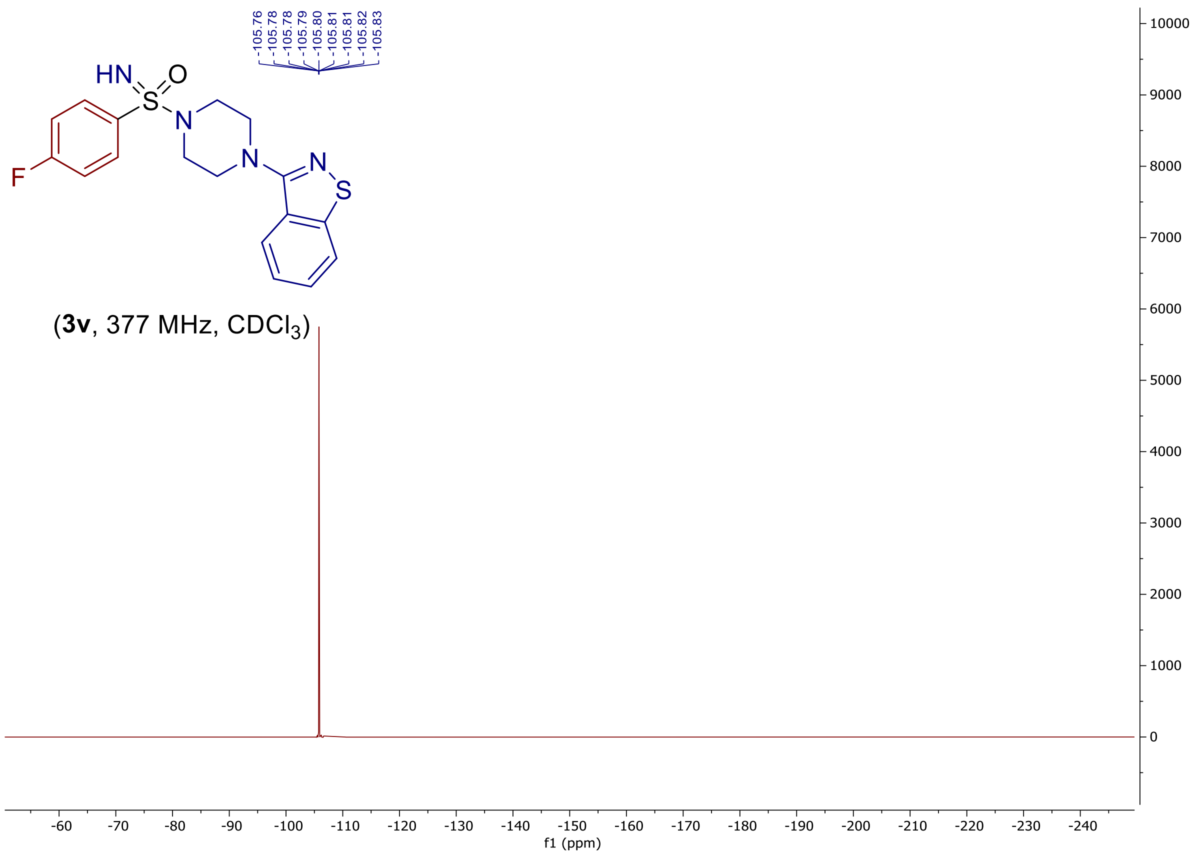

(**3v**, 377 MHz, $CDCl_3$)

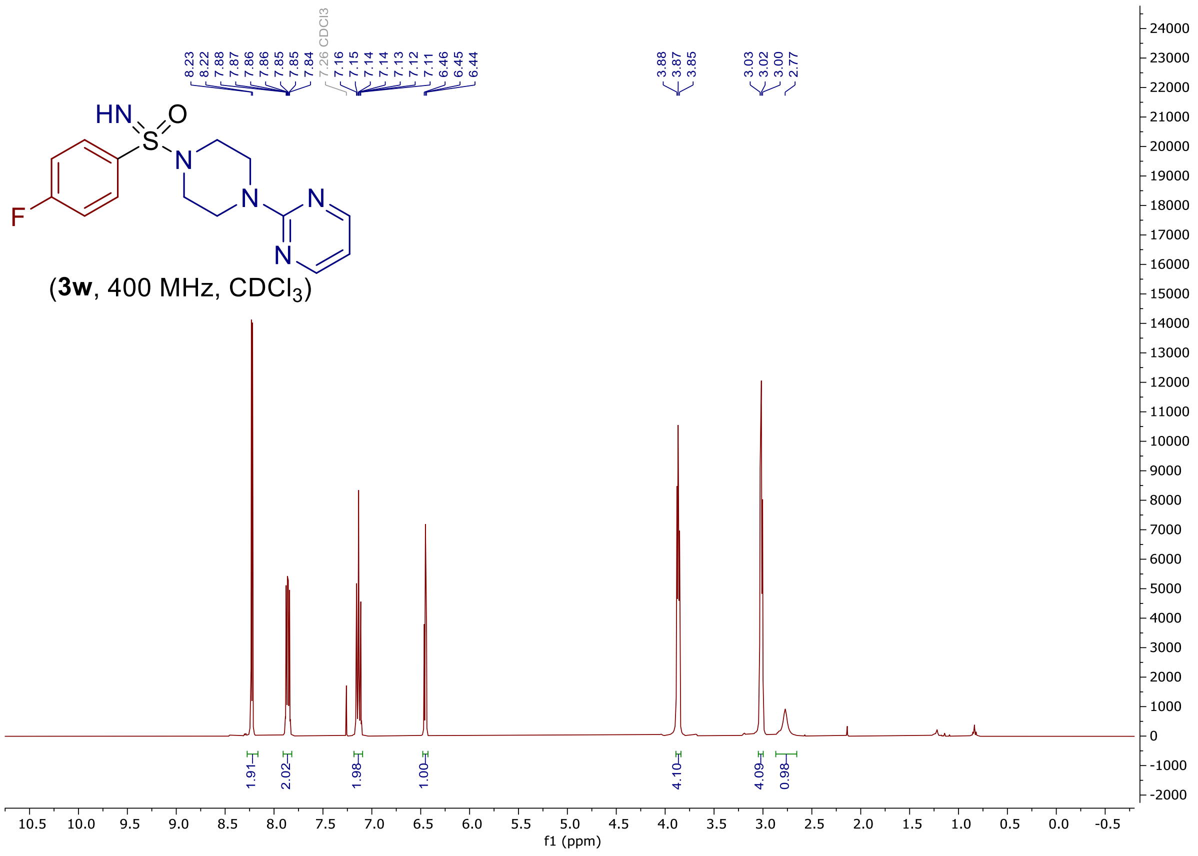

(**3w**, 400 MHz, $CDCl_3$)

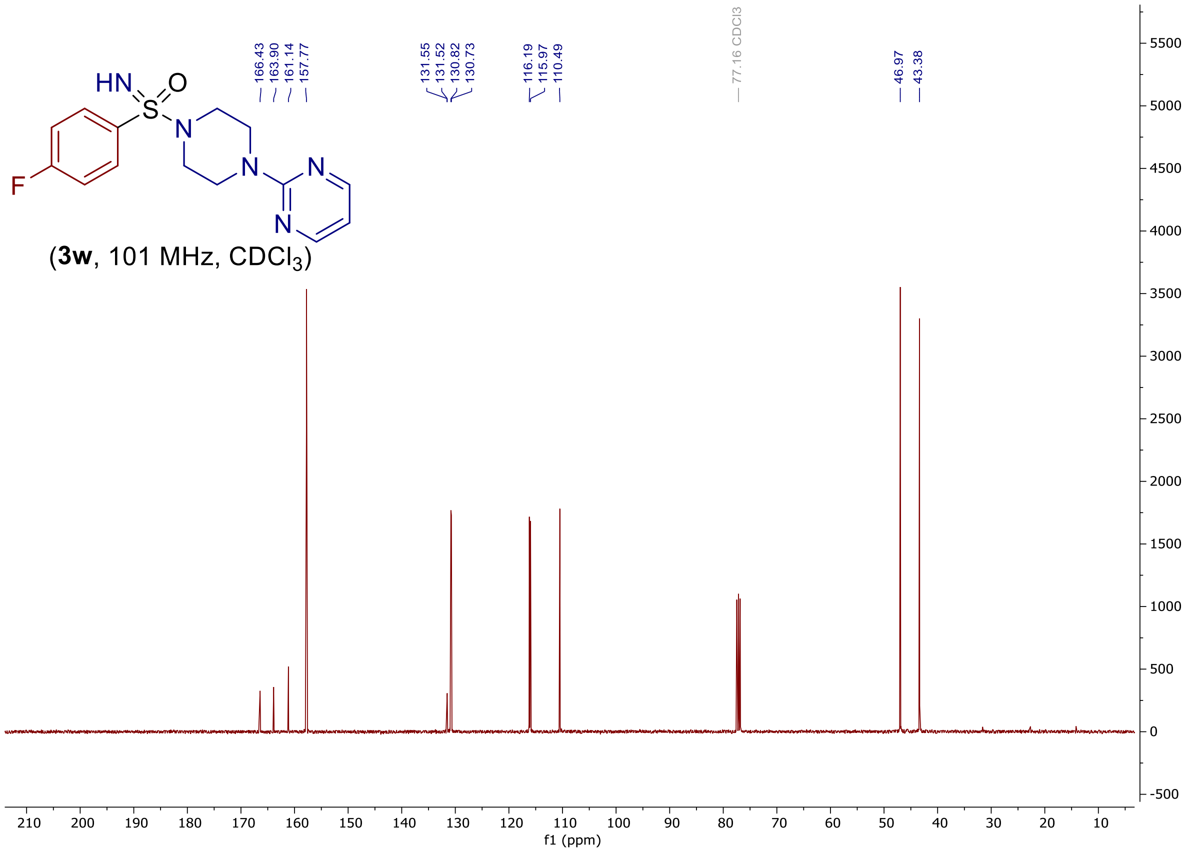

(**3w**, 101 MHz, $CDCl_3$)

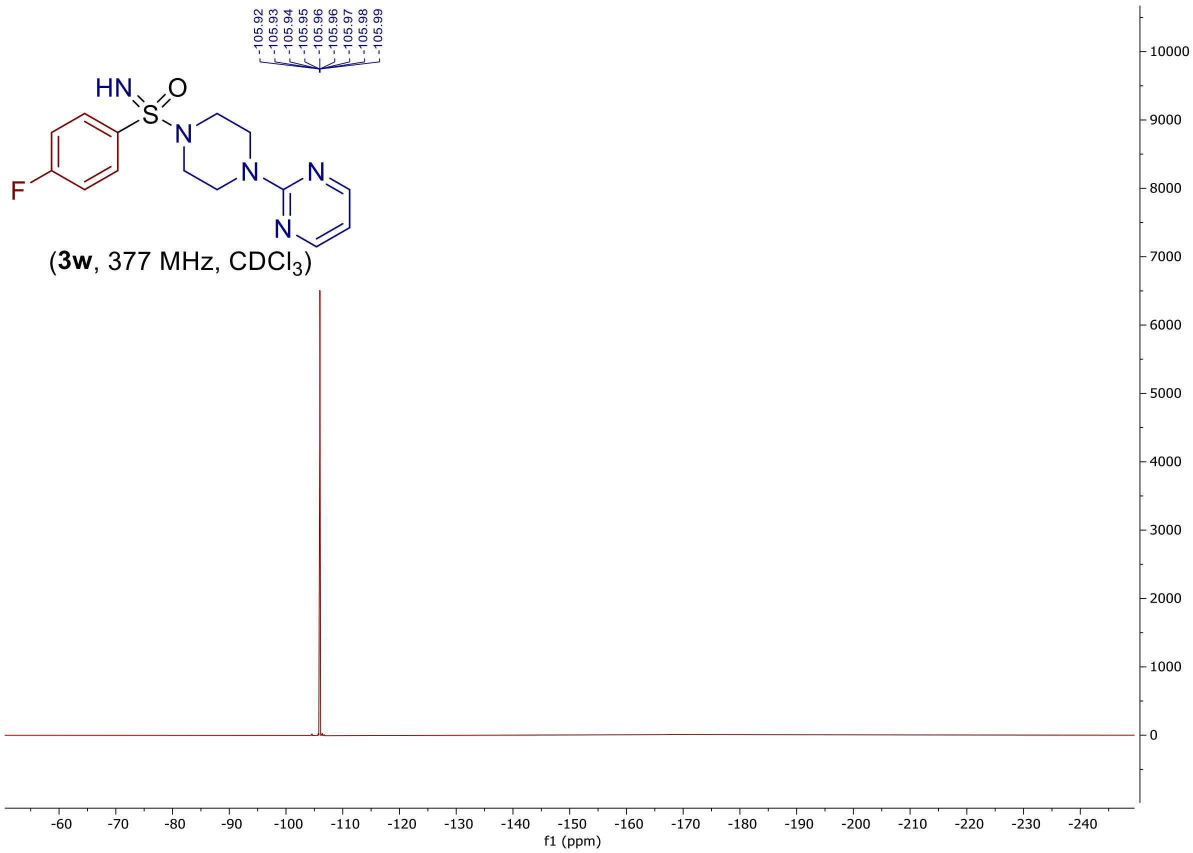
-105.92
-105.93
-105.94
-105.95
-105.96
-105.96
-105.97
-105.98
-105.99
HN
O
S
N
N
N
N
F
(3w, 377 MHz, CDCl3)
f1 (ppm)

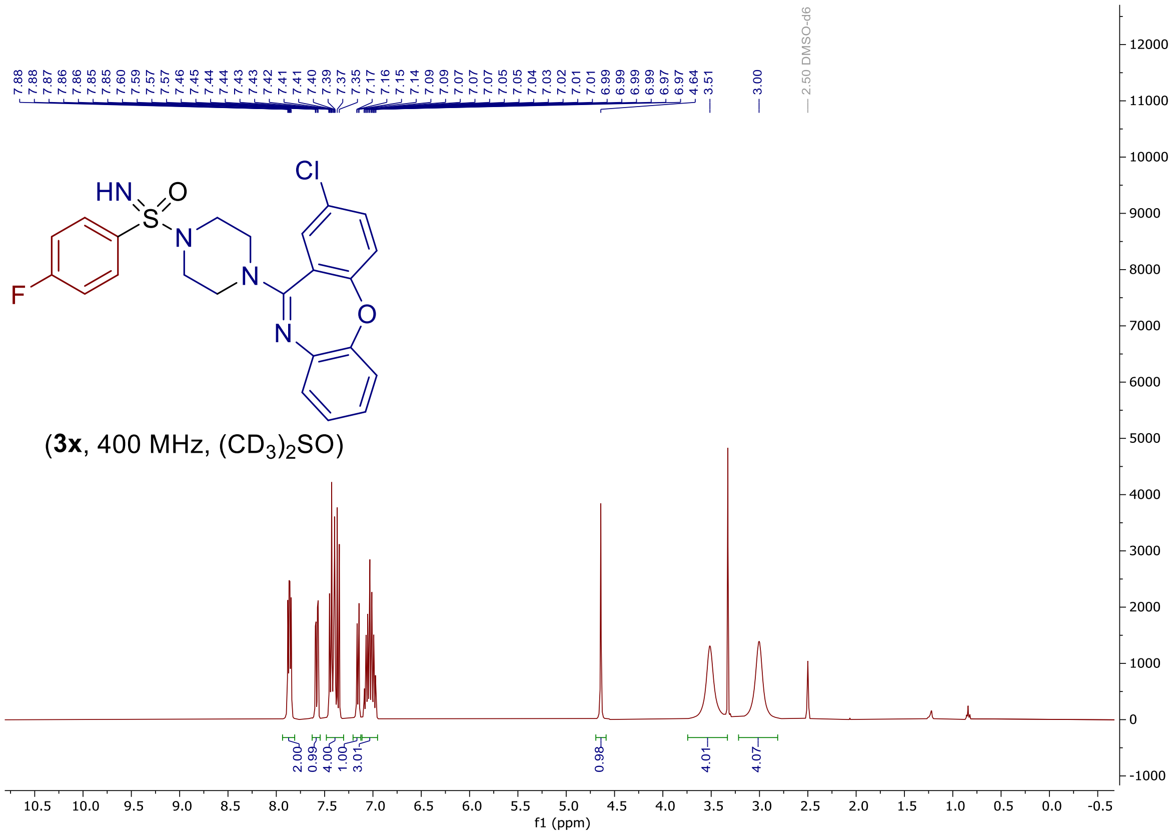
7.88
7.88
7.87
7.86
7.86
7.85
7.85
7.60
7.59
7.57
7.57
7.46
7.45
7.44
7.44
7.43
7.43
7.42
7.41
7.41
7.40
7.39
7.37
7.35
7.17
7.16
7.15
7.14
7.09
7.09
7.07
7.07
7.05
7.05
7.04
7.03
7.02
7.01
7.01
6.99
6.99
6.99
6.99
6.97
6.97
4.64
3.51
3.00
2.50 DMSO-d6
HN
O
S
N
N
Cl
O
N
F
(3x, 400 MHz, (CD3)2SO)
2.00
0.99
4.00
1.00
3.01
0.98
4.01
4.07
f1 (ppm)

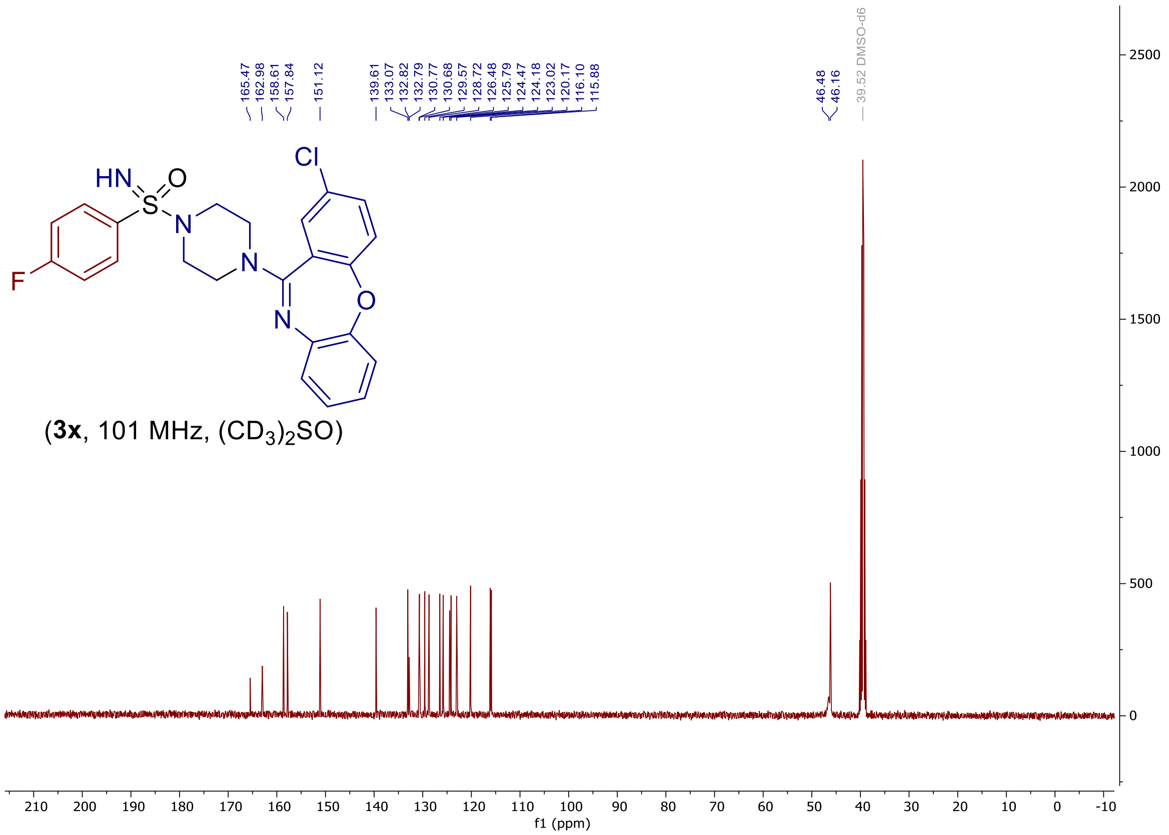

(**3x**, 101 MHz, $(CD_3)_2SO$)

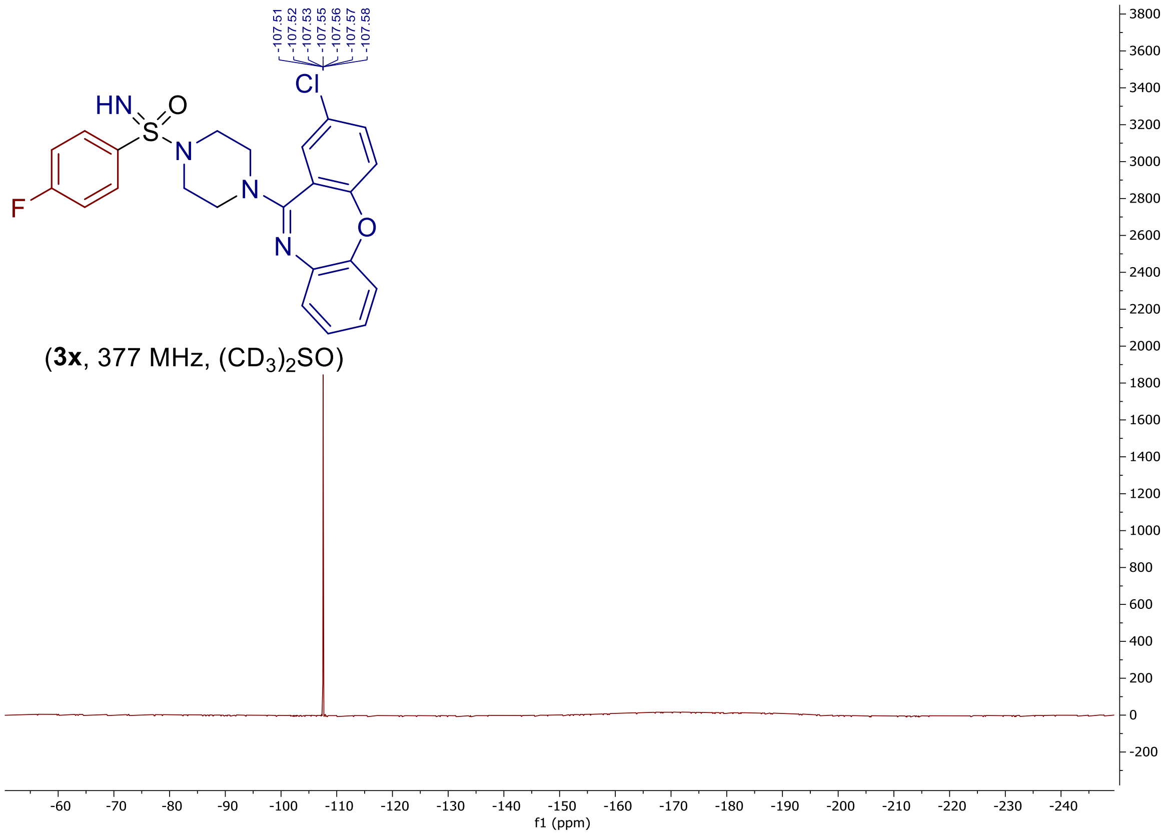

(**3x**, 377 MHz, $(CD_3)_2SO$)

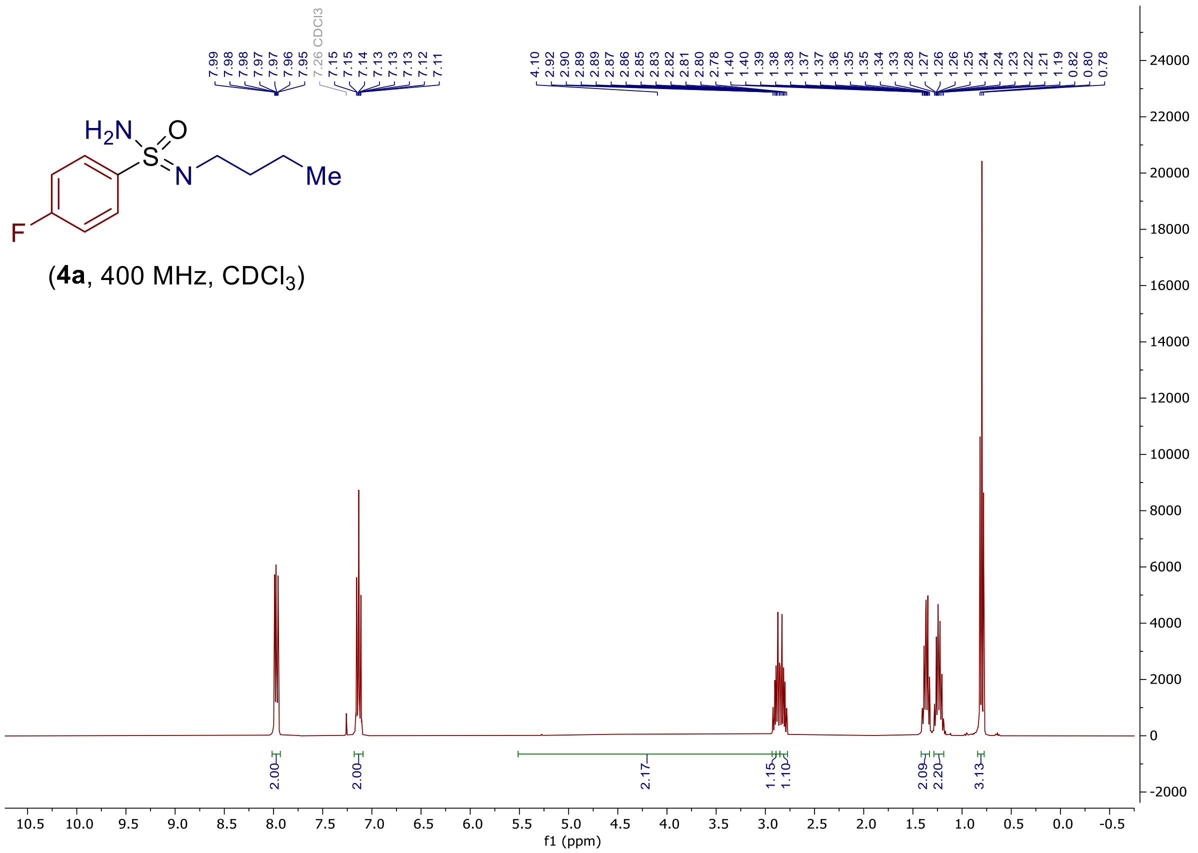
(4a, 400 MHz, CDCl3)

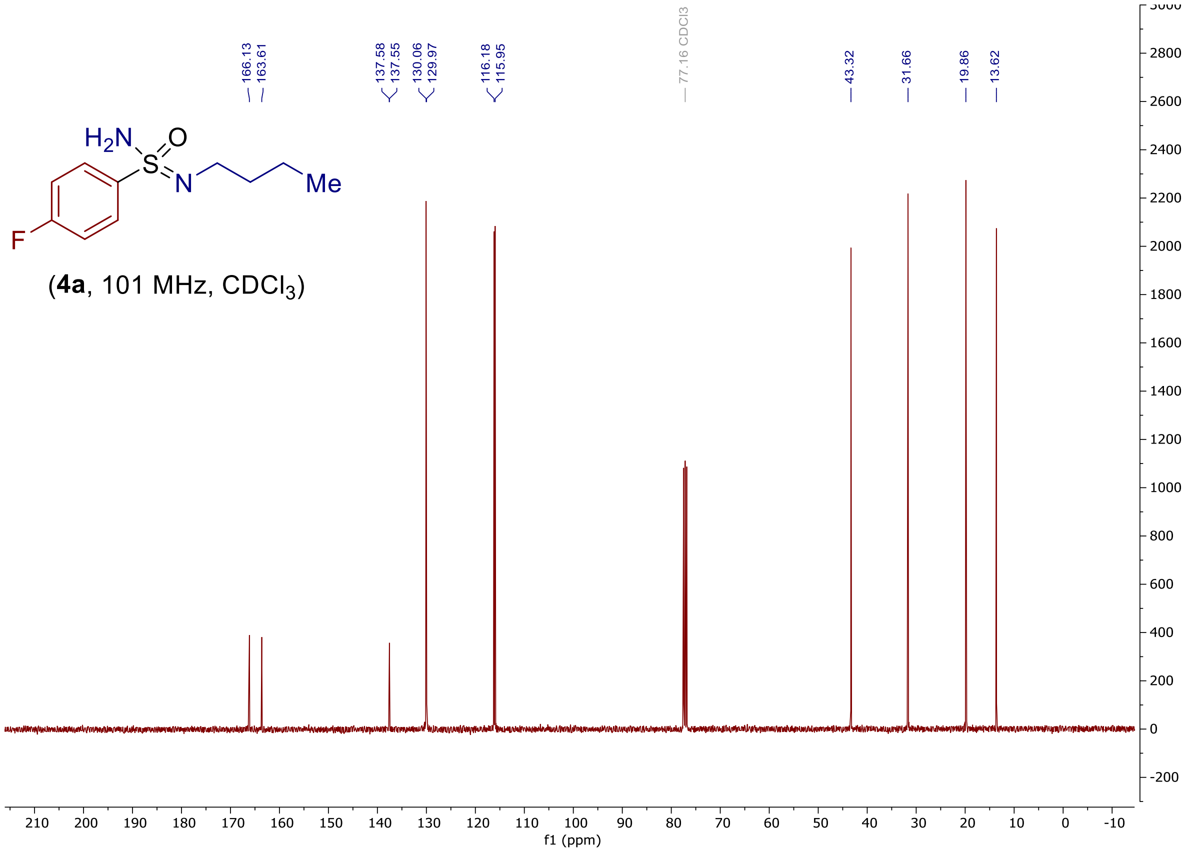
(4a, 101 MHz, CDCl3)

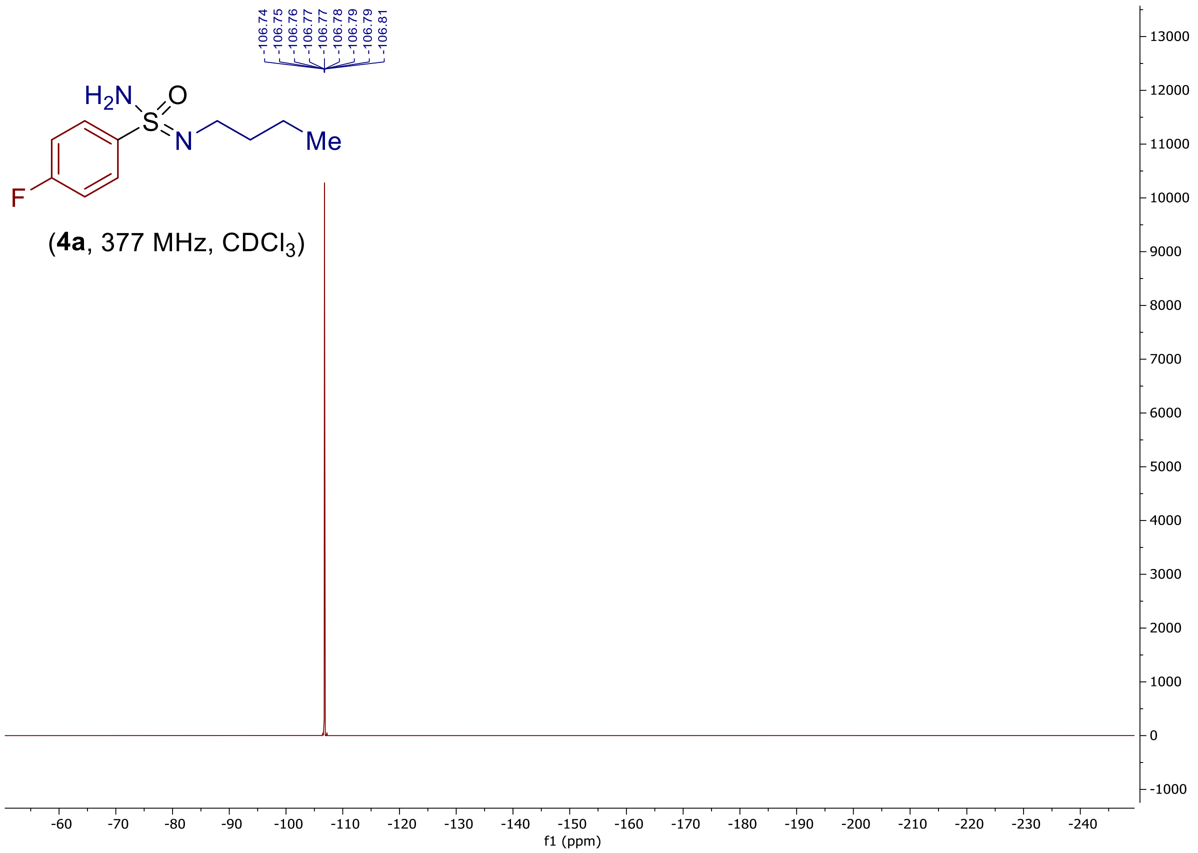

-106.74
-106.75
-106.76
-106.77
-106.77
-106.78
-106.79
-106.79
-106.81
H2N
O
S
N
Me
F
(4a, 377 MHz, CDCl3)
f1 (ppm)

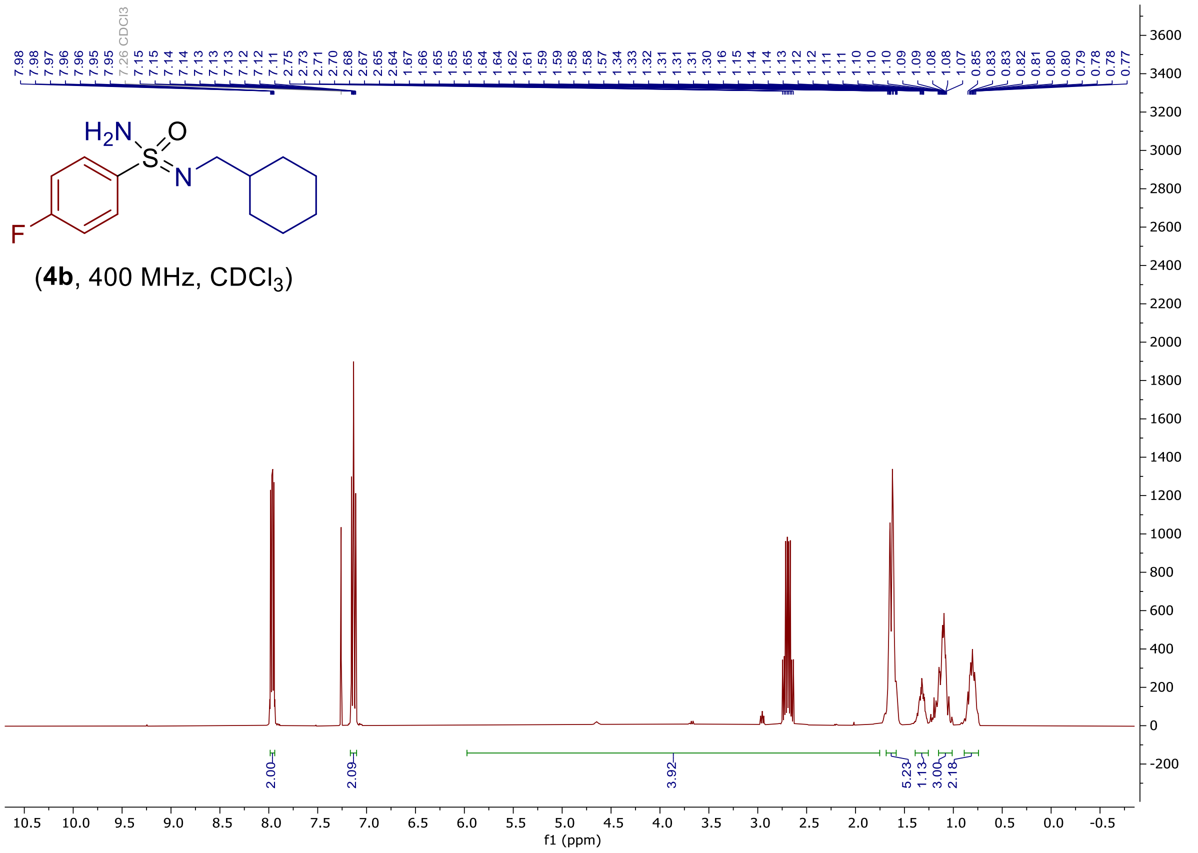

7.26 CDCl3
H2N
O
S
N
F
(4b, 400 MHz, CDCl3)
2.00
2.09
3.92
5.23
1.13
3.00
2.18
f1 (ppm)

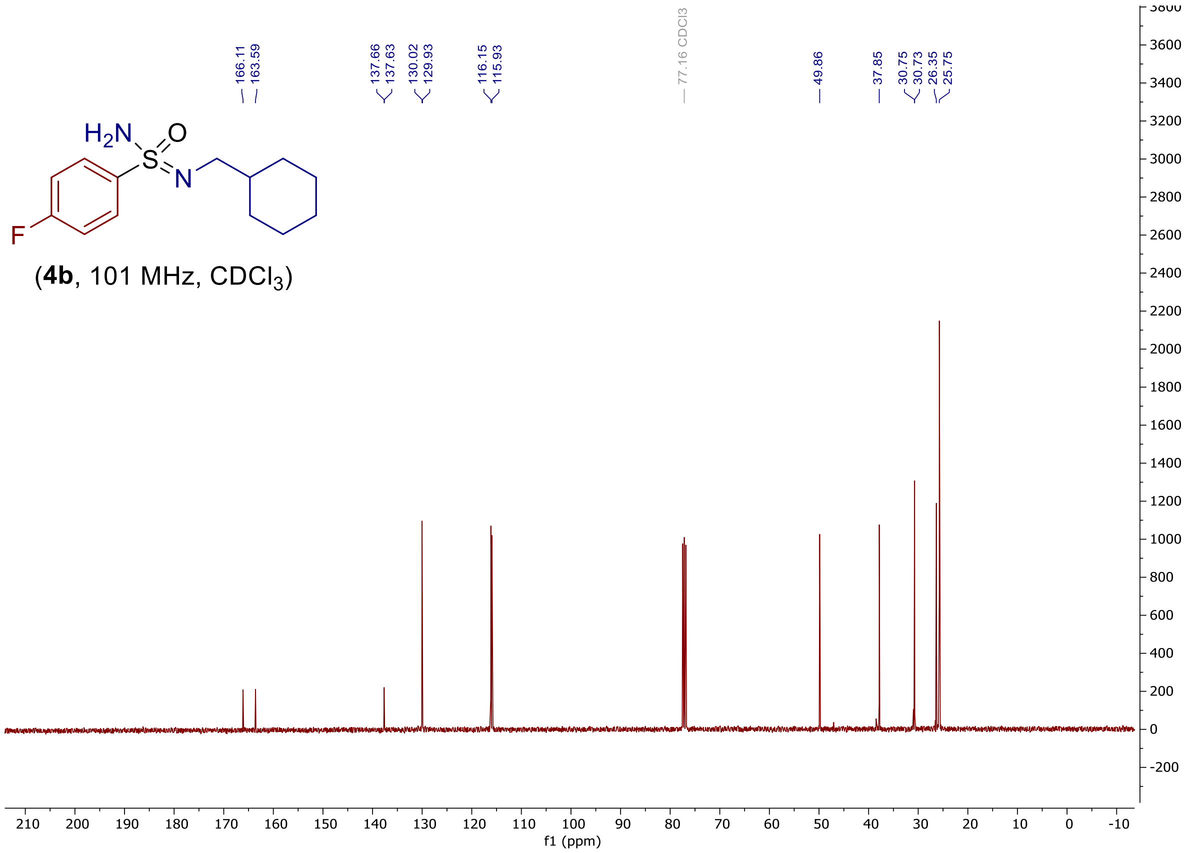
166.11
163.59
137.66
137.63
130.02
129.93
116.15
115.93
77.16 CDCl3
49.86
37.85
30.75
30.73
26.35
25.75
(4b, 101 MHz, CDCl3)
f1 (ppm)

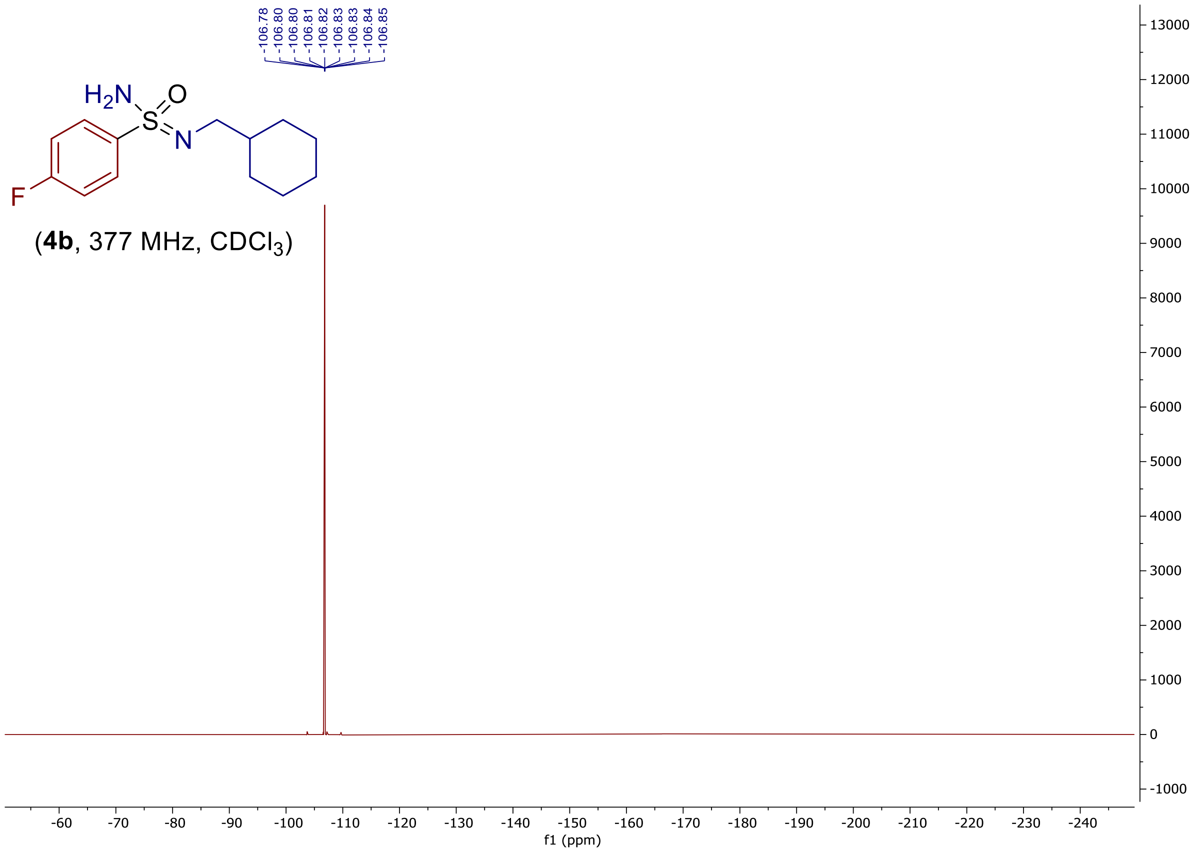
-106.78
-106.80
-106.80
-106.81
-106.82
-106.83
-106.83
-106.84
-106.85
(4b, 377 MHz, CDCl3)
f1 (ppm)

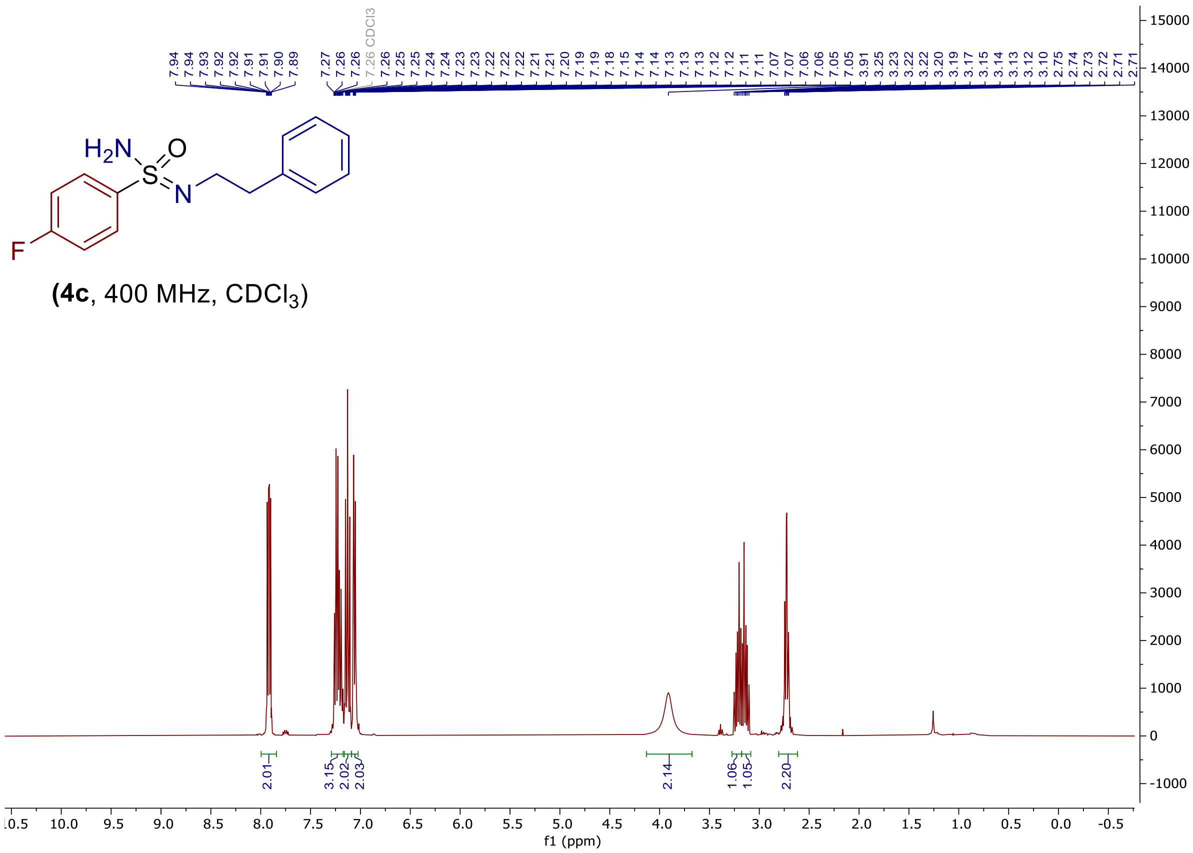
(4c, 400 MHz, CDCl3)
f1 (ppm)

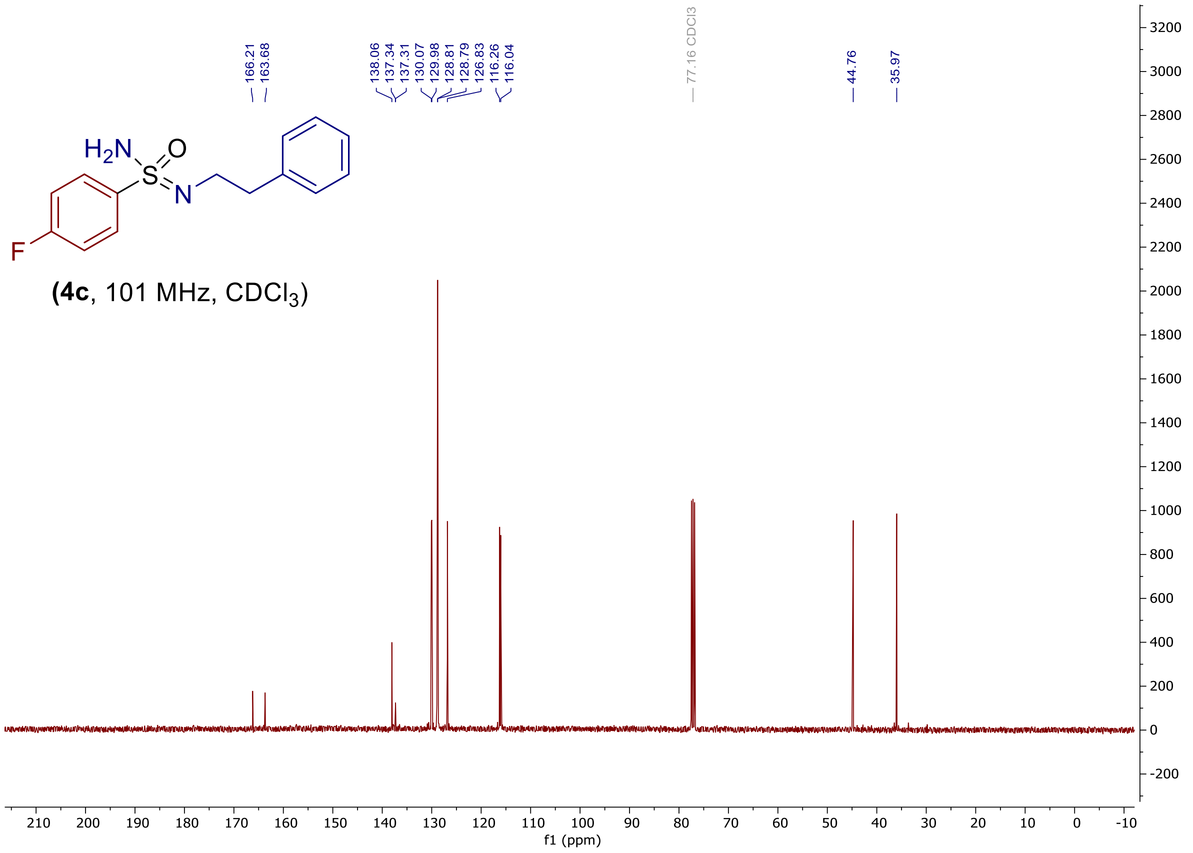
(4c, 101 MHz, CDCl3)
166.21
163.68
138.06
137.34
137.31
130.07
129.98
128.81
128.79
126.83
116.26
116.04
77.16 CDCl3
44.76
35.97
f1 (ppm)

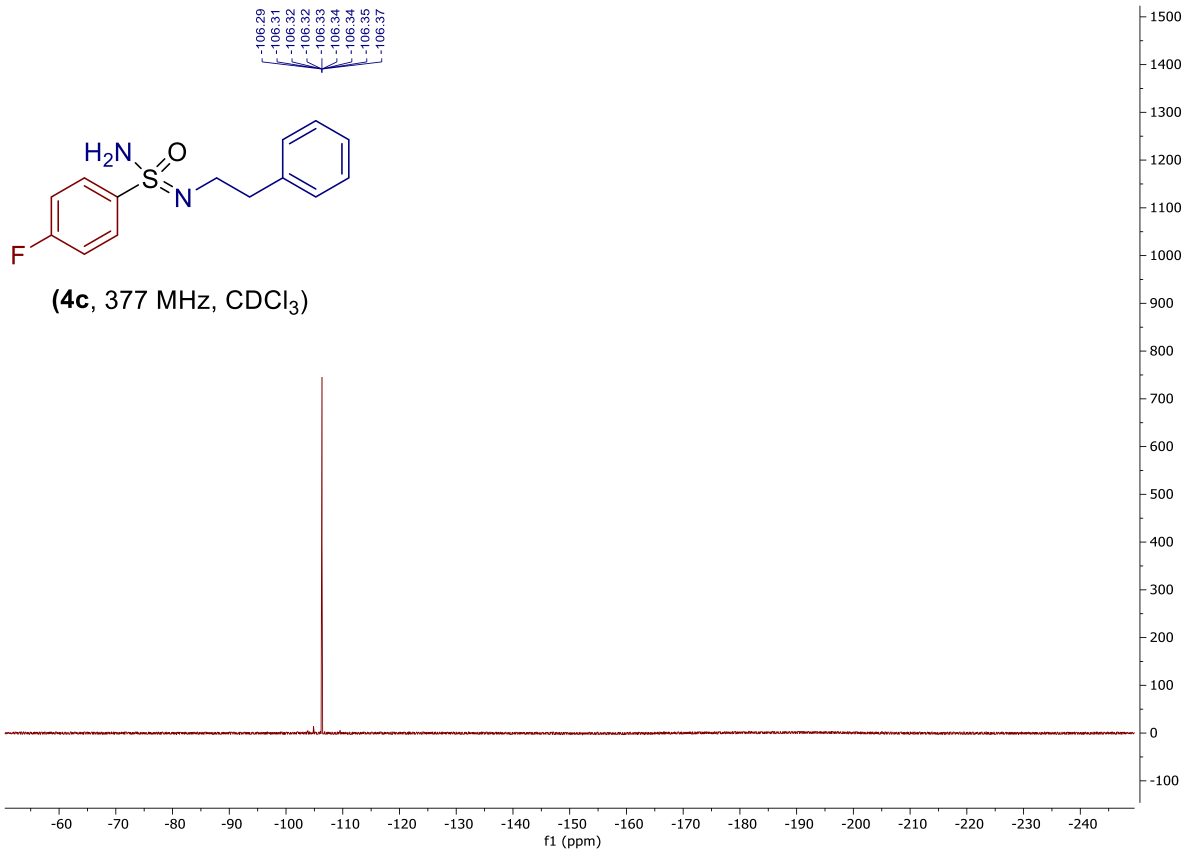
-106.29
-106.31
-106.32
-106.32
-106.33
-106.34
-106.34
-106.35
-106.37
(4c, 377 MHz, CDCl3)
f1 (ppm)

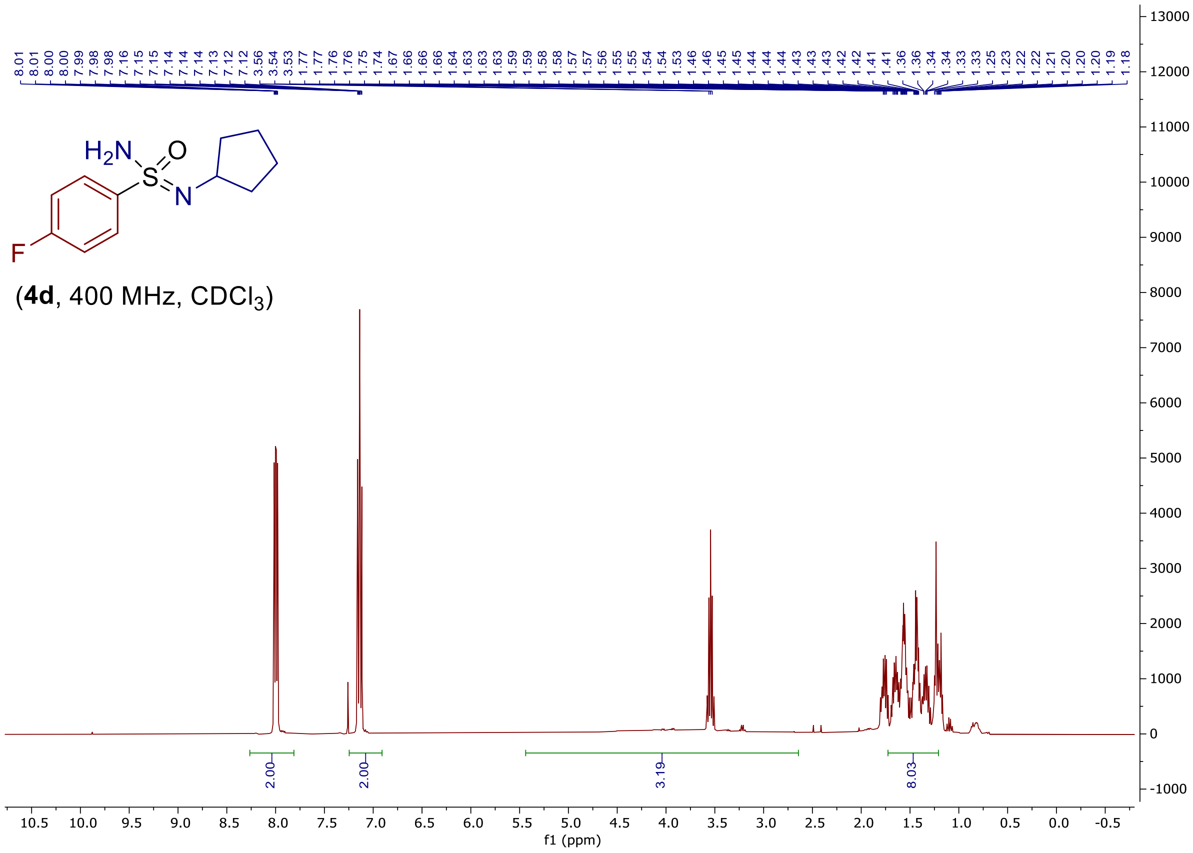
(4d, 400 MHz, CDCl3)
2.00
2.00
3.19
8.03
f1 (ppm)

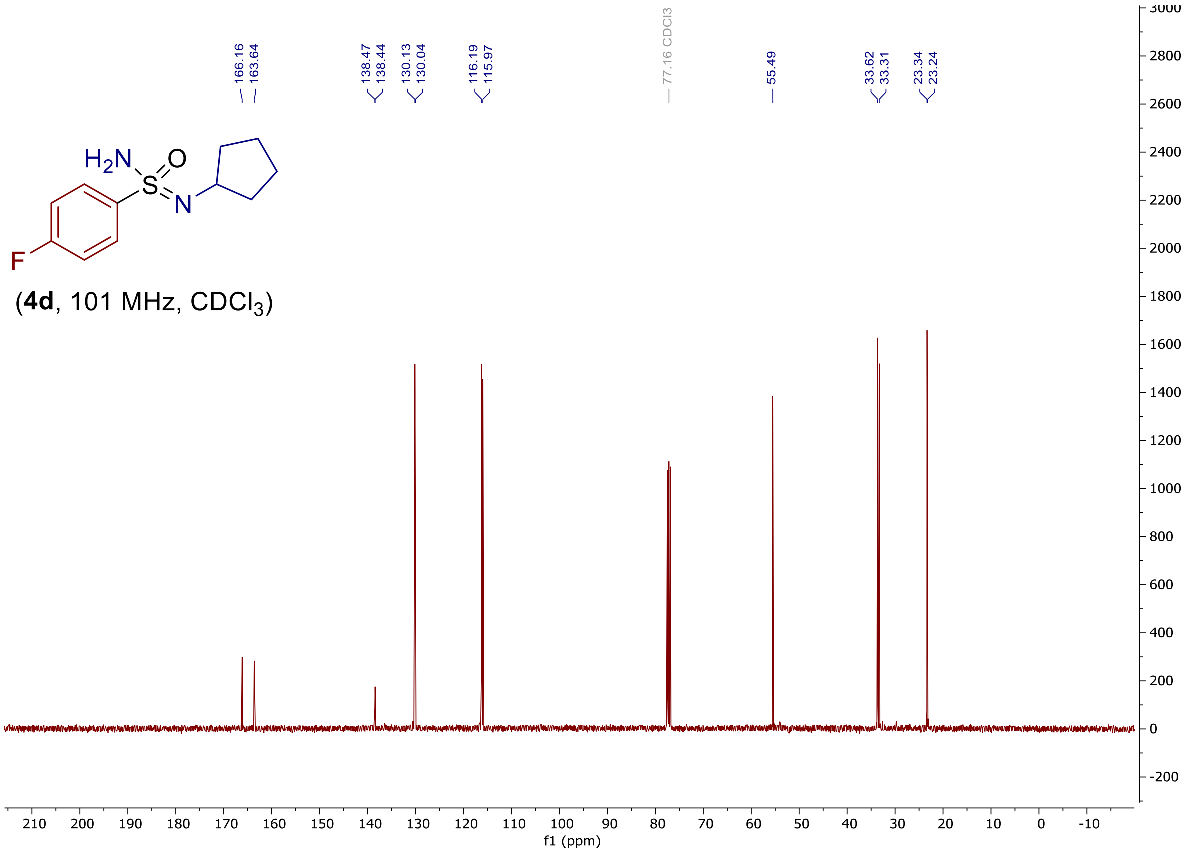
166.16
163.64
138.47
138.44
130.13
130.04
116.19
115.97
77.16 CDCl3
55.49
33.62
33.31
23.34
23.24
H2N
S
O
N
F
(**4d**, 101 MHz, $CDCl_3$)
f1 (ppm)

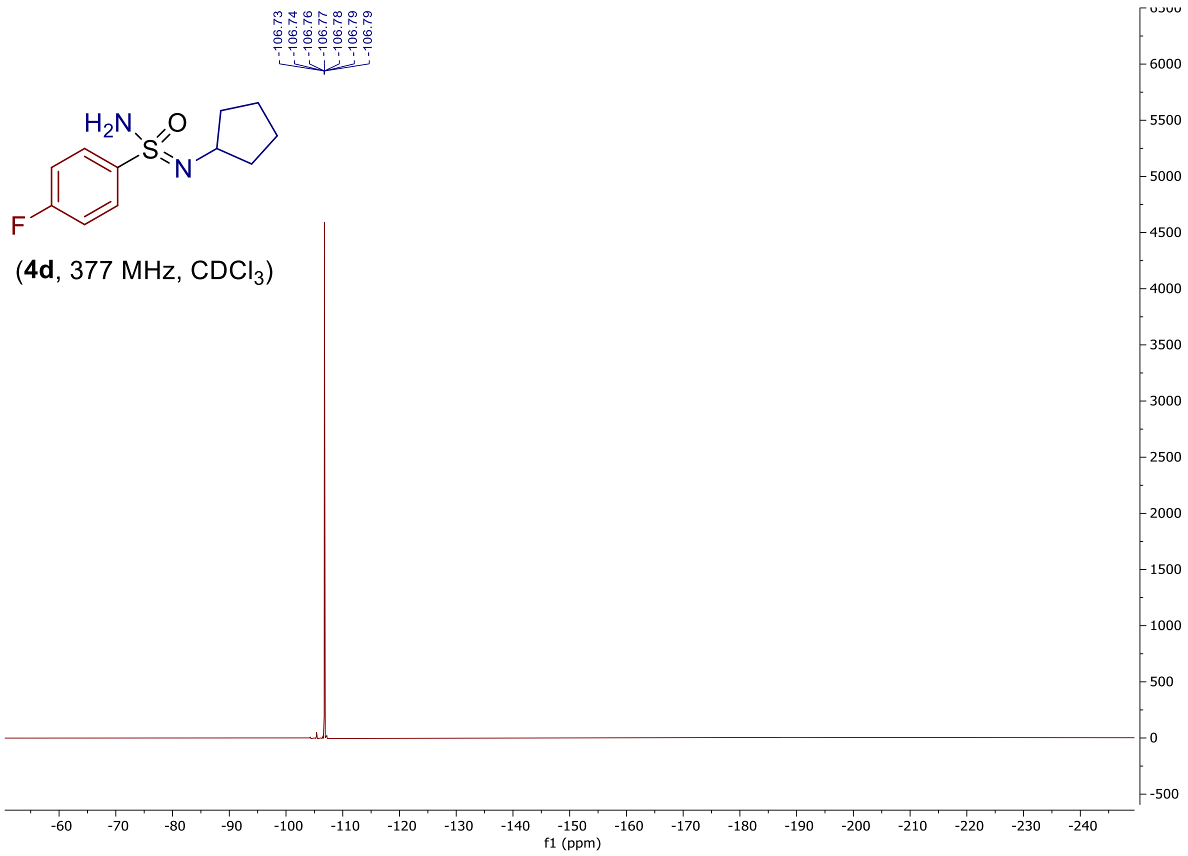
-106.73
-106.74
-106.76
-106.77
-106.78
-106.79
-106.79
H2N
S
O
N
F
(**4d**, 377 MHz, $CDCl_3$)
f1 (ppm)

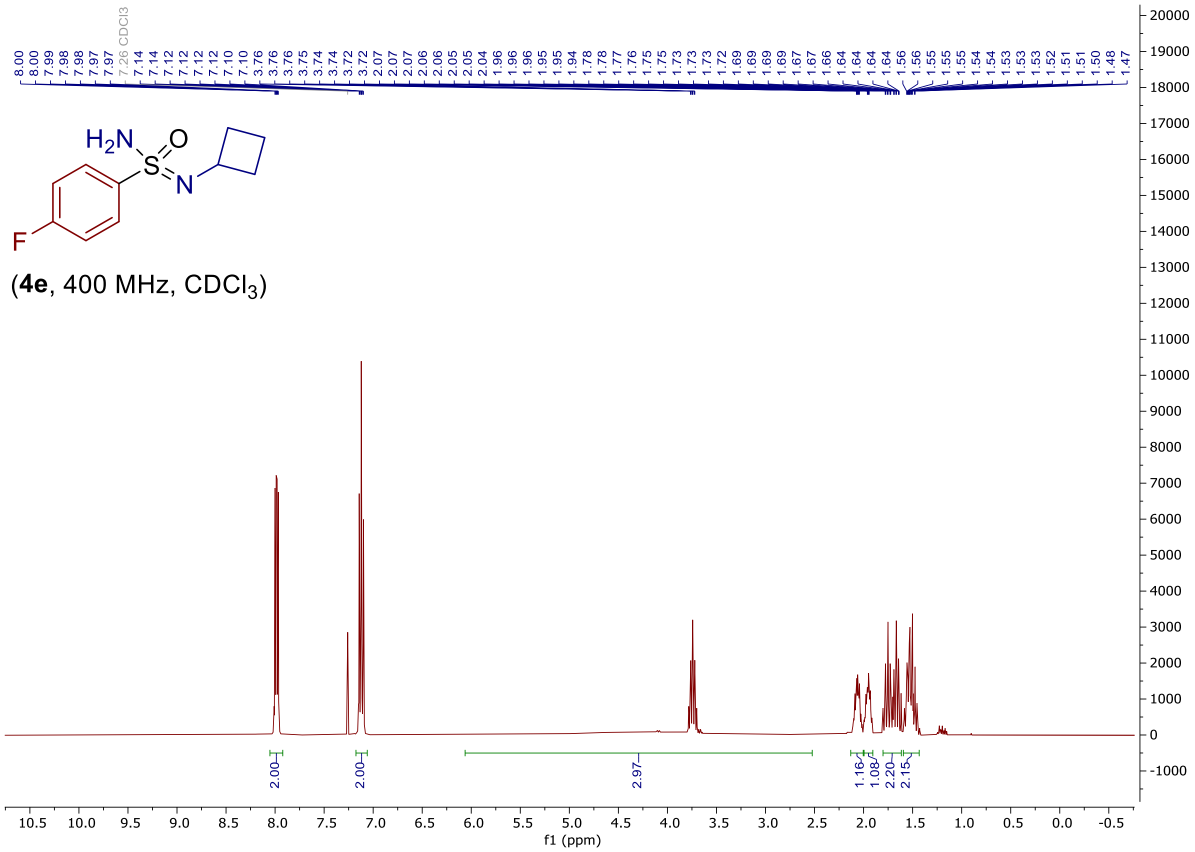
8.00 8.00 7.99 7.98 7.98 7.97 7.97 7.26 CDCl3 7.14 7.14 7.12 7.12 7.12 7.12 7.10 7.10 3.76 3.76 3.75 3.74 3.74 3.72 3.72 2.07 2.07 2.07 2.06 2.06 2.05 2.05 2.04 1.96 1.96 1.96 1.95 1.95 1.94 1.78 1.78 1.77 1.76 1.75 1.75 1.73 1.73 1.73 1.72 1.69 1.69 1.69 1.69 1.67 1.67 1.66 1.64 1.64 1.64 1.64 1.56 1.56 1.55 1.55 1.55 1.54 1.54 1.53 1.53 1.53 1.52 1.51 1.51 1.50 1.48 1.47
H2N
O
S
N
F
(4e, 400 MHz, CDCl3)
2.00
2.00
2.97
1.16
1.08
2.20
2.15
f1 (ppm)

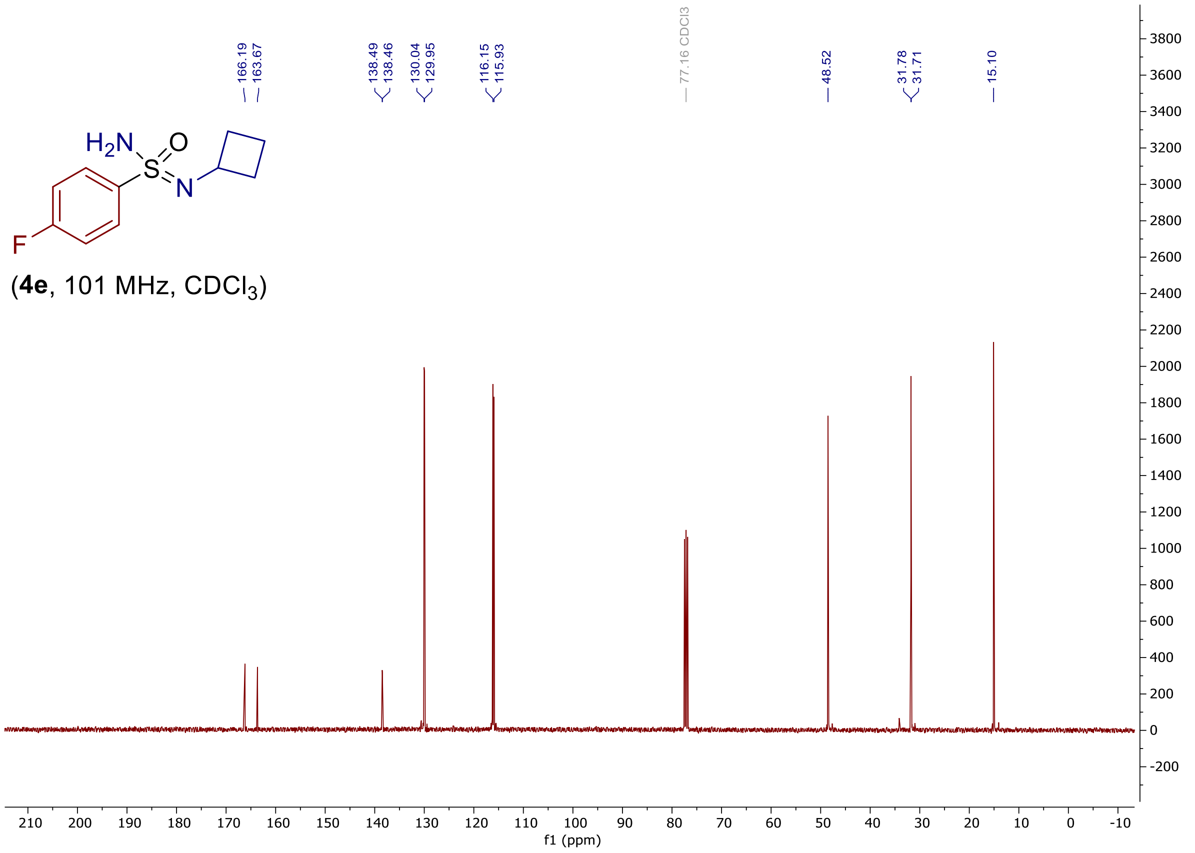
166.19 163.67 138.49 138.46 130.04 129.95 116.15 115.93 77.16 CDCl3 48.52 31.78 31.71 15.10
H2N
O
S
N
F
(4e, 101 MHz, CDCl3)
f1 (ppm)

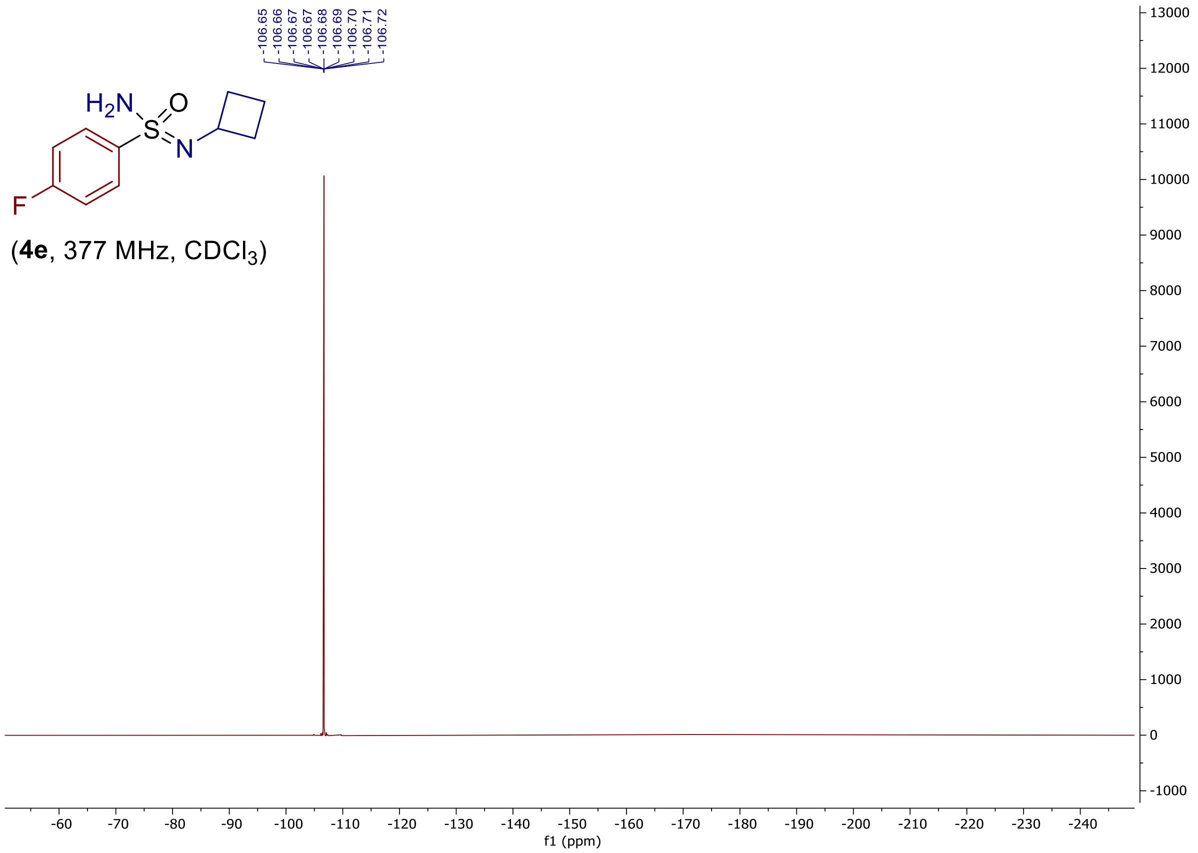

-106.65
-106.66
-106.67
-106.67
-106.68
-106.69
-106.70
-106.71
-106.72
(4e, 377 MHz, CDCl3)
f1 (ppm)

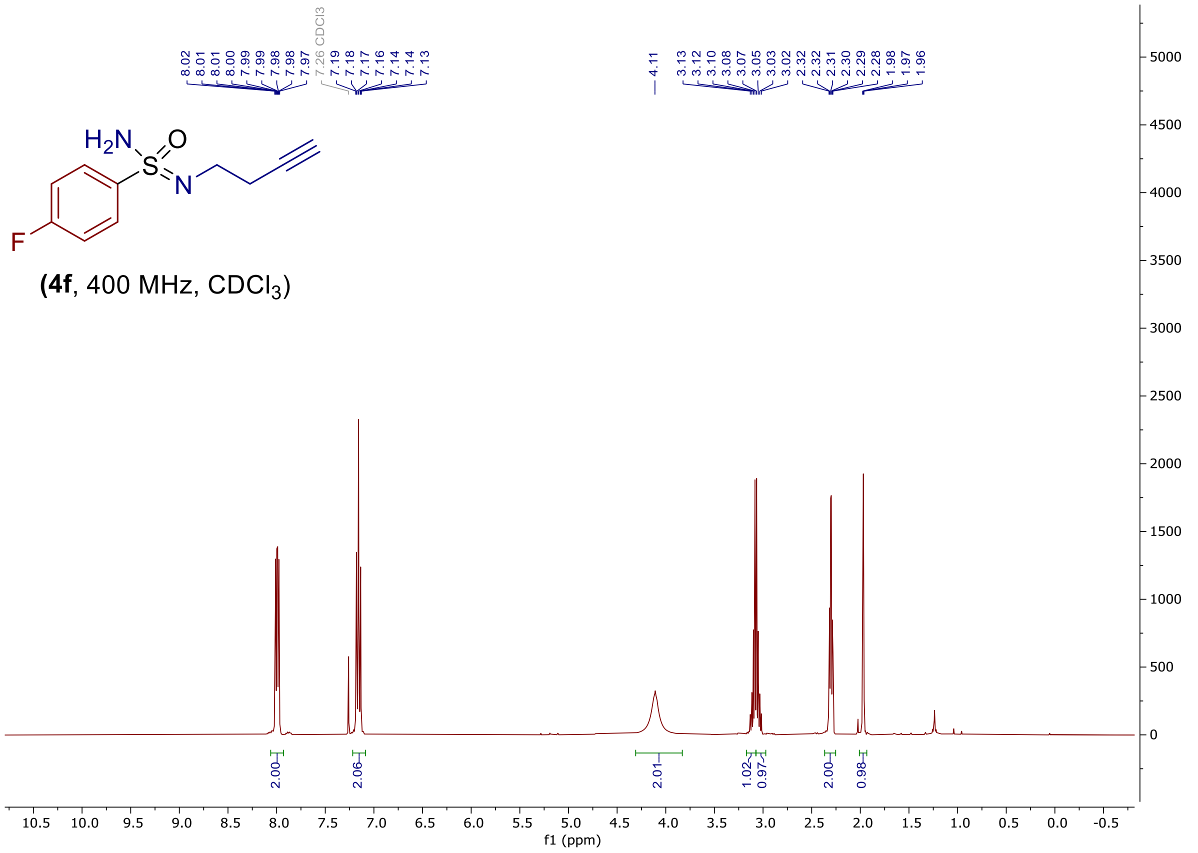

8.02
8.01
8.01
8.00
7.99
7.99
7.98
7.98
7.97
7.26 CDCl3
7.19
7.18
7.17
7.16
7.14
7.14
7.13
4.11
3.13
3.12
3.10
3.08
3.07
3.05
3.03
3.02
2.32
2.32
2.31
2.30
2.29
2.28
1.98
1.97
1.96
(4f, 400 MHz, CDCl3)
2.00
2.06
2.01
1.02
0.97
2.00
0.98
f1 (ppm)

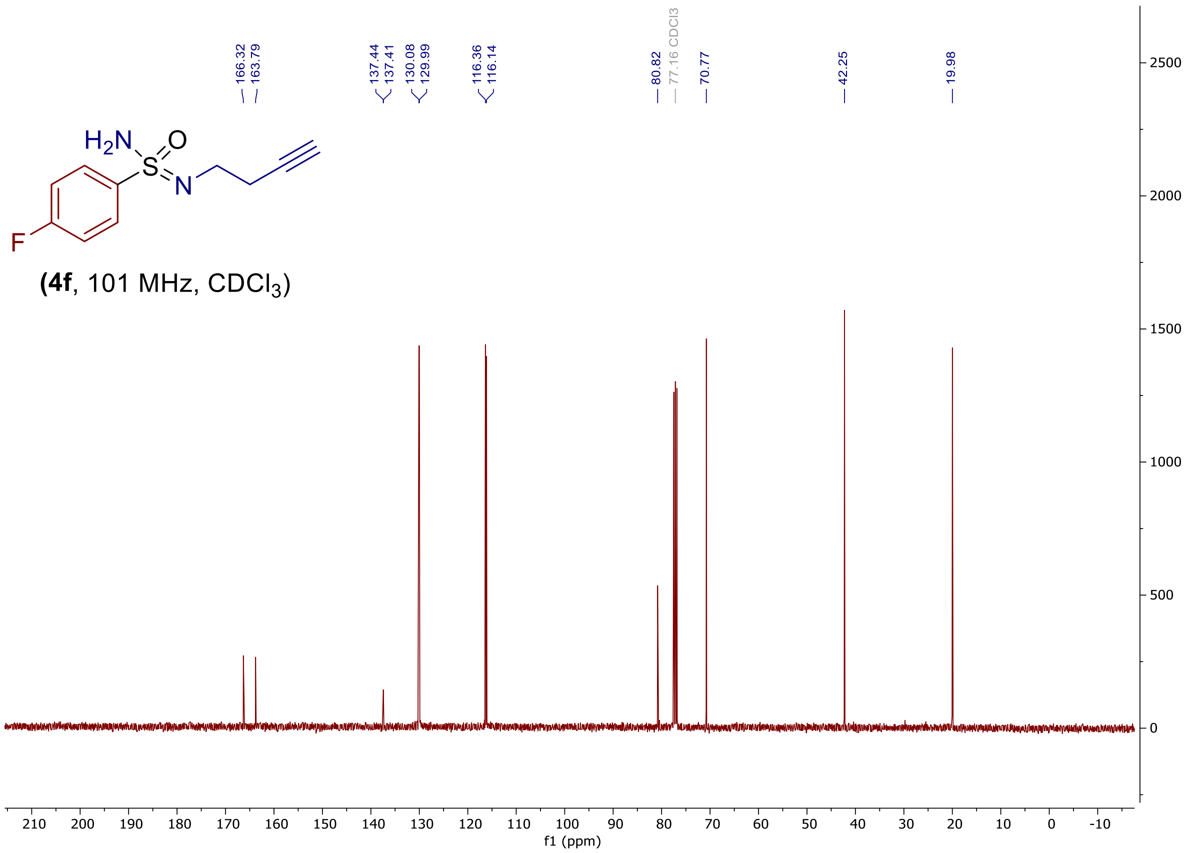

166.32
163.79
137.44
137.41
130.08
129.99
116.36
116.14
80.82
77.16 CDCl3
70.77
42.25
19.98
H_2N
S
O
N
F
(4f, 101 MHz, CDCl3)
2500
2000
1500
1000
500
0
210
200
190
180
170
160
150
140
130
120
110
100
90
80
70
60
50
40
30
20
10
0
-10
f1 (ppm)

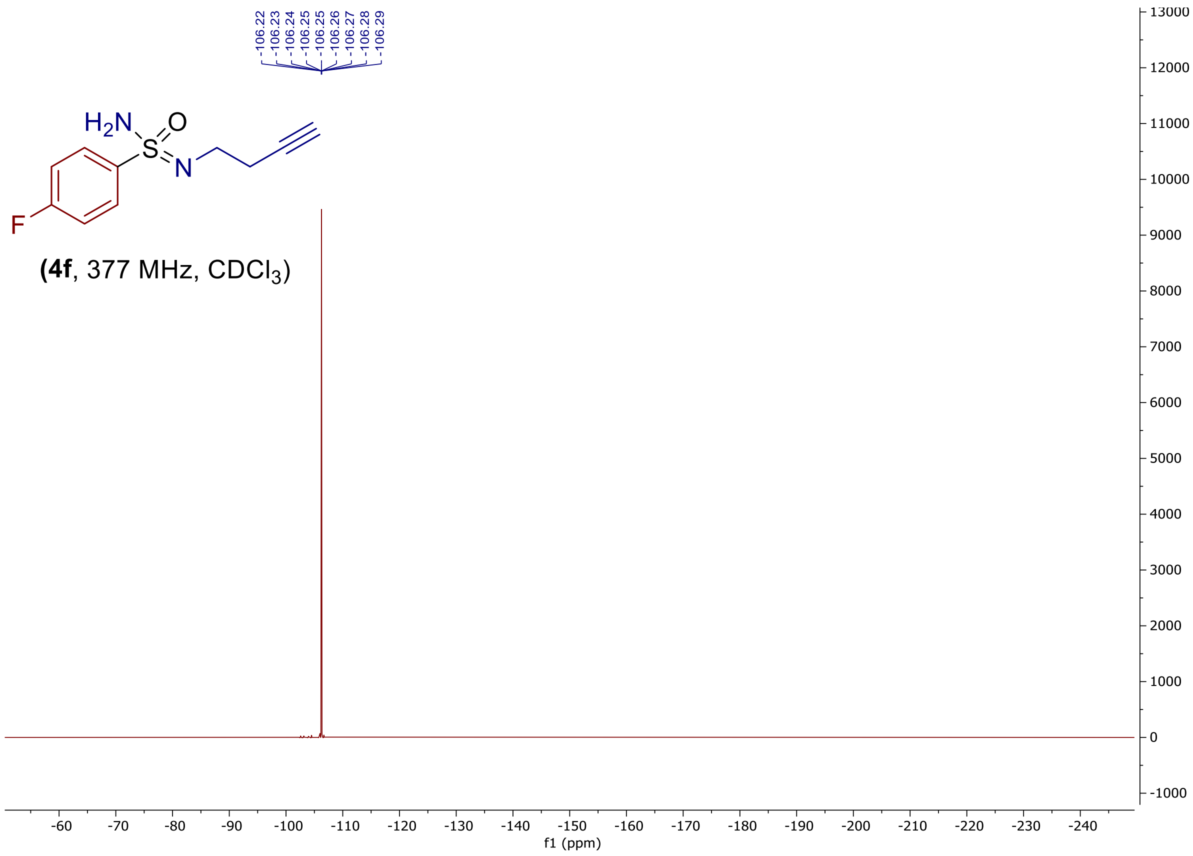

-106.22
-106.23
-106.24
-106.25
-106.25
-106.26
-106.27
-106.28
-106.29
H_2N
S
O
N
F
(4f, 377 MHz, CDCl3)
13000
12000
11000
10000
9000
8000
7000
6000
5000
4000
3000
2000
1000
0
-1000
-60
-70
-80
-90
-100
-110
-120
-130
-140
-150
-160
-170
-180
-190
-200
-210
-220
-230
-240
f1 (ppm)

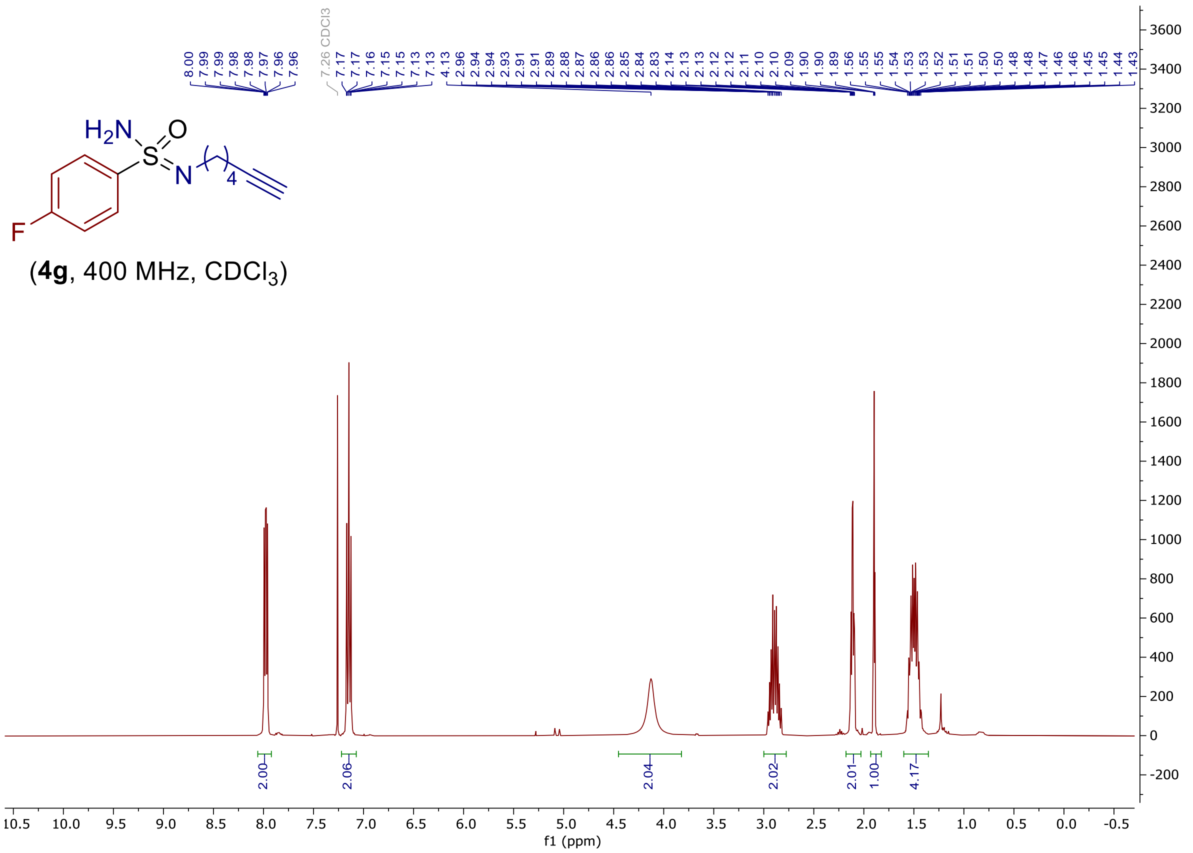
8.00
7.99
7.99
7.98
7.98
7.97
7.96
7.96
7.26 CDCl3
7.17
7.17
7.16
7.15
7.15
7.13
7.13
4.13
2.96
2.94
2.94
2.93
2.91
2.91
2.89
2.88
2.87
2.86
2.86
2.85
2.84
2.83
2.14
2.13
2.13
2.12
2.12
2.11
2.10
2.10
2.09
1.90
1.90
1.89
1.56
1.55
1.55
1.54
1.53
1.53
1.52
1.51
1.51
1.50
1.50
1.48
1.48
1.47
1.46
1.46
1.45
1.45
1.44
1.43
H2N
S
O
N
4
F
(4g, 400 MHz, CDCl3)
2.00
2.06
2.04
2.02
2.01
1.00
4.17
f1 (ppm)

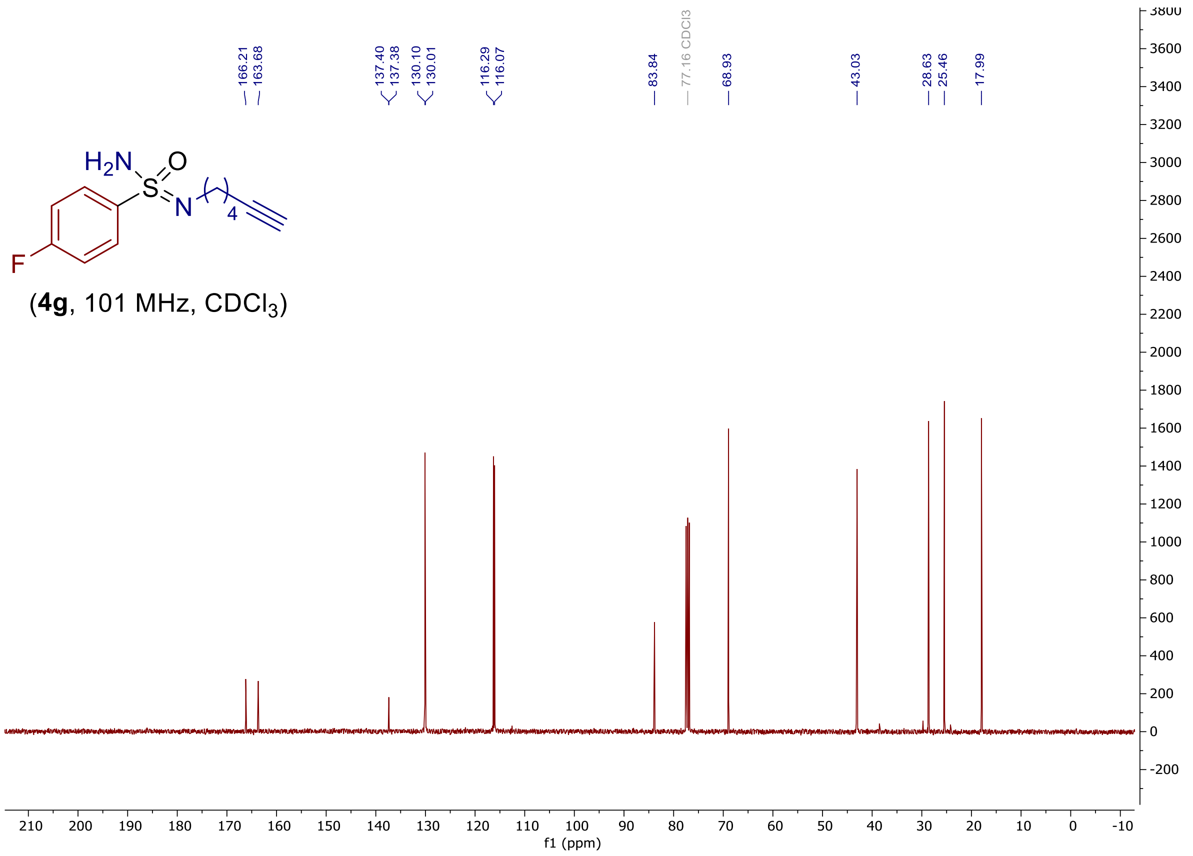
166.21
163.68
137.40
137.38
130.10
130.01
116.29
116.07
83.84
77.16 CDCl3
68.93
43.03
28.63
25.46
17.99
H2N
S
O
N
4
F
(4g, 101 MHz, CDCl3)
f1 (ppm)

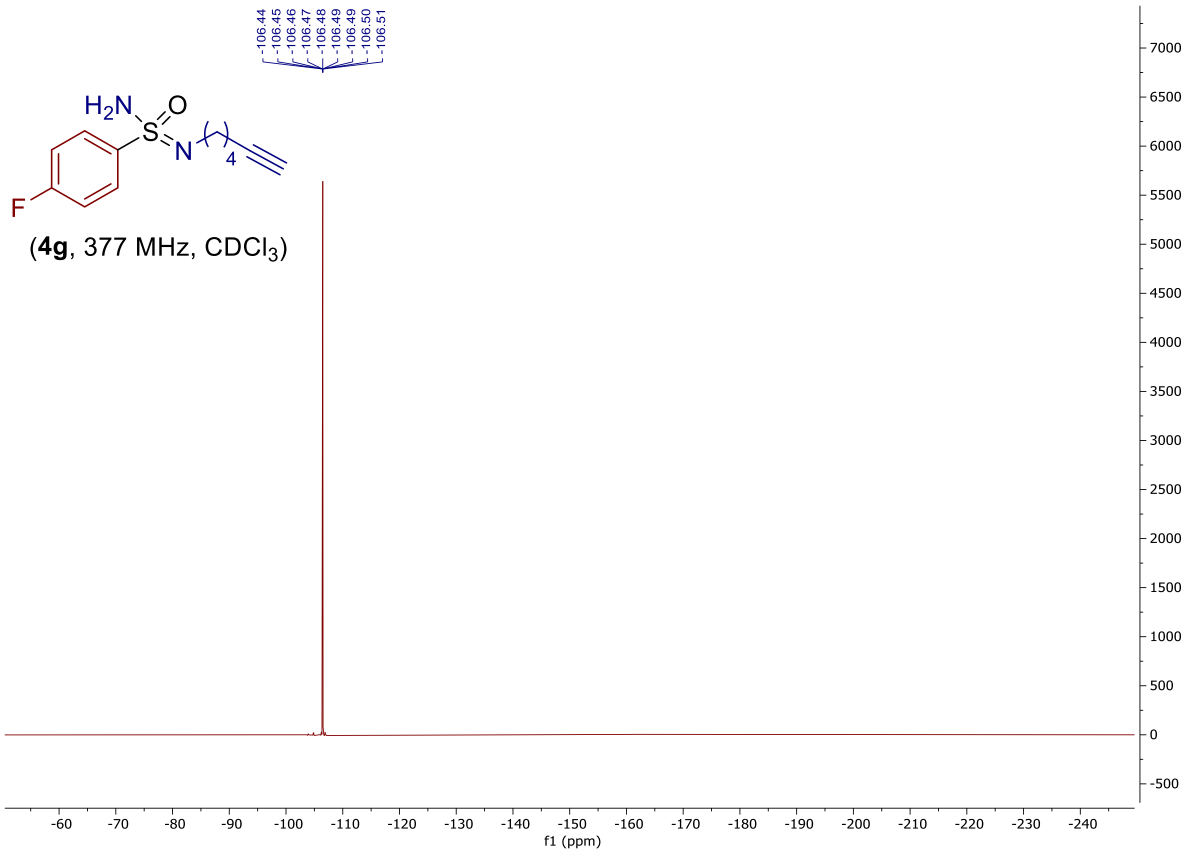

(**4g**, 377 MHz, $CDCl_3$)

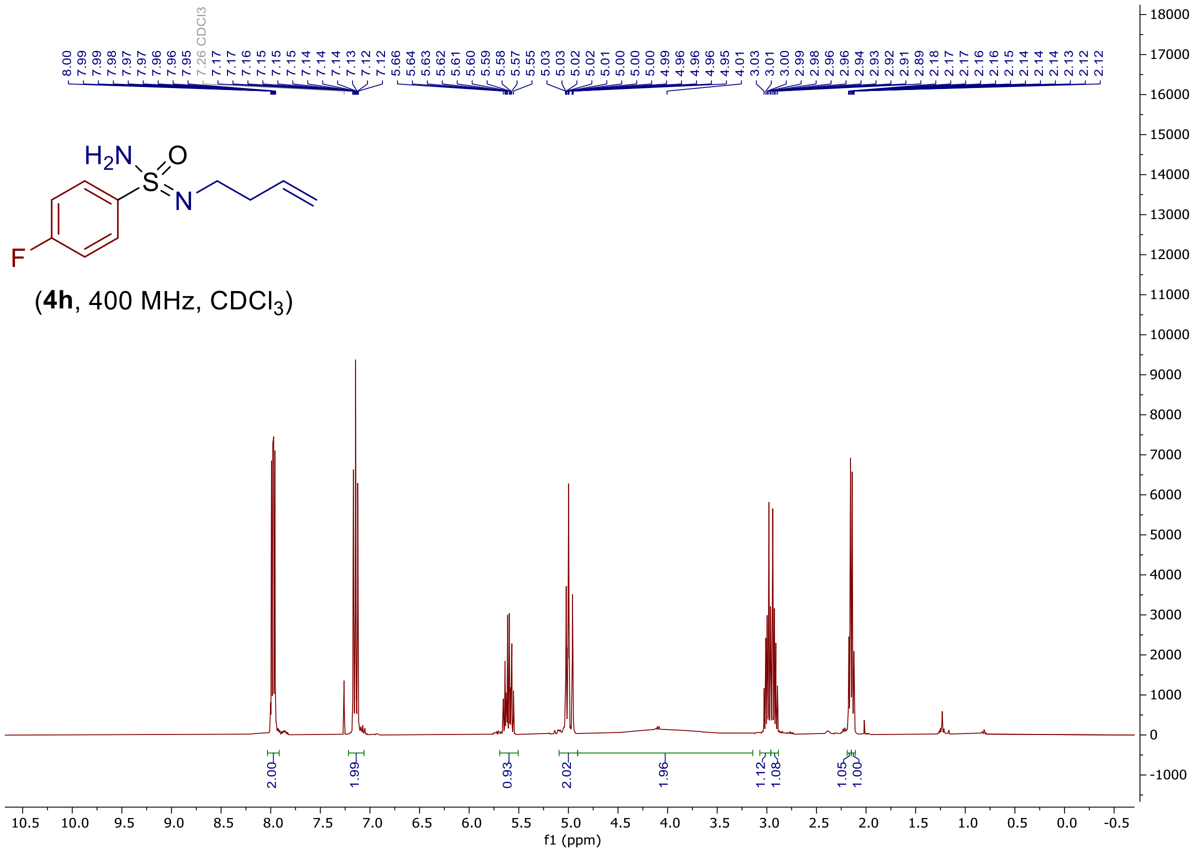

(**4h**, 400 MHz, $CDCl_3$)

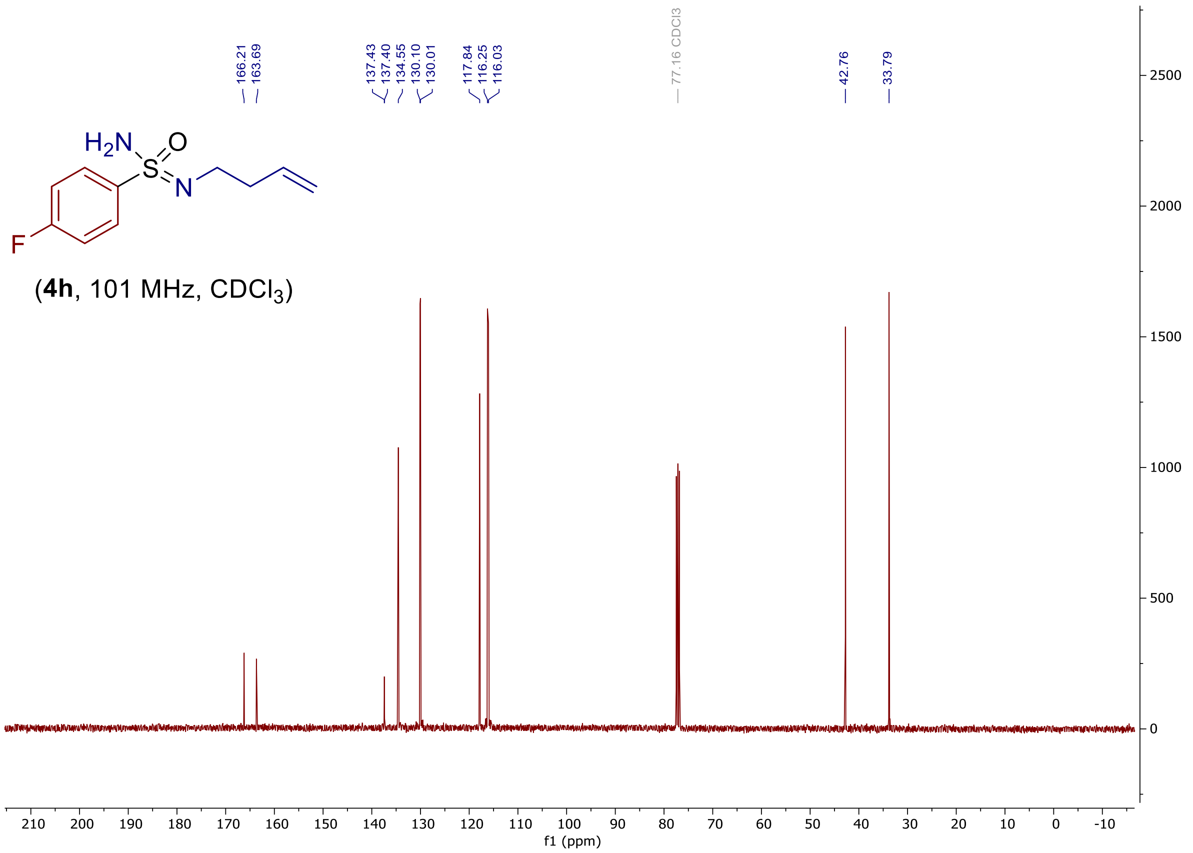
166.21
163.69
137.43
137.40
134.55
130.10
130.01
117.84
116.25
116.03
77.16 CDCl3
42.76
33.79
H2N
O
S
N
F
(4h, 101 MHz, CDCl3)
f1 (ppm)

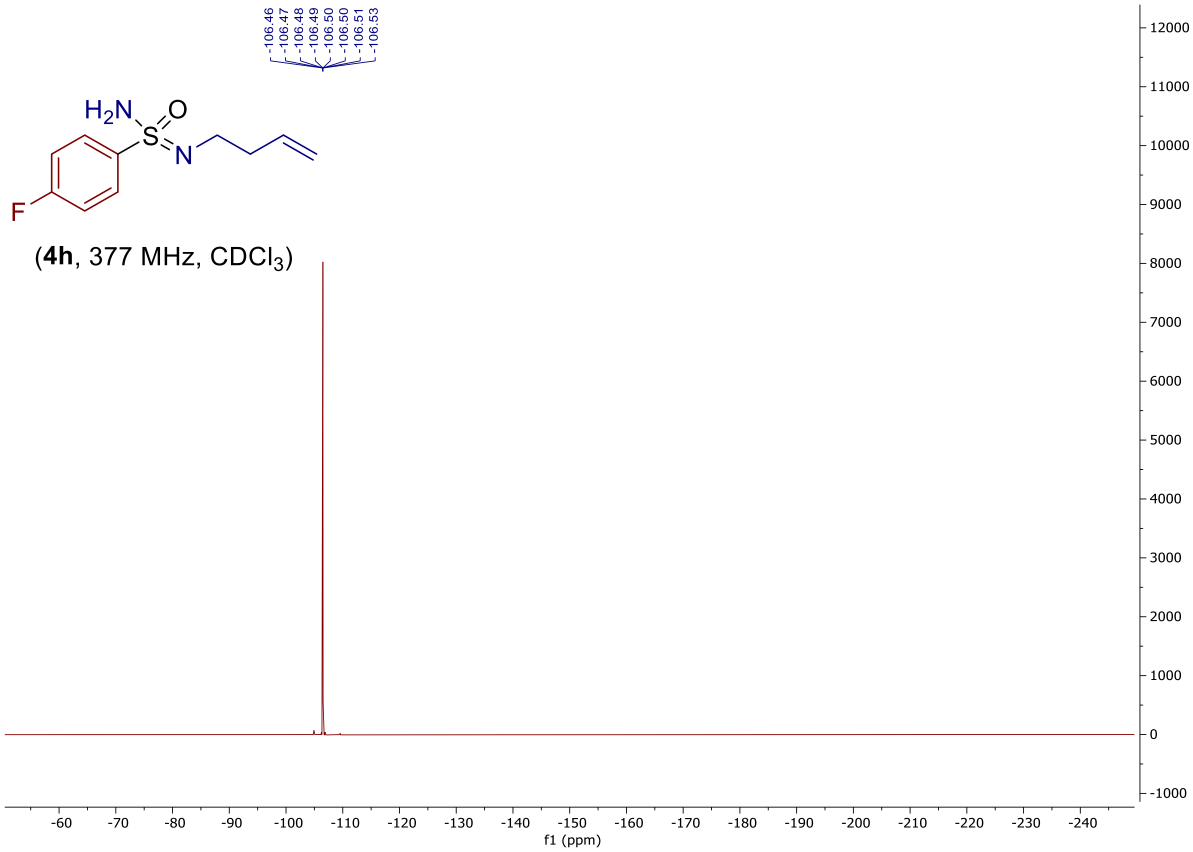
-106.46
-106.47
-106.48
-106.49
-106.50
-106.50
-106.51
-106.53
H2N
O
S
N
F
(4h, 377 MHz, CDCl3)
f1 (ppm)

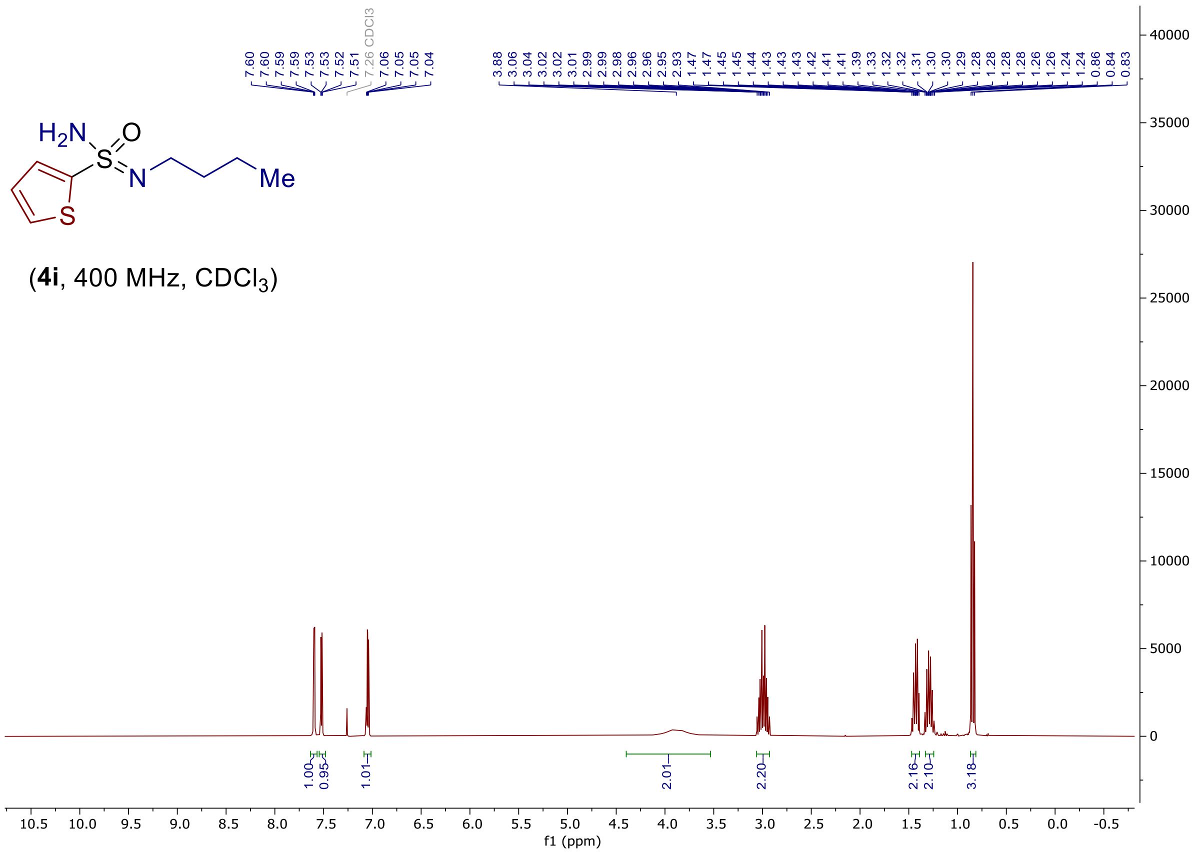

(**4i**, 400 MHz, $CDCl_3$)

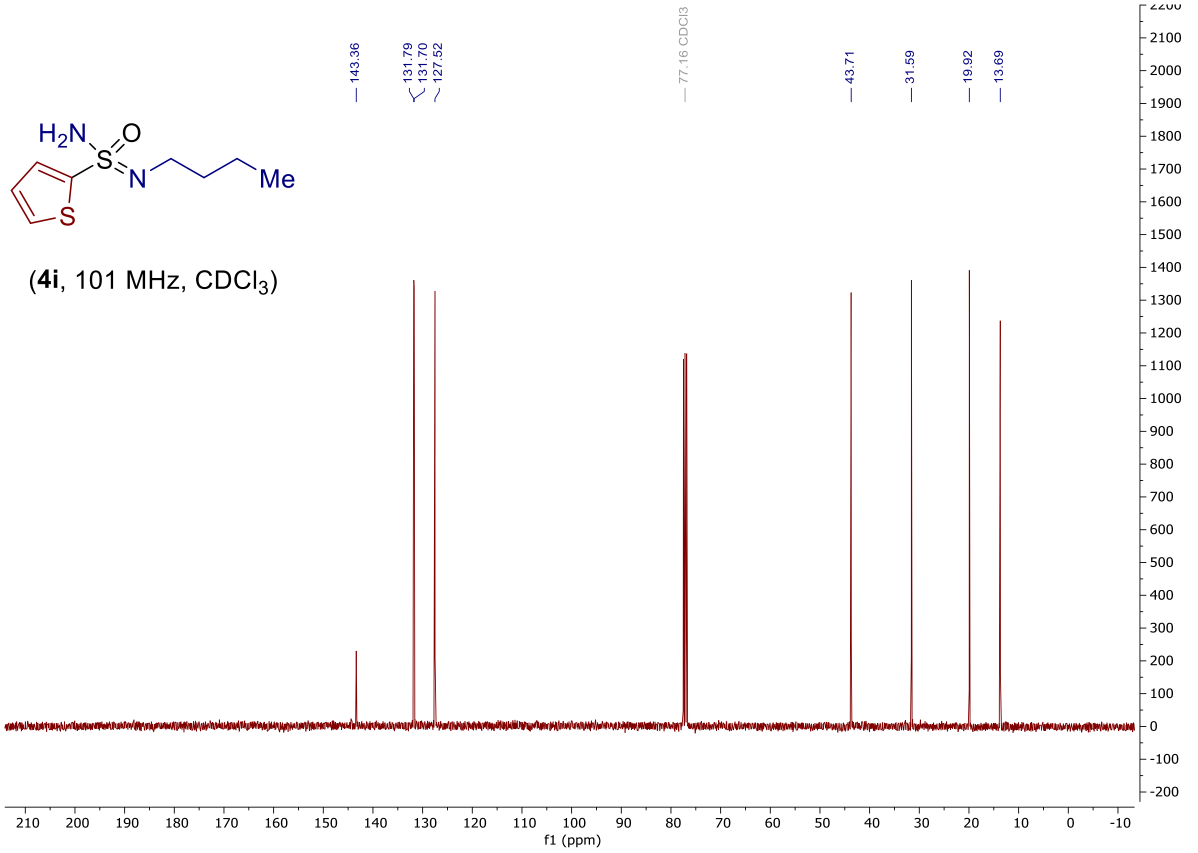

(**4i**, 101 MHz, $CDCl_3$)

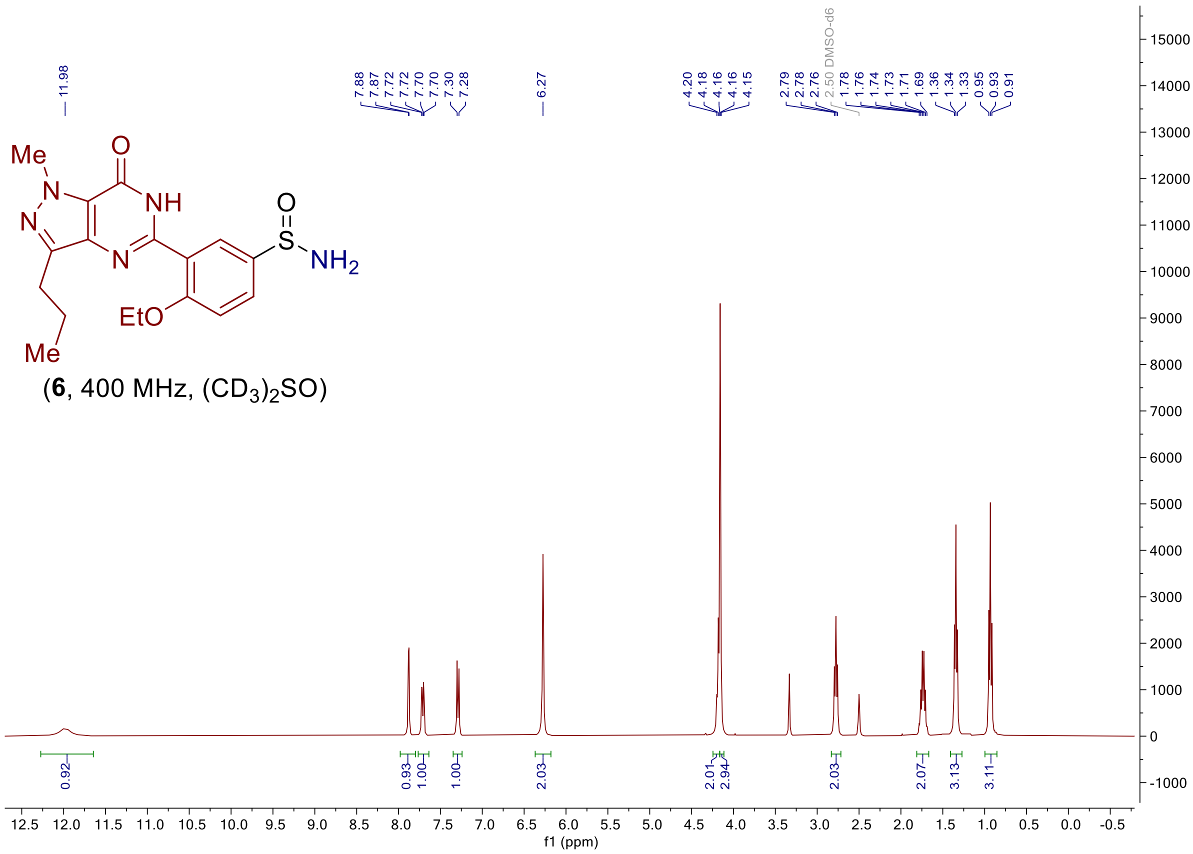
(6, 400 MHz, (CD3)2SO)
11.98
7.88 7.87 7.72 7.72 7.70 7.70 7.30 7.28
6.27
4.20 4.18 4.16 4.16 4.15
2.79 2.78 2.76 2.50 DMSO-d6 1.78 1.76 1.74 1.73 1.71 1.69 1.36 1.34 1.33 0.95 0.93 0.91
0.92 0.93 1.00 1.00 2.03 2.01 2.94 2.03 2.07 3.13 3.11
f1 (ppm)

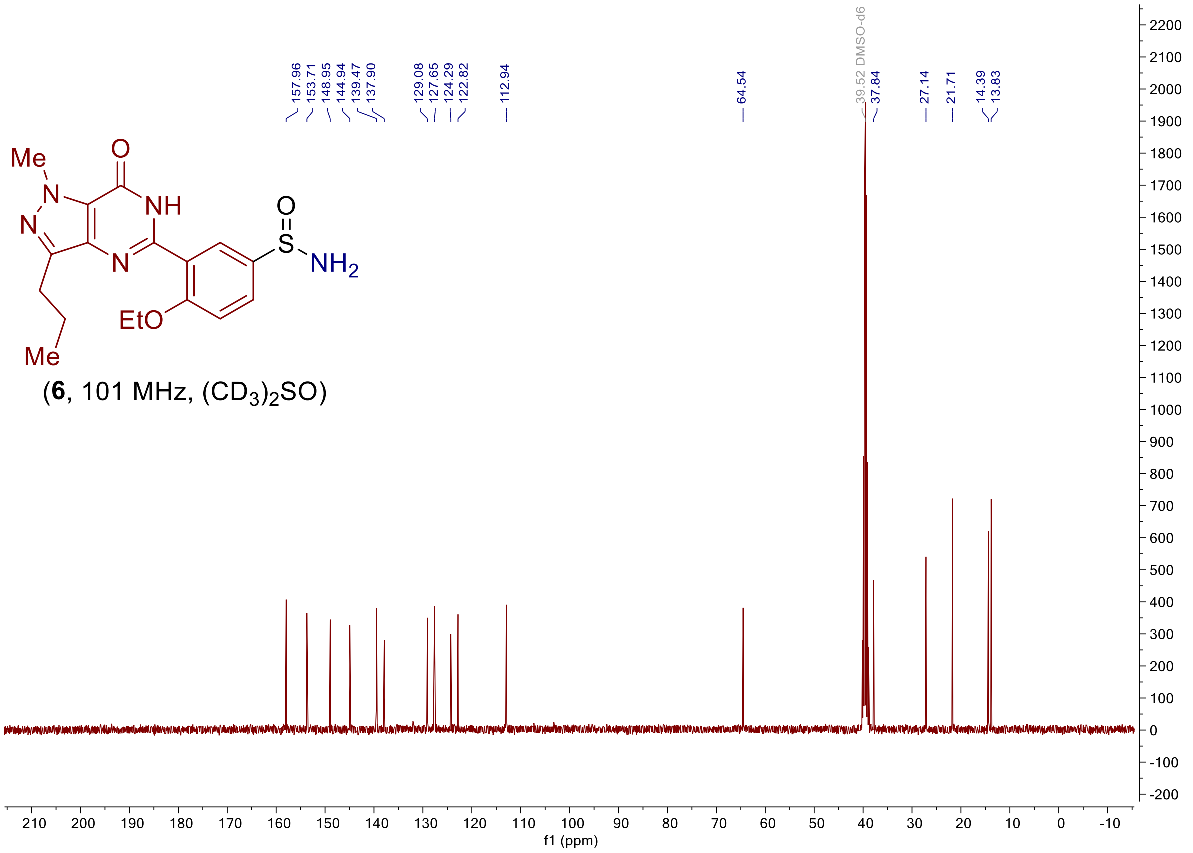
(6, 101 MHz, (CD3)2SO)
157.96 153.71 148.95 144.94 139.47 137.90
129.08 127.65 124.29 122.82
112.94
64.54
39.52 DMSO-d6 37.84
27.14 21.71 14.39 13.83
f1 (ppm)

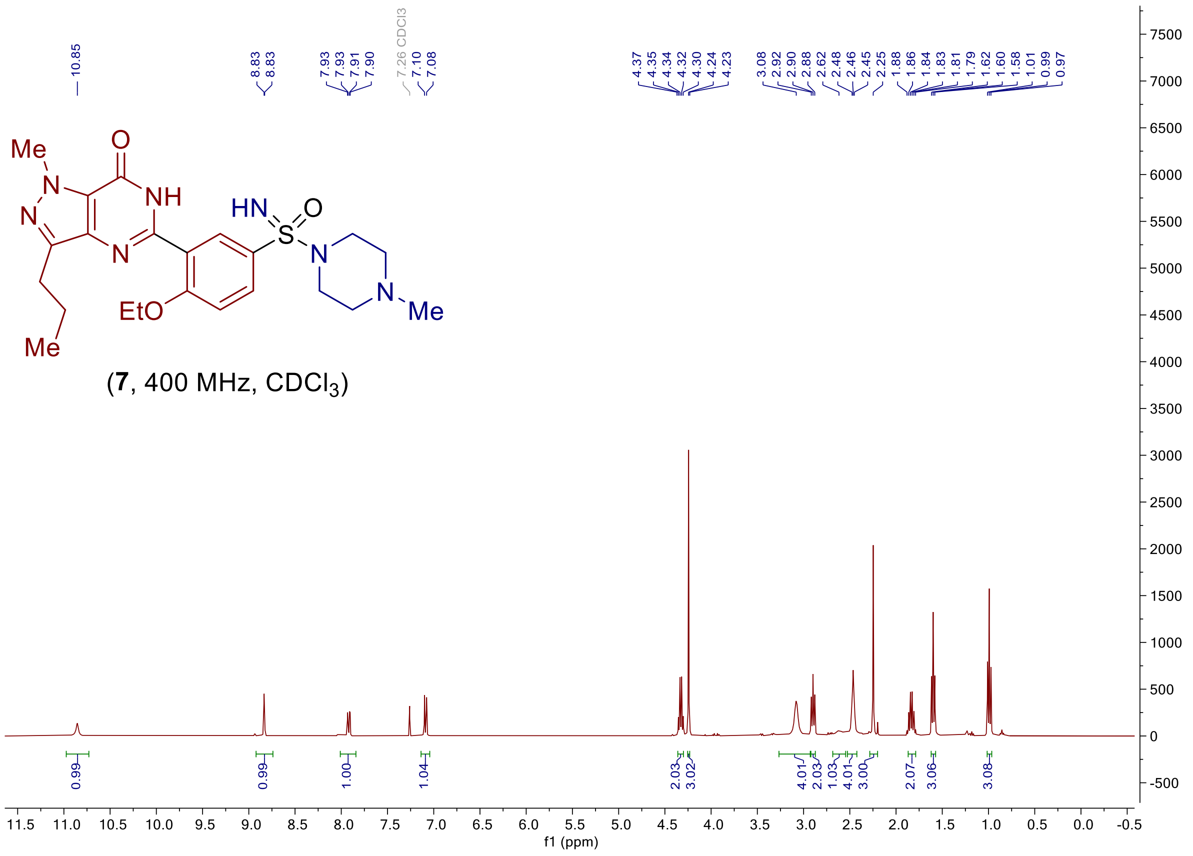

(**7**, 400 MHz, $CDCl_3$)

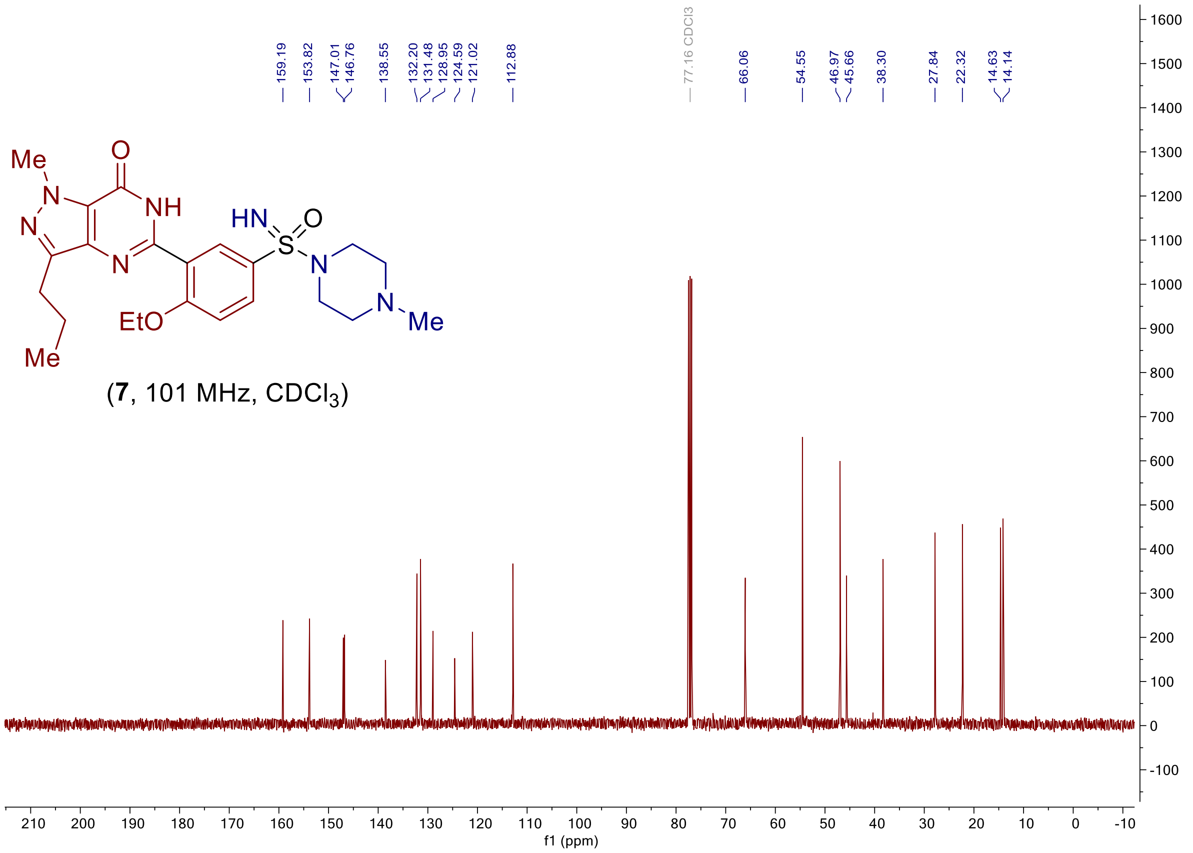

(**7**, 101 MHz, $CDCl_3$)
